# Total synthesis of dissectol A, using an enediolate-based Tsuji–Trost reaction†

**DOI:** 10.1039/d4sc01745e

**Published:** 2024-06-07

**Authors:** Ruben L. H. Andringa, Nittert Marinus, Daan V. Bunt, Elizabeth R. Haiderer, Robert B. Abramovitch, Christopher D. Brown, Kyu Y. Rhee, Martin D. Witte, Adriaan J. Minnaard

**Affiliations:** a Stratingh Institute for Chemistry, University of Groningen Nijenborgh 7, 9747 AG Groningen The Netherlands M.D.Witte@rug.nl A.J.Minnaard@rug.nl; b Department of Microbiology, Genetics and Immunology, Michigan State University East Lansing MI 48824 USA; c Weill Cornell Medicine, Division of Infectious Diseases 1315 York Avenue, Stitch Building New York NY10021 USA

## Abstract

Dissectol A is a rearranged terpene glycoside isolated from several flowering plants. Starting from glucose, the densely functionalized bicyclic structure has been prepared *via* site-selective oxidation and an intramolecular allylic alkylation reaction with an enediolate as the nucleophile. Despite earlier reports, dissectol A is not effective at inhibiting DevRS signaling in whole-cell *Mycobacterium tuberculosis* and does not inhibit growth of the bacterium.

## Abstract 2

The densely functionalized terpene glycoside dissectol A has been prepared *via* site-selective oxidation and an intramolecular Tsuji–Trost allylic alkylation reaction with an enediolate as the nucleophile.

## Graphical abstract

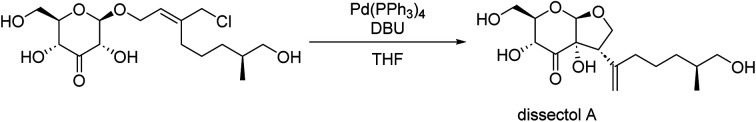

## Introduction

**Fig. 1:**
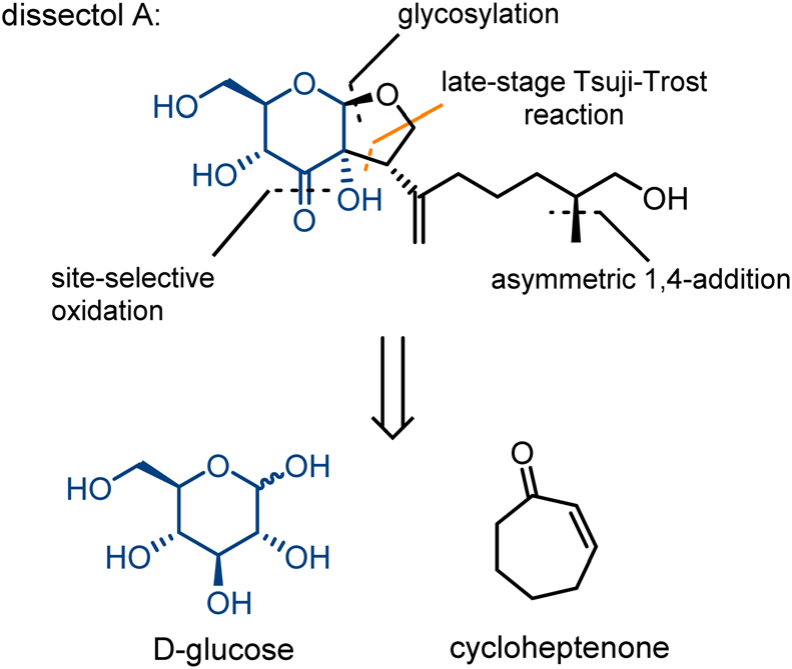
Dissectol A and its retrosynthesis strategy.

Dissectol A (Fig. 1) is a rearranged terpene glycoside, found in the aerial parts of the flowering plants *Incarvillea dissectifoliola* and *Patrinia scabiosifolia*.^1,2^ The latter is well-known and used in traditional Chinese medicine for the treatment of bacterial as well as fungal infections, and a range of inflammatory diseases.^3^

Dissectol A contains a peculiar *cis*-fused glucose-monoterpene bicycle and deviates in this way from regular terpene glycosides that are connected solely *via* a glycosidic linkage. The stereochemistry of this bicyclic motif has been independently determined beyond reasonable doubt for dissectol A and the related products 6,7-dehydrodissectol A and serratumin A, *via* 2D NMR-spectroscopy.^2,3,5^ The methyl branch in the chain of dissectol A evaded stereochemical elucidation.

**Scheme 1:**
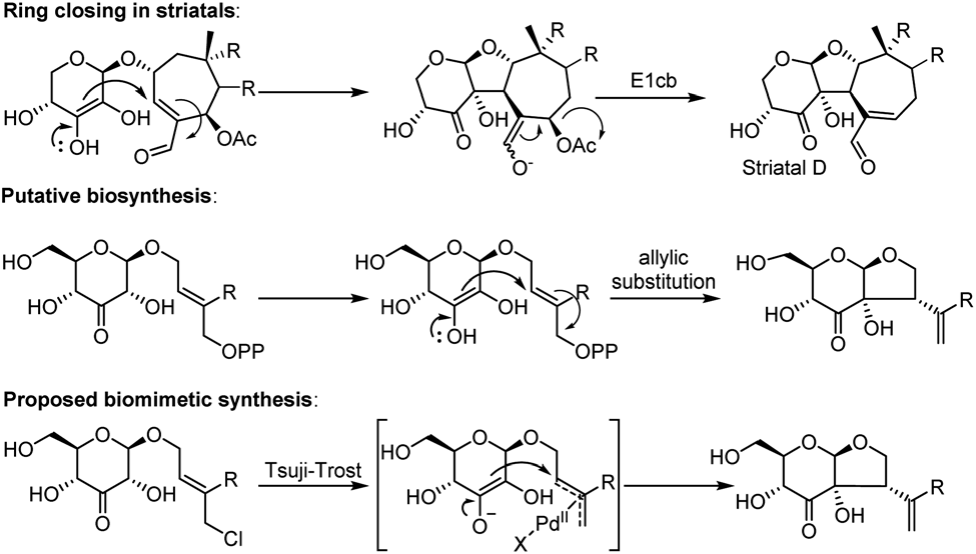
Ring-closing in striatal D, the putative biosynthesis of dissectol A and the biomimetic synthesis of dissectol A using a Tsuji–Trost reaction.

The biosynthesis of dissectol A has not been studied, and the only somewhat related natural products are the striatals, possessing a similar 6–5 fused ring-structure.^6–8^ It is assumed that in the biosynthesis of the striatals, an enediol or enediolate takes part in a conjugate addition reaction to the unsaturated aldehyde of the terpene residue (Scheme 1 for striatal D).^9^ A subsequent E1cb reaction provides the striatals. Alternatively, but less likely, the enediol takes part in an S_N_2′ allylic substitution reaction with acetate as the leaving group. For the biosynthesis of dissectol A, we postulate that an enediol or enediolate, originating from the 3-keto glucoside, performs a *bona fide* allylic substitution of (pyro)phosphate in the appropriate precursor, although neither the (pyro)phosphate precursor nor the corresponding alcohol has been reported.

Chen *et al.* studied the cytotoxic, antifungal, and antibacterial activity of dissectol A.^2^ The isolated compound did not inhibit the growth of the tumour cell lines P388 and A-549 and the fungi *Magnaporthe grisea* and *Penicillium avellaneum*. However, dissectol A did display a modest activity against *Mycobacterium tuberculosis* (*M. tb*). An independent *in silico* study suggested the *M. tb* dormancy regulating factor DevRS as a potential antibacterial target of dissectol A.^4^ The DevRS two-component regulatory system controls the metabolic state of the bacterium and is required to establish dormancy. Inhibition of DevRS signaling pathways decreases the survival of dormant *M. tb* and increases its vulnerability to antibiotics.^10,11^

Intrigued by the structure of dissectol A and its congeners, its reported activity against *M. tb*, and its proposed role as an inhibitor of DevRS, we set out to develop a synthesis strategy. Inspired by the biosynthesis of the striatals and our proposed biosynthesis of dissectol A, we hypothesized that the bicyclic core structure of dissectol A could be prepared from a 3-keto glucoside intermediate *via* a challenging late-stage Tsuji–Trost reaction (Scheme 1).^12,13^ Despite the widespread use and application of the Tsuji–Trost allylic alkylation reaction, the use of an enediol or enediolate as the nucleophile in this reaction is rare. This is probably because both carbon atoms in an enediol can act as a nucleophile, next to being an ambident nucleophile on both carbon and oxygen. Trost *et al.* reported using protected enediol allyl carbonates to prepare alpha-tertiary hydroxyaldehydes.^14,15^ In these substrates, the allyl fragment is part of the enol carbonate. This method has found application in synthesis.^16^ More recently, the Trost group reported the use of arylboronic acids as *in situ* templates for the intermolecular allylic alkylation of unprotected acyclic α-hydroxy ketones.^17^ Iridium-catalysed allylation of enediolates has been reported as well.^18,19^ Although the reported methods provided confidence in the approach, the conditions could not be applied as such in the synthesis at hand. The multiple hydroxy groups present and the possibility of enolization of the ketone in both directions complicate the system. On top of that, the stereochemistry of the side chain-branch is determined in this reaction.

A second key step is the selective oxidation of the C3-OH in the glucose residue in order to prepare the precursor for the Tsuji–Trost reaction. Although potentially the oxidation could be brought about using a protection–deprotection strategy to single out this hydroxy group, deprotection in the presence of a sensitive allylic system and an enolization-sensitive ketone would be very complicated. Therefore, we opted for a palladium-catalysed site-selective oxidation, a reaction we have studied intensively.^20–22^ Provided we could maintain the catalyst entirely in its Pd(ii) form, this oxidation reaction should be compatible with the allylic function which obviously is sensitive to Pd(0).

We report here a first synthesis of dissectol A, completing the elucidation of its structure. We also show that dissectol A is not an inhibitor of DevRS signaling using a reporter-based assay.

## Results and discussion

The synthesis commenced with a model substrate to study the Tsuji–Trost reaction. Glucosyl imidate donor 1 and isoprene acceptor 2 were prepared concisely from d-glucose and isoprene, respectively.^23^ After optimization, acceptable results in the glycosylation reaction were observed with BF_3_·OEt_2_ as a promotor at low temperature. This provided the desired glycoside 3 in 66% yield. Glucoside 3 was deacetylated with K_2_CO_3_ in methanol, to provide 4 in 58% yield. The moderate yield is due to the laborious column purification of the highly polar compound.

**Scheme 2:**
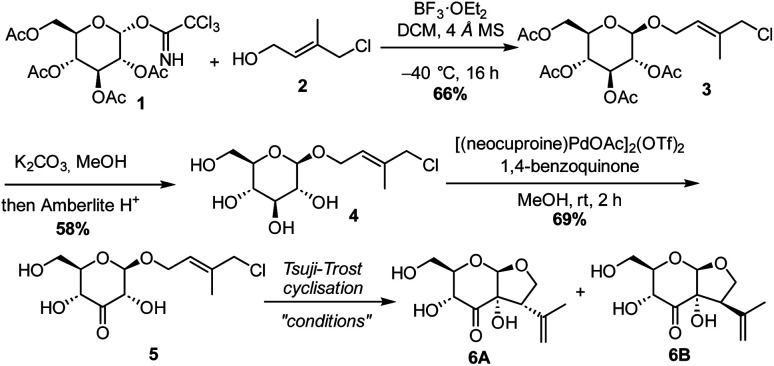
Synthesis of model substrate 5 and its use in a Tsuji–Trost reaction.

Subsequently, glucoside 4 was subjected to C3-selective Pd-catalysed oxidation. We were pleased to see full conversion to C3-ketone 5, whereas the allylic chloride was not affected. This demonstrates that the generated Pd(0) or palladium hydride is immediately re-oxidized by the benzoquinone (Scheme 2).

The group of Saicic has reported a Tsuji–Trost cyclisation reaction of an acyclic precursor containing an aldehyde and an allylic bromide to selectively form a 5-membered ring. Pyrrolidine in combination with DBU was used to form the enamine that acted as the nucleophile.^24^ Although employing these conditions we observed some formation of the desired product (Table 1, entry 1), it turned out that without pyrrolidine, so just using DBU as the base, full conversion of 5 was achieved (entry 2). This shows that in this case an enediolate or enediol is the nucleophilic intermediate and not an enamine. The NMR yield of the diastereomers 6A and 6B was improved further to 88% by increasing the catalyst loading to 30 mol% (entry 3). Although enolization of the ketone at C3 towards C4, either in the starting material or the products, possibly occurred, no epimerisation at C4 was observed. In the absence of the Pd catalyst, the reaction did not proceed (entry 4). To further optimise the reaction, various bromide and iodide salts were added in order to accelerate the Pd–allyl complex formation (entries 5–9). Sodium bromide (NaBr) and tetrabutylammonium bromide (TBAB) allowed the use of a catalyst loading of 10 mol%, with NaBr being slightly superior with respect to the yield of the desired diastereomer 6A. Compared to the high NMR yield (88%), the isolated yield of 6A/6B was lower (47%) due to the high polarity of the compounds and the separation and purification by column chromatography. The hydroxy-substituted bridgehead stereocenter is formed with complete selectivity. Due to the geometry of the enediolate intermediate, attack on the palladium complex only leads to the *cis*-fused ring system. We observed no conversion to the desired product when ligands typically used for asymmetric Tsuji–Trost reactions, such as DACH and ANDEN, were used. Steric crowding might hamper the reaction, and we found no additional measures to steer the product formation further to the desired isomer.

**Table 1:** Tsuji–Trost ring closing of 5

Entry^a^	[mol%] Pd(PPh_3_)_4_	Additive	Solvent	Yield^b^ (6A/6B)
1	10 mol%	Pyrrolidine	THF	nd
2	10 mol%	—	THF	69% (65 : 35)
3	30 mol%	—	THF	**88%** (69 : 31)
4	—	—	THF	X^c^
5	10 mol%	LiBr, 3 eq.	THF	<5%
6	10 mol%	ZnBr_2_, 1.5 eq.	THF	X^c^
7	10 mol%	NaBr, 3 eq.	THF	**74%** (66 : 34)
8	10 mol%	TBAB, 3 eq.	THF	**87%** (54 : 46)
9	10 mol%	NaI, 3 eq.	THF	<5%

a Conditions: 1.05 eq. of DBU. Reactions were stirred overnight at rt, under N_2_ atmosphere.

b Yield determined by quantitative NMR with 1,3,5-trimethoxybenzene as internal standard. Ratio of 6A : 6B is shown in parentheses.

c No product formed.

**Scheme 3:**
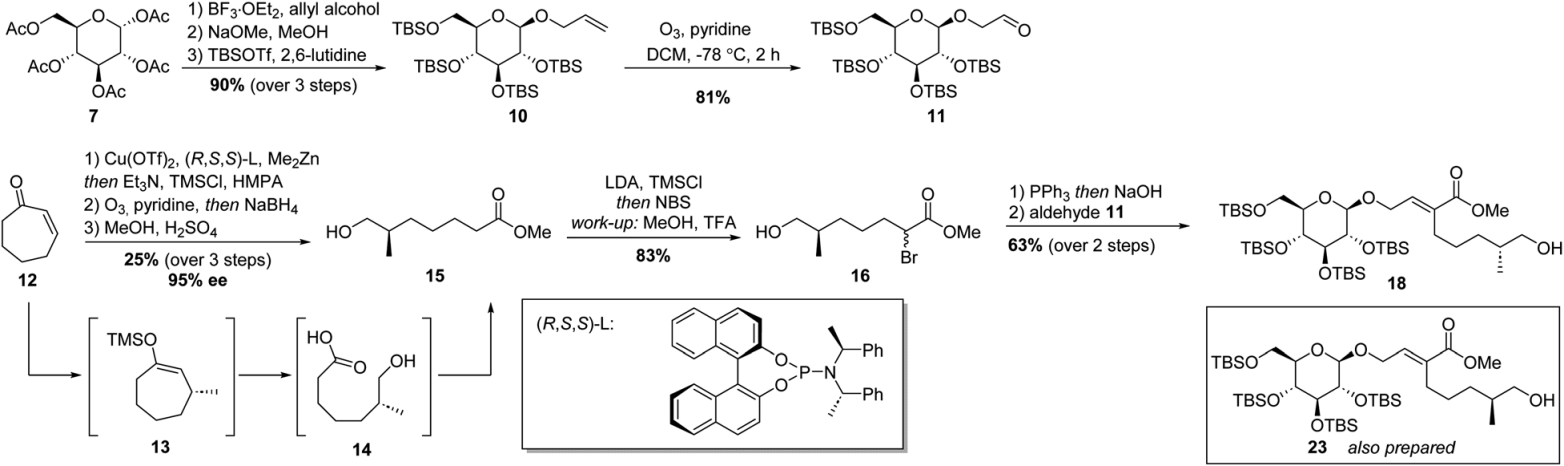
Synthesis of TBS protected glycoside 18.

With a sound method to construct the 6–5 glycoside-terpenoid ring system in hand, the total synthesis of dissectol A was started. Aldehyde 11 was prepared from per-acetyl glucose 7 (Scheme 3). A glycosylation reaction with allyl alcohol and 7 furnished the desired β-configured glucoside. The acetyl groups were exchanged for *tert*-butyldimethylsilyl groups (TBS), resulting in 10 in 90% yield over three steps. Aldehyde 11 was obtained *via* ozonolysis of 10 with pyridine as an additive. The presence of pyridine has been shown to directly generate ketones or aldehydes without the build-up of ozonides.^25^

As the stereochemistry of the methyl substituent in dissectol A was not known, both enantiomers of the terpenoid chain were prepared. Cycloheptenone 12 was subjected to a copper catalysed 1,4-addition reaction of dimethylzinc with the indicated (*R*,*S*,*S*)-phosphoramidite ligand, according to a previous report (Scheme 3).^26,27^ The resulting enolate was trapped with trimethylsilyl chloride (TMSCl) and the silyl enol ether was subjected to ozonolysis with pyridine as an additive. After reductive work-up with sodium borohydride (NaBH_4_) and esterification with methanol, *R*-configured alkyl chain 15 was obtained in 25% yield over 3 steps and in 95% ee. The enantiomeric excess was determined by derivatisation of 15 with *R*-Mosher's acid. The corresponding *S*-enantiomer was prepared with the enantiomer of the phosphoramidite ligand in the same fashion.

Initially, the hydroxyl group in 15 was protected with a *tert*-butyldimethyl silyl (TBS) group followed by α-bromination of the ester. However, this TBS group was cleaved during the formation of the Wittig reagent whereas the successive Wittig olefination with aldehyde 11 provided 18, as desired. Therefore, the procedure was adapted. The ester group in 15 was converted into the corresponding trimethylsilyl enol ether with lithium diisopropylamide (LDA) and TMSCl. Under these conditions, the hydroxyl group was protected simultaneously. Next, *N*-bromosuccinimide (NBS) was added to introduce the bromide substituent. After workup, in which partial deprotection of the hydroxyl group was observed, the crude was dissolved in methanol with a trace of trifluoroacetic acid (TFA) to provide bromo-ester 16 exclusively. Column chromatography gave 16 in 83% yield.

Bromide 16 was converted into the corresponding Wittig reagent by treatment with triphenylphosphine (PPh_3_) in water, followed by deprotonation with sodium hydroxide. Subsequent olefination with aldehyde 11 resulted in α,β-unsaturated ester 18 in 63% yield over two steps. Its diastereomer 23 with an *S*-configured methyl-branched stereocenter was prepared *idem dito*.

**Scheme 4:**
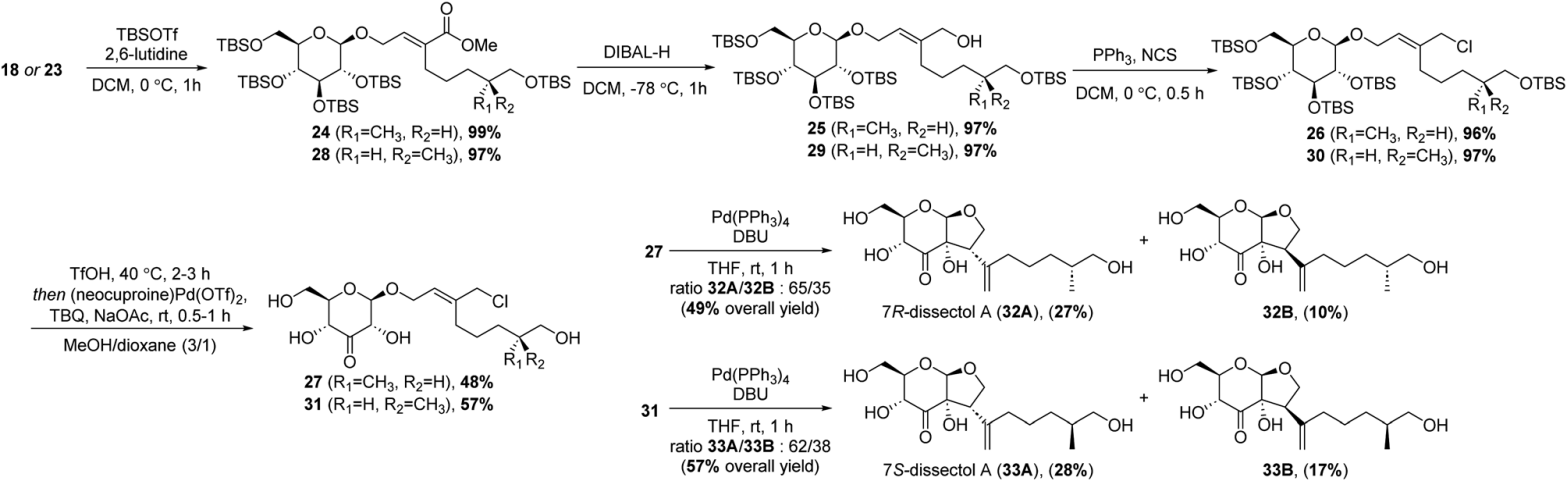
End-game of the synthesis of dissectol A.

The synthesis continued by protection of the remaining hydroxyl group in 18 using *tert*-butyldimethylsilyl triflate (TBSOTf) (Scheme 4). This was followed by reduction of the methyl ester to the corresponding allylic alcohol. This reduction was best achieved using DIBAL, and 25 was obtained in an excellent 97% yield. Conversion to the allylic chloride was achieved with PPh_3_ and *N*-chlorosuccinimide (NCS).

The silyl protecting groups were removed by methanolysis with catalytic amounts of trifluoromethanesulfonic acid (TfOH). After removal of the TBS groups, TfOH was neutralised by adding sodium acetate. Next, 2-*tert*-butyl-1,4-benzoquinone (TBQ) and palladium catalyst were added to perform a one-pot site-selective oxidation at the C3′-position. By adding NaOAc, the oxidation turned out to be slightly more selective. Unexpectedly, a minor side-reaction was observed, in which the primary hydroxyl group of the alkyl chain was oxidised and converted into the corresponding dimethyl acetal. Formation of this side product could be suppressed by using a smaller excess of TBQ. The ketones 27 and 31 were obtained in good yields of 48% and 57%, respectively.

With 27 and 31 in hand, the syntheses of dissectol A and its diastereomers was achieved in the final intramolecular Tsuji–Trost reaction. Cyclisation of 27 with Pd(PPh_3_)_4_ and DBU resulted in a mixture of the 7*R*-isomer of dissectol A (32A) and the corresponding C2-epimer 32B in an total yield of 49% (Scheme 4). These diastereomers could be partially separated by flash chromatography and pure samples of 32A and 32B were obtained in 27% and 10% yield, respectively, together with a mixed fraction (12%). The 7*S*-isomer of dissectol A (33A) and its C2-epimer 33B were obtained in a similar fashion from 31. The overall yields of 32A and 33A were 1.6% and 3.7%, respectively, with a longest linear sequence of 11 steps.

**Fig. 2:**
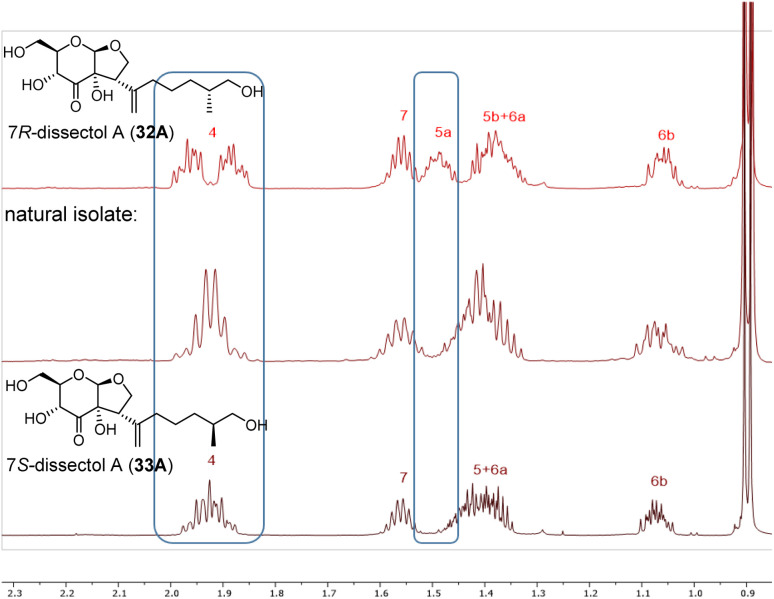
Stacked ^1^H-NMR spectra of synthetic 7*R*-dissectol A (top), natural isolate of dissectol A (middle), and synthetic 7*S*-dissectol A (bottom). The synthetic samples were measured in CD_3_OD on a 600 MHz spectrometer. The natural isolate was measured in CD_3_OD on a 400 MHz spectrometer and its ^1^H NMR spectrum was kindly provided by Prof. Chou.^5^

With the synthesis of both C7-isomers of dissectol A completed, it was investigated whether the available data of the natural isolate matched with the data of our synthetic isomers. The ^13^C NMR spectra of 32A, 33A and the natural isolate^5^ were identical in both pyridine-d5 and methanol-d4, which meant that the correct *cis*-fused bicycle had been synthesised. However, from these ^13^C NMR spectra it could not be concluded which isomer (32A or 33A) corresponded to the natural isolate. Fortunately, a small but significant difference in chemical shift was observed between the two epimers 32A and 33A in the ^1^H-NMR spectra measured in methanol-d4. This allowed to conclude that the 7*S*-configured isomer of dissectol A (33A) corresponds to the natural isolate (Fig. 2).

The specific optical rotations ([α]_D_) in methanol of synthetic 7*R*-dissectol A (32A), 7*S*-dissectol A (33A) and model substrate 6A are −30°, −42° and −25°, respectively. This does not correspond with that of the natural isolate (+125°). The latter value, in turn, deviates strongly from that of the closely related natural 6,7-dehydrodissectol A (−6.5).^5^ We argue that these conflicting values are not due to an incorrect absolute configuration assigned to dissectol A, but rather due to impurities in the natural isolate.

Both dissectol A 33A and its methyl-epimer 32A were assessed for uptake and growth inhibition of *M. tb* and for inhibitory activity during hypoxia in a DevRS reporter assay. The uptake assay was carried out with the laboratory strain H37Rv purchased from ATCC (product 27294) and showed that both compounds enter the cells and are metabolized (Fig. S1†). Neither epimer inhibited growth of *M. tb* under standard culture conditions even at high micromolar concentrations (Fig. S2†).

For the DevRS reporter assay, carried out with the Mtb CDC1551 strain provided by Cornell University, Ithaca, USA, the CDC1551(*hspX*′::GFP) reporter was used that has strong DevRS-dependent GFP fluorescence induced by hypoxia following growth for 6 days in a 96 well plate. GFP fluorescence to define DevRS signaling and optical density was measured to define growth. DevRS is not required for growth on the tested conditions, therefore, DevRS inhibitors in this assay will have inhibition of GFP fluorescence, but not inhibition of growth. Percent inhibition was normalized with a DMSO control (0% inhibition) and rifampin (100% inhibition of fluorescence and growth). As a positive control, a recent analog of DevRS signaling inhibitor HC106, MSU-43672, was used.^11^ We observed strong inhibition of reporter fluorescence in the samples treated with the control inhibitor (EC_50_ ∼ 200 nM, ESI†). However, no inhibition of the reporter fluorescence was observed with 33A and 32A. We therefore conclude that dissectol A is not an inhibitor of DevRS signaling in whole cell *M. tb*. It remains possible that dissectol is rapidly metabolized upon cell entry and that its metabolite is inactive, resulting in the lack of activity observed in whole cell *M. tb*.

## Conclusions

Dissectol A has been prepared from d-glucose and cycloheptenone and it stereochemistry has been further elucidated. The use of a palladium-catalysed site-selective alcohol oxidation is instrumental in this synthesis as it avoids a multistep protection–deprotection sequence. The observation that this oxidation reaction is compatible with the presence of an alkene and moreover an allylic chloride, means that this reaction is well-suited for application in natural product synthesis.

Key step in the synthesis is an intramolecular Tsuji–Trost reaction. Whereas ketone enolates are recognized nucleophiles in this reaction, the use of an unprotected enediolate is unprecedented, which means an extension of the scope of this well-known reaction. The reaction establishes two stereocenters. The hydroxy-substituted bridgehead stereocenter is formed with complete selectivity, whereas the stereocenter containing the side-chain is formed with moderate stereoselectivity towards the desired product.

Reports that dissectol A has activity against *M. tuberculosis*, in particular as inhibitor of the DevRS signalling, were not supported in this study. The synthetic (pure) material does not show inhibitory activity against whole cell *M. tb*.

## Data availability

The datasets supporting this article have been uploaded as part of the ESI.†

## Author contributions

All authors took part in the conceptualization of this study. R. L. H. A., N. M., and D. V. B. carried out the experimental work. E. R. H. and R. B. A. conducted the *M. tb* reporter assays. C. D. B. and K. Y. R. conducted the *M. tb* uptake and growth assays. R. L. H. A., N. M., M. D. W. and A. J. M. wrote the original draft of the manuscript. All authors contributed to the final version of the manuscript.

## Conflicts of interest

RBA is the owner of Tarn Biosciences, Inc., a company developing new antimycobacterial agents.

## Supplementary Material

SC-015-D4SC01745E-s001
