# Supplementary material for: Total synthesis of dissectol A, using an enediolate-based Tsuji–Trost reaction
Source: Chem Sci. 2024 Jun 7;15(27):10541–6. doi: 10.1039/d4sc01745e (PMC11234881; doi:10.1039/d4sc01745e)

Electronic Supplementary Material (ESI) for Chemical Science.
This journal is © The Royal Society of Chemistry 2024

Supporting Information

Total Synthesis of Dissectol A, using an Enediolate-based Tsuji-Trost Reaction

Table of contents

1. General Information S2
2. Experimental Procedures S3
3. Mtb Uptake, growth, and DevRS reporter assays S14
4. References S16
5. NMR Spectra S17

1. General Information

All solvents used for extraction, filtration and chromatography were of commercial grade, and used without further purification. Benzoquinone was purified by recrystallization from ethanol (15 g 1,4-benzoquinone from 45 mL). (neocuproine)Pd(CH_3CN)$_2$(OTf)$_2$[1] and (R)-2',2'-binaphthoyl-(*S*,*S*)-di-(1-phenylethyl)aminoylphosphine[2] were prepared according to literature procedures. (*S*)-2',2'-binaphthoyl-(*R*,*R*)-di-(1-phenylethyl)aminoylphosphine (cas: 497883-22-4) was purchased from Sigma-Aldrich. Celite (Celite® 545) was purchased from Merck. Other reagents were purchased from Sigma-Aldrich, TCI, Fluorochem and Acros and were used without further purification.

Flash chromatography was performed manually with silica (SiliaFlash P60, 230-400 mesh, Silicycle) or spherical silica (SiliaSphere PC 60A, Silicycle). TLC was performed on Merck silica gel 60, 0.25 mm plates and visualisation was done by staining with anisaldehyde stain (a mixture of AcOH (300 mL), H_2SO_4 (6 mL) and anisaldehyde (3 mL)) or potassium permanganate stain (a mixture of $KMnO_4$ (3 g), K_2CO_3 (10 g), and water (300 mL)). Ozone was generated with a Triogen LAB2B ozone generator.

^{1}H-, ^{13}C-NMR, NOESY were recorded on a Varian AMX400 (400, 100.6 MHz, respectively) or on a Bruker Avance NEO 600 (600, 150.9 MHz, respectively) at 25 °C using $CDCl_3$, CD_3OD or C_5D_5N as solvent. Chemical shift values are reported in ppm with the solvent resonance as the internal standard ($CDCl_3$: δ 7.26 for ^{1}H, δ 77.16 for ^{13}C, CD_3OD: δ 3.31 for ^{1}H, δ 49.00 for ^{13}C, C_5D_5N: δ 7.22 for ^{1}H, δ 123.87 for ^{13}C). Data are reported as follows: chemical shifts (δ), multiplicity (s = singlet, d = doublet, t = triplet, q = quartet, m = multiplet, br = broad, appt = apparent), coupling constants J (Hz), and integration. High resolution mass spectra (HRMS) were recorded on a Thermo Scientific LTQ Orbitrap XL. Optical rotations were measured on a Schmidt+Haensch polarimeter (Polartronic MH8) with a 10 cm cell (*c* given in g/100 mL) at ambient temperature (±20 °C).

2. Experimental Procedures

O-(2,3,4,6-tetra-*O*-acetyl-α-D-glucopyranosyl)trichloroacetimidate (1)

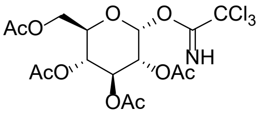

A solution of **8** (*vide infra*, 9.70 g, 27.8 mmol) in 97 mL dry DCM under nitrogen atmosphere was treated with trichloroacetonitrile (28 mL, 10 eq) and DBU (0.83 mL, 0.2 eq). The mixture was stirred at rt for 2 h, until TLC (pentane/EtOAc 1:1) indicated full conversion. The mixture was absorbed and dried on silica-gel and subsequently dry-loaded onto a silica-gel column for column chromatography (pentane/EtOAc 3/1). The purified product **1** was obtained as a pale yellow syrup (11.7 g, 86%). The product was stored at -80 °C as it was not entirely stable over time. **^{1}H NMR (400 MHz, $CDCl_3$)** δ 8.69 (s, 1H), 6.56 (d, *J* = 3.7 Hz, 1H), 5.57 (t, *J* = 9.9 Hz, 1H), 5.18 (dd, *J* = 10.2, 9.5 Hz, 1H), 5.13 (dd, *J* = 10.2, 3.7 Hz, 1H), 4.28 (dd, *J* = 12.3, 4.1 Hz, 1H), 4.21 (ddd, *J* = 10.3, 4.2, 2.1 Hz, 1H), 4.13 (dd, *J* = 12.3, 2.1 Hz, 1H), 2.08 (s, 3H), 2.05 (s, 3H), 2.03 (s, 3H), 2.02 (s, 3H). **^{13}C NMR (101 MHz, $CDCl_3$)** δ 170.5, 170.0, 169.8, 169.5, 160.8, 92.9, 70.0, 69.9, 69.7, 67.8, 61.4, 20.7, 20.6, 20.4. (CCl_3 signal not found). Spectral data matched with literature.[4a]

Preparation of *tert*-butyl hypochlorite.

An adapted literature procedure was used.[5] Bleach (500 mL, >4% NaOCl) was stirred in a 1 L round-bottom flask, protected from light and cooled in an ice/water bath. At 0 °C, a mixture of acetic acid (25 mL) and *tert*-butanol (37.5 mL) was added. The mixture was stirred vigorously for 10 min, after which it was poured into a separation funnel and the layers were partitioned. The organic layer was washed with aqueous Na_2CO_3 (10 wt%, 50 mL) and then with water (50 mL). The yellow organic product layer (14.0 mL, 31%) was collected and stored on $CaCl_2$ at 4 °C, protected from light.

1-chloro-2-methylbut-3-en-2-yl acetate and (*E*)-4-chloro-3-methylbut-2-en-1-yl acetate.

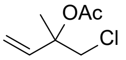

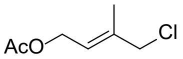

Isoprene (16 mL, 160 mmol, 1.3 eq.) was dissolved in acetic acid (70 mL) in a round-bottom flask. An ice/salt/water bath of -10 °C was prepared and set up in such a way that the reaction flask could be submerged when necessary. The flask was protected from light, and the mixture was stirred vigorously while *tert*-butyl hypochlorite (14 mL, 124 mmol) was added in portions. The flask was slowly lowered further into the cooling bath and stirred vigorously over the course of the addition, which prevented the mixture from solidifying. The reaction was quenched after 80 min, when TLC (pentane/Et_2O 95:5) showed the formation of two products (R_f 0.4 and 0.6). An aqueous NaOH solution was added to the stirring reaction mixture in small portions, until a pH of approx. 8 was reached (~49 g NaOH was added). The mixture was extracted with Et_2O, dried over anhydrous $MgSO_4$, filtered and concentrated under reduced pressure. The crude yellow mixture was purified by silica-gel column chromatography (pentane/Et_2O gradient: 100:0 to 90:10) yielding 1-chloro-2-methylbut-3-en-2-yl acetate (8.15 g, 28%) and (E)-4-chloro-3-methylbut-2-en-1-yl acetate (8.30 g, 31%), respectively, as pale yellow oils.

1-Chloro-2-methylbut-3-en-2-yl acetate (8.1 g, 34.4 mmol) was converted into (E)-4-chloro-3-methylbut-2-en-1-yl acetate by dissolution in acetic acid (19 mL), followed by addition of concentrated H_2SO_4 (125 mg, 1.27 mmol, 0.04 eq) and $CuSO_4 \cdot 5H_2O$ (290 mg, 1.16 mmol, 0.03 eq). The mixture was stirred at rt for 3 d. The resulting mixture was separated using the same method as described and yielded (E)-4-chloro-3-methylbut-2-en-1-yl acetate (4.0 g, 62%) as a pale yellow oil. Note that the products are volatile, so the solvent was not entirely removed. Characterisation of 1-chloro-2-methylbut-3-en-2-yl acetate: **^{1}H NMR (400 MHz, $CDCl_3$)** δ 6.03 (dd, *J* = 17.5, 11.0 Hz, 1H), 5.30 – 5.23 (m, 2H), 3.92 – 3.73 (m, 2H), 2.05 (s, 3H), 1.61 (s, 3H). **^{13}C NMR (101 MHz, $CDCl_3$)** δ 169.8, 138.5, 115.8, 80.9, 49.7, 22.2, 21.9. Characterisation of (E)-4-chloro-3-methylbut-2-en-1-yl acetate**:** **^{1}H NMR (400 MHz, $CDCl_3$)** δ 5.69 (tq, *J* = 6.8, 1.2 Hz, 1H), 4.62 (dd, *J* = 6.7, 0.8 Hz, 2H), 4.01 (d, *J* = 0.9 Hz, 2H), 2.06 (s, 3H), 1.82 (dt, *J* = 1.6, 0.8 Hz, 3H). **^{13}C NMR (101 MHz, $CDCl_3$)** δ 170.9, 137.0, 123.8, 60.8, 50.8, 20.9, 14.6. NMR characterisation matches literature.[5]

(*E*)-4-chloro-3-methylbut-2-en-1-ol (2)

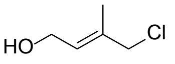

To a stirring mixture of (E)-4-chloro-3-methylbut-2-en-1-yl acetate (9.60 g, 59.0 mmol) in methanol (145 mL), a solution of Na_2CO_3 (17.5 g, 165 mmol, 2.8 eq.) in water (50 mL) was added. After 4.5 h stirring at rt, TLC (pentane/Et_2O 95:5) showed complete consumption of starting material. The reaction was diluted with water until a homogeneous mixture was obtained, which was extracted with Et_2O, dried over anhydrous $MgSO_4$, filtered and concentrated under reduced pressure. This crude was mixed with a small amount of water, and subsequently extracted with pentane to ensure removal of methanol. The pentane extract was concentrated, which

yielded **2** as a pale yellow oil (5.77 g, 81%). **^{1}H NMR (400 MHz, $CDCl_3$)** δ 5.74 (tp, *J* = 6.5, 1.2 Hz, 1H), 4.21 (t, *J* = 6.2 Hz, 2H), 4.02 (d, *J* = 0.8 Hz, 2H), 1.79 (dt, *J* = 1.5, 0.8 Hz, 3H), 1.35 (t, *J* = 5.5 Hz, 1H). **^{13}C NMR (101 MHz, $CDCl_3$)** δ 134.7, 128.8, 59.2, 51.3, 14.4. NMR characterisation matches literature.[5]

1-*O*-((*E*)-4-chloro-3-methylbut-2-enyl) 2,3,4,6-tetra-*O*-acetyl-β-D-glucose (3)

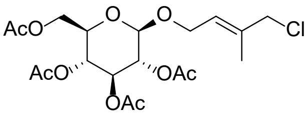

Glucosyl donor **1** (11.7 g, 23.7 mmol, 1.5 eq.) was dried by co-evaporation with anhydrous toluene under reduced pressure, twice, and subsequently stored under nitrogen atmosphere. A magnetic stirring bar, dried under vacuum, was added to the flask, followed by anhydrous DCM (470 mL). The mixture was stirred until full dissolution, after which activated 4 Å molecular sieves (~10 g) were added. The reaction was cooled to -20 °C with a cryostat, after which acceptor **2** (1.91 g, 15.8 mmol) was added. The mixture was allowed to stir for 45 min, after which $BF_3{\cdot}OEt_2$ (0.88 mL, 7.1 mmol, 0.45 eq.) was added dropwise to the stirring reaction mixture. After 3 h, the temperature was further decreased to -40 °C, and then stirred overnight. Cooling was removed and the mixture was allowed to warm to rt over the course of 1.5 h. The reaction was quenched by careful addition of Et_3N (0.99 mL, 7.1 mmol, 0.45 eq.). The mixture was filtered, the filtrate was adsorbed on Celite, dried, and subsequently loaded onto a silica-gel column for chromatography (pentane/EtOAc 3/1). Product **3** was obtained as a yellow syrup (4.68 g, 66%). **^{1}H NMR (400 MHz, $CDCl_3$)** δ 5.63 (tdd, *J* = 6.1, 2.4, 1.3 Hz, 1H), 5.19 (t, *J* = 9.5 Hz, 1H), 5.08 (t, *J* = 9.7 Hz, 1H), 4.98 (dd, *J* = 9.6, 7.9 Hz, 1H), 4.52 (d, *J* = 8.0 Hz, 1H), 4.33 (dd, *J* = 12.6, 5.9 Hz, 1H), 4.27 – 4.19 (m, 2H), 4.15 (dd, *J* = 12.3, 2.5 Hz, 1H), 4.01 (s, 2H), 3.67 (ddd, *J* = 10.0, 4.8, 2.5 Hz, 1H), 2.08 (s, 3H), 2.04 (s, 3H), 2.02 (s, 3H), 1.99 (s, 3H), 1.78 – 1.76 (m, 3H). **^{13}C NMR (101 MHz, $CDCl_3$)** δ 170.6, 170.3, 169.4, 169.3, 136.6, 125.0, 99.4, 72.8, 71.9, 71.2, 68.4, 65.1, 61.9, 50.9, 20.7, 20.7, 20.6, 20.6, 14.5. **HRMS** (ESI) calculated for $C_{19}H_{27}ClO_{10}$ ($[M+Na]^+$): 473.1185, found: 473.1181.

(*E*)-4-chloro-3-methylbut-2-enyl β-D-glucopyranoside (4)

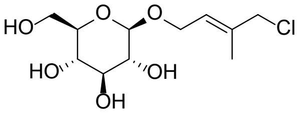

A mixture of **3** (765 mg, 1.70 mmol) and K_2CO_3 (470 mg, 3.39 mmol, 2.0 eq.) in methanol (17 mL) was stirred at rt for 1.5 h, whereupon TLC (DCM/MeOH 9/1) showed one product. Amberlite® IR120 H^+ form (washed with methanol) was added to the reaction mixture until pH ~6. The mixture was filtered and the filtrate was absorbed and dried on Celite and subsequently loaded onto a silica-gel column for chromatography (DCM/MeOH 90/10). Product **4** was obtained as a clear syrup (278 mg, 58%). **^{1}H NMR (400 MHz, CD_3OD)** δ 5.76 (ddq, *J* = 7.2, 6.0, 1.2 Hz, 1H), 4.41 (ddq, *J* = 12.6, 6.1, 0.9 Hz, 1H), 4.29 – 4.23 (m, 2H), 4.07 (d, *J* = 0.9 Hz, 2H), 3.87 (dd, *J* = 11.9, 2.0 Hz, 1H), 3.66 (dd, *J* = 12.0, 5.4 Hz, 1H), 3.36 – 3.33 (m, 1H), 3.29 – 3.23 (m, 2H), 3.17 (dd, *J* = 9.0, 7.8 Hz, 1H), 1.79 (d, *J* = 1.1 Hz, 3H). **^{13}C NMR (101 MHz, CD_3OD)** δ 135.7, 125.7, 101.9, 76.7, 76.6, 73.6, 70.2, 64.8, 61.3, 50.5, 13.1. **HRMS** (ESI): calculated for $C_{11}H_{19}ClO_6$ ($[M+Na]^+$): 305.0762, found: 305.0763.

(*E*)-4-chloro-3-methylbut-2-enyl β-D-3-oxo-glucopyranoside (5)

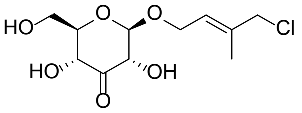

Glycoside **4** (275 mg, 0.97 mmol) and 1,4-benzoquinone (110 mg, 1.0 mmol, 1.05 eq.) were dissolved in methanol (20 mL), followed by addition of $[(neocuproine)PdOAc]_2OTf_2$ (51 mg, 49 µmol, 5 mol%). The mixture was stirred at rt for 2 h, whereupon TLC (DCM/MeOH 9/1) showed full consumption of **4**. The reaction mixture was absorbed on Celite, and subsequently dry-loaded onto a silica-gel column for chromatography (pentane/EtOAc 1/9). The product **5** was obtained as a pale yellow oil (187 mg, 69%). **^{1}H NMR (400 MHz, CD_3OD)** δ 5.77 (t, *J* = 6.7 Hz, 1H), 4.47 (dd, *J* = 12.6, 6.2 Hz, 1H), 4.38 (d, *J* = 7.9 Hz, 1H), 4.33 (dd, *J* = 12.6, 7.1 Hz, 1H), 4.22 (dd, *J* = 10.2, 1.8 Hz, 1H), 4.12 (dd, *J* = 7.9, 1.8 Hz, 1H), 4.08 (s, 2H), 3.94 (dd, *J* = 12.1, 2.1 Hz, 1H), 3.79 (dd, *J* = 12.1, 5.0 Hz, 1H), 3.33 – 3.28 (m, 1H), 1.80 (d, *J* = 1.2 Hz, 3H). **^{13}C NMR (101 MHz, CD_3OD)** δ 205.6, 136.2, 125.2, 103.3, 77.0, 76.8, 72.2, 65.1, 61.1, 50.4, 13.1. **HRMS** (ESI): calculated for $C_{11}H_{17}ClO_6$ ($[M+Na]^+$): 303.0606, found: 303.0607.

(3a*S*,5*R*,6*R*,7a*R*)-3a,5-dihydroxy-6-(hydroxymethyl)-3-(prop-1-en-2-yl)hexahydro-4*H*-furo[2,3-*b*]pyran-4-one (6A, 6B)

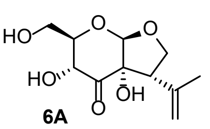

6A

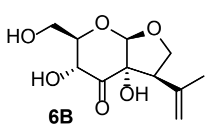

6B

Keto saccharide **5** (125 mg, 0.45 mmol) and NaBr (138 mg, 1.34 mmol, 3.0 eq.) were dried under vacuum and kept under nitrogen atmosphere. An oven-dried stirring bar and anhydrous THF (5 mL) were added, and the mixture was stirred at rt for 45 min. $Pd(PPh_3)_4$ (103 mg, 89 µmol, 20 mol%) was added and the mixture was allowed to stir at rt for 2 h. Next, a solution of DBU (70 µL, 0.47 mmol, 1.05 eq.) in anhydrous THF (5 mL) was added. After stirring for 16 h, methanol was added, whereupon which a bright yellow precipitate formed. The

solids were filtered off and the residue was washed with additional methanol. The filtrate was loaded on Celite and purified by silica column chromatography (DCM/MeOH gradient: 100/0, 99/1 and 98/2). This yielded a mixture of **6A** and **6B** as a clear oil (51 mg, 47%) in a ratio of 5/2, respectively. Characterisation of **6A**: **^{1}H NMR (400 MHz, methanol-*d*$_4$)** δ 4.97 (q, *J* = 1.3 Hz, 1H), 4.95 (s, 1H), 4.79 (d, *J* = 1.6 Hz, 1H), 4.58 (d, *J* = 10.2 Hz, 1H), 4.30 (t, *J* = 8.2 Hz, 1H), 4.20 (dd, *J* = 10.2, 7.8 Hz, 1H), 3.91 (dd, *J* = 12.0, 2.2 Hz, 1H), 3.82 (dd, *J* = 12.1, 4.6 Hz, 1H), 3.51 (t, *J* = 9.4 Hz, 1H), 3.39 (ddd, *J* = 10.2, 4.6, 2.2 Hz, 1H), 1.69 (s, 3H). **^{13}C NMR (101 MHz, methanol-*d*$_4$)** δ 207.5, 138.0, 113.8, 108.9, 86.2, 76.9, 70.8, 70.4, 60.9, 51.4, 22.0. Characterisation of **6B**: **^{1}H NMR (400 MHz, methanol-*d*$_4$)** δ 5.08 (dd, *J* = 3.0, 1.6 Hz, 2H), 4.98 (s, 1H), 4.43 – 4.31 (m, 2H), 4.18 (d, *J* = 10.3 Hz, 1H), 3.87 (dd, *J* = 12.1, 2.2 Hz, 1H), 3.71 (dd, *J* = 12.1, 4.9 Hz, 1H), 3.38 (ddd, *J* = 10.3, 4.9, 2.2 Hz, 1H), 2.97 (t, *J* = 7.6 Hz, 1H), 1.71 (s, 3H). **^{13}C NMR (151 MHz, methanol-*d*$_4$)** δ 207.6, 141.3, 115.4, 108.7, 89.0, 75.8, 73.6, 71.5, 61.2, 57.2, 23.0. **HRMS** (ESI): calculated for $C_{11}H_{16}O_6$ ($[M+Na]^+$): 267.0839, found: 267.0839.

General procedure for the Tsuji-Trost cyclizations in Table 1

Keto saccharide **5** (10 mg) was dried under vacuum and kept under nitrogen atmosphere in a 4 mL vial equipped with a septum. An oven-dried stirring bar was added, followed by an anhydrous solvent (0.5 mL) and the mixture was stirred for 1 h. The desired amount of $Pd(PPh_3)_4$ was added and stirring was continued for another h, after which DBU (1.05 eq.) dissolved in anhydrous solvent (0.5 mL) was added. After 16 h, the solvent was removed under reduced pressure and 1,3,5-trimethoxybenzene was added as the internal standard for quantitative NMR. Deuterated methanol (~0.6 mL) was added and a ^{1}H NMR measurement was performed (600 MHz, ns = 16, d1 = 60, at = 4.0) The yield and diastereomeric ratio were determined by integration. The yields are calculated relative to a qNMR measurement of the starting material **5**, serving as a standard.

D-glucose pentaacetate (7)

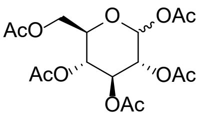

D-Glucose (10.0 g, 55.5 mmol) was dissolved in 120 mL anhydrous pyridine. The mixture was cooled to 0 °C and acetic anhydride (36.7 mL, 389 mmol, 7 eq.) was added in small portions. The resulting mixture was stirred for 16 h, during which it was allowed to warm up to rt. The stirring mixture was cooled down to 0 °C again, and subsequently diluted with cold water. The cold mixture was extracted with EtOAc, the combined organic layers were washed with aqueous HCl (1 M), aqueous $NaHCO_3$ (sat.) and water, dried over anhydrous Na_2SO_4, filtered and concentrated under reduced pressure to obtain **7** as a white solid (16.3 g, 75%). **^{1}H NMR (400 MHz, $CDCl_3$)** δ 6.33 (d, *J* = 3.7 Hz, 1H), 5.47 (t, *J* = 9.8 Hz, 1H), 5.14 (t, *J* = 9.8 Hz, 1H), 5.09 (dd, *J* = 10.3, 3.7 Hz, 1H), 4.27 (dd, *J* = 12.6, 4.2 Hz, 1H), 4.14 – 4.07 (m, 2H), 2.18 (s, 3H), 2.09 (s, 3H), 2.04 (s, 3H), 2.02 (s, 3H), 2.01 (s, 3H). **^{13}C NMR (101 MHz, $CDCl_3$)** δ 170.6, 170.2, 169.6, 169.4, 168.7, 89.1, 69.8, 69.8, 69.2, 67.9, 61.4, 20.9, 20.7, 20.6, 20.5, 20.4. (Only the signals of the α-product are reported, since the β-anomer was formed only in negligible amount.) NMR characterisation matches literature.[3]

2,3,4,5-tetra-O-acetyl-D-glucose (8)

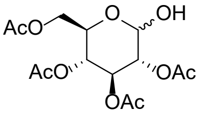

To a stirring solution of **7** (15.0 g, 38.4 mmol) in 110 mL dry THF under nitrogen atmosphere was added benzylamine (4.6 mL, 42.3 mmol, 1.1 eq.). The mixture was stirred at rt for 2 d, after which TLC (pentane/EtOAc 1:1) indicated full consumption of the starting material. The mixture was adsorbed on silica-gel and dried, and was subsequently dry-loaded onto a silica-gel column for column chromatography (pentane/EtOAc gradient: 7/3 to 5/5). Removal of the solvent yielded **8** as a brown syrup (13.0 g, 97%). The product was obtained as a mixture of α/β anomers, ratio: 3/1, respectively. Only the signals of the α-product are reported. **^{1}H NMR (400 MHz, $CDCl_3$)** δ 5.53 (t, *J* = 9.8 Hz, 1H), 5.47 (d, *J* = 3.6 Hz, 1H), 5.08 (t, *J* = 9.6 Hz, 1H), 4.91 (dd, *J* = 10.3, 3.6 Hz, 1H), 4.30 – 4.21 (m, 2H), 4.17 – 4.10 (m, 2H), 2.10 (s, 3H), 2.08 (s, 3H), 2.03 (s, 3H), 2.02 (s, 3H). **^{13}C NMR (101 MHz, $CDCl_3$)** δ 170.8, 170.1, 170.1, 169.6, 90.2, 71.0, 69.8, 68.4, 67.3, 61.9, 20.7, 20.7, 20.7, 20.6. NMR characterisation matches literature.[4]

1-*O*-Allyl 2,3,4,6-tetra-*O*-*tert*-butyldimethylsilyl-β-D-glucopyranoside (10)

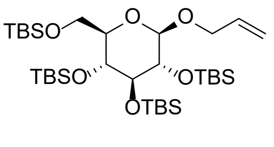

Allyl alcohol (5.4 mL, 79 mmol, 1.5 eq.) was added to a solution of **7** (20.5 g, 52.5 mmol, 1 eq) in anhydrous DCM (260 mL, 0.2 M) under N_2 atmosphere. The mixture was cooled to 0 °C followed by slow addition of $BF_3{\cdot}Et_2O$ (6.8 mL, 55 mmol, 1.05 eq) over approximately 5 min. The mixture was stirred for 20 h at rt, then cooled to 0 °C and $NaHCO_3$ (6.5 g, in 150 mL water,

1.5 eq.) was added. The mixture was allowed to phase separate, and the aqueous layer was extracted once with DCM (150 mL). The combined organic layers were washed with water (200 mL), dried with $MgSO_4$, filtered and concentrated *in vacuo*. The crude (20.4 g) was dissolved in MeOH (175 ml, 0.3 M) followed by addition of sodium (120 mg, 5.3 mmol, 0.1 eq.). The mixture was stirred for 16 h at rt and then concentrated *in vacuo*. The resulting yellow foam was dissolved in anhydrous DCM (66 mL, 0.8 M) and 2,6-lutidine (70 mL, 600 mmol, 11.4 eq.). The mixture was cooled to 0 °C followed by addition of TBDMS triflate (97 mL, 420 mmol, 8 eq.). The mixture was stirred at 40 °C for 19 h. Additional DCM (150 mL) was added and the mixture was washed twice with water (200 mL), with aqueous $CuSO_4$ (200 mL, sat.) and again with water (200 mL). The organic layer was dried with Na_2SO_4, filtered and concentrated *in vacuo*. The crude was purified by column chromatography (silica, eluent: 3% Et_2O/pentane) and gave product **10** (31.8 g, 90% over three steps) as a colourless syrup. **^{1}H NMR (400 MHz, $CDCl_3$)** δ 6.00 – 5.88 (m, 1H), 5.31 – 5.22 (m, 1H), 5.17 – 5.12 (m, 1H), 4.74 (d, *J* = 6.7 Hz, 1H), 4.38 (dd, *J* = 12.4, 5.3 Hz, 1H), 3.97 (dd, *J* = 12.4, 6.2 Hz, 1H), 3.92 (d, *J* = 2.9 Hz, 1H), 3.82 – 3.69 (m, 4H), 3.60 (d, *J* = 6.7 Hz, 1H), 0.91 – 0.84 (m, 36H), 0.10 – 0.02 (m, 24H). **^{13}C NMR (101 MHz, $CDCl_3$)** δ 134.6, 117.1, 101.1, 82.6, 79.3, 77.9, 70.2, 64.2, 26.1, 26.1, 26.0, 25.9, 18.5, 18.2, 18.2, 18.0, -4.0, -4.2, -4.4, -4.6, -4.6, -4.9, -5.1. Characterization matches literature.[6]

2-Oxoethyl 2,3,4,6-tetra-*O*-*tert*-butyldimethylsilyl-β-D-glucopyranoside (11)

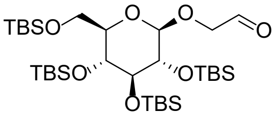

A procedure from literature was adapted.[7] Allyl ether **10** (12.3 g, 19.5 mmol, 1 eq) was placed in a Schlenk flask and dissolved in anhydrous DCM (200 mL, 0.1 M) under N_2 atmosphere. To the mixture was added anhydrous pyridine (4.7 mL, 58 mmol, 3 eq) followed by cooling to -78 °C. Next, ozone was bubbled through the mixture for 2 h until a blue colour persisted. Nitrogen was bubbled through the mixture which subsequently was allowed to warm to rt. The mixture was washed once with water (200 mL), dried with $MgSO_4$, filtered and concentrated *in vacuo*. The crude was purified by column chromatography (120 mL silica, eluent: 10% Et_2O/pentane) to obtain aldehyde **11** (10.7 g, 81%) as a colourless syrup. **^{1}H NMR (400 MHz, $CDCl_3$)** δ 9.79 – 9.77 (m, 1H), 4.75 (d, *J* = 6.5 Hz, 1H), 4.20 (dd, *J* = 17.3, 1.8 Hz, 1H), 4.08 (dd, *J* = 17.3, 1.1 Hz, 1H), 3.92 (d, *J* = 3.0 Hz, 1H), 3.83 – 3.65 (m, 5H), 0.94 – 0.83 (m, 36H), 0.13 – 0.02 (m, 24H). **^{13}C NMR (101 MHz, $CDCl_3$)** δ 202.0, 102.9, 82.7, 79.0, 77.7, 74.7, 70.2, 64.0, 26.1, 26.0, 25.9, 18.5, 18.2, 18.1, 18.0, -4.1, -4.2, -4.4, -4.6, -4.6, -4.7, -5.2, -5.2. Characterization matches literature.[6]

Methyl (*R*)-7-hydroxy-6-methylheptanoate (15)

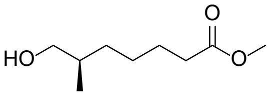

A procedure from literature was adapted.[8,9] Copper(II) triflate (172 mg, 0.48 mmol, 7 mol%) and (*R*)-2,2'-binaphthoyl-(*S*,*S*)-di-(1-phenylethyl)aminoylphosphine (514 mg, 0.95 mmol, 14 mol%) were dissolved in anhydrous DCM (200 mL) and the mixture was stirred at rt for 30 min under N_2 atmosphere. Subsequently, the solution was cooled to −25 °C and Me_2Zn (85 mL, 1.2 M in toluene, 102 mmol, 1.5 eq) was added dropwise over 10 min. After stirring for 5 min, a solution of 2-cyclohepten-1-one (7.6 ml, 68.1 mmol, 1 eq.) in anhydrous DCM (150 mL) was added over a period of 5 h with a syringe pump. The resulting solution was stirred at −25 °C overnight, after which Et_3N (28 mL, 204 mmol, 3 eq.), TMS chloride (43 mL, 340 mmol, 5 eq.) and HMPA (59 mL, 340 mmol, 5 eq) were added. The resulting mixture was stirred for 3 h while slowly warming to rt. The reaction mixture was quenched with water (400 mL) and extracted twice with Et_2O (1 L and 250 mL). The combined organic layers were washed with brine (200 mL) and then concentrated *in vacuo* (≤40 °C). The crude was quickly purified by column chromatography (240 mL silica priorly deactivated with Et_3N, eluent: 600 mL 30% Et_2O/pentane) resulting in impure TMS enol ether as a yellow oil, which was used in the next step without further purification. The crude TMS enol ether was split in half and subjected to ozonolysis in two batches. Half of the crude (7 g, 35 mmol, 1 eq.) was placed in a Schlenk flask and dissolved in anhydrous DCM (350 mL) under N_2 atmosphere. Pyridine (16 mL, 105 mmol, 3 eq.) was added to the mixture which was then cooled to -78 °C. Next, ozone was bubbled through the mixture for 2-3 h until a blue colour persisted. Nitrogen was bubbled through the mixture and the mixture was allowed to warm to rt. This process was repeated with the second batch. The reaction mixtures were combined and transferred to a 2 L round-bottom flask with MeOH (300 mL). Next $NaBH_4$ (10.1 g, 270 mmol, 4 eq) was added in small batches over 10 min. The mixture was stirred for 1.5 h and then quenched by addition of HCl (500 mL, 3 M in water). The layers were partitioned and the aqueous layer was extracted with DCM (2 × 250 mL). The combined organic layers were dried with Na_2SO_4, filtered and concentrated *in vacuo*. The crude carboxylic acid was then dissolved in MeOH (126 mL) followed by addition of concentrated H_2SO_4 (0.36 mL, 6.8 mmol, 0.1 eq.). The resulting mixture was refluxed for 24 h and then quenched with aqueous $NaHCO_3$ (50 mL, sat.). Additional water (150 mL) was added and

the mixture extracted with Et_2O (5 × 125 mL). The combined organic layers were washed with brine (100 mL), dried with Na_2SO_4, filtered and concentrated *in vacuo*. The crude was purified by column chromatography (550 mL silica, eluent: 5.5 L 20% EtOAc/pentane) to obtain methyl ester **15** (2.96 g, 25% yield over 3 steps) as a yellow oil. **^{1}H NMR (400 MHz, $CDCl_3$)** δ 3.65 (s, 3H), 3.47 (dd, *J* = 10.5, 5.9 Hz, 1H), 3.40 (dd, *J* = 10.5, 6.4 Hz, 1H), 2.30 (t, *J* = 7.5 Hz, 2H), 1.66 – 1.53 (m, 4H), 1.46 – 1.22 (m, 3H), 1.16 – 1.06 (m, 1H), 0.89 (d, *J* = 6.7 Hz, 3H). **^{13}C NMR (101 MHz, $CDCl_3$)** δ 174.4, 68.3, 51.6, 35.7, 34.1, 32.8, 26.6, 25.3, 16.6. **HRMS** (ESI) calculated for $C_9H_{19}O_3$ ($[M+H]^+$): 175.1329, found: 175.1325. **$[\alpha]_D$** +9.3° (*c* 1.5, $CHCl_3$).

Methyl (*R*)-7-hydroxy-6-methylheptanoate (*R*)-Mosher's ester derivative

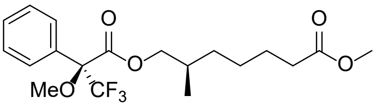

Alcohol **15** (3.9 mg, 22 µmol, 1 eq.) was dissolved in anhydrous pyridine (0.2 mL) under N_2 atmosphere. (*R*)-(-)-MTPA-Cl (10 µL, 56 µmol, 2.5 eq, Mosher's chloride) was added and the reaction mixture was stirred overnight at rt. Aqueous $NaHCO_3$ (0.2 mL, sat.) and water (1 mL) were added and the mixture was extracted with Et_2O (3 × 1 mL). The combined organic layers were washed with brine (2 mL), dried over $MgSO_4$, filtered and concentrated *in vacuo*. The residue was filtered over a small pad of silica (eluent: 40% Et_2O/pentane, R_f=0.8) to obtain Mosher's ester (8 mg, 92%). To remove residual solvents, the product was co-evaporated with $CDCl_3$. The diastereomeric excess was determined by ^{1}H NMR and turned out to be 95%. **^{1}H NMR (600 MHz, $CDCl_3$)** δ 7.55 – 7.48 (m, 2H), 7.44 – 7.36 (m, 3H), 4.22 (dd, *J* = 10.7, 5.7 Hz, 0.023H), 4.17 (dd, *J* = 10.7, 6.4 Hz, 0.977H), 4.13 (dd, *J* = 10.7, 5.7 Hz, 0.977H), 4.07 (dd, *J* = 10.8, 6.5 Hz, 0.023H), 3.66 (s, 3H), 3.55 (s, 3H), 2.28 (t, *J* = 7.5 Hz, 2H), 1.89 – 1.80 (m, *J* = 6.6 Hz, 1H), 1.62 – 1.52 (m, 2H), 1.39 – 1.12 (m, 4H), 0.92 (d, *J* = 6.7 Hz, 3H). **^{13}C NMR (151 MHz, $CDCl_3$)** δ 174.2, 166.8, 132.5, 129.7, 128.5, 127.5, 123.49 (q, *J* = 288.5 Hz), 84.77 (q, *J* = 27.6 Hz), 71.2, 55.6, 51.6, 34.0, 32.8, 32.4, 26.4, 25.1, 16.8.

Methyl (2R/S,6*R*)-bromo-7-hydroxy-6-methylheptanoate (16)

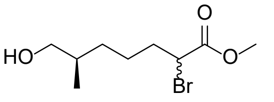

A fresh solution of LDA was prepared by adding *n*-BuLi (20 mL, 1.6 M in hexane, 32.0 mmol, 2.5 eq.) dropwise to a solution of diisopropylamine (4.5 mL, 32.0 mmol, 2.5 eq.) in anhydrous THF (32 mL) at -40 °C under N_2 atmosphere. The mixture was stirred for 30 min at -40 °C and then cooled to -78 °C. Meanwhile a solution of methyl ester **15** (2.23 g, 12.8 mmol, 1 eq) and TMS chloride (5.3 mL, 38.4 mmol, 3 eq) in anhydrous THF (32 mL) was prepared and added dropwise to the LDA solution over 15 min. Stirring was continued for 1 h at -78 °C whereupon cooling was removed and NBS (2.85 g, 16.0 mmol, 1.25 eq) was added under a stream of N_2. The mixture was stirred for 1 h at 0 °C followed by addition of aqueous $NaHCO_3$ (100 mL, sat.) and Na_2SO_3 (1.6 g). The mixture was extracted with Et_2O (3 × 100 mL) and the combined layers were washed with brine (100 mL), dried with Na_2SO_4, filtered and concentrated *in vacuo*. The crude was still partially TMS protected, hence it was dissolved in MeOH (64 mL) followed by addition of TFA (0.22 mL, 2.9 mmol, 23 mol%). The mixture was stirred for 1 h at rt, concentrated *in vacuo* and co-evaporated with toluene. The resulting crude was purified by column chromatography (160 mL silica, eluent: 1.5 L 20% EtOAc/pentane) to obtain bromo ester **16** (2.67 g, 83%) as a yellow oil and as a diastereomeric mixture. **^{1}H NMR (400 MHz, $CDCl_3$)** δ 4.23 (t, *J* = 7.3 Hz, 1H), 3.77 (s, 3H), 3.53 – 3.44 (m, 1H), 3.43 (dd, *J* = 10.5, 6.2 Hz, 1H), 2.13 – 1.91 (m, 2H), 1.68 – 1.57 (m, 1H), 1.56 – 1.23 (m, 3H), 1.19 – 1.07 (m, 1H), 0.91 (d, *J* = 6.7 Hz, 3H). **^{13}C NMR (101 MHz, $CDCl_3$)** δ 170.5, 170.5, 68.2, 68.1, 53.1, 53.1, 45.8, 45.7, 35.6, 35.6, 35.2, 35.1, 32.4, 32.3, 24.8, 24.8, 16.6, 16.5. Due to difficult ionisation, an HRMS could not be obtained. **$[\alpha]_D$** +9.0° (*c* 1.0, $CHCl_3$).

(*R*,*E*)-8-hydroxy-3-(methoxycarbonyl)-7-methyl-2-octenyl 2,3,4,6-tetra-*O*-*tert*-butyldimethylsilyl-β-D-glucopyranoside (18)

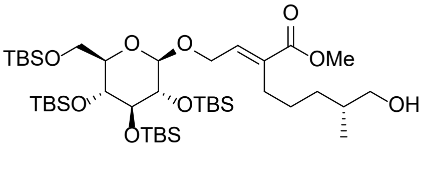

A mixture of bromo ester **16** (2.67 g, 10.6 mmol, 1 eq.), PPh_3 (2.91 g, 11.1 mmol, 1.05 eq) and water (21 mL, 0.5 M) was stirred and heated to 90 °C for 2 d under N_2 atmosphere. Thereafter the mixture was cooled to 0 °C and NaOH (1.27 g, 32 mmol, 3 eq) and DCM (10 mL) were added. The resulting suspension was stirred thoroughly for 15 min, after which more water (10 mL) was added. The emulsion was extracted with DCM (3 × 10 mL) and the combined organic layers were dried with Na_2SO_4, filtered and concentrated *in vacuo*. The crude Wittig reagent was used in the next step without further purification. The Wittig reagent (1 eq) and aldehyde **11** (7.3 g, 10.7 mmol, 1.02 eq) were dissolved in anhydrous DCM under N_2 atmosphere and then stirred at 35 °C for 18 h. The mixture was concentrated *in vacuo* and purified by column chromatography (480 mL silica, eluents: 1 L 10% Et_2O/pentane, then 2

L 20% Et_2O/pentane, then 1 L 25% Et_2O/pentane and finally 2 L 30% Et_2O/pentane) to obtain α,β-unsaturated ester **18** (5.59 g, 63% yield over 2 steps) as a colourless oil. **^{1}H NMR (400 MHz, $CDCl_3$)** δ 6.84 (t, *J* = 6.0 Hz, 1H), 4.73 (d, *J* = 6.6 Hz, 1H), 4.52 (dd, *J* = 14.1, 5.5 Hz, 1H), 4.23 (dd, *J* = 14.1, 6.6 Hz, 1H), 3.92 (d, *J* = 2.9 Hz, 1H), 3.84 – 3.72 (m, 7H), 3.59 (d, *J* = 6.6 Hz, 1H), 3.49 (dt, *J* = 11.5, 5.8 Hz, 1H), 3.42 (dt, *J* = 10.5, 6.0 Hz, 1H), 2.30 – 2.22 (m, 2H), 1.69 – 1.56 (m, 1H), 1.50 – 1.34 (m, 3H), 1.17 – 1.07 (m, 1H), 0.94 – 0.84 (m, 39H), 0.12 – 0.03 (m, 24H). **^{13}C NMR (101 MHz, $CDCl_3$)** δ 167.9, 138.5, 133.5, 101.3, 82.7, 79.1, 77.8, 70.2, 68.3, 65.4, 64.2, 51.9, 35.7, 33.0, 27.6, 26.6, 26.1, 26.0, 25.9, 18.4, 18.2, 18.1, 17.9, 16.7, -3.9, -4.2, -4.4, -4.6, -4.7, -4.7, -5.1. **HRMS** (ESI) calculated for $C_{41}H_{86}O_9Si_4Na$ ($[M+Na]^+$): 857.5241, found: 857.5267. **[α]*D*** -4.3° (*c* 1.0, $CHCl_3$)

Methyl (*S*)-7-hydroxy-6-methylheptanoate (19)

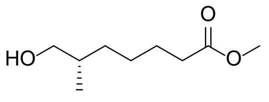

A procedure from literature was adapted.[8,9] Copper(II) triflate (60 mg, 0.17 mmol, 0.9'mol%) and (*S*)-2,2'-binaphthoyl-(*R*,*R*)-di-(1-phenylethyl)aminoylphosphine (180 mg, 0.33 mmol, 1.4 mol%) were dissolved in anhydrous DCM (55 mL) and the mixture was stirred at rt for 30 min under N_2 atmosphere. Subsequently, the solution was cooled to −25 °C and Me_2Zn (24 mL, 1.2 M in toluene, 29 mmol, 1.5 eq) was added dropwise over 10 min. After stirring for an additional 5 min, a solution of 2-cyclohepten-1-one (2.13 g, 19.3 mmol, 1 eq.) in anhydrous DCM (40 mL) was added over a period of 5 h with a syringe pump. The resulting solution was stirred at −25 °C overnight, after which Et_3N (8.1 mL, 58 mmol, 3 eq), TMS chloride (12.3 mL, 96 mmol, 5 eq) and HMPA (16.9 mL, 96 mmol, 5 eq) were added. The resulting mixture was stirred for 3 h while slowly warming to rt. The reaction mixture was quenched with water (200 mL) and extracted twice with Et_2O (1 L and 100 mL). The combined organic layers were concentrated *in vacuo* (≤40 °C) and the crude was quickly purified by column chromatography (80 mL silica previously deactivated with Et_3N, eluent: 300 mL 30% Et_2O/pentane) resulting in impure TMS enol ether as a yellow oil, which was used in the next step without further purification. The crude TMS enol ether was dissolved in DCM (39 mL) and MeOH (39 mL) and then cooled to -78 °C under N_2 atmosphere. Next, ozone was bubbled through the mixture for 2 h until a blue colour persisted. Nitrogen was bubbled through the mixture and $NaBH_4$ (7.3 g, 193 mmol, 10 eq) was added in small batches over 10 min. The mixture was allowed to slowly warm to rt and after 1 h the cooling bath was removed. Slowly warming to rt was continued and the mixture was stirred thoroughly overnight. Thereafter, it was quenched by slow addition of HCl (60 mL, 5 M in water). After stirring for 2 h, additional HCl (300 mL, 2 M in water) was added and the mixture was extracted with DCM (5 × 50 mL). The combined organic layers were dried with Na_2SO_4, filtered and concentrated *in vacuo*. The crude carboxylic acid was then dissolved in MeOH (39 mL) followed by addition of concentrated H_2SO_4 (0.21 mL, 3.8 mmol, 0.2 eq). The resulting mixture was refluxed for 24 h and then concentrated *in vacuo* to remove most MeOH. Aqueous $NaHCO_3$ (50 mL, sat.) was added and the mixture was extracted with Et_2O (3 × 50 mL). The combined organic layers were washed with brine (50 mL), dried with Na_2SO_4, filtered and concentrated *in vacuo*. The crude was purified by column chromatography (90 mL silica, eluents: 500 mL 20% EtOAc/pentane and then 200 mL 25% EtOAc/pentane) to obtain methyl ester **19** (1.49 g, 44% yield over 3 steps) as a yellow oil. **^{1}H NMR (400 MHz, $CDCl_3$)** δ 3.66 (s, 3H), 3.53 – 3.46 (m, 1H), 3.46 – 3.39 (m, 1H), 2.32 (t, *J* = 7.5 Hz, 2H), 1.67 – 1.56 (m, 3H), 1.48 – 1.24 (m, 4H), 1.18 – 1.08 (m, 1H), 0.91 (d, *J* = 6.7 Hz, 3H). **^{13}C NMR (101 MHz, $CDCl_3$)** δ 174.4, 68.4, 51.6, 35.7, 34.2, 32.8, 26.6, 25.3, 16.7. **HRMS** (ESI) calculated for $C_9H_{19}O_3$ ($[M+H]^+$): 175.1329, found: 175.1325. **[α]*D*** -8.8° (*c* 1.0, $CHCl_3$).

Methyl (*S*)-7-hydroxy-6-methylheptanoate (*R*)-Mosher's ester derivative

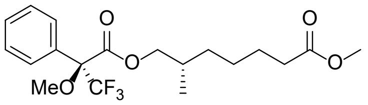

Alcohol **19** (2.6 mg, 15 µmol, 1 eq) was dissolved in anhydrous pyridine (0.15 mL) under N_2 atmosphere. (*R*)-(-)-MTPA-Cl (7 µL, 37 µmol, 2.5 eq., Mosher's chloride) was added and the reaction mixture was stirred for 1.5 h at rt. Aqueous $NaHCO_3$ (0.5 mL, sat.) and water (0.5 mL) were added and the mixture was extracted with Et_2O (3 × 1.5 mL). The combined organic layers were dried over $MgSO_4$, filtered and concentrated *in vacuo*. The residue was co-evaporated with toluene and then with $CDCl_3$ to remove residual solvents and to obtain Mosher's ester (5.5 mg, 94%). The diastereomeric excess was determined by ^{1}H NMR and turned out to be 95%. **^{1}H NMR (600 MHz, $CDCl_3$)** δ 7.53 – 7.49 (m, 2H), 7.43 – 7.38 (m, 3H), 4.22 (dd, *J* = 10.7, 5.7 Hz, 0.975H), 4.17 (dd, *J* = 10.8, 6.4 Hz, 0.025H), 4.13 (dd, *J* = 10.7, 5.7 Hz, 0.025H), 4.07 (dd, *J* = 10.7, 6.5 Hz, 0.975H), 3.66 (s, 3H), 3.58 – 3.52 (m, 3H), 2.28 (t, *J* = 7.5 Hz, 2H), 1.85 (dh, *J* = 13.2, 6.7 Hz, 1H), 1.63 – 1.55 (m, 2H), 1.39 – 1.13 (m, 4H), 0.91 (d, *J* = 6.8 Hz, 3H). **^{13}C NMR (151 MHz, $CDCl_3$)** δ 174.2, 166.8, 132.5, 129.7, 128.5, 127.5, 123.49 (q, *J* = 288.4 Hz), 84.78 (d, *J* = 27.8 Hz), 71.2, 55.6, 51.6, 34.0, 32.8, 32.4, 29.8, 26.3, 25.1, 16.8.

Methyl (2R/S,6*S*)-2-bromo-7-hydroxy-6-methylheptanoate (20)

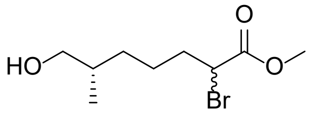

A fresh solution of LDA was prepared by adding *n*-BuLi (14.2 mL, 1.6 M in hexane, 22.6 mmol, 2.5 eq.) dropwise to a solution of diisopropylamine (3.2 mL, 22.6 mmol, 2.5 eq.) in anhydrous THF (23 mL) at -40 °C under N_2 atmosphere. The mixture was stirred for 30 min at -40 °C and then cooled to -78 °C. Meanwhile a solution of methyl ester **19** (1.58 g, 9.06 mmol, 1 eq.) and TMS chloride (3.6 mL, 28.1 mmol, 3.1 eq.) in anhydrous THF (23 mL) was prepared and added dropwise to the LDA solution over 15 min. Stirring was continued for 1 h at -78 °C whereupon cooling was removed and NBS (2.26 g, 12.7 mmol, 1.4 eq.) was added under a stream of N_2. The mixture was stirred for 1 h at 0 °C followed by addition of aqueous $NaHCO_3$ (100 mL, sat.) and Na_2SO_3 (1.6 g). The mixture was extracted with Et_2O (3 × 100 mL) and the combined layers were washed with brine (100 mL), dried with Na_2SO_4, filtered and concentrated *in vacuo*. The crude was still partially TMS protected, hence it was dissolved in MeOH (50 mL) followed by addition of TFA (0.20 mL, 2.6 mmol, 29 mol%). The mixture was stirred for 30 min at rt, concentrated *in vacuo* and co-evaporated with toluene. The resulting crude was purified by column chromatography (100 mL silica, eluent: 1 L 20% EtOAc/pentane) to obtain bromo ester **20** (2.19 g, 96%) as a yellow oil and as a diastereomeric mixture. **^{1}H NMR (400 MHz, $CDCl_3$)** δ 4.26 – 4.16 (m, 1H), 3.78 (s, 3H), 3.53 – 3.44 (m, 1H), 3.43 (dd, *J* = 10.5, 6.3 Hz, 1H), 2.13 – 1.92 (m, 2H), 1.68 – 1.29 (m, 4H), 1.20 – 1.08 (m, 1H), 0.91 (d, *J* = 6.7 Hz, 3H). **^{13}C NMR (101 MHz, $CDCl_3$)** δ 170.5, 170.5, 68.2, 68.1, 53.1, 53.1, 45.8, 45.7, 35.6, 35.6, 35.2, 35.1, 32.4, 32.3, 24.8, 24.8, 16.6, 16.5. Due to difficult ionisation, an HRMS could not be obtained. **[α]*D*** -9.8° (*c* 1.0, $CHCl_3$)

(*S*,*E*)-8-hydroxy-3-(methoxycarbonyl)-7-methyl-2-octenyl 2,3,4,6-tetra-*O*-*tert*-butyldimethylsilyl-β-D-glucopyranoside (23)

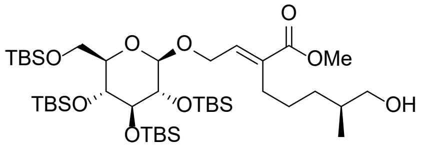

A mixture of bromo ester **20** (4.12 g, 16.3 mmol, 1 eq), PPh_3 (4.48 g, 17.1 mmol, 1.05 eq) and water (33 mL, 0.5 M) was stirred and heated to 90 °C for 2 d under N_2 atmosphere. Thereafter the mixture was cooled to 0 °C and NaOH (1.95 g, 48.8 mmol, 3 eq) and DCM (10 mL) were added. The resulting suspension was stirred thoroughly for 30 min after which more water (10 mL) was added. The emulsion was extracted with DCM (3 × 10 mL) and the combined organic layers were dried with Na_2SO_4, filtered and concentrated *in vacuo*. The crude Wittig reagent was used in the next step without further purification. The Wittig reagent (1 eq) and aldehyde **11** (11.6 g, 17.1 mmol, 1.05 eq.) were dissolved in anhydrous DCM under N_2 atmosphere and then stirred at 35 °C for 18 h. The mixture was concentrated *in vacuo* and purified by column chromatography (750 mL silica, eluents: 1 L 10% Et_2O/pentane, then 3 L 20% Et_2O/pentane, then 1 L 25% Et_2O/pentane and finally 3 L 30% Et_2O/pentane) to obtain α,β-unsaturated ester 23 (8.10 g, 60% yield over 2 steps) as a colourless oil. **^{1}H NMR (400 MHz, $CDCl_3$)** δ 6.84 (t, *J* = 6.0 Hz, 1H), 4.73 (d, *J* = 6.6 Hz, 1H), 4.53 (dd, *J* = 14.1, 5.6 Hz, 1H), 4.22 (dd, *J* = 14.1, 6.5 Hz, 1H), 3.92 (d, *J* = 2.2 Hz, 1H), 3.84 – 3.71 (m, 7H), 3.59 (d, *J* = 6.5 Hz, 1H), 3.52 – 3.38 (m, 2H), 2.35 – 2.19 (m, 2H), 1.69 – 1.56 (m, 1H), 1.50 – 1.37 (m, 3H), 1.17 – 1.07 (m, 1H), 0.93 – 0.83 (m, 39H), 0.12 – 0.02 (m, 24H). **^{13}C NMR (101 MHz, $CDCl_3$)** δ 167.9, 138.5, 133.5, 101.4, 82.7, 79.2, 77.8, 70.2, 68.4, 65.4, 64.2, 51.9, 35.6, 33.0, 27.6, 26.6, 26.1, 26.0, 25.9, 25.9, 18.5, 18.2, 18.1, 18.0, 16.6, -3.9, -4.2, -4.4, -4.6, -4.6, -4.7, -5.1. **HRMS** (APCI) calculated for $C_{41}H_{86}O_9Si_4Na$ ($[M+Na]^+$): 857.5241, found: 857.5310. **[α]*D*** -11° (*c* 1.0, $CHCl_3$).

(*R*,*E*)-8-(*tert*-butyldimethylsilyl)oxy-3-(methoxycarbonyl)-7-methyl-2-octenyl 2,3,4,6-tetra-*O*-*tert*-butyldimethylsilyl-β-D-glucopyranoside (24)

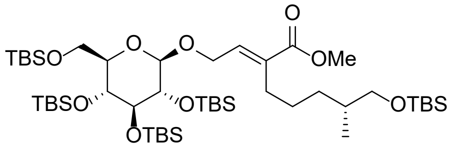

TBDMS triflate (2.0 mL, 8.7 mmol, 1.3 eq.) was added to a solution of alcohol **18** (5.59 g, 6.69 mmol, 1 eq) and 2,6-lutidine (1.5 mL, 13 mmol, 2 eq) in anhydrous DCM (45 mL, 0.15 M) at 0 °C under N_2 atmosphere. The mixture was stirred for 1.5 h at rt whereupon the reaction was quenched with water (100 mL). The mixture was extracted with DCM (2 × 50 mL) and the combined layers were dried with $MgSO_4$, filtered and concentrated *in vacuo*. The crude was purified by column chromatography (110 mL silica, eluent: 400 mL 5% Et_2O/pentane) to obtain **24** (6.26 g, 99%) as a colorless oil. **^{1}H NMR (400 MHz, $CDCl_3$)** δ 6.83 (t, *J* = 6.0 Hz, 1H), 4.73 (d, *J* = 6.6 Hz, 1H), 4.51 (dd, *J* = 14.1, 5.5 Hz, 1H), 4.22 (dd, *J* = 14.1, 6.6 Hz, 1H), 3.93 (d, *J* = 2.9 Hz, 1H), 3.83 – 3.72 (m, 7H), 3.59 (d, *J* = 6.6 Hz, 1H), 3.42 (dd, *J* = 9.7, 5.8 Hz, 1H), 3.35 (dd, *J* = 9.7, 6.4 Hz, 1H), 2.28 – 2.22 (m, 2H), 1.63 – 1.52 (m, 1H), 1.48 – 1.29 (m, 3H), 1.11 – 1.00 (m, 1H), 0.92 – 0.83 (m, 48H), 0.12 – 0.02 (m, 30H). **^{13}C NMR (101 MHz, $CDCl_3$)** δ 167.9, 138.4, 133.6, 101.4, 82.7, 79.2, 77.8, 70.2, 68.4, 65.5, 64.2, 51.9, 35.7, 33.1, 27.8, 26.7, 26.1, 26.1, 26.1, 26.0,

25.9, 18.5, 18.5, 18.2, 18.1, 18.0, 16.8, -3.9, -4.2, -4.4, -4.6, -4.6, -4.7, -5.1, -5.2, -5.2. **HRMS** (ESI) calculated for $C_{47}H_{100}O_9Si_5Na$ ($[M+Na]^+$): 971.6106, found: 971.6136. **[α]*D*** -3.5° (*c* 1.0, $CHCl_3$).

(*S,E*)-8-(*tert*-butyldimethylsilyl)oxy-3-(methoxycarbonyl)-7-methyl-2-octenyl 2,3,4,6-tetra-*O*-*tert*-butyldimethylsilyl-β-D-glucopyranoside (28)

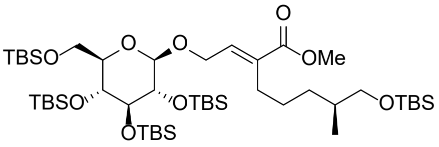

TBDMS triflate (2.9 mL, 12.6 mmol, 1.3 eq.) was added to a solution of alcohol **23** (8.10 g, 9.70 mmol, 1 eq.) and 2,6-lutidine (2.2 mL, 19.4 mmol, 2 eq.) in anhydrous DCM (65 mL, 0.15 M) at 0 °C under N_2 atmosphere. The mixture was stirred at rt for 1 h whereupon the reaction was quenched with water (100 mL). The mixture was extracted with DCM (2 × 50 mL) and the combined layers were dried with $MgSO_4$, filtered and concentrated *in vacuo*. The crude was purified by column chromatography (110 mL silica, eluent: 500 mL 5% Et_2O/pentane) to obtain **28** (8.96 g, 97%) as a colourless oil. **^{1}H NMR (400 MHz, $CDCl_3$)** δ 6.83 (t, *J* = 6.0 Hz, 1H), 4.73 (d, *J* = 6.7 Hz, 1H), 4.52 (dd, *J* = 14.1, 5.5 Hz, 1H), 4.21 (dd, *J* = 14.1, 6.6 Hz, 1H), 3.93 (d, *J* = 2.9 Hz, 1H), 3.84 – 3.72 (m, 7H), 3.59 (d, *J* = 6.6 Hz, 1H), 3.42 (dd, *J* = 9.7, 5.9 Hz, 1H), 3.35 (dd, *J* = 9.7, 6.5 Hz, 1H), 2.33 – 2.16 (m, 2H), 1.63 – 1.56 (m, 1H), 1.48 – 1.30 (m, 3H), 1.13 – 1.00 (m, 1H), 0.93 – 0.83 (m, 48H), 0.12 – 0.01 (m, 30H). **^{13}C NMR (101 MHz, $CDCl_3$)** δ 167.9, 138.4, 133.6, 101.4, 82.7, 79.2, 77.8, 70.2, 68.4, 65.5, 64.2, 51.9, 35.7, 33.1, 27.8, 26.7, 26.1, 26.1, 26.1, 26.0, 25.9, 18.5, 18.5, 18.2, 18.1, 18.0, 16.8, -3.9, -4.2, -4.4, -4.6, -4.6, -4.7, -5.1, -5.2, -5.2. **HRMS** (ESI) calculated for $C_{47}H_{104}O_9Si_5N$ ($[M+NH_4]^+$): 966.6552, found: 966.6544. **[α]*D*** -9.5° (*c* 1.0, $CHCl_3$).

(*R,E*)-8-(*tert*-butyldimethylsilyl)oxy-3-(hydroxymethyl)-7-methyl-2-octenyl 2,3,4,6-tetra-*O*-*tert*-butyldimethylsilyl-β-D-glucopyranoside (25)

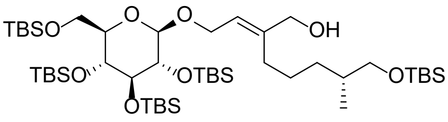

DIBAL-H (13.5 mL, 1 M in DCM, 13.5 mmol, 2.1 eq.) was added dropwise over 5 min to a solution of α,β-unsaturated ester **24** (6.25 g, 6.58 mmol, 1 eq.) in anhydrous DCM (44 mL, 0.15 M) at -78 °C under N_2 atmosphere. After 1 h stirring at -78 °C the reaction was quenched by addition of MeOH (6 mL), whereupon the reaction was allowed to warm to rt. Aqueous NH_4Cl (10 mL, sat.) and Rochelle salt (30 mL, sat.) were added and the mixture was stirred thoroughly at rt for 1 h. The emulsion was extracted with Et_2O (3 × 50 mL) and the combined organic layers were washed with brine (50 mL), dried with $MgSO_4$, filtered and concentrated *in vacuo*. The crude was purified by column chromatography (120 mL silica, eluent: 500 mL 20% Et_2O/pentane) to obtain **25** (5.88 g, 97%) as a colourless oil. **^{1}H NMR (400 MHz, $CDCl_3$)** δ 5.63 (t, *J* = 6.4 Hz, 1H), 4.72 (d, *J* = 6.7 Hz, 1H), 4.39 (dd, *J* = 12.2, 6.2 Hz, 1H), 4.12 (dd, *J* = 12.2, 6.9 Hz, 1H), 4.05 (d, *J* = 6.1 Hz, 2H), 3.90 (d, *J* = 2.9 Hz, 1H), 3.83 – 3.71 (m, 4H), 3.57 (d, *J* = 6.7 Hz, 1H), 3.42 (dd, *J* = 9.7, 5.9 Hz, 1H), 3.36 (dd, *J* = 9.7, 6.4 Hz, 1H), 2.16 – 2.01 (m, 2H), 1.63 – 1.53 (m, 1H), 1.48 – 1.33 (m, 3H), 1.31 (t, *J* = 6.4 Hz, 1H, OH), 1.11 – 1.01 (m, 1H), 0.92 – 0.83 (m, 48H), 0.10 – 0.02 (m, 30H). **HRMS** (ESI) calculated for $C_{46}H_{100}O_8Si_5Na$ ($[M+Na]^+$): 943.6157, found: 943.6187. **[α]*D*** -3.4° (*c* 1.0, $CHCl_3$).

(*S,E*)-8-(*tert*-butyldimethylsilyl)oxy-3-(hydroxymethyl)-7-methyl-2-octenyl 2,3,4,6-tetra-*O*-*tert*-butyldimethylsilyl-β-D-glucopyranoside (29)

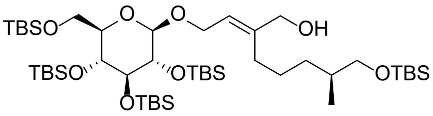

DIBAL-H (20.7 mL, 1 M in DCM, 20.7 mmol, 2.2 eq.) was added dropwise over 5 min to a solution of α,β-unsaturated ester **28** (8.94 g, 9.41 mmol, 1 eq.) in anhydrous DCM (63 mL, 0.15 M) at -78 °C under N_2 atmosphere. After 1 h stirring at -78 °C, the reaction was quenched by addition of MeOH (10 mL), whereupon the reaction was allowed to warm to rt. Aqueous NH_4Cl (15 mL, sat.) and Rochelle salt (45 mL, sat.) were added and the mixture was stirred thoroughly at rt for 1 h. The emulsion was extracted with Et_2O (3 × 60 mL) and the combined organic layers were washed with brine (50 mL), dried with $MgSO_4$, filtered and concentrated *in vacuo*. The crude was purified by column chromatography (150 mL silica, eluent: 500 mL 20% Et_2O/pentane) to obtain **29** (8.44 g, 97%) as a colourless oil. **^{1}H NMR (400 MHz, $CDCl_3$)** δ 5.63 (t, *J* = 6.5 Hz, 1H), 4.72 (d, *J* = 6.8 Hz, 1H), 4.40 (dd, *J* = 12.2, 6.2 Hz, 1H), 4.12 (dd, *J* = 12.2, 6.9 Hz, 1H), 4.05 (s, 2H), 3.90 (d, *J* = 2.8 Hz, 1H), 3.84 – 3.71 (m, 4H), 3.57 (d, *J* = 6.7 Hz, 1H), 3.42 (dd, *J* = 9.7, 5.9 Hz, 1H), 3.36 (dd, *J* = 9.8, 6.4 Hz, 1H), 2.12 – 2.02 (m, 2H), 1.66 – 1.54 (m, 1H), 1.46 – 1.31 (m, 3H), 1.25 (s, 1H, OH), 1.11 – 1.00 (m, 1H), 0.91 – 0.83 (m, 48H), 0.11 – 0.00 (m, 30H). **^{13}C NMR (101 MHz, $CDCl_3$)** δ 142.5, 122.7, 101.0, 82.6, 79.3, 78.0, 70.3, 68.4, 66.8, 65.1, 64.3, 35.8, 33.3, 28.8, 26.3, 26.1, 26.1, 26.1, 26.0, 25.9, 18.5, 18.5, 18.2, 18.2, 18.0, 16.8, -3.8, -4.2, -4.4, -4.6, -4.6, -4.8, -5.1, -5.1, -5.2. **HRMS** (ESI) calculated for $C_{46}H_{104}O_8Si_5N$ ($[M+NH_4]^+$): 938.6603, found: 938.6594. **[α]*D*** -8.4° (*c* 1.0, $CHCl_3$).

(*R,E*)-8-(*tert*-butyldimethylsilyl)oxy-3-(chloromethyl)-7-methyl-2-octenyl 2,3,4,6-tetra-*O*-*tert*-butyldimethylsilyl-β-D-glucopyranoside (26)

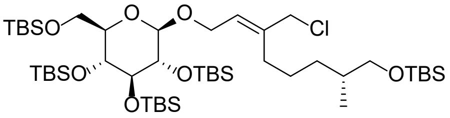

To a solution of allyl alcohol **25** (5.87 g, 6.37 mmol, 1 eq.) and PPh_3 (2.51 g, 9.55 mmol, 1.5 eq.) in anhydrous DCM (32 mL, 0.2 M) was added NCS (1.28 g, 9.55 mmol, 1.5 eq.) at 0 °C under N_2 atmosphere. The reaction mixture was stirred at 0 °C for 30 min and then allowed to warm to rt. The mixture was concentrated *in vacuo* and the crude was suspended in pentane (400 mL) by sonicating for 5 min. The suspension was filtered and concentrated *in vacuo*. The clear oil with white solids was resuspended in pentane (~25 mL) again and then filtered over silica (eluent 1% Et_2O/pentane) to obtain allylic chloride **26** (5.66 g, 96%) as a colourless oil. **^{1}H NMR (400 MHz, $CDCl_3$)** δ 5.72 (t, *J* = 6.2 Hz, 1H), 4.71 (d, *J* = 6.7 Hz, 1H), 4.39 (dd, *J* = 12.6, 5.8 Hz, 1H), 4.10 (dd, *J* = 12.7, 6.8 Hz, 1H), 4.04 (s, 2H), 3.92 (d, *J* = 2.8 Hz, 1H), 3.82 – 3.72 (m, 4H), 3.57 (d, *J* = 6.7 Hz, 1H), 3.43 (dd, *J* = 9.7, 5.9 Hz, 1H), 3.37 (dd, *J* = 9.7, 6.3 Hz, 1H), 2.24 – 2.09 (m, 2H), 1.64 – 1.55 (m, 1H), 1.51 – 1.32 (m, 3H), 1.12 – 1.01 (m, 1H), 0.92 – 0.85 (m, 48H), 0.11 – 0.02 (m, 30H). **^{13}C NMR (101 MHz, $CDCl_3$)** δ 138.4, 127.6, 101.2, 82.6, 79.3, 77.9, 70.2, 68.3, 65.2, 64.2, 49.5, 35.7, 33.2, 28.8, 26.1, 26.1, 26.0, 25.9, 25.7, 18.5, 18.5, 18.2, 18.2, 18.0, 16.8, -3.8, -4.2, -4.4, -4.6, -4.6, -4.8, -5.1, -5.2. **HRMS** (ESI) calculated for $C_{46}H_{99}ClO_7Si_5Na$ ($[M+Na]^+$): 961.5818, found: 961.5846. **[α]*D*** -3.4° (*c* 1.0, $CHCl_3$).

(*S,E*)-8-(*tert*-butyldimethylsilyl)oxy-3-(chloromethyl)-7-methyl-2-octenyl 2,3,4,6-tetra-*O*-*tert*-butyldimethylsilyl-β-D-glucopyranoside (30)

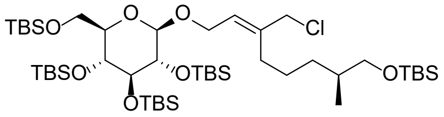

To a solution of allyl alcohol **29** (5.87 g, 6.37 mmol, 1 eq.) and PPh_3 (2.51 g, 9.55 mmol, 1.5 eq.) in anhydrous DCM (32 mL, 0.2 M) was added NCS (1.28 g, 9.55 mmol, 1.5 eq.) at 0 °C under N_2 atmosphere. The reaction mixture was stirred at 0 °C for 30 min and then allowed to warm to rt. The mixture was concentrated *in vacuo* and the crude was suspended in pentane (400 mL) by sonicating for 5 min. $MgSO_4$ was added and the mixture was filtered and concentrated *in vacuo*. The clear oil with white solids was resuspended in pentane (100 mL), filtered and concentrated *in vacuo*. The resulting residue was redissolved in a minimum amount of pentane (~10 mL), filtered over a plug of cotton and concentrated *in vacuo* to obtain allyl chloride **30** (8.29 g, 97%) as a colourless oil. **^{1}H NMR (400 MHz, $CDCl_3$)** δ 5.71 (t, *J* = 6.2 Hz, 1H), 4.71 (d, *J* = 6.7 Hz, 1H), 4.39 (dd, *J* = 12.7, 5.9 Hz, 1H), 4.10 (dd, *J* = 12.7, 6.7 Hz, 1H), 4.04 (s, 2H), 3.92 (d, *J* = 2.7 Hz, 1H), 3.84 – 3.71 (m, 4H), 3.57 (d, *J* = 6.7 Hz, 1H), 3.42 (dd, *J* = 9.7, 6.0 Hz, 1H), 3.37 (dd, *J* = 9.7, 6.4 Hz, 1H), 2.22 – 2.10 (m, 2H), 1.64 – 1.53 (m, 1H), 1.49 – 1.34 (m, 3H), 1.11 – 1.01 (m, 1H), 0.91 – 0.85 (m, 48H), 0.10 – 0.03 (m, 30H). **^{13}C NMR (101 MHz, $CDCl_3$)** δ 138.4, 127.6, 101.2, 82.6, 79.2, 77.9, 70.1, 68.3, 65.2, 64.2, 49.5, 35.7, 33.2, 28.8, 26.1, 26.1, 26.0, 25.9, 25.7, 18.5, 18.5, 18.2, 18.1, 18.0, 16.8, -3.9, -4.2, -4.4, -4.6, -4.6, -4.8, -5.1, -5.2. **HRMS** (ESI) calculated for $C_{46}H_{103}ClO_7Si_5N$ ($[M+NH_4]^+$): 956.6264, found: 956.6248. **[α]*D*** -8.2° (*c* 1.0, $CHCl_3$).

(*R,E*)-3-(chloromethyl)-8-hydroxy-7-methyl-2-octenyl 3-deoxy-3-oxo-β-D-glucopyranoside (27)

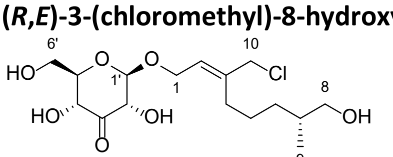

To a mixture of **26** (4.67 g, 4.96 mmol, 1 eq.) in MeOH/dioxane (3/1 v/v, 50 mL) was added dropwise trifluoromethanesulfonic acid (0.22 mL, 2.48 mmol, 0.5 eq.) at rt. The mixture was heated to 40 °C, stirred for 2 h, and then quenched by adding NaOAc (224 mg, 2.73 mmol, 0.55 eq). Next, 2-*tert*-butyl-1,4-benzoquinone (978 mg, 5.95 mmol, 1.2 eq) was added at rt followed by addition of (neocuproine)$Pd(CH_3CN)_2(OTf)_2$ (312 mg, 0.45 mmol, 9 mol%). After stirring at rt for 30 min, activated carbon (600 mg) was added. The black mixture was filtered over Celite and washed with MeOH. The yellow filtrate was concentrated *in vacuo*, dissolved in EtOAc (30 mL) and transferred to a separatory funnel. Aqueous $NaHCO_3$ (10 mL, sat.) and brine (30 mL) were added followed by washing. The aqueous layer was back-extracted with EtOAc (4 × 25 mL) and all organic layers were combined. These were dried with Na_2SO_4, filtered and concentrated *in vacuo*. The crude was loaded onto Celite (9 g) and then purified by column chromatography (45 g spherical silica, eluents: 500 mL 3% MeOH/$CHCl_3$, 500 mL 4% MeOH/$CHCl_3$, 500 mL 5% MeOH/$CHCl_3$ and finally 500 mL 6% MeOH/$CHCl_3$) to obtain keto-sugar **27** (865 mg, 48%) as a syrup. **^{1}H NMR (400 MHz, CD_3OD)** δ 5.79 (t, *J* = 6.6 Hz, 1H, H2), 4.49 (dd, *J* = 12.5, 6.0 Hz, 1H, H1a), 4.39 (d, *J* = 7.9 Hz, 1H, H1'), 4.35 (dd, *J* = 12.6, 7.2 Hz, 1H, H1b), 4.23 (dd, *J* = 10.1, 1.7 Hz, 1H, H4'), 4.16 – 4.10 (m, 3H, H2', H10), 3.95 (dd, *J* = 12.1, 2.1 Hz, 1H, H6'a), 3.80 (dd, *J* = 12.1, 5.0 Hz, 1H, H6'b), 3.41 (dd, *J* = 10.6, 6.0 Hz, 1H, H8a), 3.37 – 3.28 (m, 2H, H8b, H5'), 2.33 – 2.18 (m, 2H, H4), 1.66 – 1.38 (m, 4H), 1.17 – 1.07 (m, 1H), 0.91 (d, *J* = 6.7 Hz, 3H, H9). **^{13}C NMR (101 MHz, CD_3OD)** δ 207.0 (C3'), 141.6 (C3), 127.4 (C2), 104.8 (C1'), 78.4 (C5'), 78.2 (C2'), 73.6 (C4'), 68.3 (C8), 66.4 (C1), 62.6 (C6'), 49.7 (C10), 36.7 (C7), 34.2 (C5/C6), 29.4 (C4), 26.7 (C5/C6), 17.1 (C9). **HRMS** (ESI) calculated for $C_{16}H_{27}ClO_7Na$ ($[M+Na]^+$): 389.1338, found: 389.1342. **[α]*D*** -11° (*c* 1.0, MeOH).

(*S*,*E*)-3-(chloromethyl)-8-hydroxy-7-methyl-2-octenyl 3-deoxy-3-oxo-β-D-glucopyranoside (31)

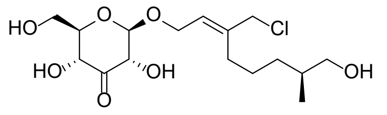

To a mixture of **30** (8.28 g, 8.81 mmol, 1 eq.) in MeOH/dioxane (3/1, 90 mL) was added dropwise trifluoromethanesulfonic acid (0.39 mL, 4.40 mmol, 0.5 eq) at rt. The mixture was heated to 40 °C, stirred for 3 h, and then quenched by adding NaOAc (397 mg, 4.84 mmol, 0.55 eq.). Next, 2-*tert*-butyl-1,4-benzoquinone (1.52 g, 9.25 mmol, 1.05 eq) was added at rt followed by addition of (neocuproine)Pd(CH_3CN)$_2$(OTf)$_2$ (555 mg, 0.80 mmol, 9 mol%). After stirring at rt for 1 h, activated carbon (1 g) was added. The black mixture was filtered over Celite and washed with MeOH. The orange filtrate was concentrated *in vacuo*, dissolved in EtOAc (50 mL) and transferred to a separatory funnel. Aqueous $NaHCO_3$ (15 mL, sat.) and brine (45 mL) were added followed by washing. The aqueous layer was back-extracted with EtOAc (4 × 50 mL) and all organic layers were combined. These were dried with Na_2SO_4, filtered and concentrated *in vacuo*. The crude was dissolved in minimum amount of $CHCl_3$ and then purified by column chromatography (100 mL spherical silica, eluents: 100 mL CHCL$_3$, 1 L 3% MeOH/$CHCl_3$, 500 mL 4% MeOH/$CHCl_3$ and then 500 mL 5% MeOH/$CHCl_3$) to obtain keto-sugar **31** (1.83 g, 57%) as a syrup. **^{1}H NMR (400 MHz, CD_3OD)** δ 5.79 (t, *J* = 6.6 Hz, 1H, H2), 4.49 (dd, *J* = 12.6, 6.1 Hz, 1H, H1a), 4.39 (d, *J* = 7.9 Hz, 1H, H1'), 4.34 (dd, *J* = 12.6, 7.2 Hz, 1H, H1b), 4.23 (dd, *J* = 10.1, 1.6 Hz, 1H, H4'), 4.15 – 4.11 (m, 3H, H2', H10), 3.95 (dd, *J* = 12.1, 2.1 Hz, 1H, H6'a), 3.80 (dd, *J* = 12.1, 5.0 Hz, 1H, H6'b), 3.41 (dd, *J* = 10.6, 6.0 Hz, 1H, H8a), 3.37 – 3.32 (m, 1H, H8b), 3.32 – 3.28 (m, 1H, H5', overlaps with residual CD_3OD), 2.25 (t, *J* = 7.6 Hz, 2H, H4), 1.64 – 1.39 (m, 4H), 1.17 – 1.06 (m, 1H), 0.91 (d, *J* = 6.7 Hz, 3H, H9). **^{13}C NMR (101 MHz, CD_3OD)** δ 207.0 (C3'), 141.6 (C3), 127.4 (C2), 104.8 (C1'), 78.4 (C5'), 78.2 (C2'), 73.6 (C4'), 68.3 (C8), 66.4 (C1), 62.6 (C6'), 49.7 (C10), 36.7 (C7), 34.2 (C5/C6), 29.4 (C4), 26.7 (C5/C6), 17.1 (C9). **HRMS** (ESI) calculated for $C_{16}H_{26}ClO_7$ ([M-H]$^-$): 365.1362, found: 365.1376. **[α]*$_D$*** -24° (*c* 0.5, MeOH).

7-*epi*-dissectol A (32A) & 2,7-bis-*epi*-dissectol A (32B)

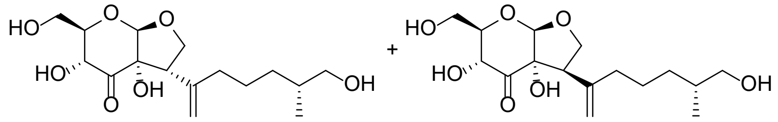

Keto-sugar **27** (807 mg, 2.20 mmol, 1 eq) and Pd(PPh_3)$_4$ (762 mg, 0.66 mmol, 30 mol%) were dissolved in anhydrous THF (22 mL, 0.1 M) under N_2 atmosphere, followed by addition of DBU (0.49 ml, 3.30 mmol, 1.5 eq). The reaction mixture was stirred at rt for 1 h during which a yellow suspension formed. Water (10 mL), brine (20 mL) and EtOAc (20 mL) were added. The organic layer was collected and the aqueous layer was extracted with EtOAc (7× 20 mL). The combined organic layers were dried with Na_2SO_4, filtered and concentrated *in vacuo*. The residue was partitioned between toluene (40 mL) and water/MeOH (1/1 v/v, 40 mL). The aqueous layer was collected and the toluene layer was back-extracted once with water/MeOH (1/1 v/v, 15 mL). The combined aqueous layers were washed with toluene (40 mL) and this organic layer was back-extracted again with water/MeOH (1/1 v/v, 1 × 15 mL). The combined aqueous layers were concentrated *in vacuo* resulting a yellow syrup (600 mg, ratio **32A**/**32B**: 65/35 as determined by ^{1}H NMR). The crude was dissolved in a minimum amount of DCM and then purified by column chromatography (60 g spherical silica, eluents: 1.5 L 8% *i*PrOH/DCM and 200 mL 15% *i*PrOH/DCM) to obtain 7-*epi*-dissectol A (**32A**, 194 mg, 27% yield, does not contain **32B**), 2,7-bis-*epi*-dissectol A (**32B**, 74 mg, 10% yield, contains 5% of **32A**) and a mixed fraction (87 mg, 12% yield, ratio **32A**/**32B**: 35/65) as syrups.

Characterization of **32A**: **^{1}H NMR (600 MHz, CD_3OD)** δ 5.02 (s, 1H, H10a), 4.97 (s, 1H, H1'), 4.94 (s, 1H, H10b), 4.58 (d, *J* = 10.2 Hz, 1H, H4'), 4.33 (t, *J* = 8.1 Hz, 1H, H1a), 4.17 (dd, *J* = 10.1, 8.0 Hz, 1H, H1b), 3.91 (dd, *J* = 12.0, 1.6 Hz, 1H, H6'a), 3.83 (dd, *J* = 12.1, 4.4 Hz, 1H, H6'b), 3.52 (t, *J* = 9.4 Hz, 1H, H2), 3.42 – 3.37 (m, 2H, H5', H8a), 3.35 – 3.31 (m, 1H, H8b), 1.97 (ddd, *J* = 15.3, 9.3, 6.0 Hz, 1H, H4a), 1.88 (ddd, *J* = 14.8, 9.2, 5.1 Hz, 1H, H4b), 1.60 – 1.53 (m, 1H, H7), 1.53 – 1.45 (m, 1H, H5a), 1.44 – 1.32 (m, 2H, H5b, H6a), 1.11 – 1.01 (m, 1H, H6b), 0.90 (d, *J* = 6.7 Hz, 3H, H9). **^{13}C NMR (151 MHz, CD_3OD)** δ 208.9 (C3'), 142.9 (C3), 114.4 (C10), 110.4 (C1'), 87.2 (C2'), 78.3 (C5'), 72.5 (C1), 72.3 (C4'), 68.3 (C8), 62.2 (C6'), 51.5 (C2), 38.0 (C4), 36.7 (C7), 33.9 (C6), 26.4 (C5), 17.1 (C9). NMR characterisation (in CD_3OD) does **not** match spectra of natural isolate (spectra supplied by Chou and co-workers).[10] **^{1}H NMR (600 MHz, Pyridine-*d5*)** δ 5.57 (s, 1H, H1'), 5.41 (d, *J* = 10.1 Hz, 1H, H4'), 5.13 (s, 1H, H10a), 5.03 (s, 1H, H10b), 4.60 (dd, *J* = 10.3, 7.7 Hz, 1H, H1a), 4.53 (t, *J* = 8.0 Hz, 1H, H1b), 4.50 (dd, *J* = 12.1, 2.1 Hz, 1H, H6'a), 4.47 (dd, *J* = 12.1, 4.0 Hz, 1H, H6'b), 3.92 (ddd, *J* = 10.1, 3.7, 2.3 Hz, 1H, H5'), 3.88 (t, *J* = 9.3 Hz, 1H, H2), 3.64 (dd, *J* = 10.4, 5.8 Hz, 1H, H8a), 3.56 (dd, *J* = 10.4, 6.5 Hz, 1H, H8b), 2.07 (dt, *J* = 15.3, 7.6 Hz, 1H, H4a), 2.01 (ddd, *J* = 15.3, 9.5, 4.5 Hz, 1H, H4b), 1.73 – 1.62 (m, 1H, H7), 1.50 – 1.39 (m, 2H, H5a, H6a), 1.38 – 1.30 (m, 1H, H5a), 1.06 – 1.00 (m, 1H, H6a), 0.97 (d, *J* = 6.7 Hz, 3H, H9). **^{13}C NMR (151 MHz, Pyridine-*d5*)** δ 210.0 (C3'), 143.1 (C3), 113.7 (C10), 110.7 (C1'), 87.6 (C2'), 79.0 (C5'), 72.8 (C4'), 72.2 (C1), 67.7 (C8), 62.4 (C6'),

51.8 (C2), 37.5 (C4), 36.7 (C7), 33.7 (C6), 25.8 (C5), 17.6 (C9). NMR characterisation (in C_5D_5N) is consistent with literature.[11,12] **HRMS** (ESI) calculated for $C_{16}H_{26}O_7Na$ ([M+Na]$^+$): 353.1571, found: 353.1579. **[α]*D*** -30° (*c* 1.0, MeOH).

Characterization of **32B**: **^{1}H NMR (600 MHz, CD_3OD)** δ 5.20 (s, 1H, H10a), 5.12 (s, 1H, H10b), 4.99 (s, 1H, H1'), 4.41 – 4.35 (m, 2H, H1), 4.20 (d, *J* = 10.3 Hz, 1H, H4'), 3.88 (dd, *J* = 12.1, 2.1 Hz, 1H, H6'a), 3.72 (dd, *J* = 12.1, 4.9 Hz, 1H, H6'b), 3.42 – 3.36 (m, 2H, H5', H8a), 3.34 – 3.32 (m, 1H, H8b), 3.02 (t, *J* = 7.8 Hz, 1H, H2), 1.99 – 1.88 (m, 2H, H4), 1.61 – 1.53 (m, 1H, H7), 1.49 – 1.34 (m, 3H, H5, H6a), 1.11 – 1.04 (m, 1H, H6b), 0.89 (d, *J* = 6.7 Hz, 3H, H9). **^{13}C NMR (151 MHz, CD_3OD)** δ 209.0 (C3'), 147.0 (C10), 114.3 (C3), 110.3 (C1'), 90.4 (C2'), 77.3 (C5'), 75.1 (C4'), 73.4 (C1), 68.4 (C8), 62.6 (C6'), 57.4 (C2), 38.7 (C4), 36.7 (C7), 33.9 (C6), 26.1 (C5), 17.0 (C9). **HRMS** (ESI) calculated for $C_{16}H_{26}O_7Na$ ([M+Na]$^+$): 353.1571, found: 353.1577. **[α]*D*** -96° (*c* 1.0, MeOH).

dissectol A (33A) & 2-*epi*-dissectol A (33B)

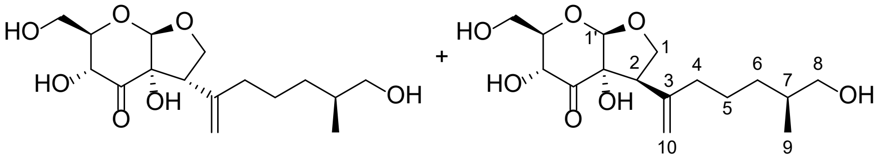

Keto-sugar **31** (1.80 g, 4.91 mmol, 1 eq.) and $Pd(PPh_3)_4$ (1.70 g, 1.47 mmol, 30 mol%) were dissolved in anhydrous THF (49 mL, 0.1 M) under N_2 atmosphere, followed by addition of DBU (1.1 ml, 7.36 mmol, 1.5 eq.). The reaction mixture was stirred at rt for 1 h during which a yellow suspension formed. Water (10 mL), brine (40 mL) and EtOAc (30 mL) were added. The organic layer was collected and the aqueous layer was extracted with EtOAc (7 × 30 mL). The combined organic layers were dried with Na_2SO_4, filtered and concentrated *in vacuo*. The residue was partitioned between toluene (80 mL) and water/MeOH (1/1 v/v, 80 mL). The aqueous layer was collected and the toluene layer was back-extracted with water/MeOH (1/1 v/v, 1 × 20 mL & 1 × 10 mL). The combined aqueous layers were washed with toluene (80 mL) and this organic layer was back-extracted again with water/MeOH (1/1 v/v, 1 × 10 mL). The combined aqueous layers were concentrated *in vacuo* resulting a yellow syrup (1.16 g, ratio **33A**/**33B**: 62/38 as determined by ^{1}H NMR). The crude was dissolved in a minimum amount of DCM and then purified by column chromatography (120 mL spherical silica, eluents: 2 L 8% *i*PrOH/DCM, 1 L 9% *i*PrOH/DCM and 1 L 12% *i*PrOH/DCM) to obtain dissectol A (**33A**, 461 mg, 28% yield, does not contain **33B**), 2-*epi*-dissectol A (**33B**, 271 mg, 17% yield, contains 2% of **33A**) and a mixed fraction (33 mg, 2% yield, ratio **33A**/**33B**: 40/60) as syrups.

Characterization of **33A**: **^{1}H NMR (600 MHz, CD_3OD)** δ 5.02 (s, 1H, H10a), 4.97 (s, 1H, H1'), 4.95 (s, 1H, H10b), 4.58 (d, *J* = 10.2 Hz, 1H, H4'), 4.32 (t, *J* = 8.1 Hz, 1H, H1a), 4.17 (dd, *J* = 10.3, 7.8 Hz, 1H, H1b), 3.91 (dd, *J* = 12.1, 2.1 Hz, 1H, H6'a), 3.83 (dd, *J* = 12.1, 4.4 Hz, 1H, H6'b), 3.51 (t, *J* = 9.3 Hz, 1H, H2), 3.42 – 3.38 (m, 2H, H5', H8a), 3.35 – 3.32 (m, 1H, H8b), 1.98 – 1.87 (m, 2H, H4), 1.60 – 1.52 (m, 1H, H7), 1.49 – 1.34 (m, 3H, H5, H6a), 1.11 – 1.04 (m, 1H, H6b), 0.90 (d, *J* = 6.7 Hz, 3H, H9). **^{13}C NMR (151 MHz, CD_3OD)** δ 208.9 (C3'), 142.8 (C3), 114.4 (C10), 110.4 (C1'), 87.2 (C2'), 78.3 (C5'), 72.5 (C1), 72.3 (C4'), 68.3 (C8), 62.2 (C6'), 51.4 (C2), 38.0 (C4), 36.7 (C7), 33.9 (C6), 26.4 (C5), 17.1 (C9). NMR characterisation (in CD_3OD) matches the spectra of natural isolate (spectra supplied by Chou and co-workers).[10] **^{1}H NMR (600 MHz, Pyridine-*d5*)** δ 5.58 (s, 1H, H1'), 5.42 (d, *J* = 10.1 Hz, 1H, H4'), 5.14 (s, 1H, H10a), 5.03 (s, 1H, H10b), 4.61 (dd, *J* = 10.2, 7.7 Hz, 1H, H1a), 4.54 (t, *J* = 8.0 Hz, 1H, H1b), 4.50 (dd, *J* = 12.1, 2.0 Hz, 1H, H6'a), 4.47 (dd, *J* = 12.1, 4.0 Hz, 1H, H6'b), 3.93 (ddd, *J* = 10.1, 3.6, 2.4 Hz, 1H, H5'), 3.87 (t, *J* = 9.3 Hz, 1H, H2), 3.65 (dd, *J* = 10.4, 5.7 Hz, 1H, H8a), 3.57 (dd, *J* = 10.4, 6.4 Hz, 1H, H8b), 2.09 – 1.98 (m, *J* = 6.7 Hz, 2H, H4), 1.73 – 1.62 (m, 1H, H7), 1.46 – 1.33 (m, 3H, H5, H6a), 1.06 – 0.99 (m, 1H, H6b), 0.97 (d, *J* = 6.7 Hz, 3H, H9). **^{13}C NMR (151 MHz, Pyridine-*d5*)** δ 210.0 (C3'), 143.1 (C3), 113.8 (C10), 110.7 (C1'), 87.6 (C2'), 79.1 (C5'), 72.8 (C4'), 72.2 (C1), 67.7 (C8), 62.4 (C6'), 51.8 (C2), 37.5 (C4), 36.8 (C7), 33.7 (C6), 25.8 (C5), 17.6 (C9). NMR characterisation (in C_5D_5N) matches literature.[11,12] **HRMS** (ESI) calculated for $C_{16}H_{26}O_7Na$ ([M+Na]$^+$): 353.1571, found: 353.1564. **[α]*D*** -42° (*c* 1.0, MeOH), lit.[11] $[\alpha]^{26}_D$ +125° (*c* 2.0, MeOH).

Characterization of **33B**: **^{1}H NMR (600 MHz, CD_3OD)** δ 5.20 (s, 1H, H10a), 5.12 (s, 1H, H10b), 4.99 (s, 1H, H1'), 4.40 – 4.35 (m, 2H, H1), 4.20 (d, *J* = 10.3 Hz, 1H, H4'), 3.88 (dd, *J* = 12.1, 2.1 Hz, 1H, H6'a), 3.72 (dd, *J* = 12.2, 4.9 Hz, 1H, H6'b), 3.42 – 3.37 (m, 2H, H5', H8a), 3.35 – 3.32 (m, 1H, H8b), 3.02 (t, *J* = 7.8 Hz, 1H, H2), 1.97 (ddd, *J* = 15.6, 9.6, 6.3 Hz, 1H, H4a), 1.90 (ddd, *J* = 14.9, 9.4, 5.1 Hz, 1H, H4b), 1.60 – 1.50 (m, 2H, H5a, H7), 1.44 – 1.29 (m, 2H, H6a, H5b), 1.05 (dddd, *J* = 12.8, 10.1, 7.9, 5.0 Hz, 1H, H6b), 0.90 (d, *J* = 6.7 Hz, 3H, H9). **^{13}C NMR (151 MHz, CD_3OD)** δ 209.0 (C3'), 147.0 (C10), 114.3 (C3), 110.3 (C1'), 90.4 (C2'), 77.3 (C5'), 75.1 (C4'), 73.4 (C1), 68.3 (C8), 62.6 (C6'), 57.4 (C2), 38.7 (C4), 36.8 (C7), 34.0 (C6), 26.3 (C5), 17.1 (C9). **HRMS** (ESI) calculated for $C_{16}H_{26}O_7Na$ ([M+Na]$^+$): 353.1571, found: 353.1568. **[α]*D*** -108° (*c* 1.0, MeOH).

Dissectol uptake by *Mycobacterium tuberculosis* in vitro

A 10 mM solution of dissectol A in DMSO was prepared and stored at -20 C. The laboratory strain of *Mycobacterium tuberculosis* H37Rv was cultured in Middlebrook 7H9 media supplemented with 0.2% (w/v) glucose, 0.5 g/L fatty-acid free bovine serum albumin, 0.085% (w/v) NaCl, 50 mM glycerol and 0.04% (v/v) tyloxapol to an OD_{600} of 0.8. From this culture, cells were transferred in 1 mL aliquots to a sterile nylon filter and media was removed by vacuum filtration. The deposited cells were transferred to Middlebrook 7H10 agar plates supplemented with 0.2% (w/v) glucose, 0.5 g/L fatty-acid free bovine serum albumin, 0.085% (w/v) NaCl, and 50 mM glycerol and incubated at 37 C supplemented with 5% CO_2 for five days. The cell-laden filter was transferred to a 6-well plate containing modified Sauton's media containing 6% glycerol and incubated for 24 hrs at 37 C with 5% CO_2. The filter-mounted cells were then transferred to a new 6-well plate containing 10 µM dissectol A and incubated at 37 ^{o}C with 5% CO_2 for 24 hrs. Cells were lysed into 1 mL of 2:2:1 HPLC-grade methanol:acetonitrile:water containing 250 uL of 0.1 mm zirconia beads using a cell homogenizer set to 6000 rpm x 30 seconds x 3 cycles at 4 ^{o}C. Aliquots (650 µL) of lysate, spent media and input media were filter sterilized by passing through a 0.2 um SpinX column. Abundance of dissectol A was measured using LC-MS time of flight (Agilent 1200 LC, 6220 MS TOF) by detecting the [M+H] ionized species at m/z 331.172. Normal phase chromatography using a Cogent Diamond Hydride type C column stationary phase and a gradient mobile phase from acetonitrile with 0.2% (v/v) formic acid to ddH_2O with 0.2% (v/v) formic acid was performed. We assume that the difference in abundance between the input media and spent media is equal to the amount taken up by the cell and calculate % Uptake and % Present in cell using the following formulae:

% Uptake = 100*(1 – [Spent Media]/[Input media])

% Present in cells = 100*([Lysate]/[Input media])

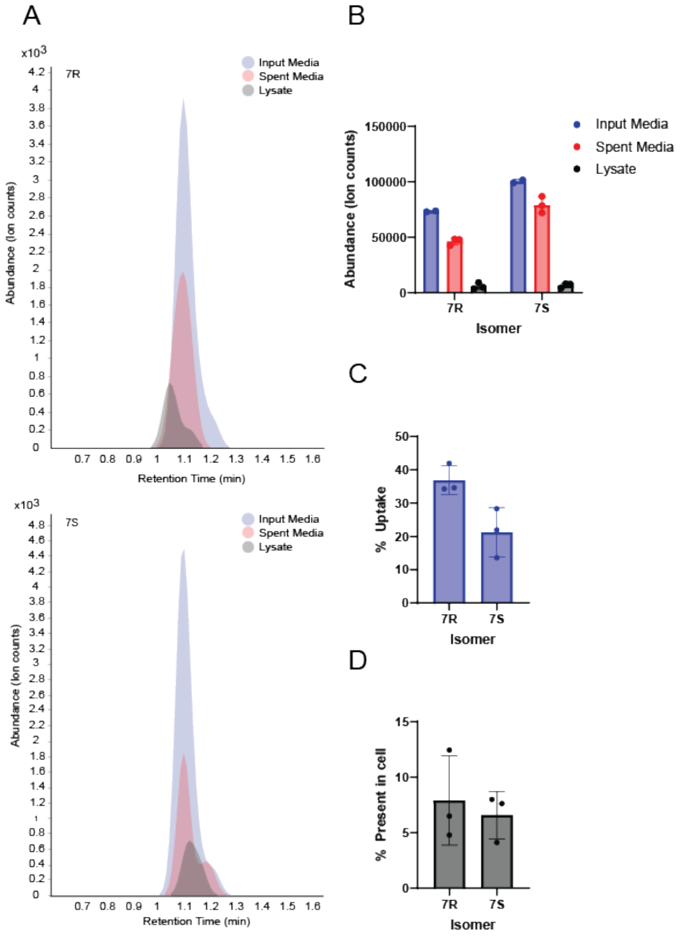

Figure S1: Uptake of dissectol A by *M. tuberculosis*: A) Abundance of $[M+H]^+$ m/z 331.172 at retention time 1.1 min by LC-MS TOF comparing input media, spent media, and cell lysate. B) Abundance of dissectol A isomers quantified by summing total ion counts under the curve from RT 0.9 – 1.3 minutes. C) % Uptake for each isomer. D) % of each isomer present in cell.

Growth of Mtb in the presence of dissectol A

Laboratory strain H37Rv was cultured in 7H9 media supplemented with 0.2% (w/v) glucose, 0.5 g/L fatty-acid free bovine serum albumin, 0.085% (w/v) NaCl, 50 mM glycerol and 0.04% (v/v) tyloxapol in 96-well plates in the presence of dissectol from 0 μM to 500 μM. Cultures were incubated at 37 C with 5% CO2. Optical density at 600 nm (OD_{600}) readings were obtained at regular intervals. Two independent experiments were performed, and each experiment included three technical replicates.

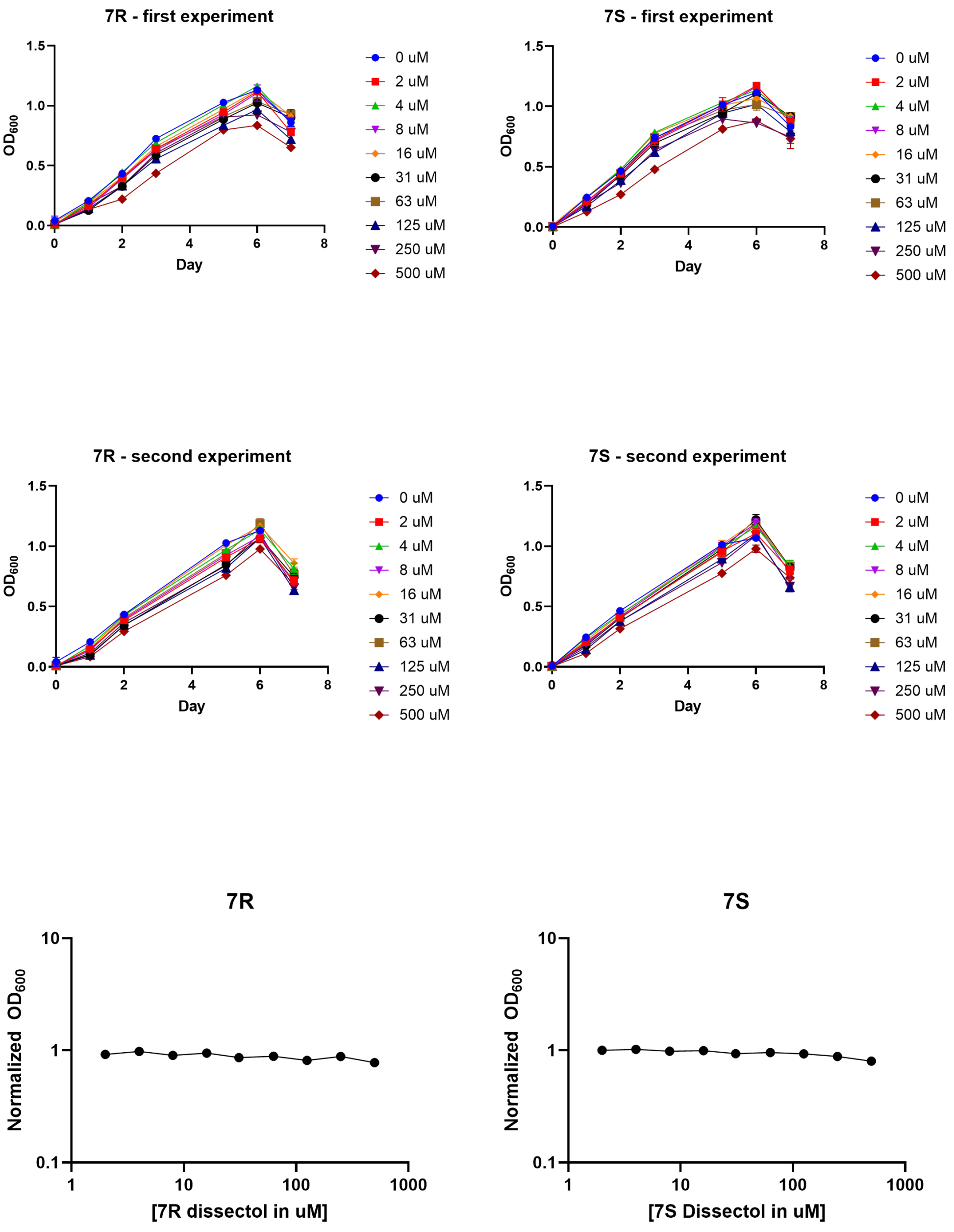

Figure S2: Growth of M. tuberculosis in the presence of dissectol A. OD_{600} was monitored over a 7 day incubation of laboratory strain H37Rv in 7H9 media in the presence of 0 – 500 μM dissectol. OD_{600} readings from day 5 of the incubation, representing late log phase growth, were normalized to the 0 μM control and plotted against the full range of concentrations. No impairment in growth was detected for either epimer.

Whole Cell M. tb DevRS reporter assay

The reporter assay was conducted as previously described by Zheng et al., 2017. Both dissectol A **33A** (7Rf1) and its methyl-epimer **32A** (7Sf1) were tested in a DevRS reporter assay. For this assay, the CDC1551(*hspX'*::GFP) reporter

was used that has strong GFP fluorescence induced by hypoxia following growth for 6 days in a 96 well plate. Both GFP fluorescence and optical density (OD) were measured. Percent inhibition was normalized with a DMSO control (0% inhibition) and rifampin (100% inhibition; no growth). As a positive control, a recent analog of HC106, MSU-43672 was used (cite Zheng et al., 2020). Strong inhibition of reporter fluorescence is observed with the latter inhibitor (EC50 ~200 nM). However, no inhibition of the reporter fluorescence is observed with **33A** and **32A**.

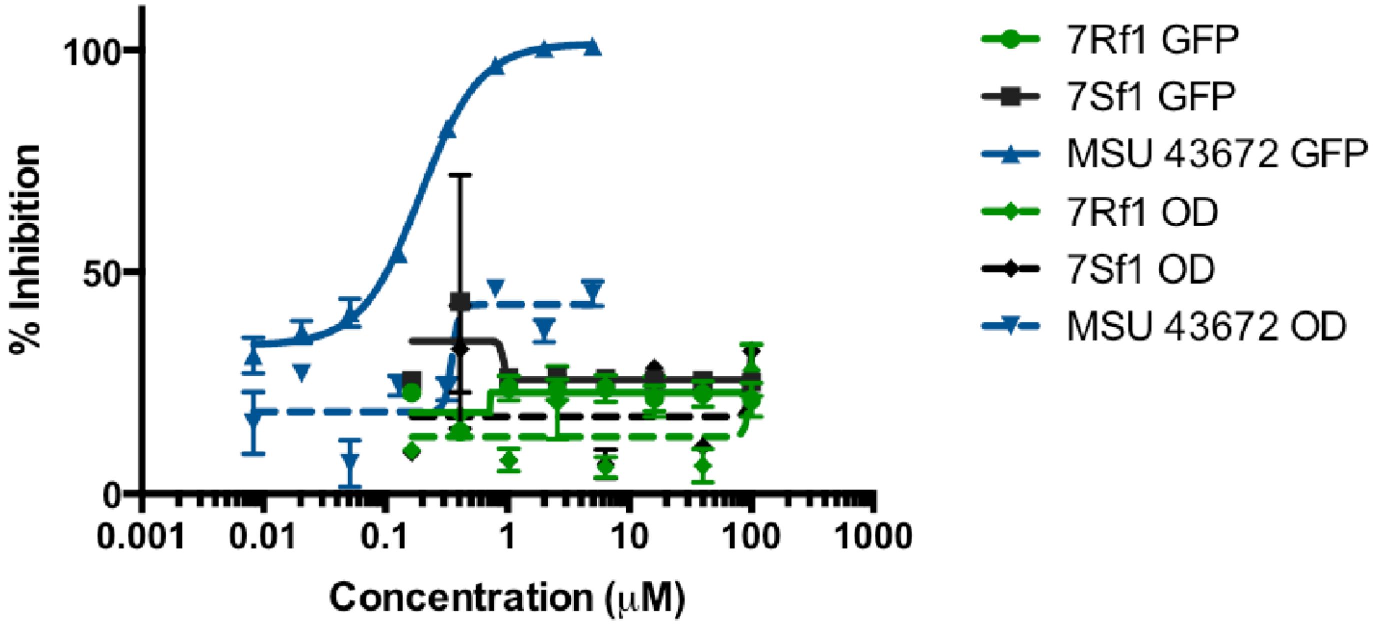

	MSU 43672	7Rf1	7Sf1
HillSlope	1.775	ND	ND
EC50	0.1938	ND	ND
EC50 95% CI	0.1609 to 0.2334	ND	ND
EC90	0.668	ND	ND

3. References

(1) Conley, N. R.; Labios, L. A.; Pearson, D. M.; McCrory, C. C. L.; Waymouth, R. M. *Organometallics* **2007**, *26*, 5447–5453.

(2) Smith, C. R.; Mans, D. J.; RajanBabu, T. V. *Org. Synth.* **2008**, *85*, 238.

(3) Doyle, L. M.; O'Sullivan, S.; Di Salvo, C.; McKinney, M.; McArdle, P.; Murphy, P. V. *Org. Lett.* **2017**, *19*, 5802–5805.

(4) Rokade, S. M.; Bhate, P. M. *Carbohydr. Res.* **2015**, *416*, 21–23.

(4a) Andersen, S. M.; Heuckendorff, M.; Jensen, H. H. *Org. Lett.* **2015**, *17*, 944–947.

(5) Bravo, F.; McDonald, F. E.; Neiwert, W. A.; Do, B.; Hardcastle, K. I. *Org. Lett.* **2003**, *5*, 2123–2126.

(6) Barrett, A. G. M.; Beall, J. C.; Braddock, D. C.; Flack, K.; Gibson, V. C.; Salter, M. M. *J. Org. Chem.* **2000**, *65*, 6508–6514.

(7) Willand-Charnley, R.; Fisher, T. J.; Johnson, B. M.; Dussault, P. H. *Org. Lett.* **2012**, *14*, 2242–2245.

(8) Ferrer, C.; Fodran, P.; Barroso, S.; Gibson, R.; Hopmans, E. C.; Damsté, J. S.; Schouten, S.; Minnaard, A. J. *Org. Biomol. Chem.* **2013**, *11*, 2482–2492.

(9) Casas-Arce, E.; Ter Horst, B.; Feringa, B. L.; Minnaard, A. J. *Chem. - A Eur. J.* **2008**, *14*, 4157–4159.

(10) Zhang, X.; Rui, M.; Xu, H.; Chou, G. *Chem. Biodivers.* **2020**, *17*.

(11) Chen, W.; Shen, Y.; Xu, J. *Planta Med.* **2003**, *69*, 579–582.

(12) Chen, W.; Shen, Y.; Xu, J. *Chinese Chem. Lett.* **2003**, *14*, 385–388.

4. NMR Spectra (in CD_3OD)

1H NMR comparison of natural isolate vs. synthetic dissectol A

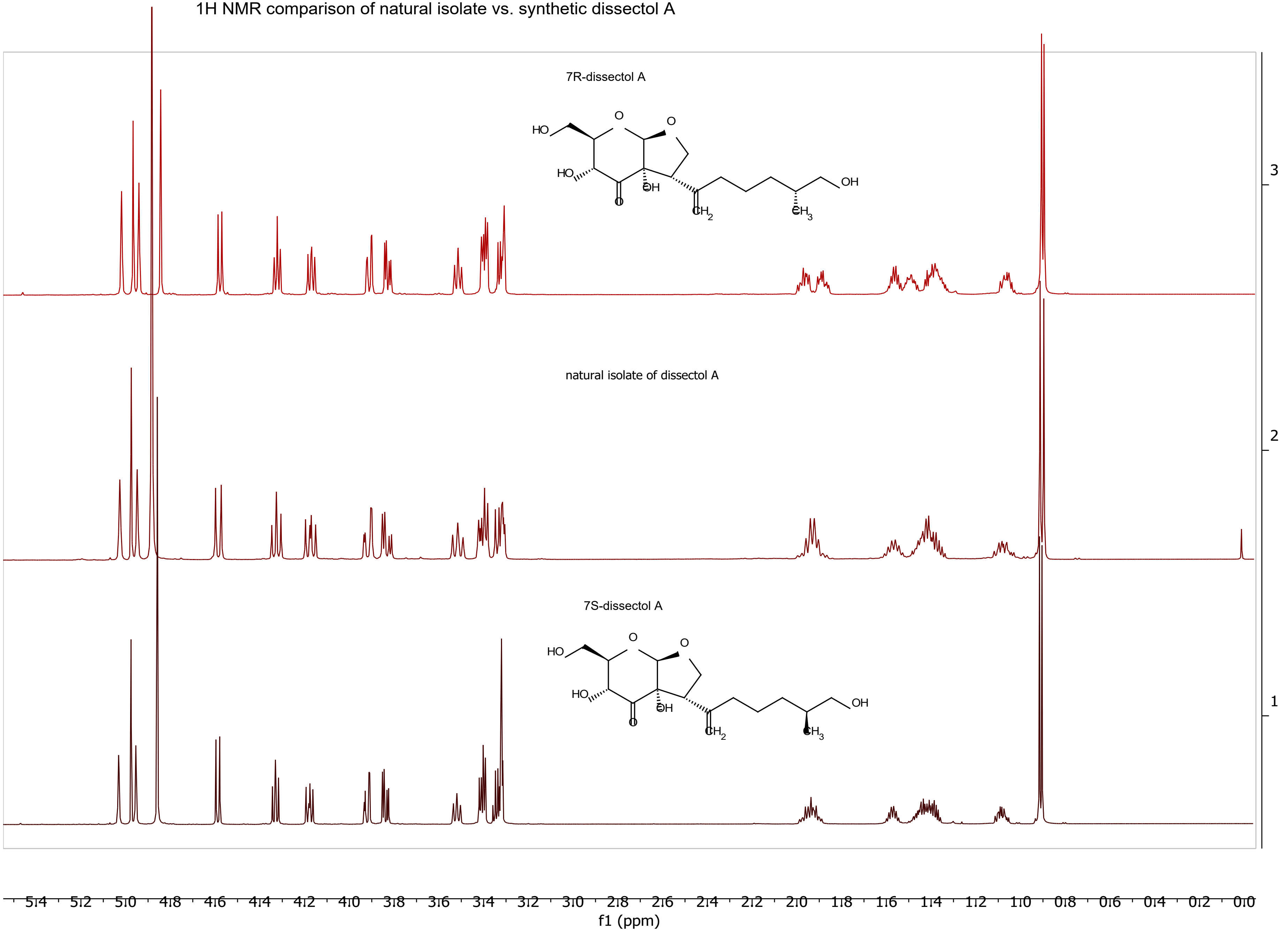

1H NMR comparison of natural isolate vs. synthetic dissectol A (zoom-in)

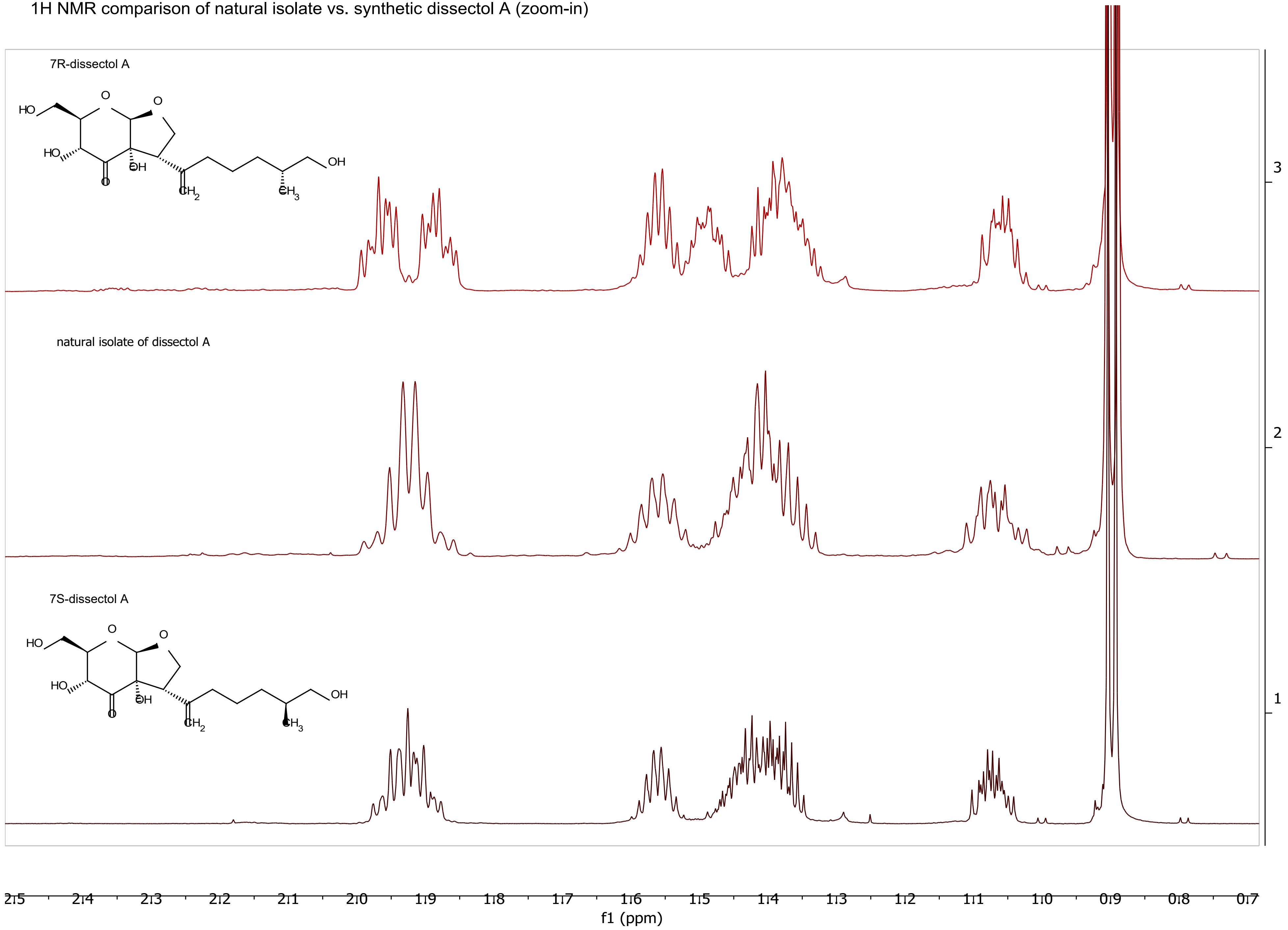

13C NMR comparison of natural isolate vs. synthetic dissectol A

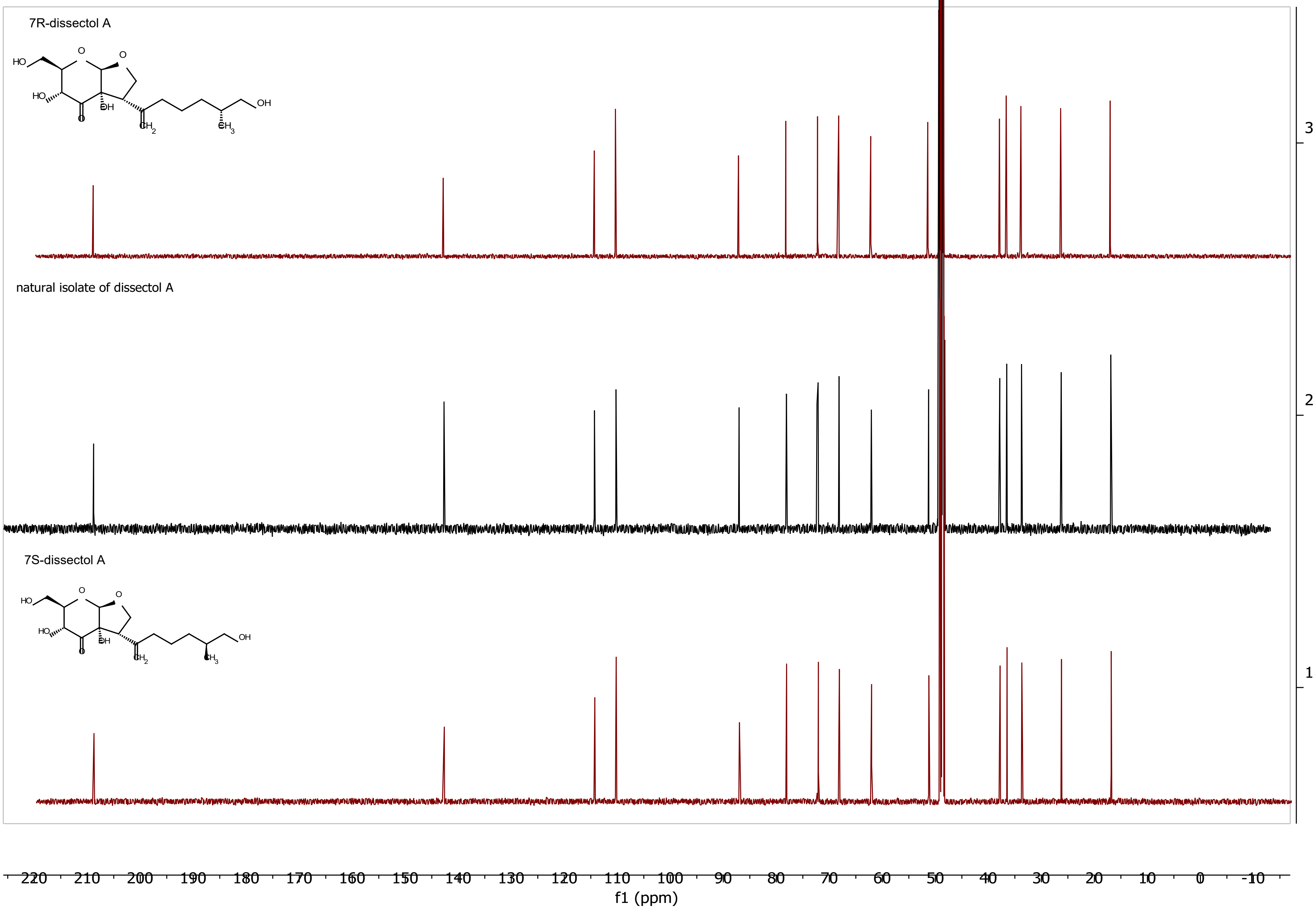

Appendix: NMR spectra

Total Synthesis of Dissectol A and Serratumin A

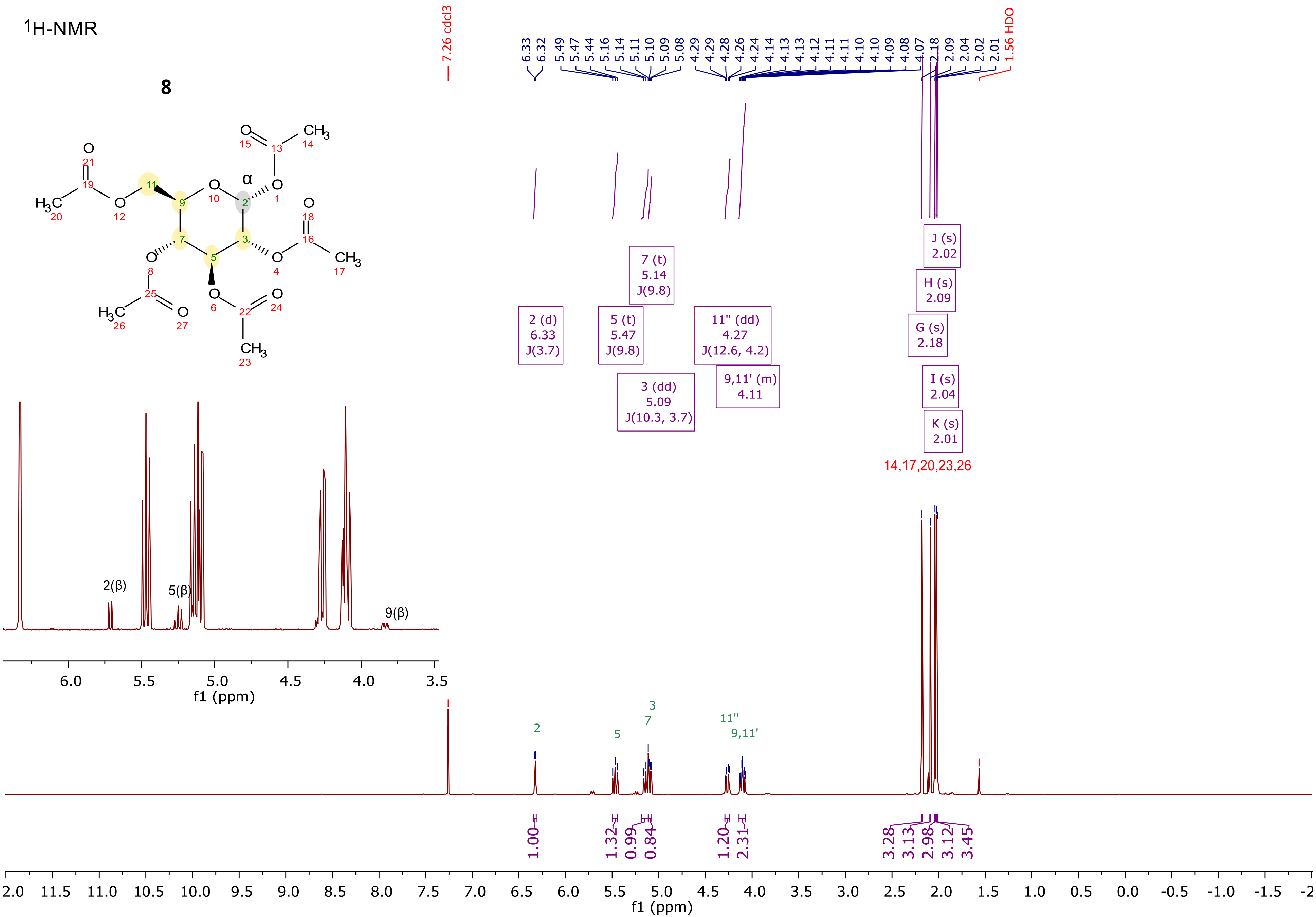

1H-NMR
8
α
7.26 cdcl3
1.56 HDO
2 (d) 6.33 J(3.7)
5 (t) 5.47 J(9.8)
7 (t) 5.14 J(9.8)
3 (dd) 5.09 J(10.3, 3.7)
11'' (dd) 4.27 J(12.6, 4.2)
9,11' (m) 4.11
G (s) 2.18
H (s) 2.09
I (s) 2.04
J (s) 2.02
K (s) 2.01
14,17,20,23,26
2(β)
5(β)
9(β)
f1 (ppm)

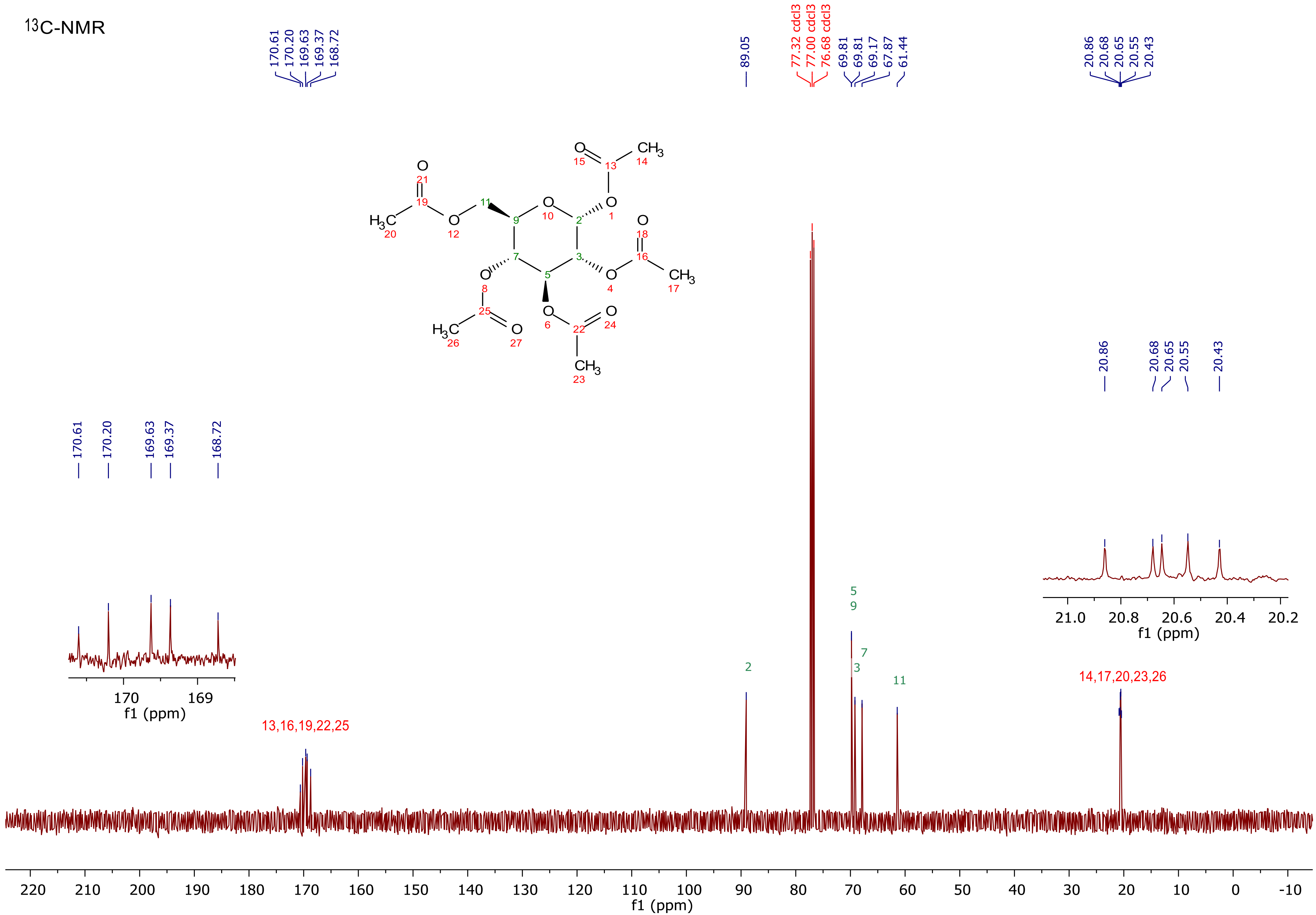

13C-NMR
170.61
170.20
169.63
169.37
168.72
89.05
77.32 cdcl3
77.00 cdcl3
76.68 cdcl3
69.81
69.81
69.17
67.87
61.44
20.86
20.68
20.65
20.55
20.43
13,16,19,22,25
2
5
9
7
3
11
14,17,20,23,26
f1 (ppm)

^{1}H-NMR

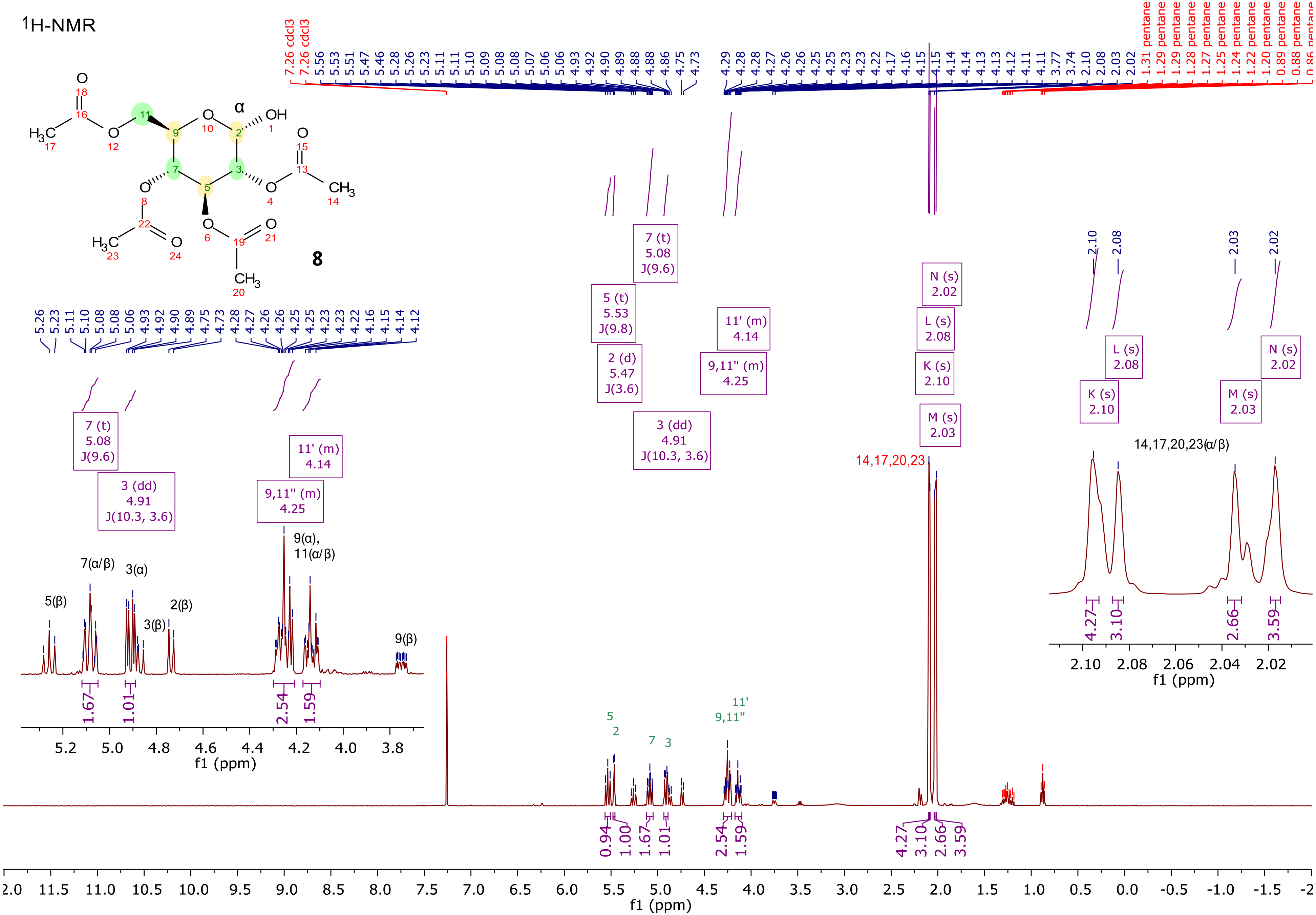

8
14,17,20,23
14,17,20,23(α/β)
5 (t) 5.53 J(9.8)
2 (d) 5.47 J(3.6)
7 (t) 5.08 J(9.6)
3 (dd) 4.91 J(10.3, 3.6)
11' (m) 4.14
9,11'' (m) 4.25
N (s) 2.02
L (s) 2.08
K (s) 2.10
M (s) 2.03
5(β)
7(α/β)
3(α)
3(β)
2(β)
9(α), 11(α/β)
9(β)
f1 (ppm)

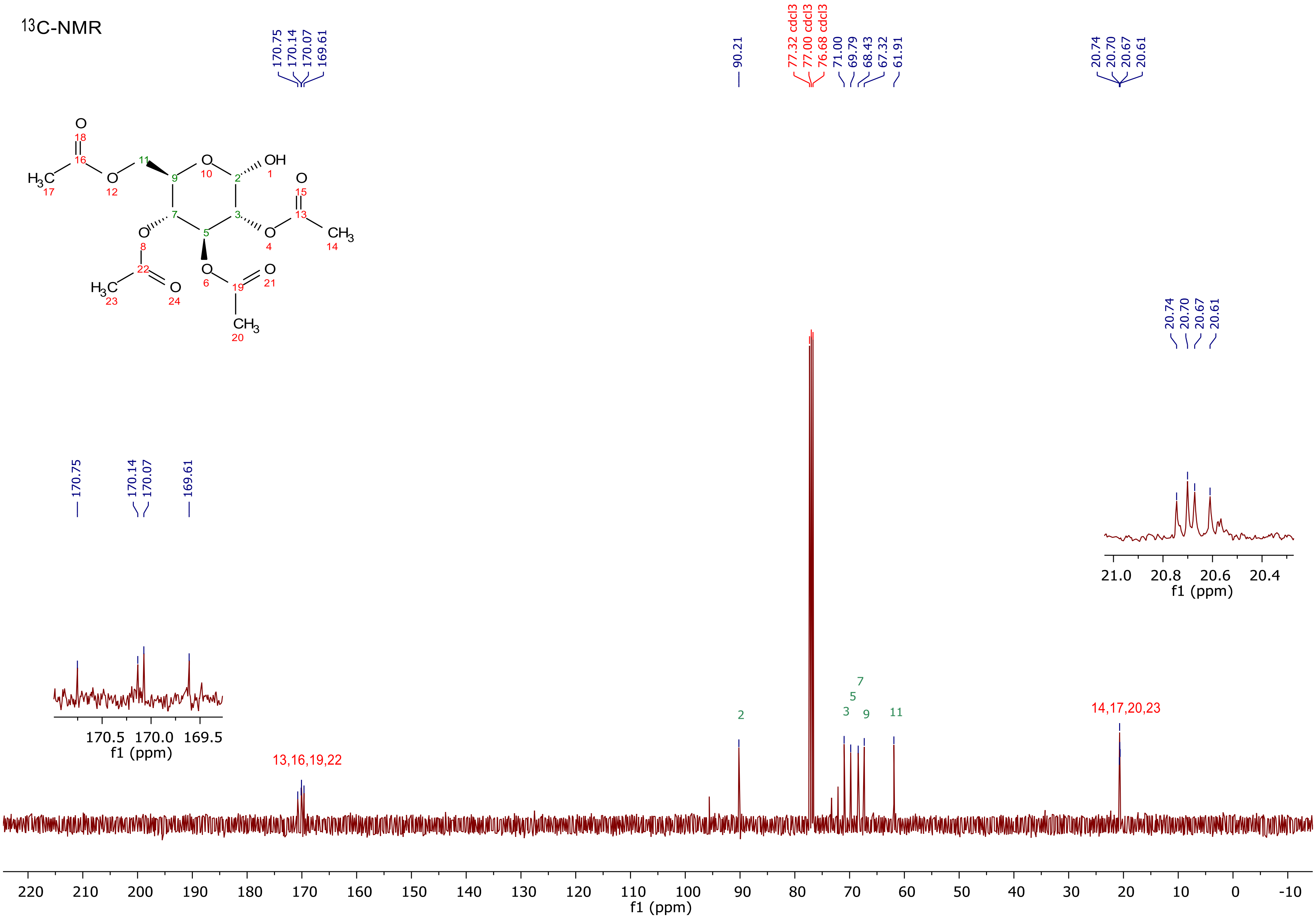

13C-NMR
170.75
170.14
170.07
169.61
90.21
77.32 cdcl3
77.00 cdcl3
76.68 cdcl3
71.00
69.79
68.43
67.32
61.91
20.74
20.70
20.67
20.61
13,16,19,22
2
3
5
7
9
11
14,17,20,23
f1 (ppm)

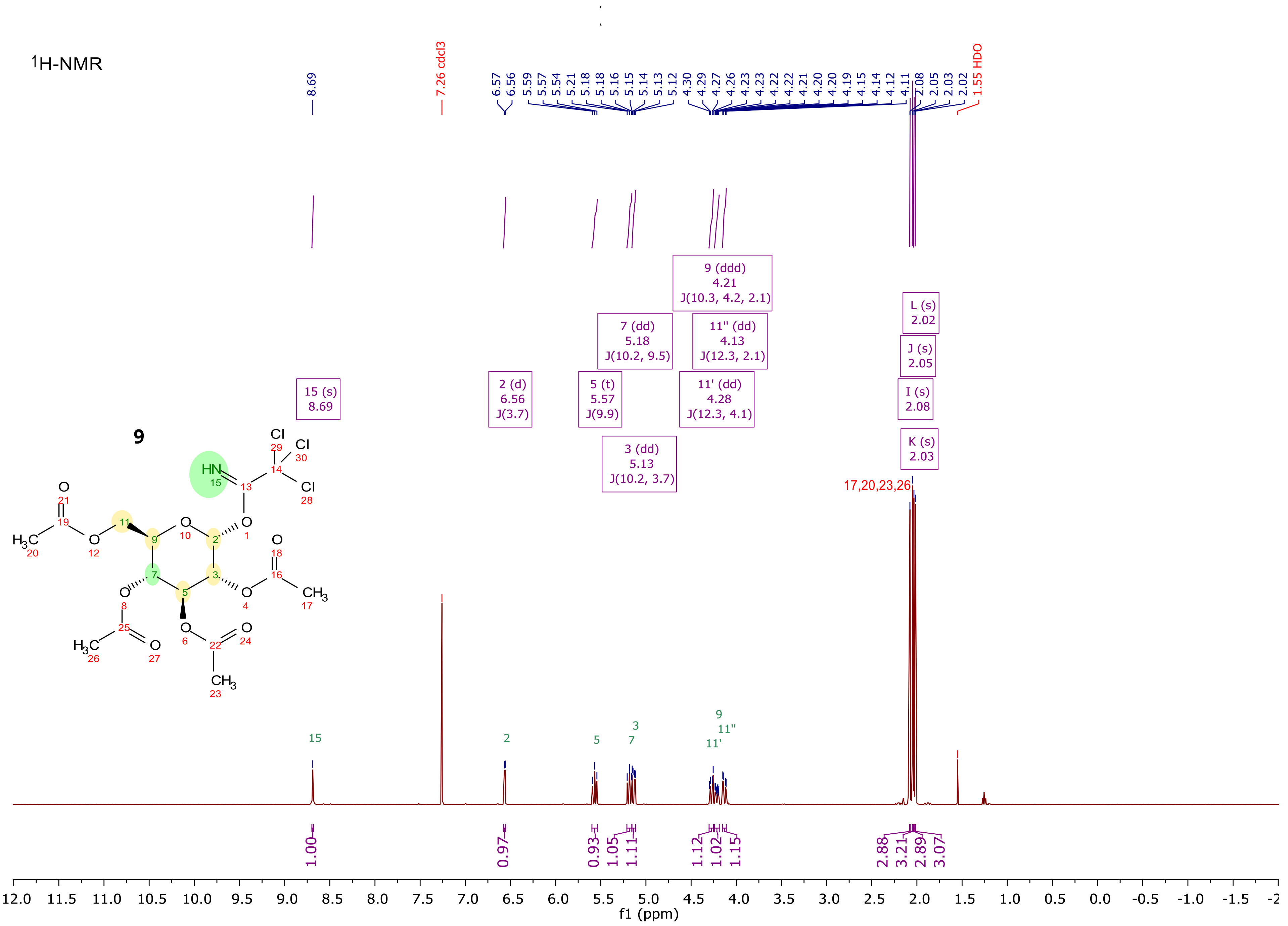

1H-NMR
8.69
7.26 cdcl3
6.57
6.56
5.59
5.57
5.54
5.21
5.18
5.18
5.16
5.15
5.14
5.13
5.12
4.30
4.29
4.27
4.26
4.23
4.23
4.22
4.22
4.21
4.20
4.20
4.19
4.15
4.14
4.12
4.11
2.08
2.05
2.03
2.02
1.55 HDO
9 (ddd) 4.21 J(10.3, 4.2, 2.1)
7 (dd) 5.18 J(10.2, 9.5)
11'' (dd) 4.13 J(12.3, 2.1)
15 (s) 8.69
2 (d) 6.56 J(3.7)
5 (t) 5.57 J(9.9)
11' (dd) 4.28 J(12.3, 4.1)
3 (dd) 5.13 J(10.2, 3.7)
L (s) 2.02
J (s) 2.05
I (s) 2.08
K (s) 2.03
17,20,23,26
9
1.00
0.97
0.93
1.05
1.11
1.12
1.02
1.15
2.88
3.21
2.89
3.07
f1 (ppm)

^{13}C-NMR

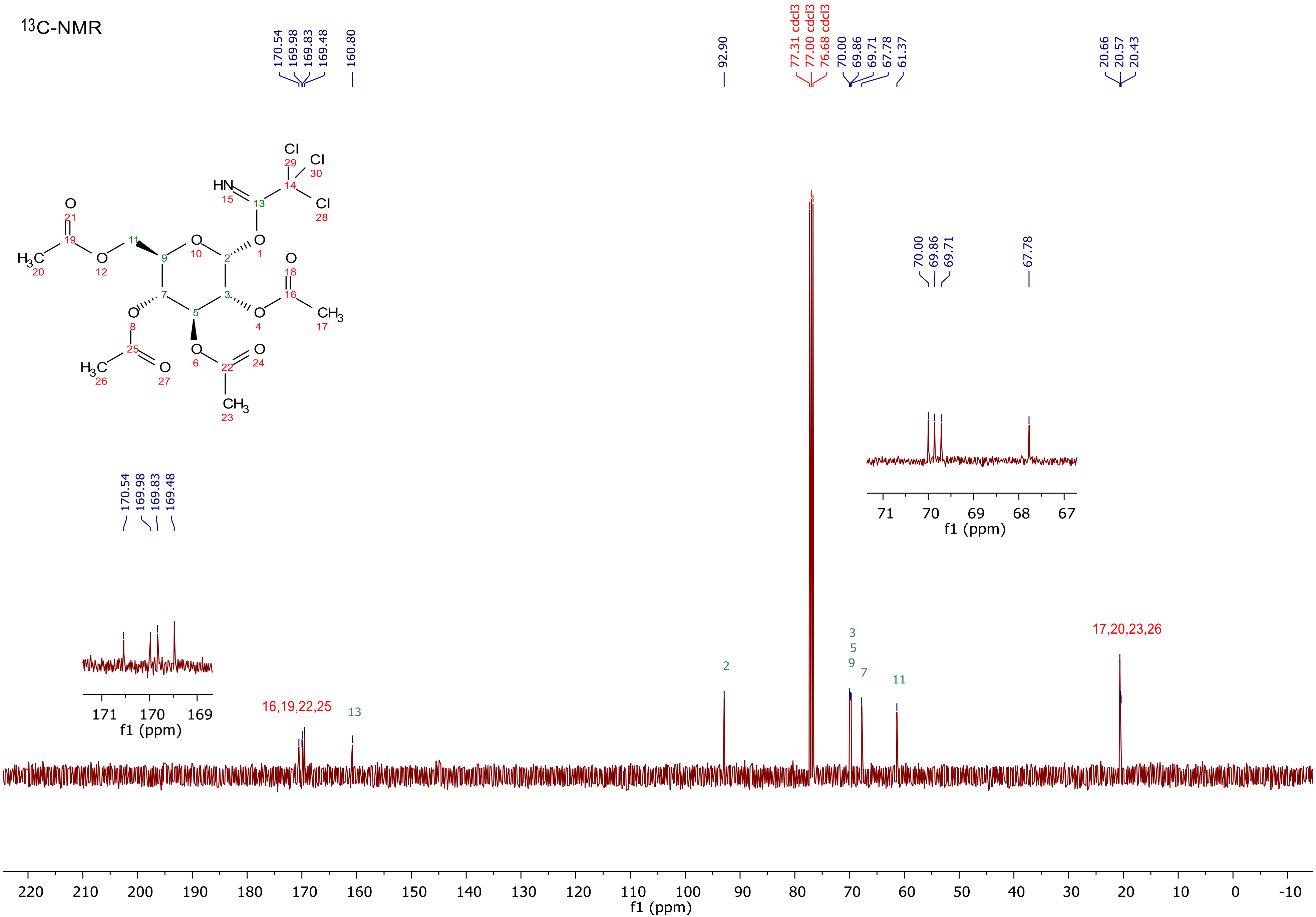

170.54
169.98
169.83
169.48
160.80
92.90
77.31 cdcl3
77.00 cdcl3
76.68 cdcl3
70.00
69.86
69.71
67.78
61.37
20.66
20.57
20.43
16,19,22,25
13
2
3
5
9
7
11
17,20,23,26
f1 (ppm)

^{1}H-NMR

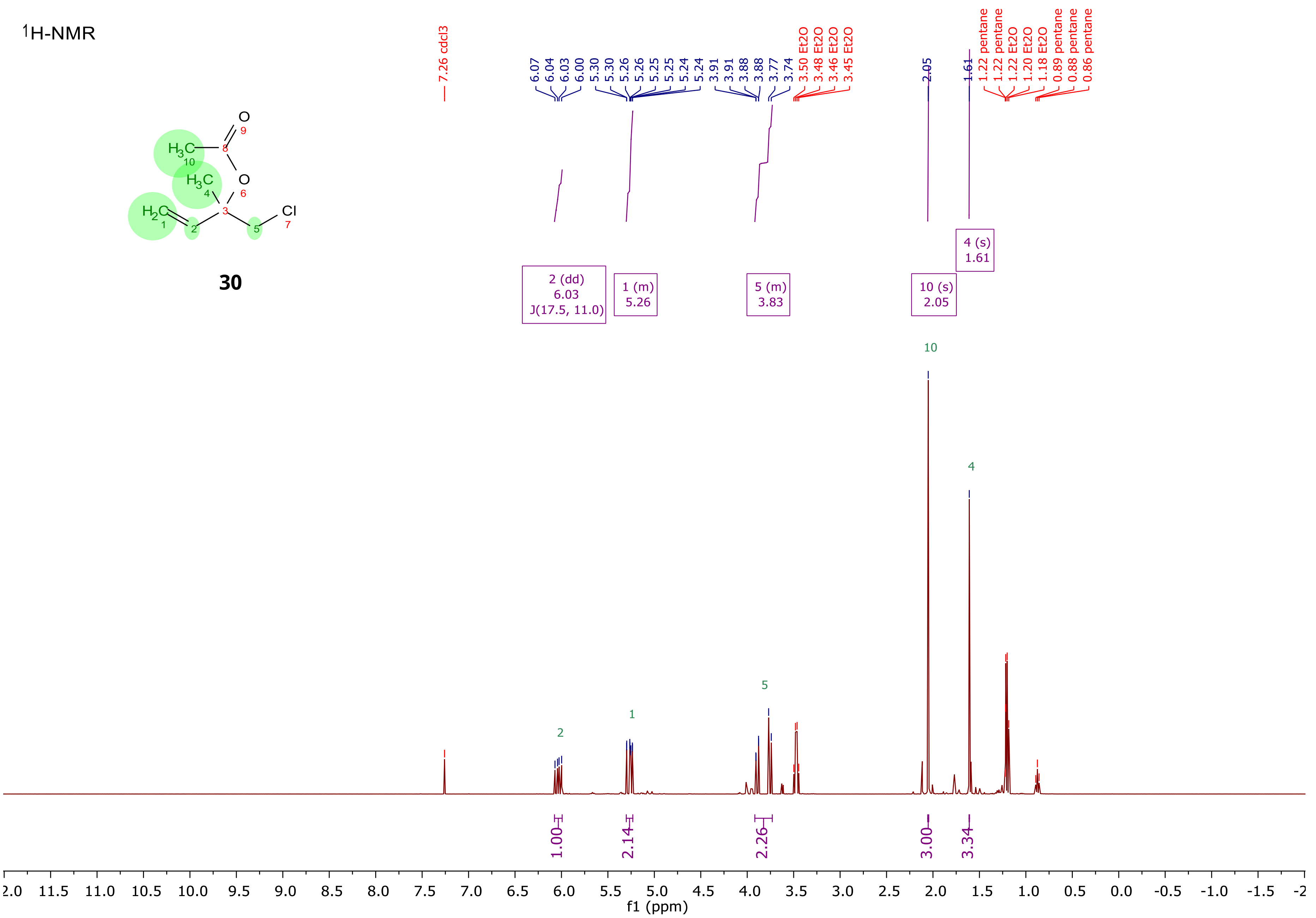

O
9
H3C
10
8
H3C
4
O
6
H2C
1
2
3
5
Cl
7
7.26 cdcl3
6.07
6.04
6.03
6.00
5.30
5.30
5.26
5.26
5.25
5.25
5.24
5.24
3.91
3.91
3.88
3.88
3.77
3.74
3.50 Et2O
3.48 Et2O
3.46 Et2O
3.45 Et2O
2.05
1.61
1.22 pentane
1.22 pentane
1.22 Et2O
1.20 Et2O
1.18 Et2O
0.89 pentane
0.88 pentane
0.86 pentane
2 (dd)
6.03
J(17.5, 11.0)
1 (m)
5.26
5 (m)
3.83
10 (s)
2.05
4 (s)
1.61
10
4
5
1
2
1.00
2.14
2.26
3.00
3.34
f1 (ppm)

30

13C-NMR

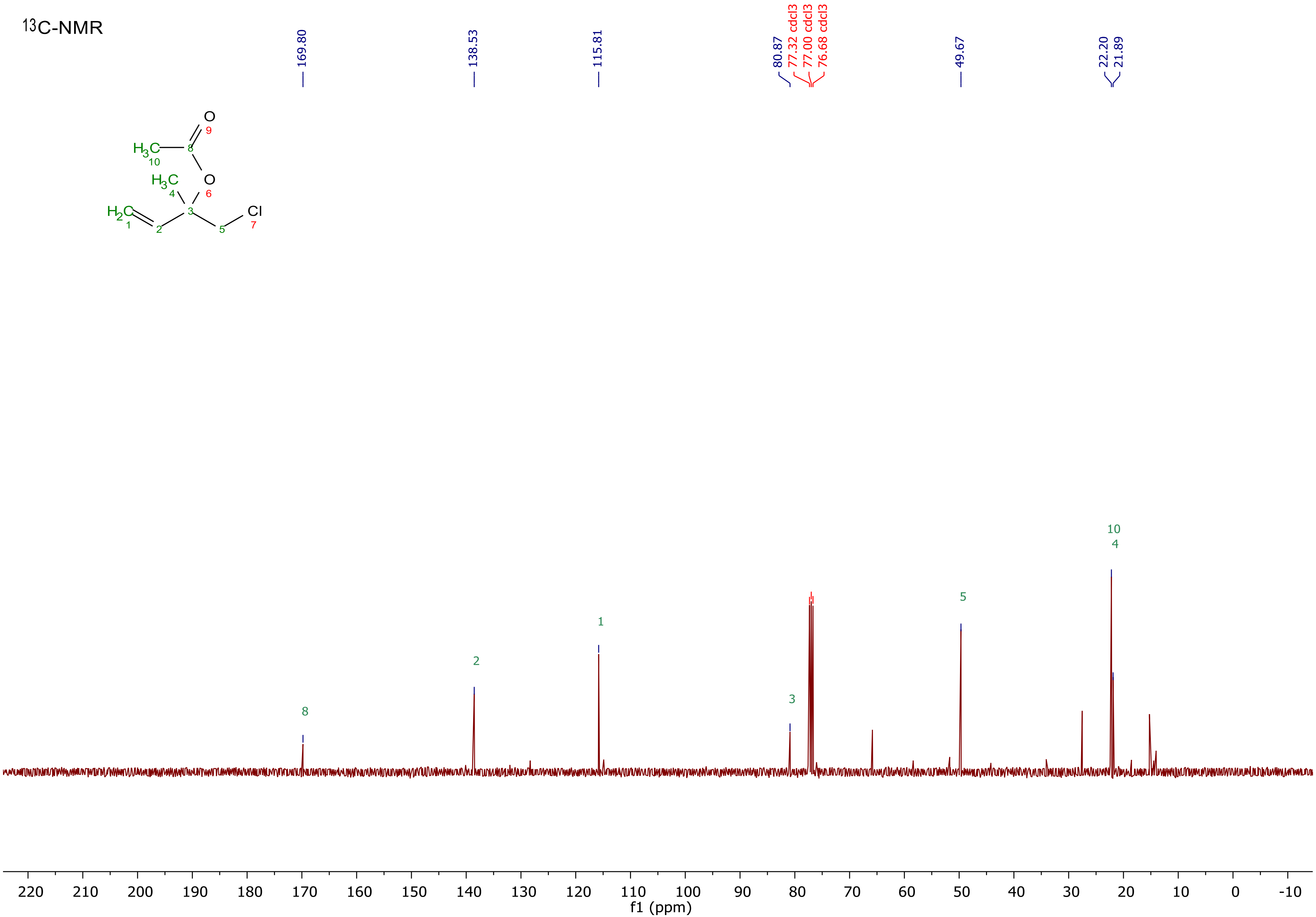

169.80
138.53
115.81
80.87
77.32 cdcl3
77.00 cdcl3
76.68 cdcl3
49.67
22.20
21.89
O
9
H_3C
8
10
H_3C
O
4
6
H_2C
3
Cl
1
2
5
7
8
2
1
3
5
10
4
220
210
200
190
180
170
160
150
140
130
120
110
100
90
80
70
60
50
40
30
20
10
0
-10
f1 (ppm)

^{1}H-NMR

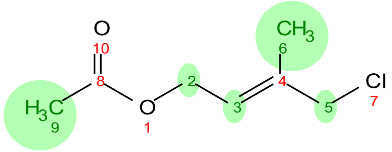

O
10
8
H3C
9
O
1
2
3
4
CH3
6
5
Cl
7

31

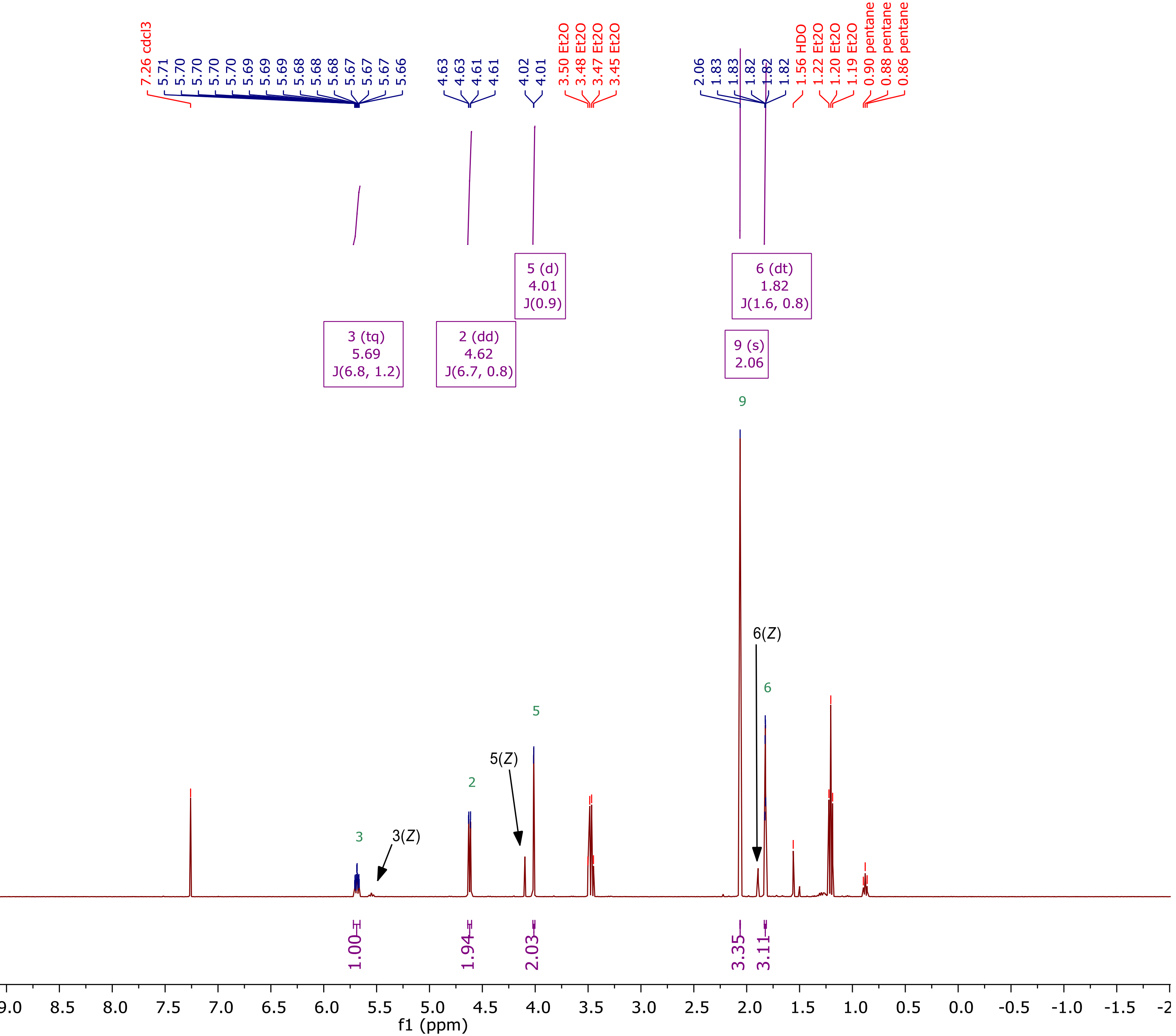

7.26 cdcl3
5.71
5.70
5.70
5.70
5.70
5.69
5.69
5.69
5.68
5.68
5.67
5.67
5.67
5.66
4.63
4.63
4.61
4.61
4.02
4.01
3.50 Et2O
3.48 Et2O
3.47 Et2O
3.45 Et2O
2.06
1.83
1.83
1.82
1.82
1.82
1.56 HDO
1.22 Et2O
1.20 Et2O
1.19 Et2O
0.90 pentane
0.88 pentane
0.86 pentane
5 (d) 4.01 J(0.9)
6 (dt) 1.82 J(1.6, 0.8)
3 (tq) 5.69 J(6.8, 1.2)
2 (dd) 4.62 J(6.7, 0.8)
9 (s) 2.06
9
6(Z)
6
5
5(Z)
2
3
3(Z)
1.00
1.94
2.03
3.35
3.11
f1 (ppm)

^{13}C-NMR

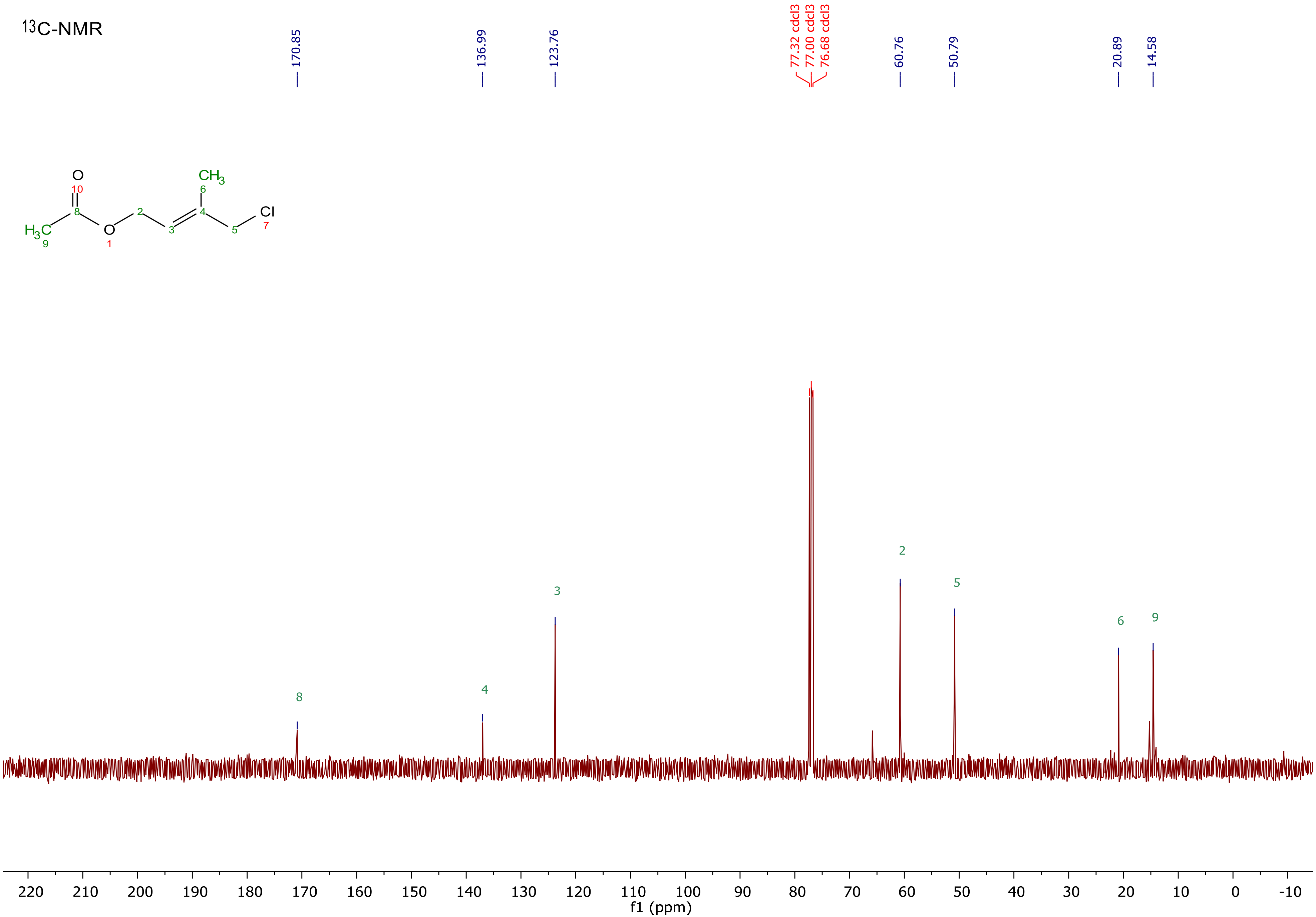

170.85
136.99
123.76
77.32 cdcl3
77.00 cdcl3
76.68 cdcl3
60.76
50.79
20.89
14.58
O
10
CH3
6
8
2
4
Cl
7
H3C
9
O
1
3
5
8
4
3
2
5
6
9
220 210 200 190 180 170 160 150 140 130 120 110 100 90 80 70 60 50 40 30 20 10 0 -10
f1 (ppm)

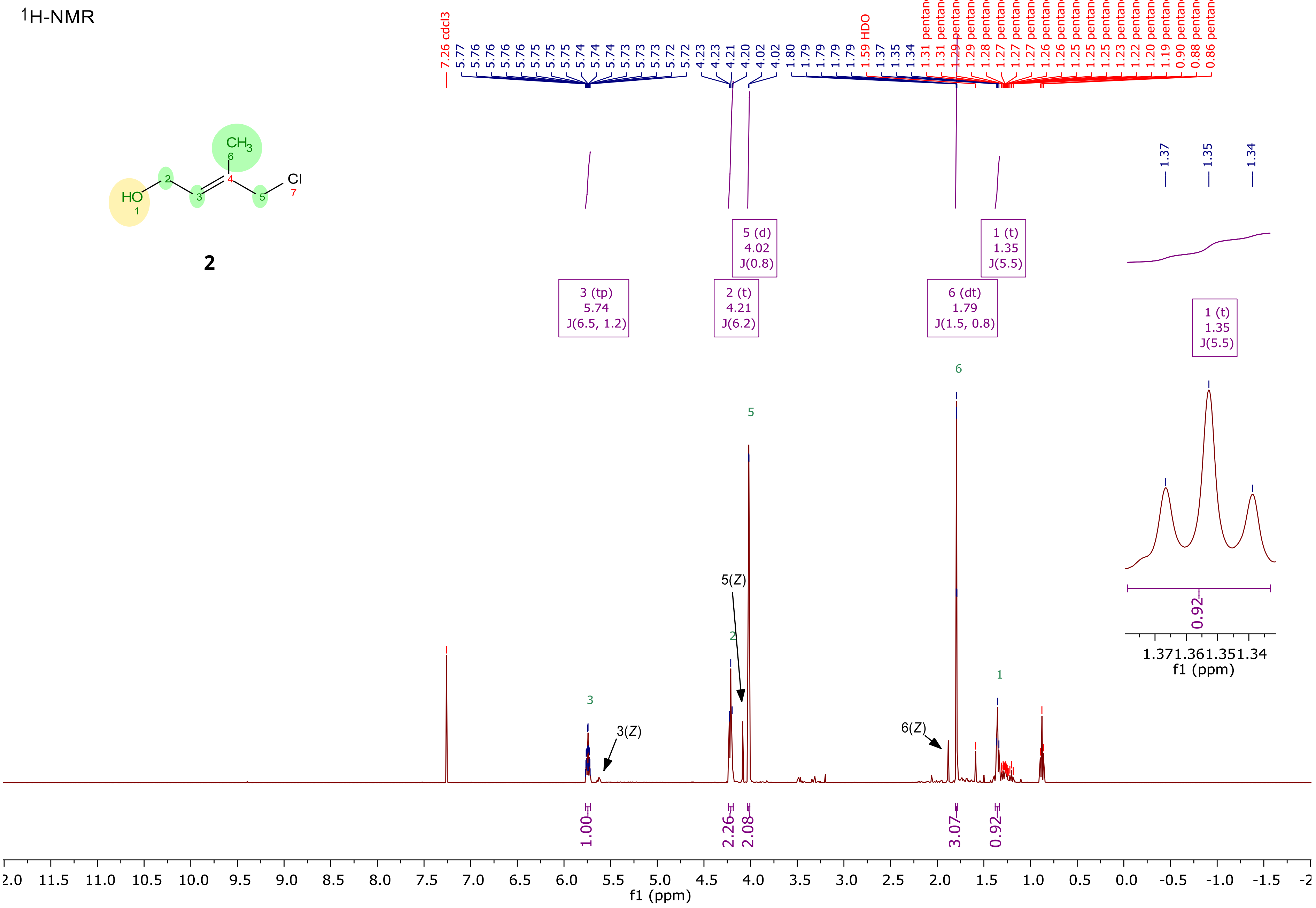

1H-NMR
HO
CH3
Cl
2
7.26 cdcl3
1.59 HDO
3 (tp) 5.74 J(6.5, 1.2)
2 (t) 4.21 J(6.2)
5 (d) 4.02 J(0.8)
6 (dt) 1.79 J(1.5, 0.8)
1 (t) 1.35 J(5.5)
5(Z)
3(Z)
6(Z)
1.00
2.26
2.08
3.07
0.92
f1 (ppm)

^{13}C-NMR

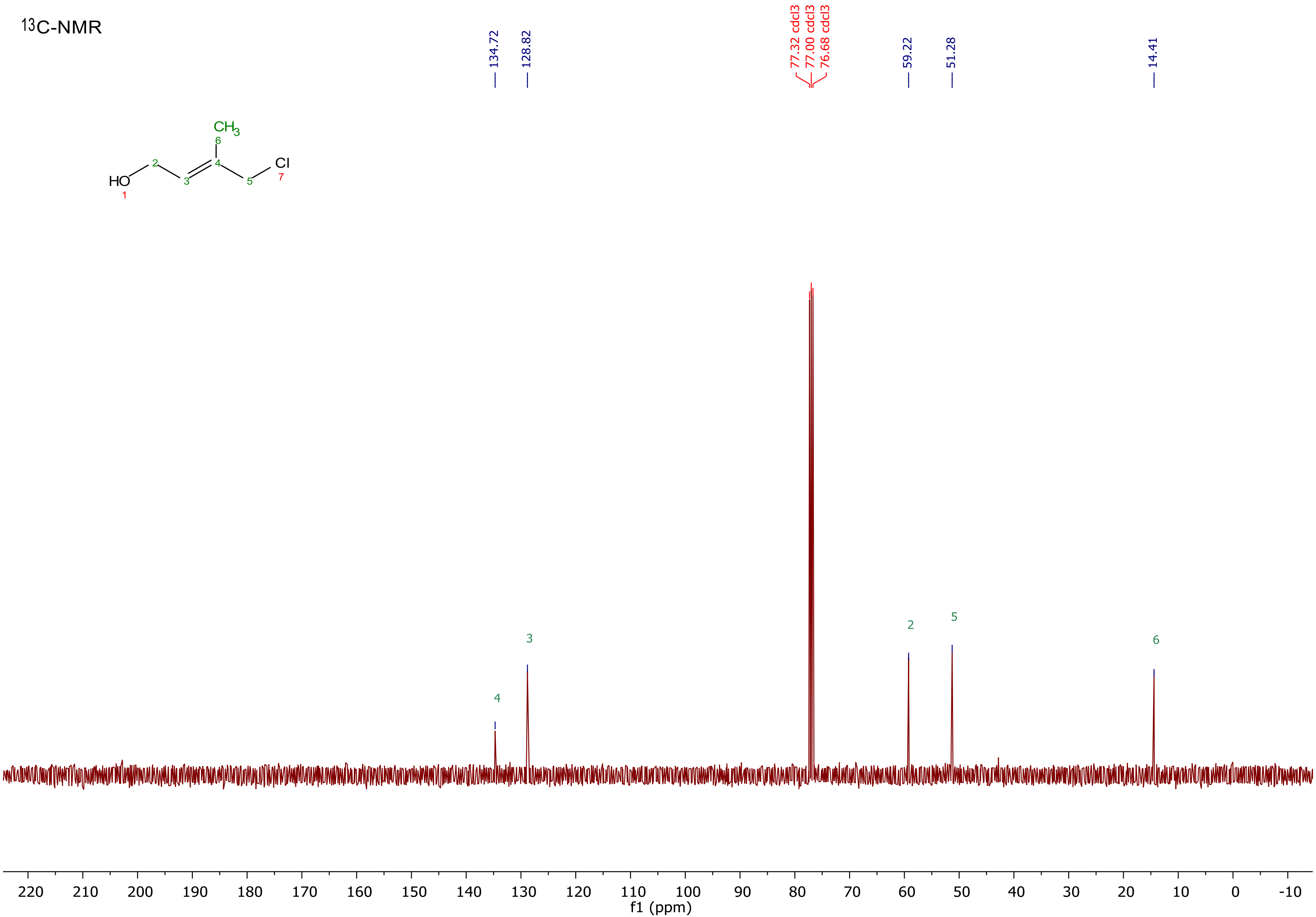

134.72
128.82
77.32 cdcl3
77.00 cdcl3
76.68 cdcl3
59.22
51.28
14.41
CH3
HO
Cl
1
2
3
4
5
6
7
220 210 200 190 180 170 160 150 140 130 120 110 100 90 80 70 60 50 40 30 20 10 0 -10
f1 (ppm)

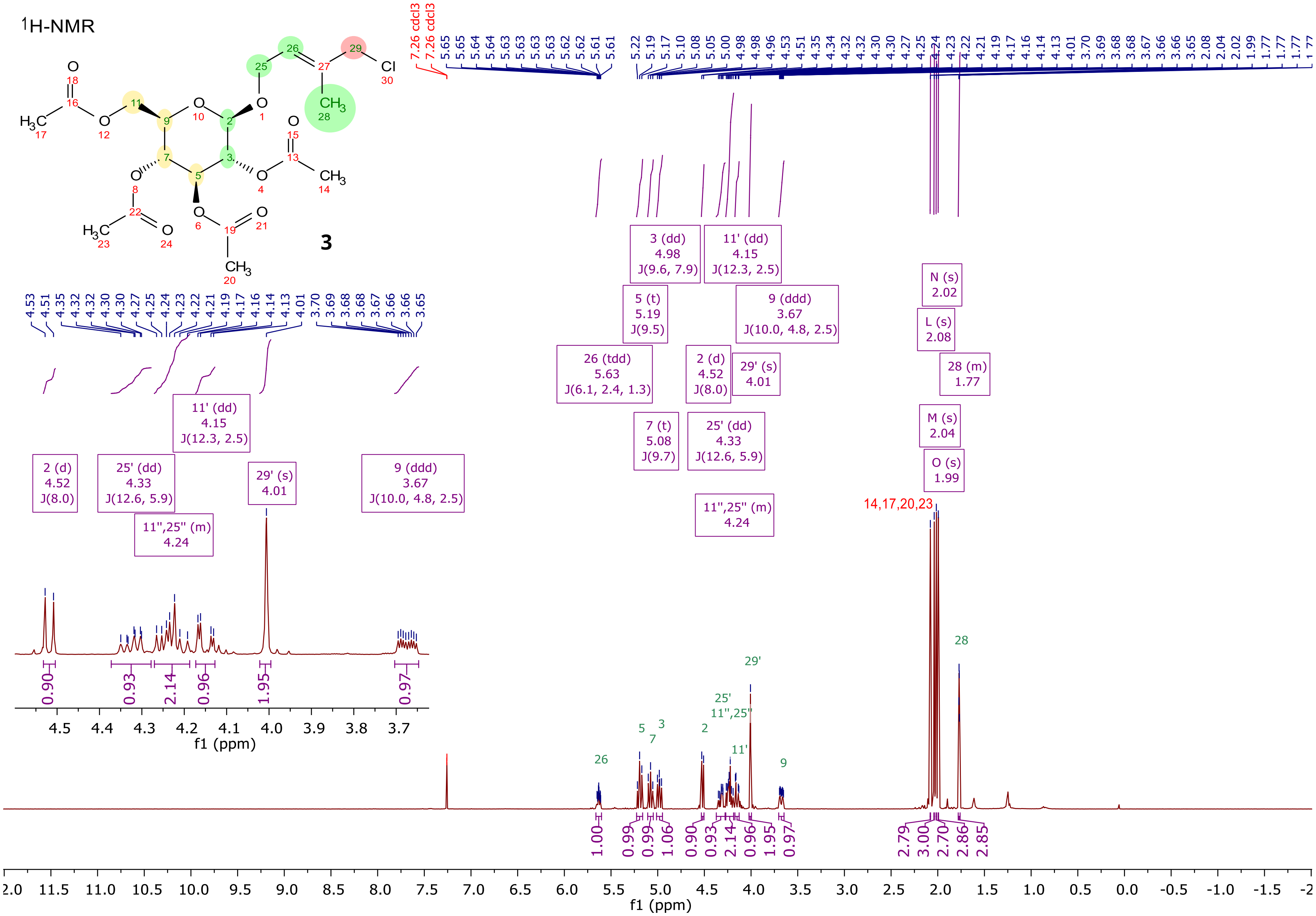

1H-NMR
3
26 (tdd) 5.63 J(6.1, 2.4, 1.3)
3 (dd) 4.98 J(9.6, 7.9)
5 (t) 5.19 J(9.5)
7 (t) 5.08 J(9.7)
2 (d) 4.52 J(8.0)
11' (dd) 4.15 J(12.3, 2.5)
9 (ddd) 3.67 J(10.0, 4.8, 2.5)
29' (s) 4.01
25' (dd) 4.33 J(12.6, 5.9)
11",25" (m) 4.24
N (s) 2.02
L (s) 2.08
28 (m) 1.77
M (s) 2.04
O (s) 1.99
14,17,20,23
f1 (ppm)

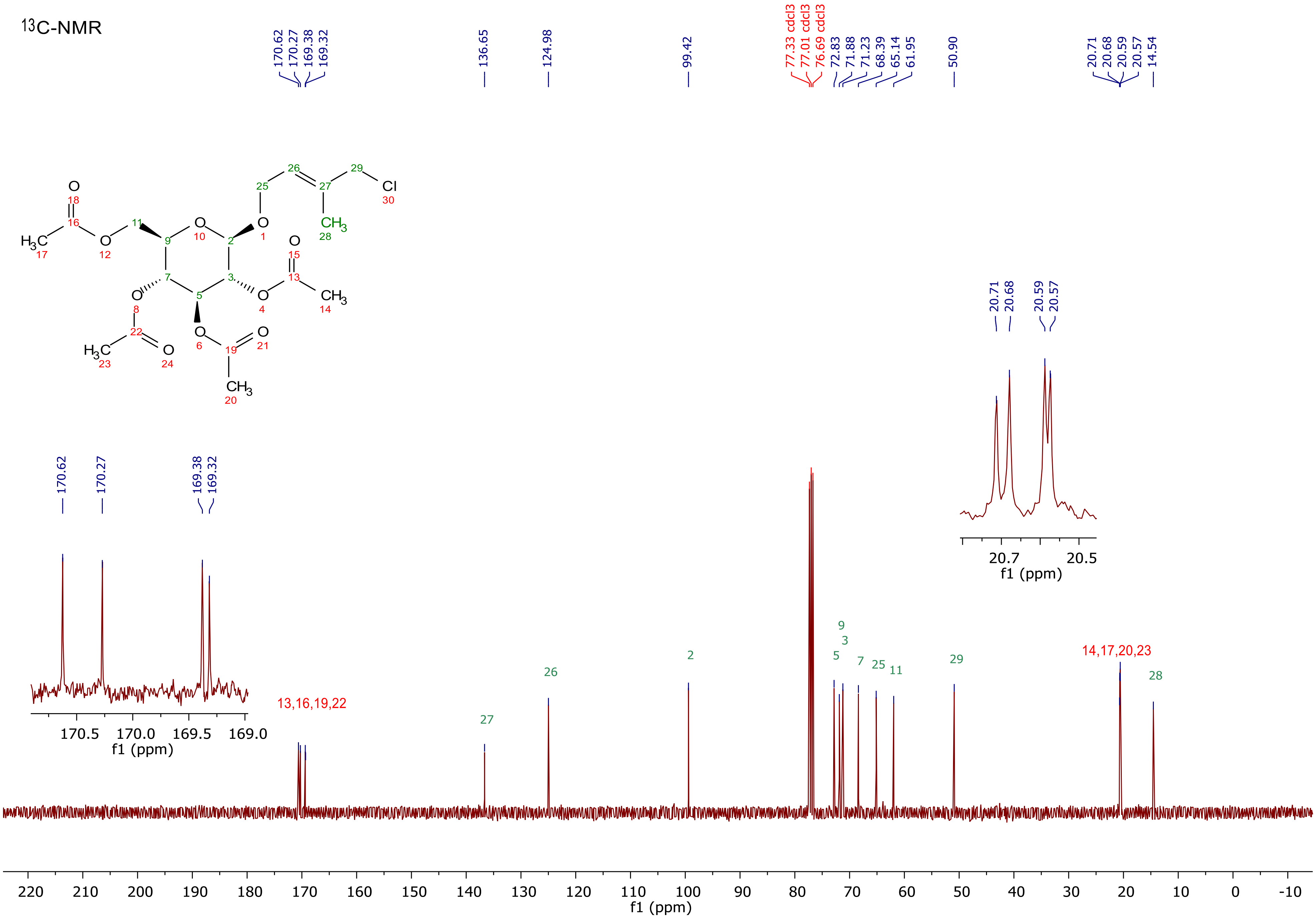

13C-NMR
170.62
170.27
169.38
169.32
136.65
124.98
99.42
77.33 cdcl3
77.01 cdcl3
76.69 cdcl3
72.83
71.88
71.23
68.39
65.14
61.95
50.90
20.71
20.68
20.59
20.57
14.54
13,16,19,22
27
26
2
9
3
5
7
25
11
29
14,17,20,23
28
f1 (ppm)

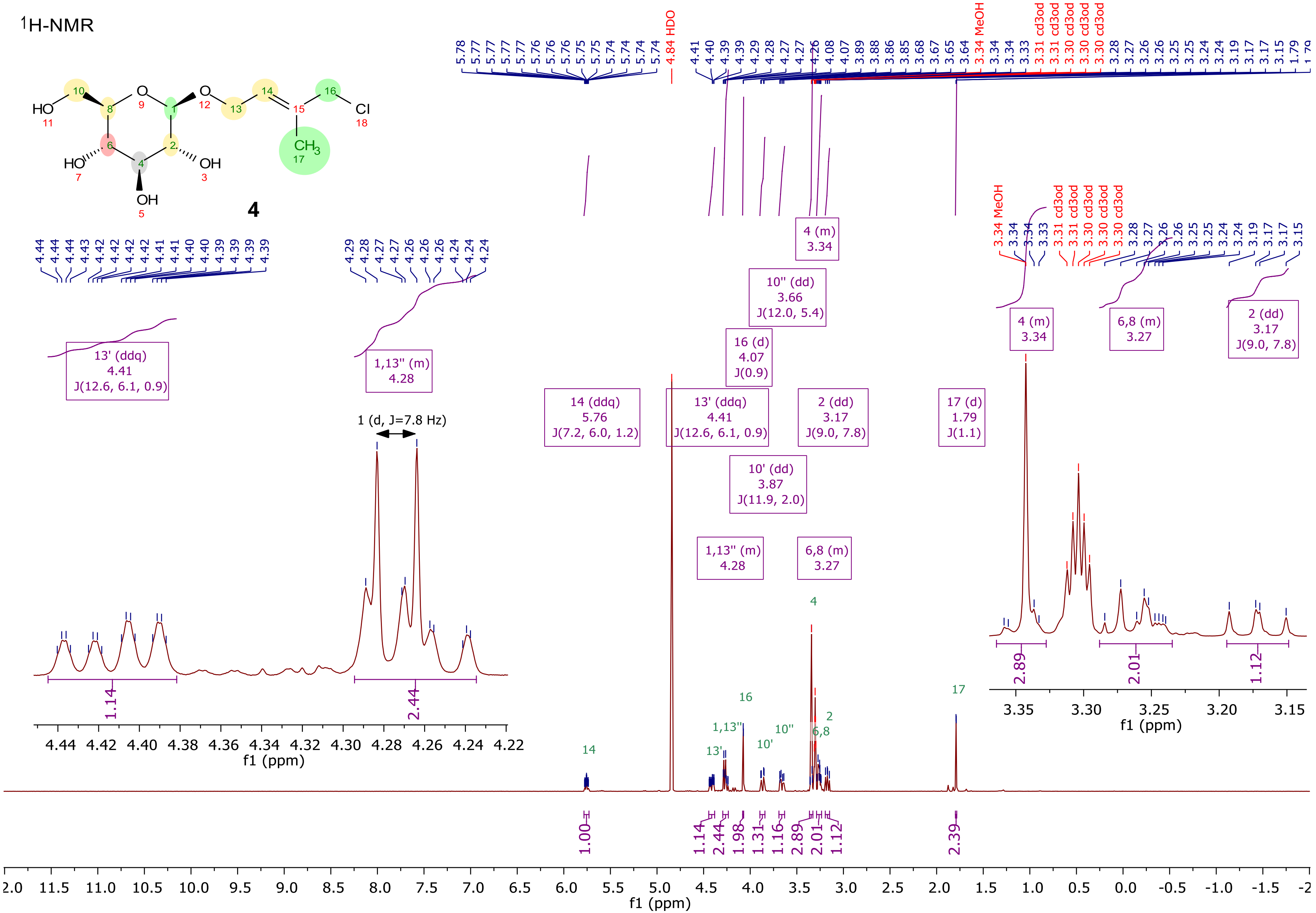

1H-NMR
HO
11
10
8
O
9
1
O
12
13
14
15
16
Cl
18
CH3
17
HO
7
6
4
2
OH
3
OH
5
4
4 (m)
3.34
10'' (dd)
3.66
J(12.0, 5.4)
16 (d)
4.07
J(0.9)
14 (ddq)
5.76
J(7.2, 6.0, 1.2)
13' (ddq)
4.41
J(12.6, 6.1, 0.9)
2 (dd)
3.17
J(9.0, 7.8)
17 (d)
1.79
J(1.1)
10' (dd)
3.87
J(11.9, 2.0)
1,13'' (m)
4.28
6,8 (m)
3.27
1 (d, J=7.8 Hz)
4.84 HDO
3.34 MeOH
3.31 cd3od
3.30 cd3od
1.14
2.44
1.00
1.98
1.31
1.16
2.89
2.01
1.12
2.39
f1 (ppm)

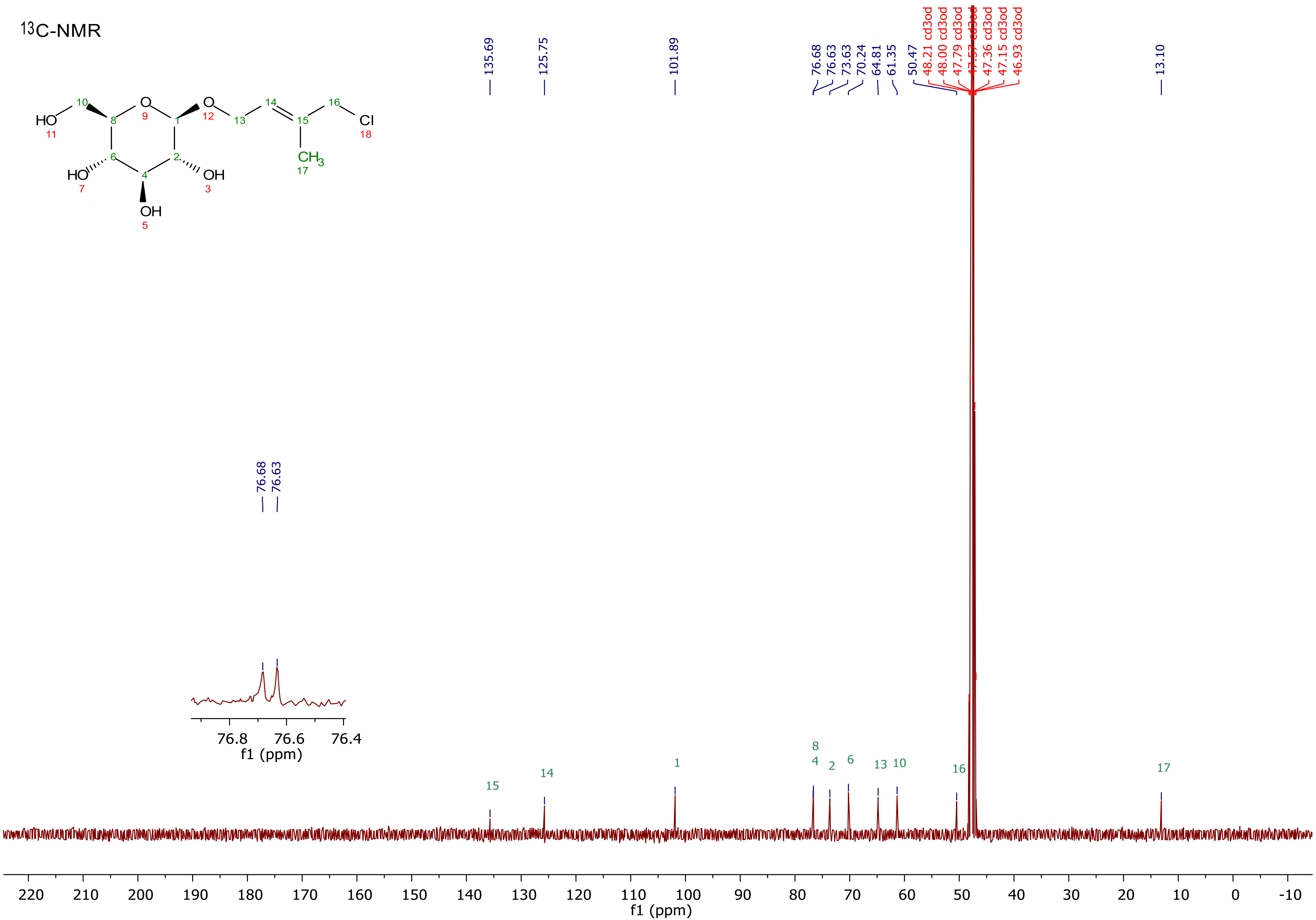
13C-NMR
135.69
125.75
101.89
76.68
76.63
73.63
70.24
64.81
61.35
50.47
48.21 cd3od
48.00 cd3od
47.79 cd3od
47.36 cd3od
47.15 cd3od
46.93 cd3od
13.10
76.8
76.6
76.4
f1 (ppm)
f1 (ppm)

^{1}H-NMR

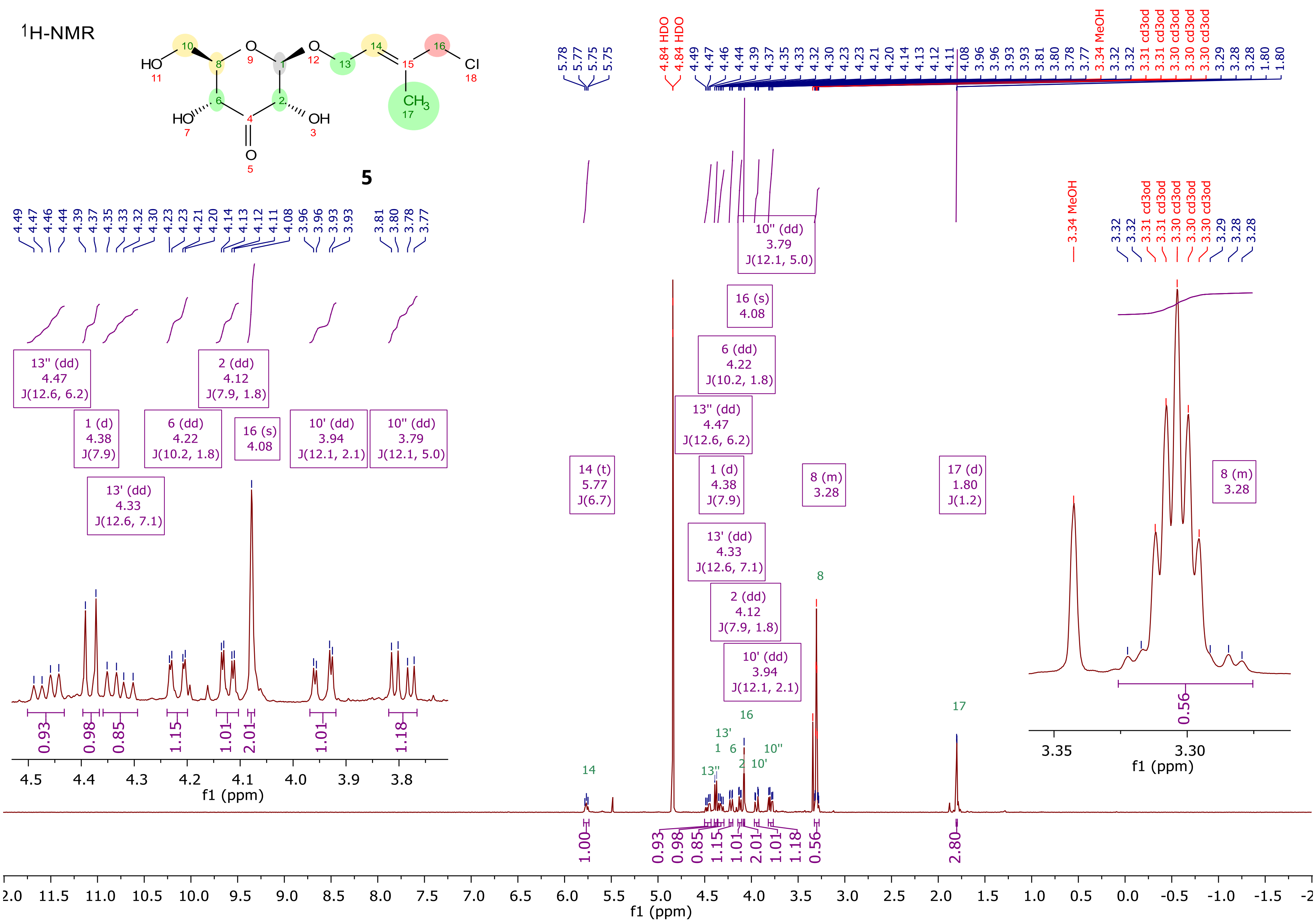

5

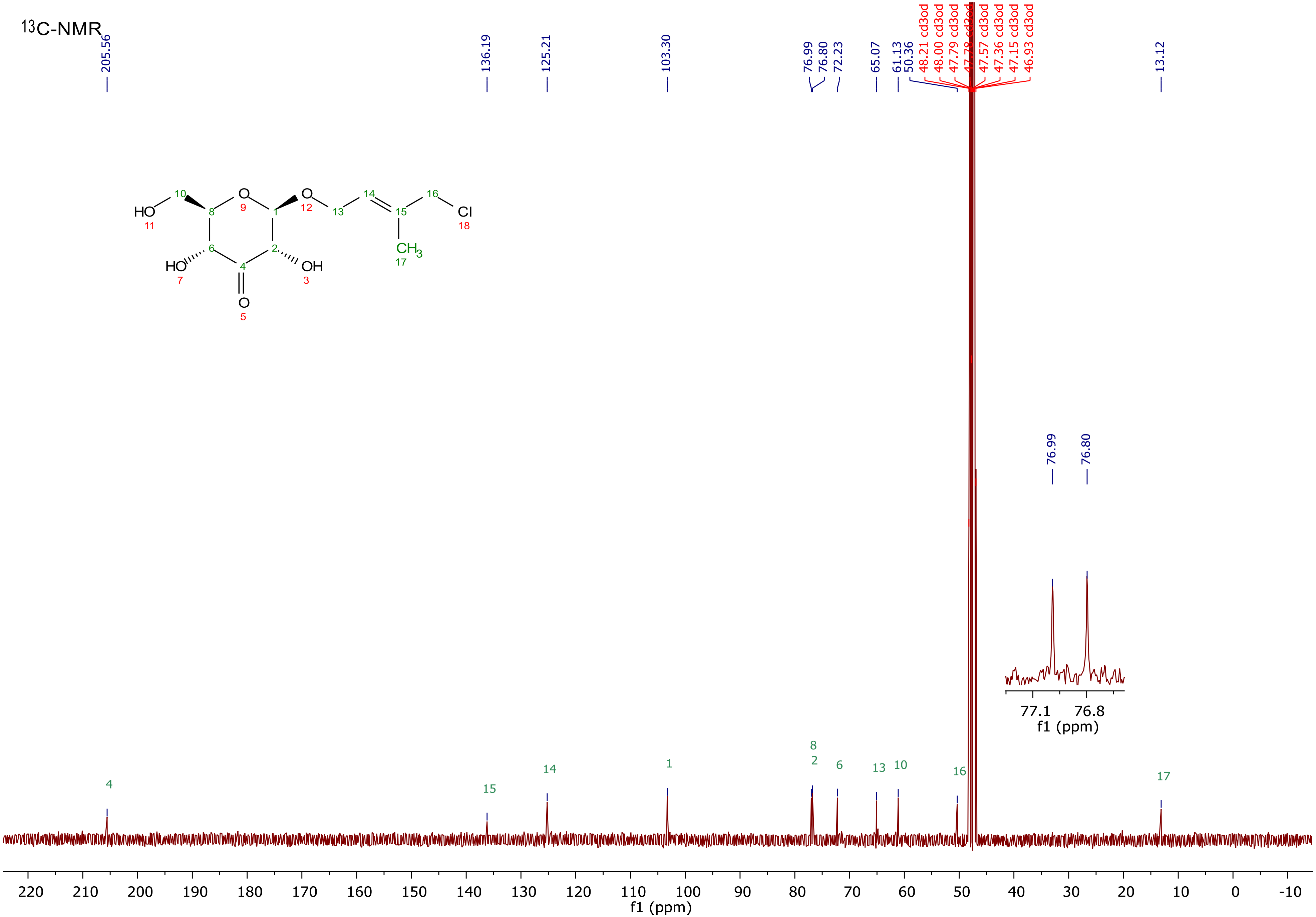

13C-NMR
205.56
136.19
125.21
103.30
76.99
76.80
72.23
65.07
61.13
50.36
48.21 cd3od
48.00 cd3od
47.79 cd3od
47.78 cd3od
47.57 cd3od
47.36 cd3od
47.15 cd3od
46.93 cd3od
13.12
HO
11
10
8
O
9
1
O
12
13
14
15
16
Cl
18
CH3
17
HO
7
6
4
2
OH
3
O
5
77.1
76.8
f1 (ppm)
8
2
6
13
10
16
17
4
15
14
1
220
210
200
190
180
170
160
150
140
130
120
110
100
90
80
70
60
50
40
30
20
10
0
-10
f1 (ppm)

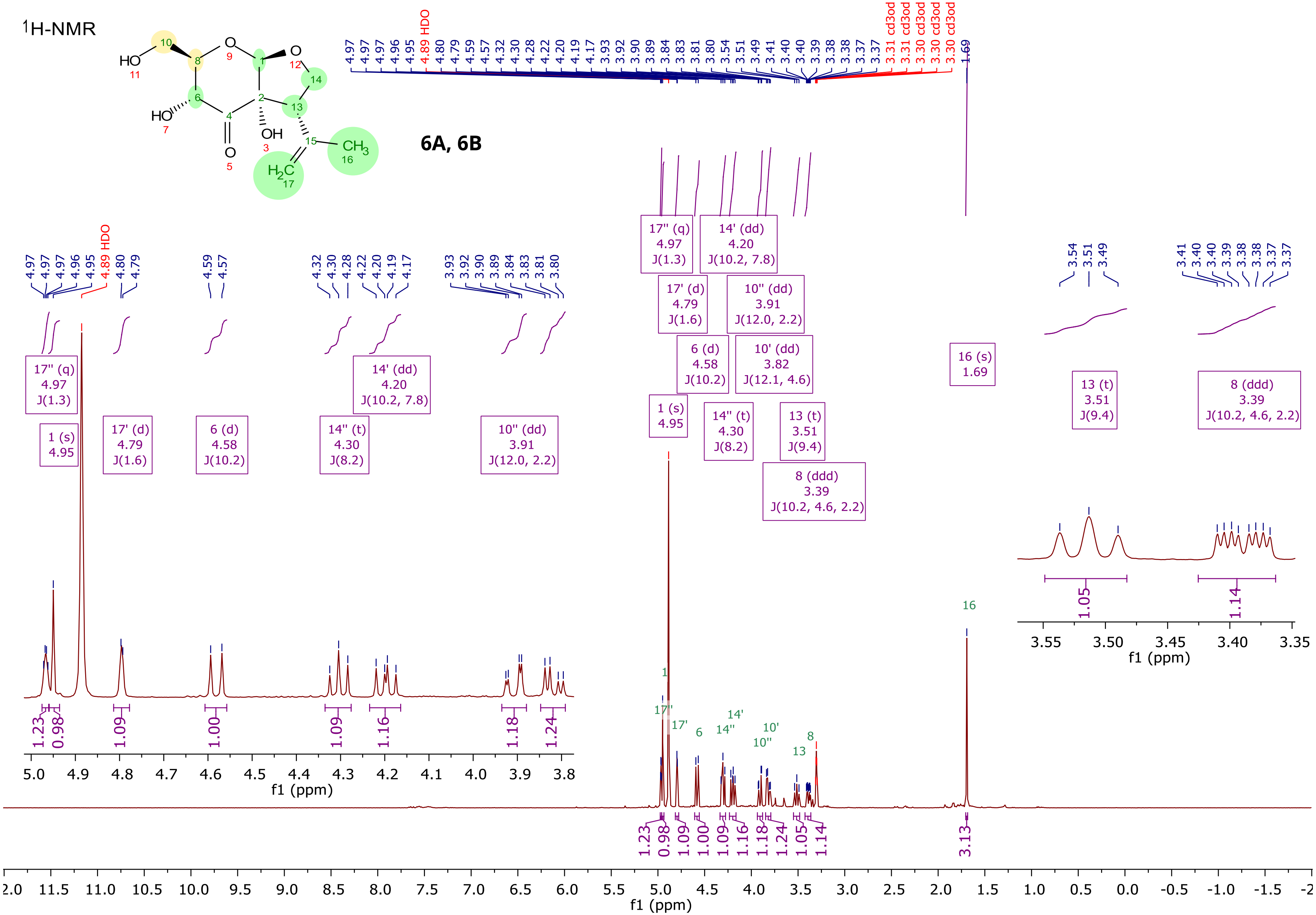

1H-NMR
6A, 6B
17'' (q) 4.97 J(1.3)
14' (dd) 4.20 J(10.2, 7.8)
17' (d) 4.79 J(1.6)
10'' (dd) 3.91 J(12.0, 2.2)
6 (d) 4.58 J(10.2)
10' (dd) 3.82 J(12.1, 4.6)
1 (s) 4.95
14'' (t) 4.30 J(8.2)
13 (t) 3.51 J(9.4)
8 (ddd) 3.39 J(10.2, 4.6, 2.2)
16 (s) 1.69
4.89 HDO
3.31 cd3od
f1 (ppm)

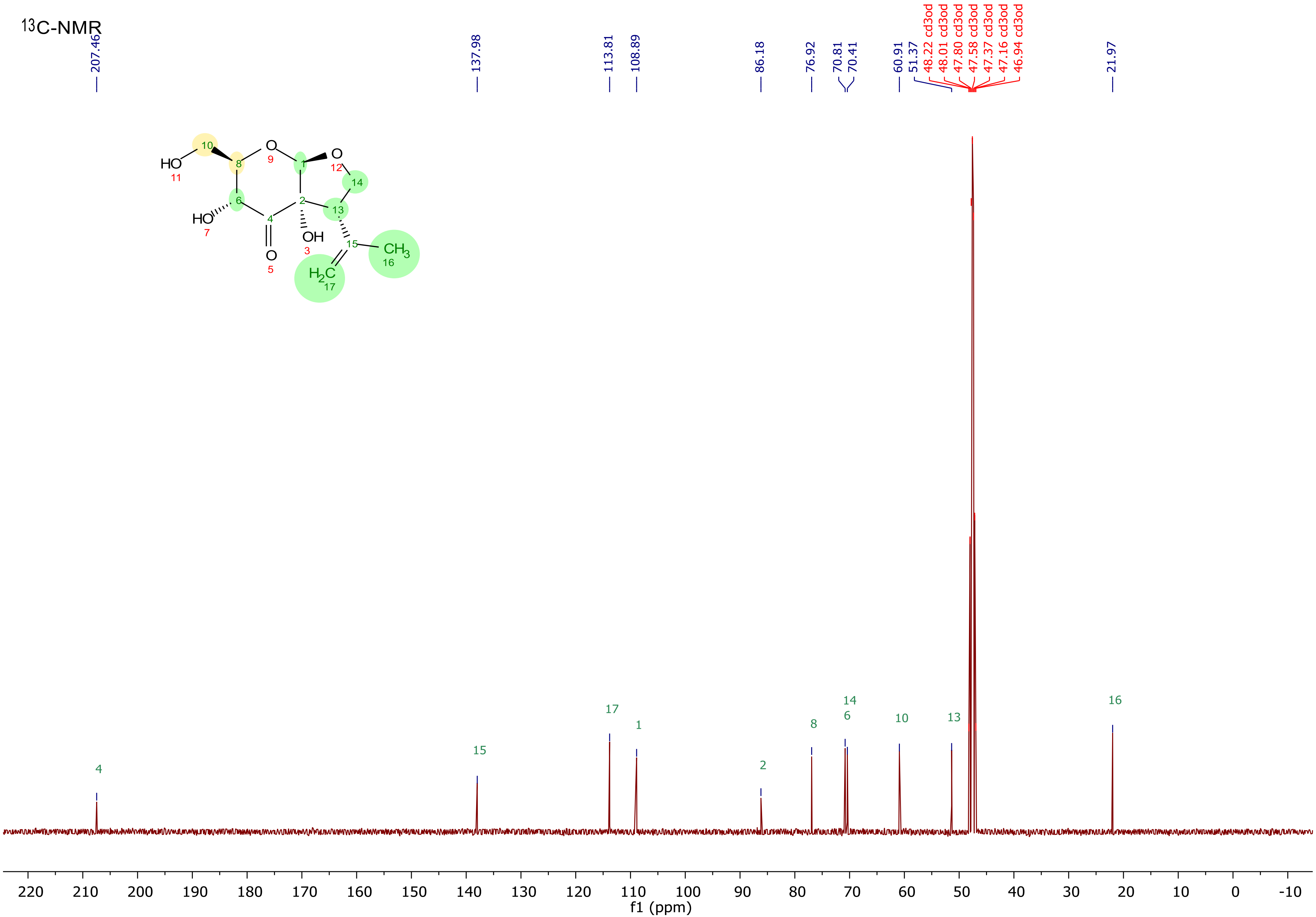
13C-NMR
207.46
137.98
113.81
108.89
86.18
76.92
70.81
70.41
60.91
51.37
48.22 cd3od
48.01 cd3od
47.80 cd3od
47.58 cd3od
47.37 cd3od
47.16 cd3od
46.94 cd3od
21.97
f1 (ppm)

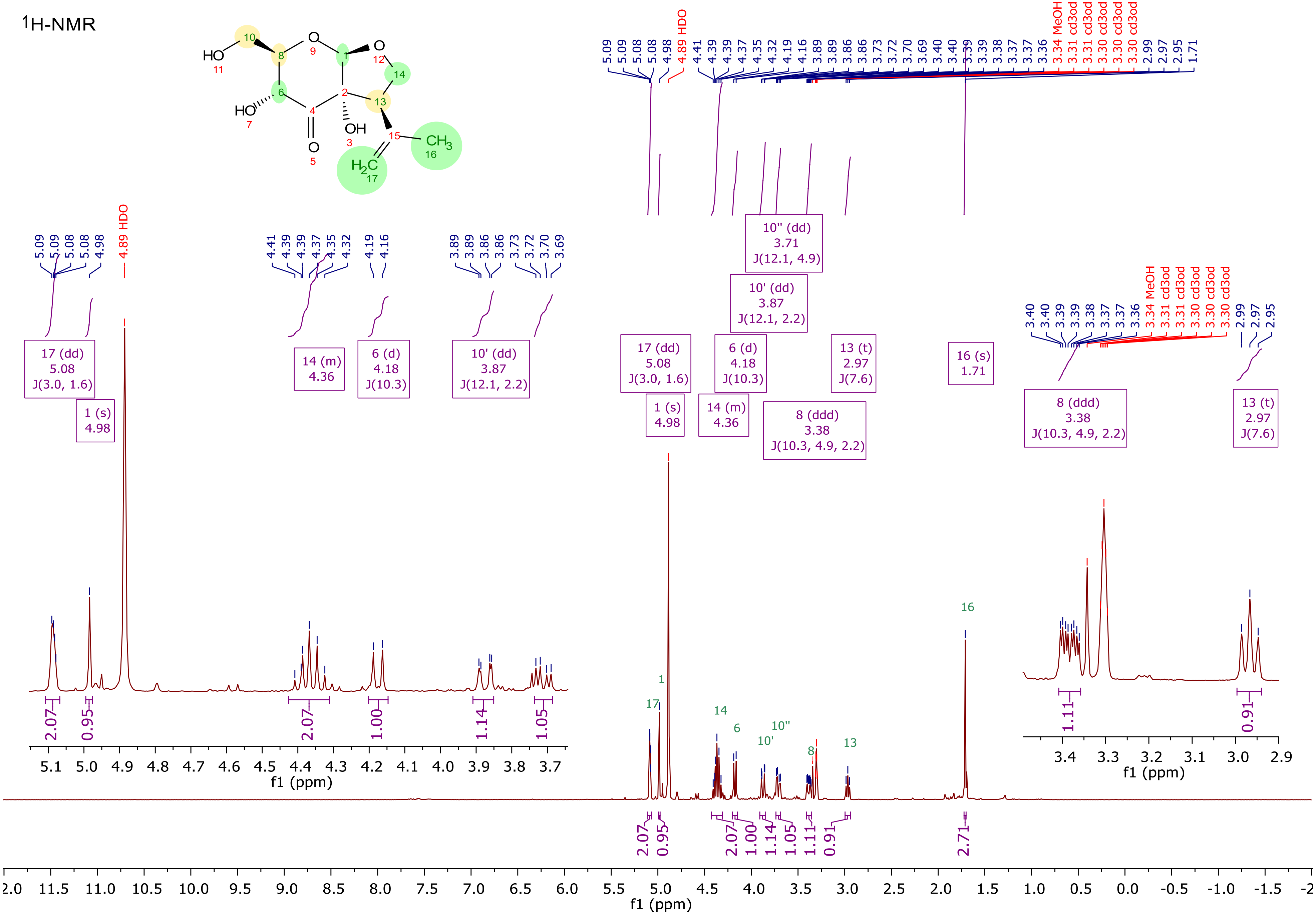

1H-NMR
17 (dd) 5.08 J(3.0, 1.6)
1 (s) 4.98
4.89 HDO
14 (m) 4.36
6 (d) 4.18 J(10.3)
10' (dd) 3.87 J(12.1, 2.2)
10'' (dd) 3.71 J(12.1, 4.9)
8 (ddd) 3.38 J(10.3, 4.9, 2.2)
13 (t) 2.97 J(7.6)
16 (s) 1.71
3.34 MeOH
3.31 cd3od
f1 (ppm)

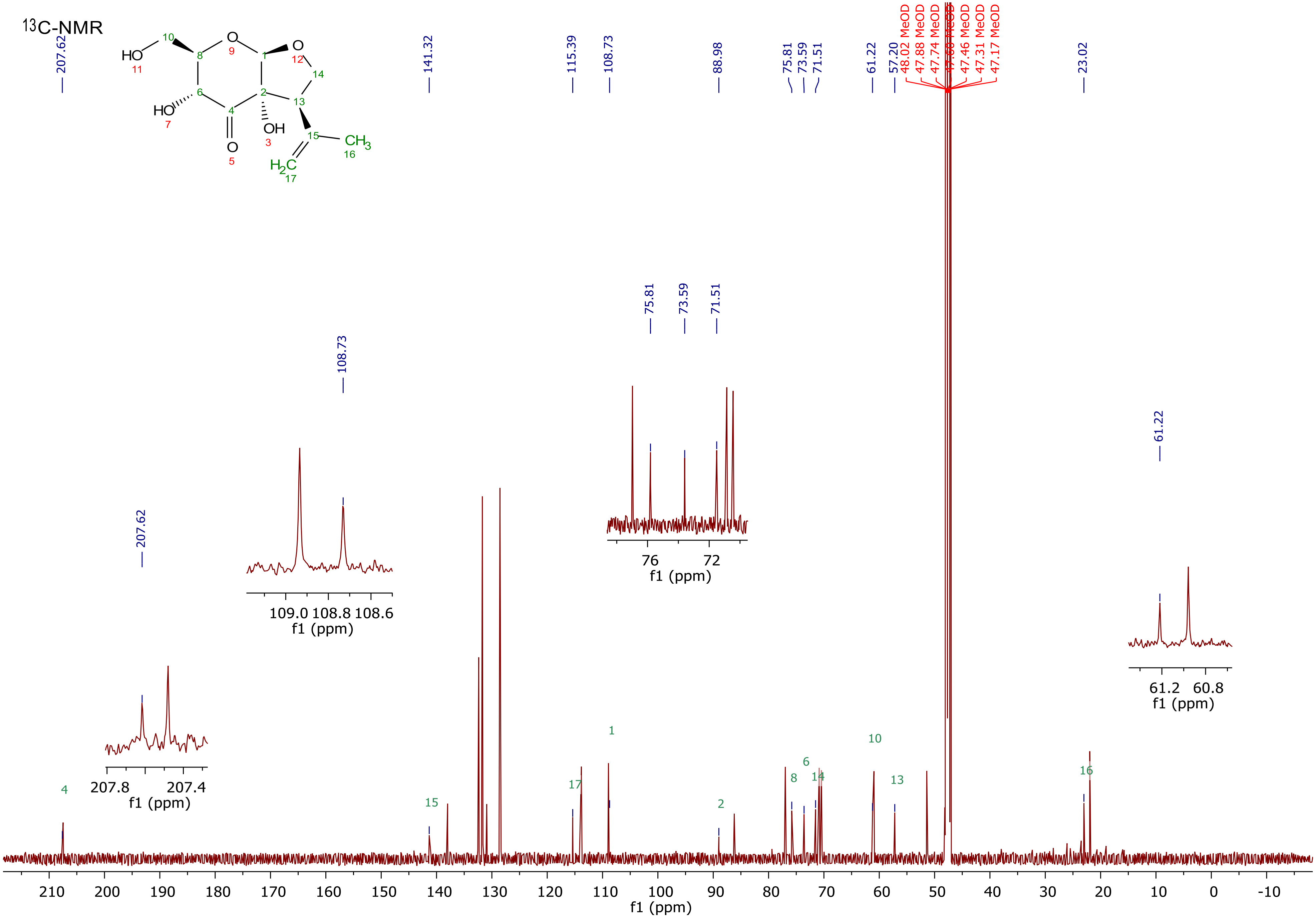

13C-NMR
207.62
141.32
115.39
108.73
88.98
75.81
73.59
71.51
61.22
57.20
48.02 MeOD
47.88 MeOD
47.74 MeOD
47.46 MeOD
47.31 MeOD
47.17 MeOD
23.02
HO
OH
CH3
H2C
f1 (ppm)

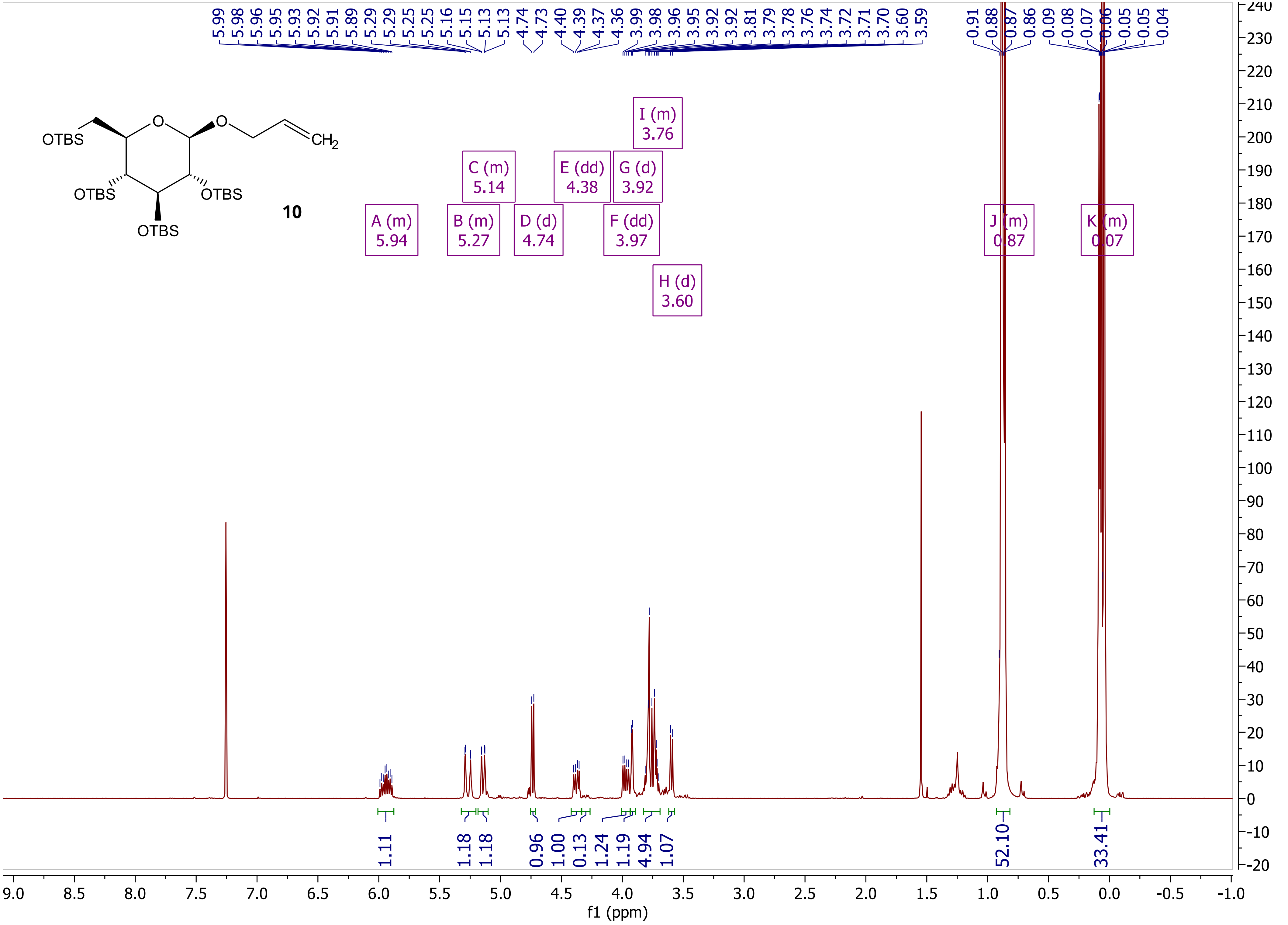

OTBS
O
O
CH2
OTBS
OTBS
OTBS
10
5.99 5.98 5.96 5.95 5.93 5.92 5.91 5.89 5.29 5.29 5.25 5.25 5.16 5.15 5.13 5.13 4.74 4.73 4.40 4.39 4.37 4.36 3.99 3.98 3.96 3.95 3.92 3.92 3.81 3.79 3.78 3.76 3.74 3.72 3.71 3.70 3.60 3.59
0.91 0.88 0.87 0.87 0.86 0.09 0.08 0.07 0.06 0.05 0.05 0.04
A (m) 5.94
B (m) 5.27
C (m) 5.14
D (d) 4.74
E (dd) 4.38
F (dd) 3.97
G (d) 3.92
H (d) 3.60
I (m) 3.76
J (m) 0.87
K (m) 0.07
1.11
1.18
1.18
0.96
1.00
0.13
1.24
1.19
4.94
1.07
52.10
33.41
f1 (ppm)

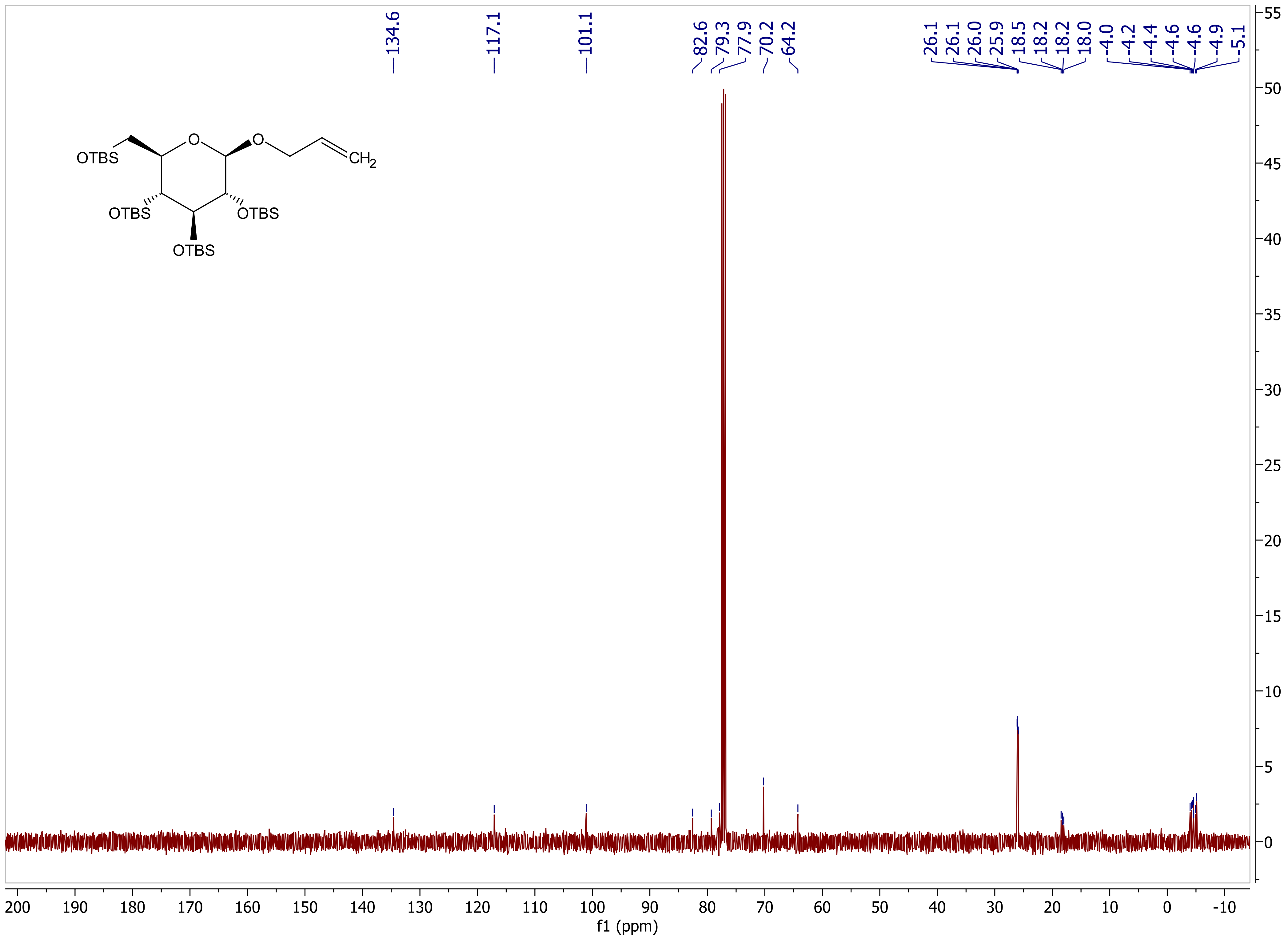

134.6
117.1
101.1
82.6
79.3
77.9
70.2
64.2
26.1
26.1
26.0
25.9
18.5
18.2
18.2
18.0
-4.0
-4.2
-4.4
-4.6
-4.6
-4.9
-5.1
OTBS
O
O
CH2
OTBS
OTBS
OTBS
f1 (ppm)

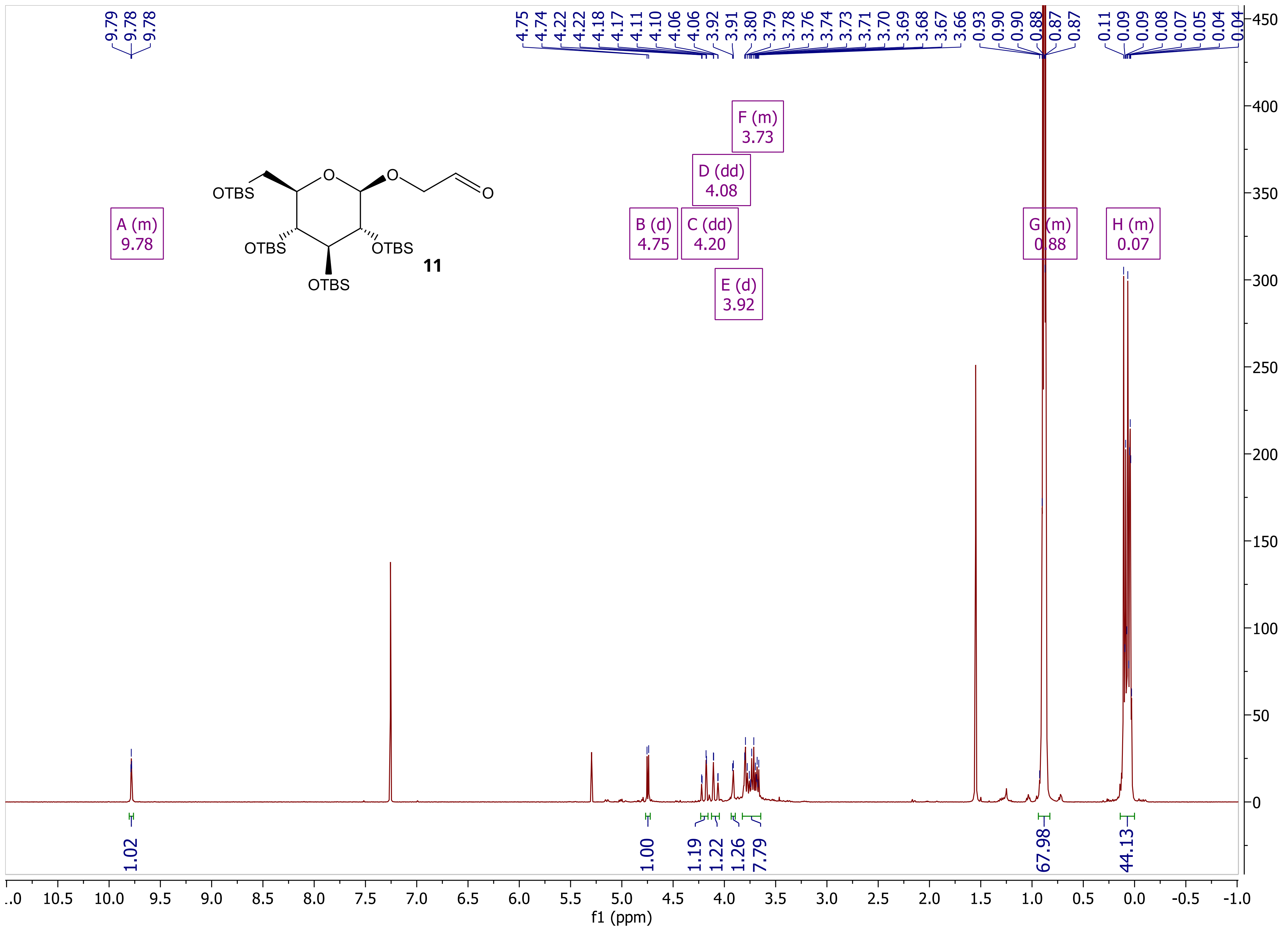

9.79
9.78
9.78
4.75
4.74
4.22
4.22
4.18
4.17
4.11
4.10
4.06
4.06
3.92
3.91
3.80
3.79
3.78
3.76
3.74
3.73
3.71
3.70
3.69
3.68
3.67
3.66
0.93
0.90
0.90
0.88
0.87
0.87
0.11
0.09
0.09
0.08
0.07
0.05
0.04
0.04
OTBS
O
O
O
OTBS
OTBS
OTBS
11
A (m) 9.78
B (d) 4.75
C (dd) 4.20
D (dd) 4.08
E (d) 3.92
F (m) 3.73
G (m) 0.88
H (m) 0.07
1.02
1.00
1.19
1.22
1.26
7.79
67.98
44.13
f1 (ppm)

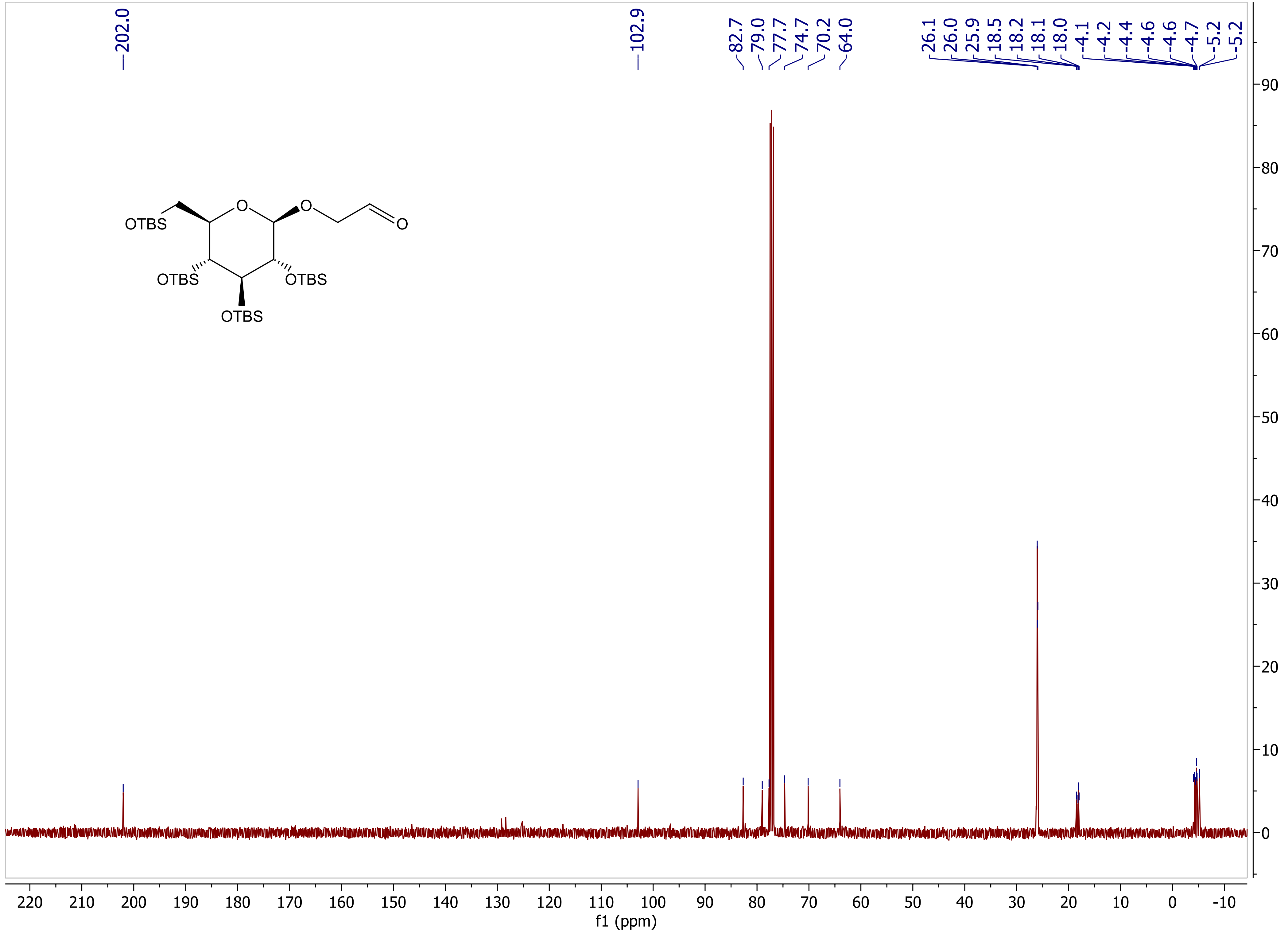

202.0
102.9
82.7
79.0
77.7
74.7
70.2
64.0
26.1
26.0
25.9
18.5
18.2
18.1
18.0
-4.1
-4.2
-4.4
-4.6
-4.6
-4.7
-5.2
-5.2
OTBS
O
O
O
OTBS
OTBS
OTBS
f1 (ppm)

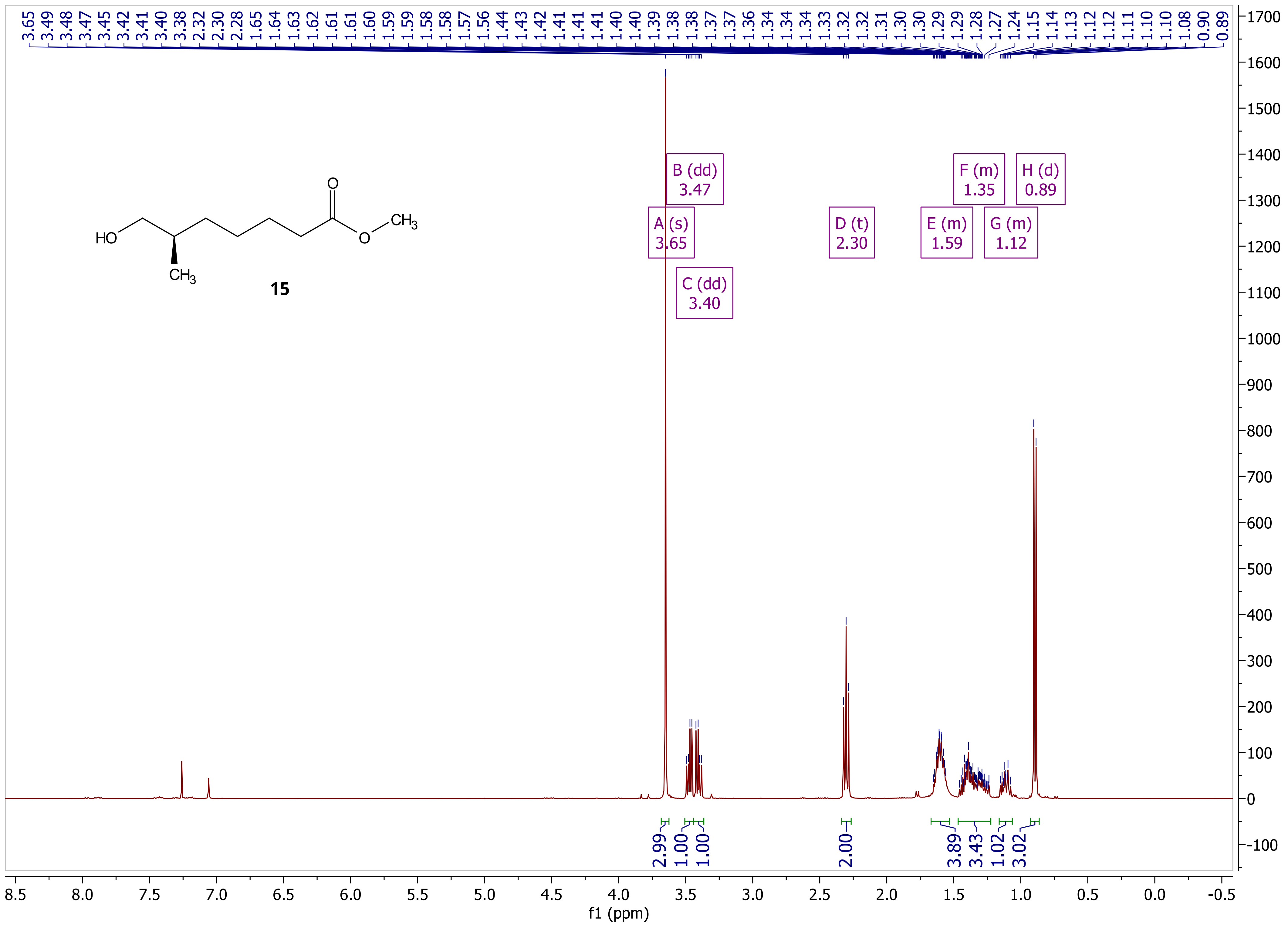

3.65 3.49 3.48 3.47 3.45 3.42 3.41 3.40 3.38 2.32 2.30 2.28 1.65 1.64 1.63 1.62 1.61 1.61 1.60 1.59 1.59 1.58 1.58 1.57 1.56 1.44 1.43 1.42 1.41 1.41 1.41 1.40 1.40 1.39 1.38 1.38 1.37 1.37 1.36 1.34 1.34 1.34 1.33 1.32 1.32 1.31 1.30 1.30 1.29 1.29 1.28 1.27 1.24 1.15 1.14 1.13 1.12 1.12 1.11 1.10 1.10 1.08 0.90 0.89
HO
O
O
CH3
CH3
15
A (s) 3.65
B (dd) 3.47
C (dd) 3.40
D (t) 2.30
E (m) 1.59
F (m) 1.35
G (m) 1.12
H (d) 0.89
2.99 1.00 1.00 2.00 3.89 3.43 1.02 3.02
f1 (ppm)

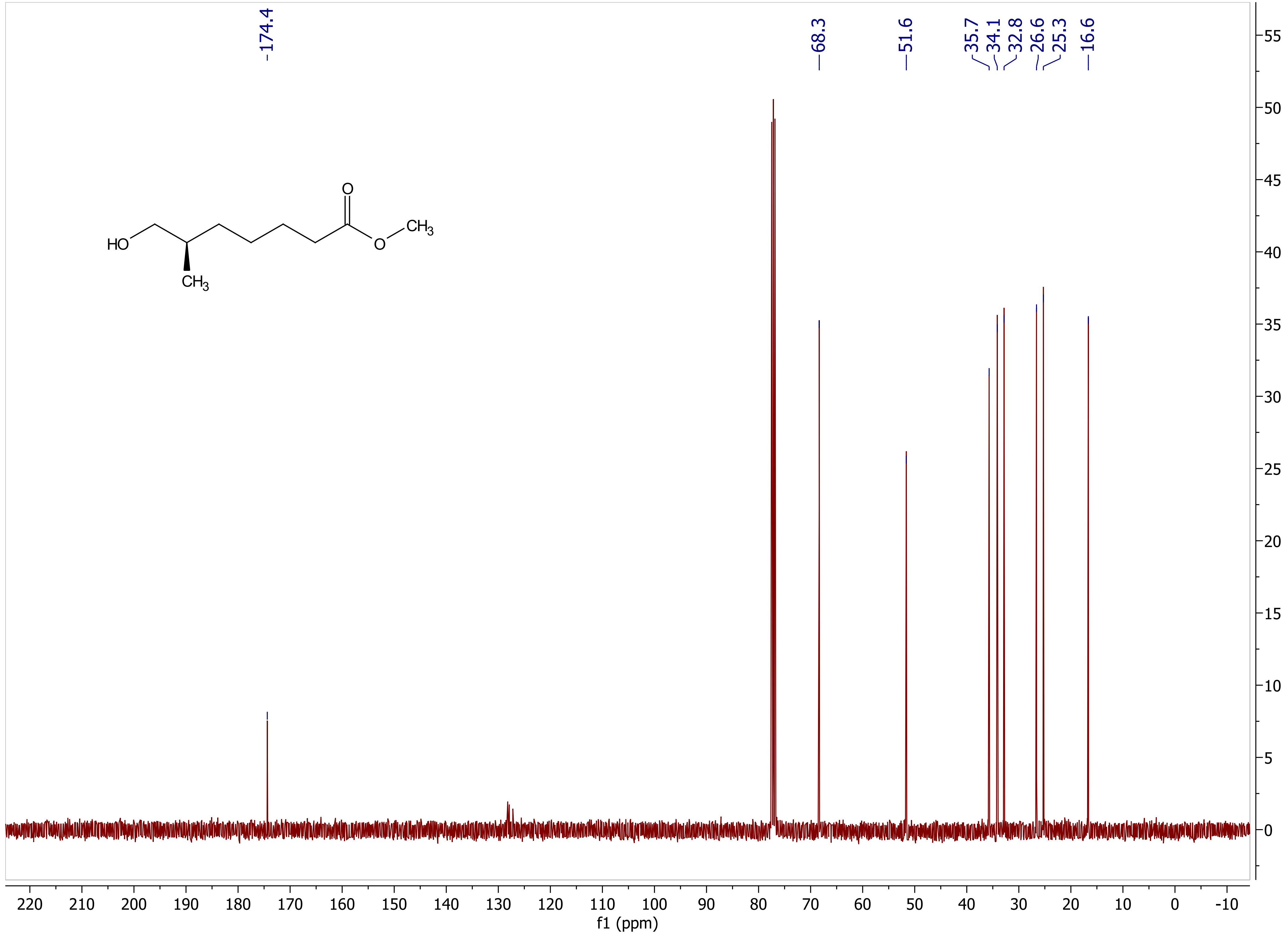

174.4
68.3
51.6
35.7
34.1
32.8
26.6
25.3
16.6
HO
O
O
CH3
CH3
f1 (ppm)

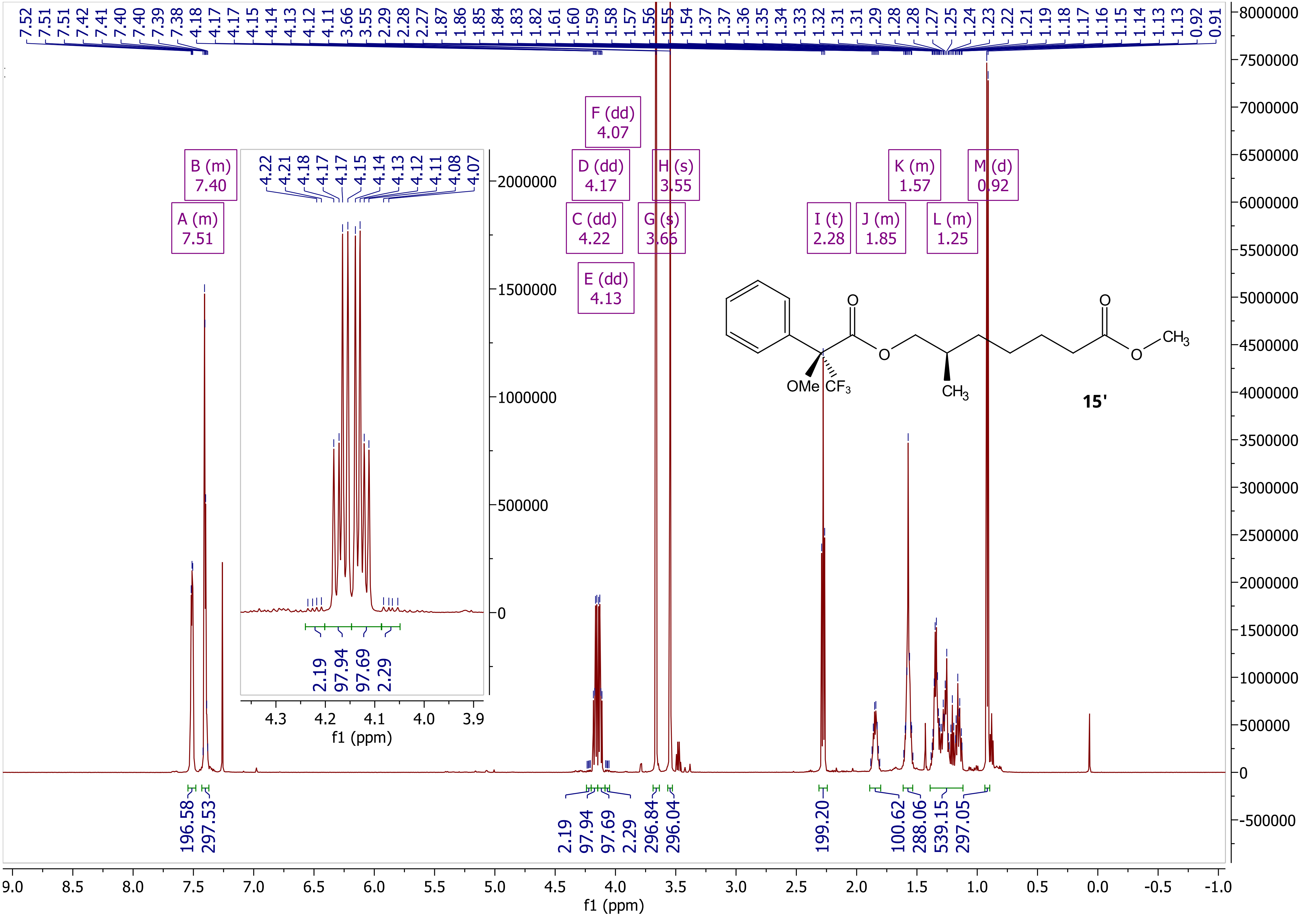

A (m)
7.51
B (m)
7.40
C (dd)
4.22
D (dd)
4.17
E (dd)
4.13
F (dd)
4.07
G (s)
3.66
H (s)
3.55
I (t)
2.28
J (m)
1.85
K (m)
1.57
L (m)
1.25
M (d)
0.92
OMe
CF3
CH3
15'
f1 (ppm)

13C NMR of Methyl (R)-7-hydroxy-6-methylheptanoate (R)-Mosher's ester derivative (39')

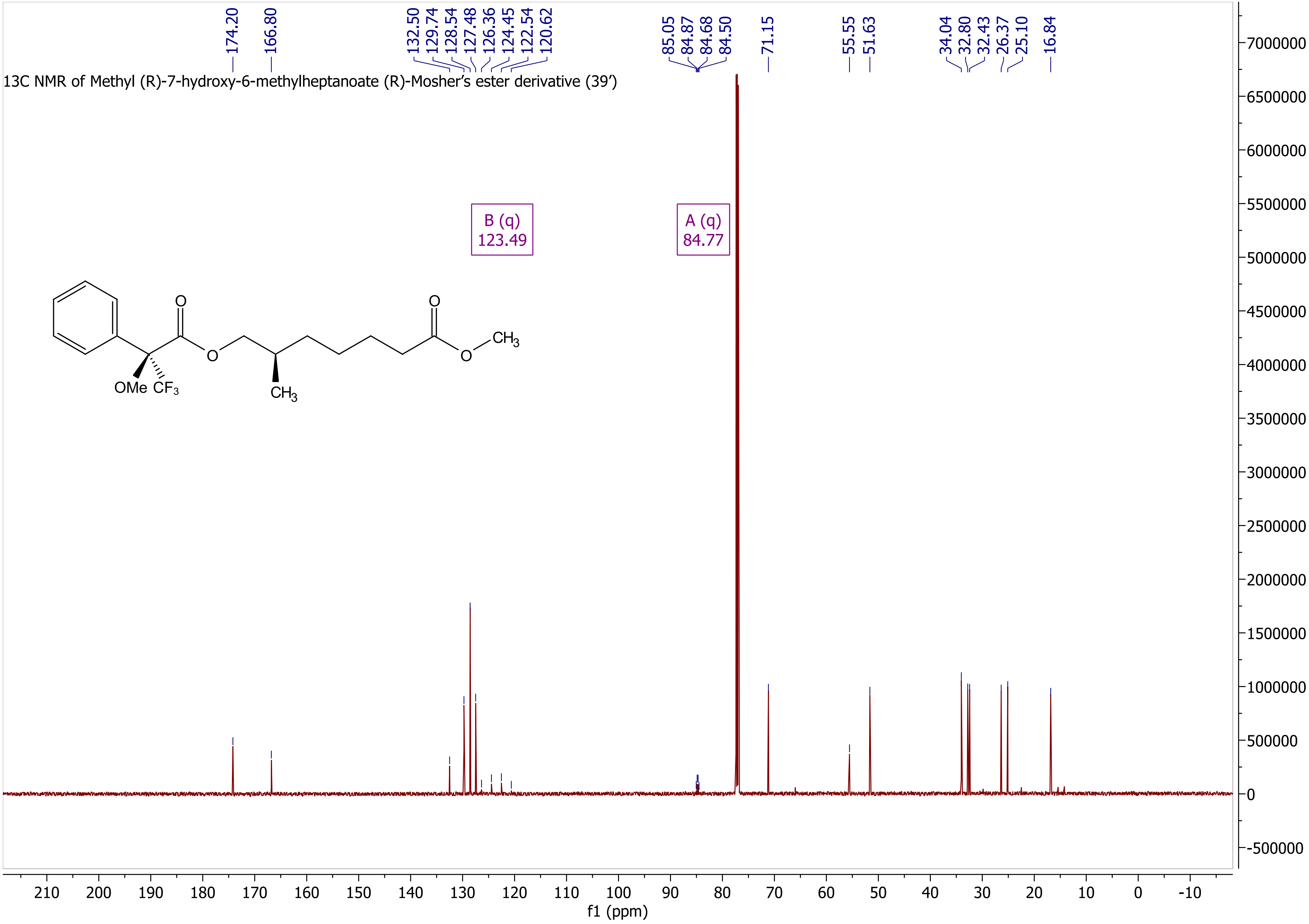

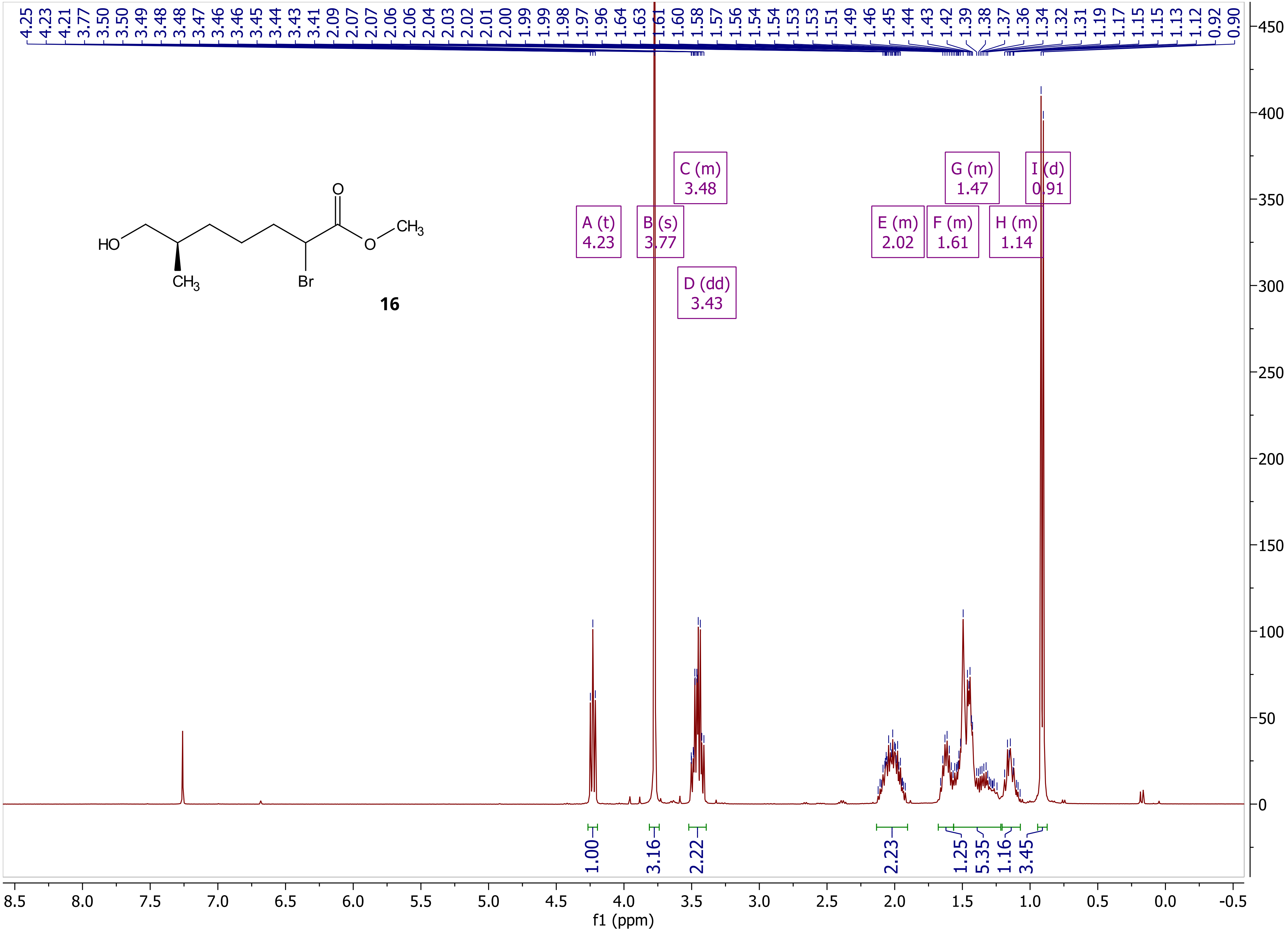

4.25
4.23
4.21
3.77
3.50
3.50
3.49
3.48
3.48
3.47
3.46
3.46
3.45
3.44
3.43
3.41
2.09
2.07
2.07
2.06
2.06
2.04
2.03
2.02
2.01
2.00
1.99
1.99
1.98
1.97
1.96
1.64
1.63
1.61
1.60
1.58
1.57
1.56
1.54
1.54
1.53
1.53
1.51
1.49
1.46
1.45
1.44
1.43
1.42
1.39
1.38
1.37
1.36
1.34
1.32
1.31
1.19
1.17
1.15
1.15
1.13
1.12
0.92
0.90
HO
CH3
Br
O
O
CH3
16
A (t)
4.23
B (s)
3.77
C (m)
3.48
D (dd)
3.43
E (m)
2.02
F (m)
1.61
G (m)
1.47
H (m)
1.14
I (d)
0.91
1.00
3.16
2.22
2.23
1.25
5.35
1.16
3.45
f1 (ppm)

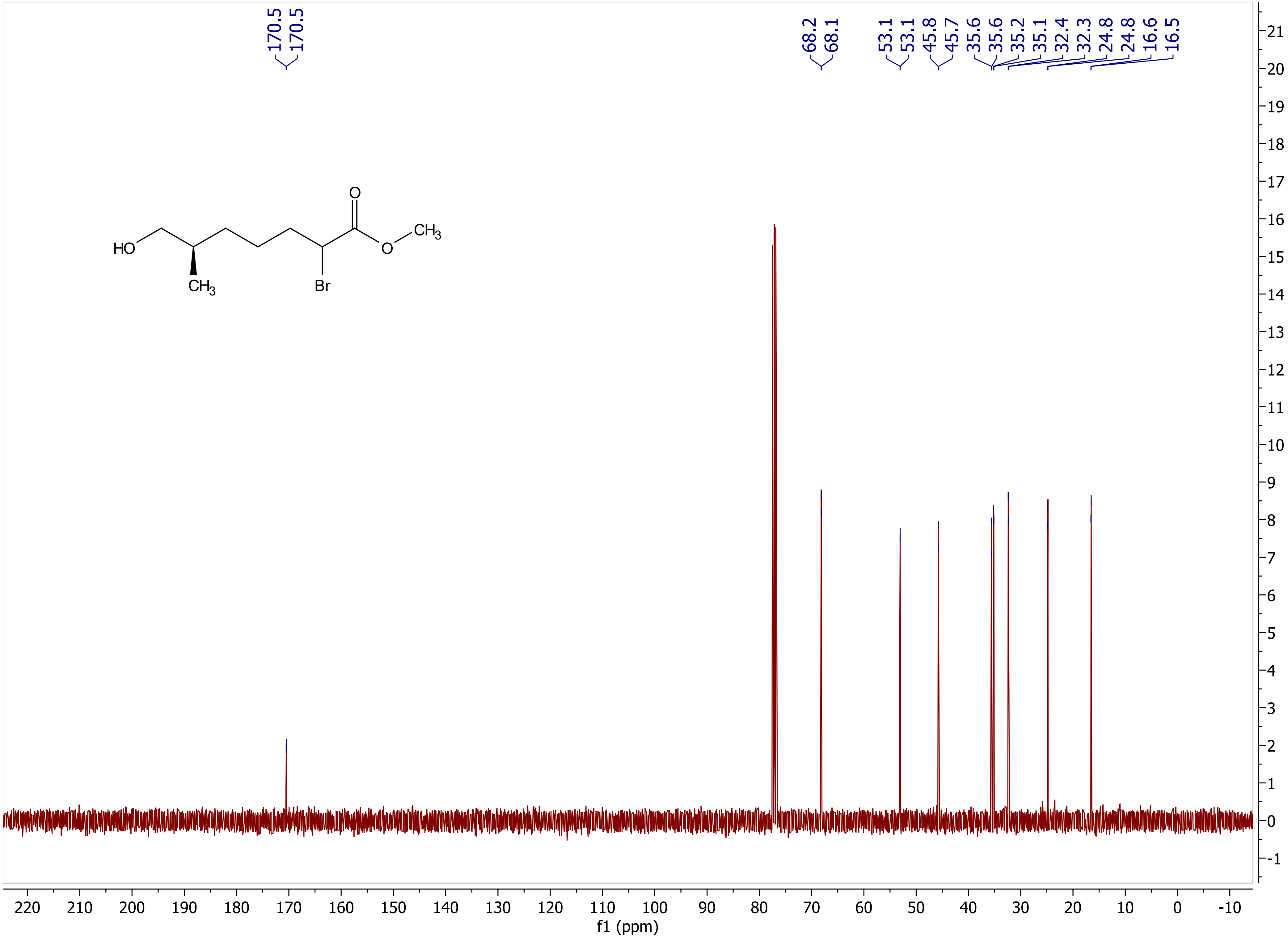
170.5
170.5
68.2
68.1
53.1
53.1
45.8
45.7
35.6
35.6
35.2
35.1
32.4
32.3
24.8
24.8
16.6
16.5
HO
CH3
Br
O
O
CH3
f1 (ppm)

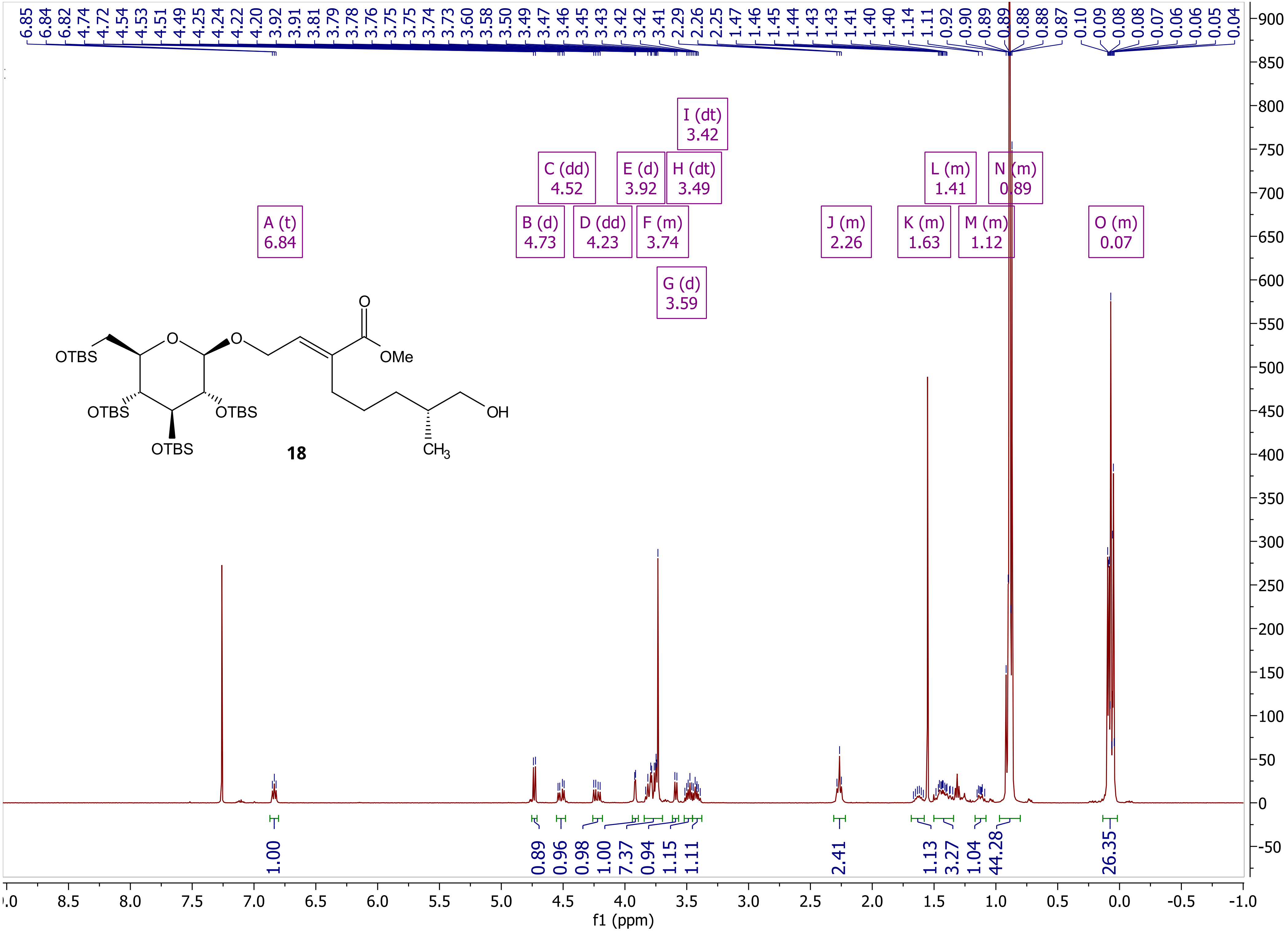

I (dt) 3.42
C (dd) 4.52
E (d) 3.92
H (dt) 3.49
L (m) 1.41
N (m) 0.89
A (t) 6.84
B (d) 4.73
D (dd) 4.23
F (m) 3.74
J (m) 2.26
K (m) 1.63
M (m) 1.12
O (m) 0.07
G (d) 3.59
OTBS
OMe
OH
CH3
18
1.00
0.89
0.96
0.98
1.00
7.37
0.94
1.15
1.11
2.41
1.13
3.27
1.04
44.28
26.35
f1 (ppm)

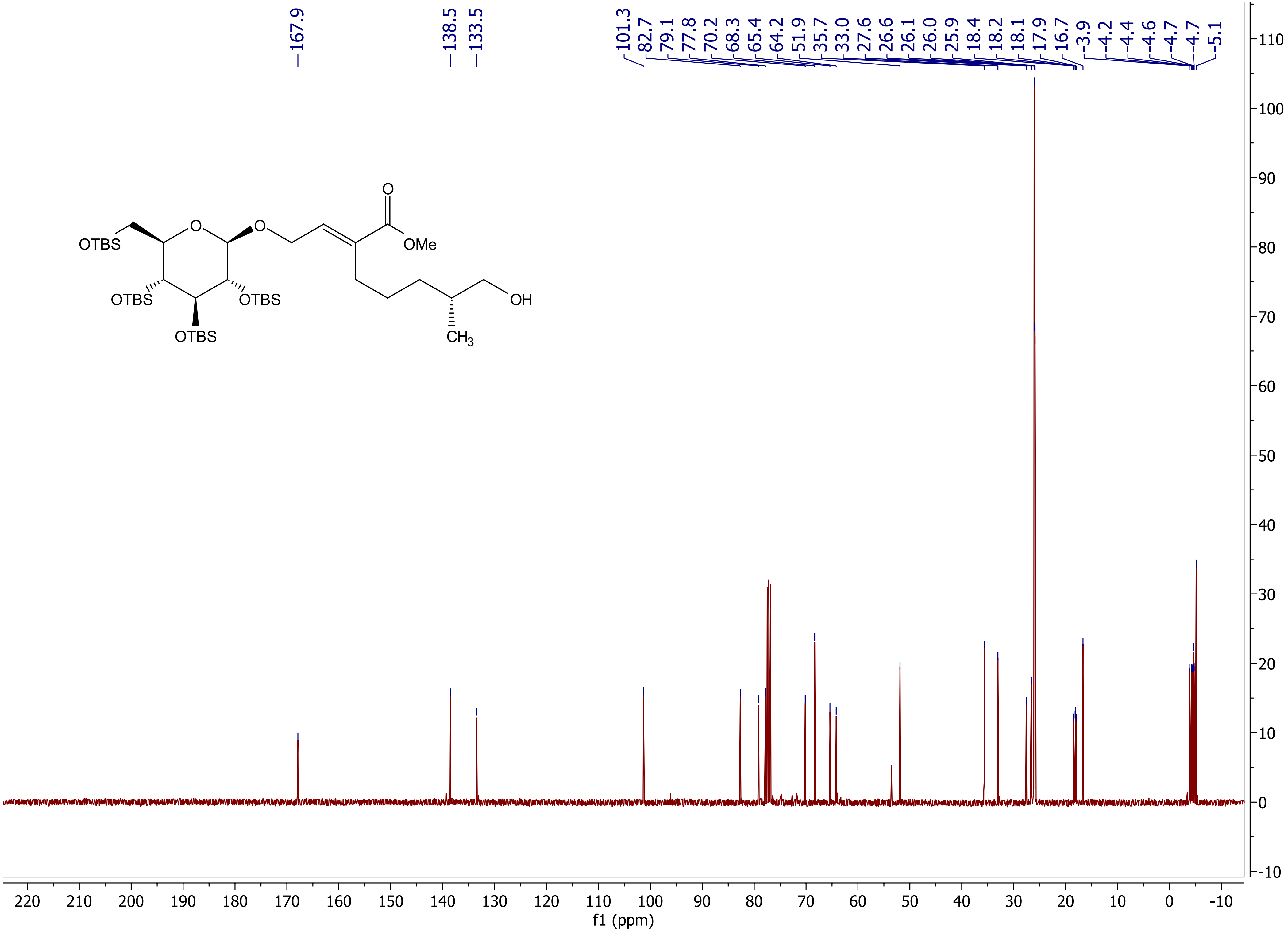

167.9
138.5
133.5
101.3
82.7
79.1
77.8
70.2
68.3
65.4
64.2
51.9
35.7
33.0
27.6
26.6
26.1
26.0
25.9
18.4
18.2
18.1
17.9
16.7
-3.9
-4.2
-4.4
-4.6
-4.7
-4.7
-5.1
OTBS
O
O
O
OMe
OTBS
OTBS
OTBS
OH
CH_3
f1 (ppm)

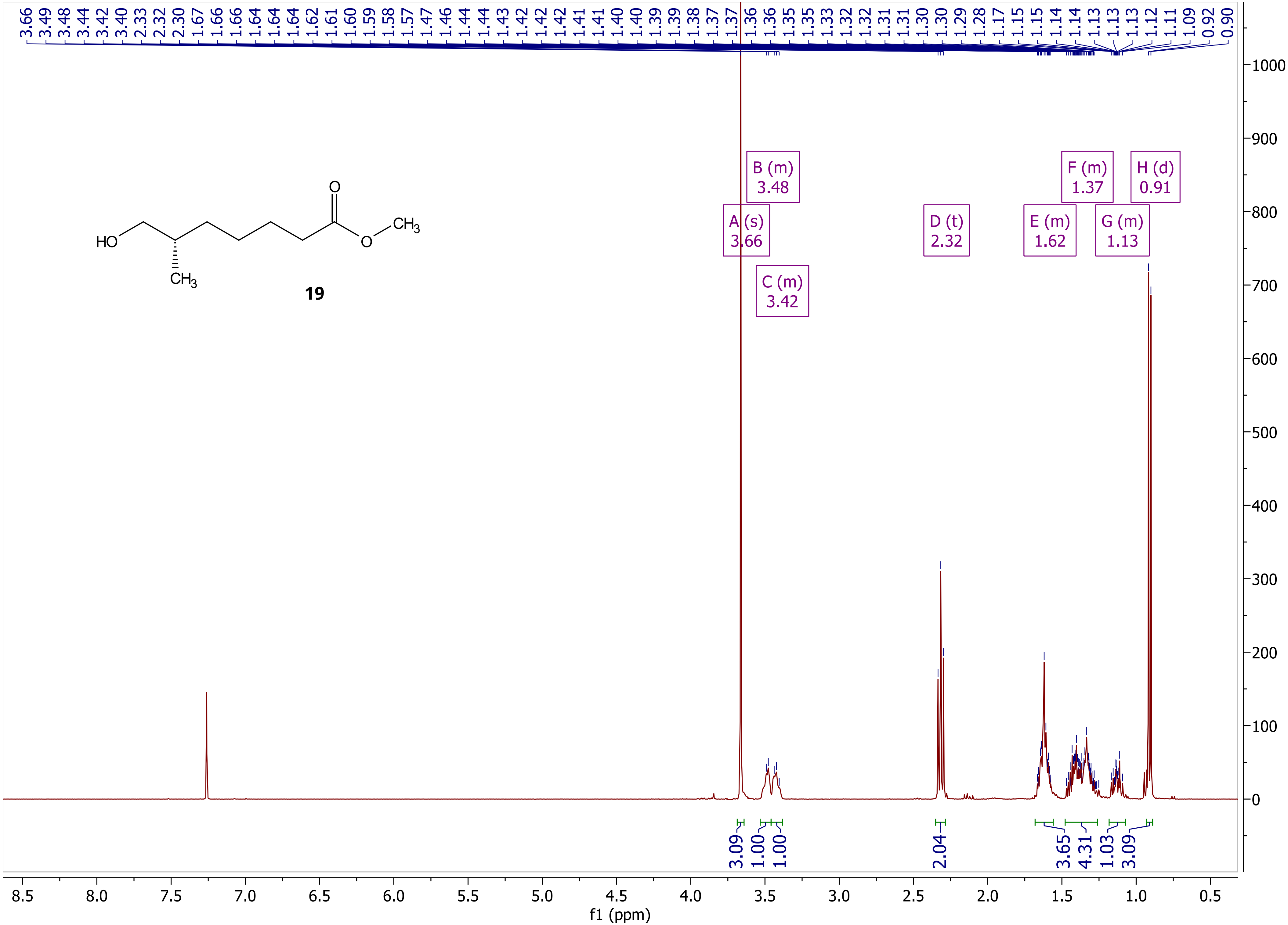

HO
O
O
CH3
CH3
19
A (s) 3.66
B (m) 3.48
C (m) 3.42
D (t) 2.32
E (m) 1.62
F (m) 1.37
G (m) 1.13
H (d) 0.91
3.09
1.00
1.00
2.04
3.65
4.31
1.03
3.09
f1 (ppm)

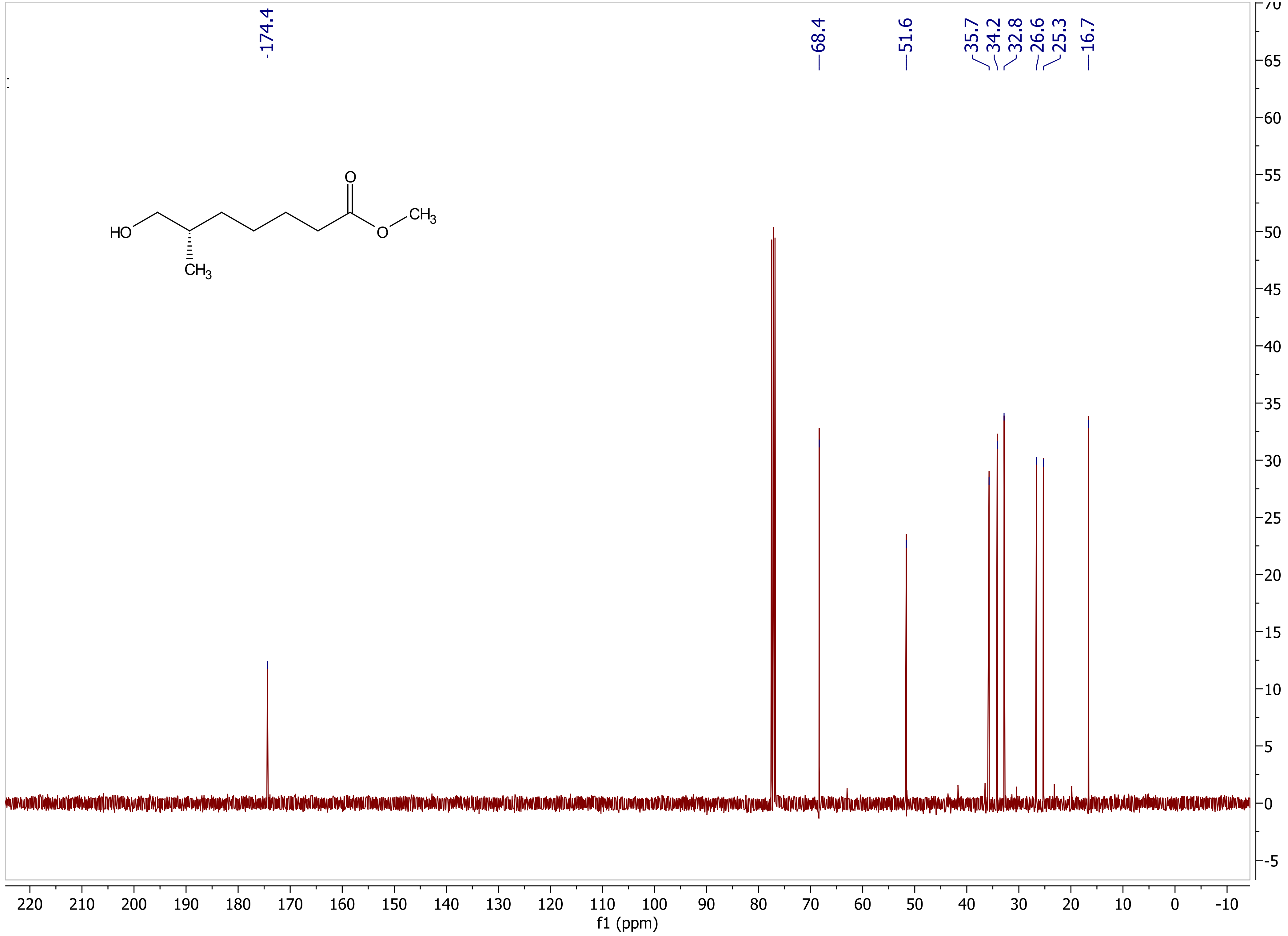

174.4
68.4
51.6
35.7
34.2
32.8
26.6
25.3
16.7
HO
O
CH3
CH3
220 210 200 190 180 170 160 150 140 130 120 110 100 90 80 70 60 50 40 30 20 10 0 -10
f1 (ppm)
70 65 60 55 50 45 40 35 30 25 20 15 10 5 0 -5

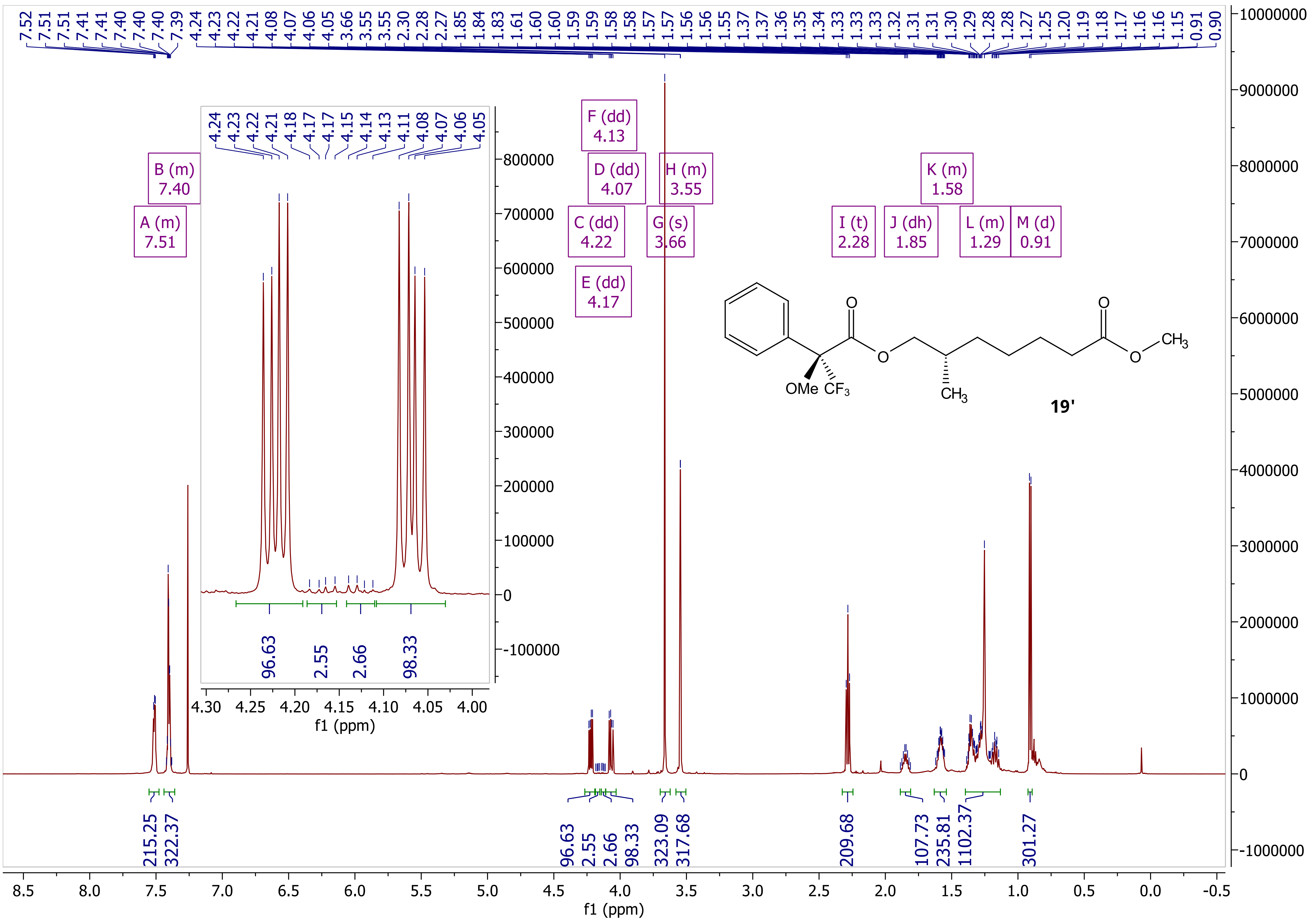

A (m) 7.51
B (m) 7.40
C (dd) 4.22
D (dd) 4.07
E (dd) 4.17
F (dd) 4.13
G (s) 3.66
H (m) 3.55
I (t) 2.28
J (dh) 1.85
K (m) 1.58
L (m) 1.29
M (d) 0.91
O
OMe CF3
CH3
19'
f1 (ppm)

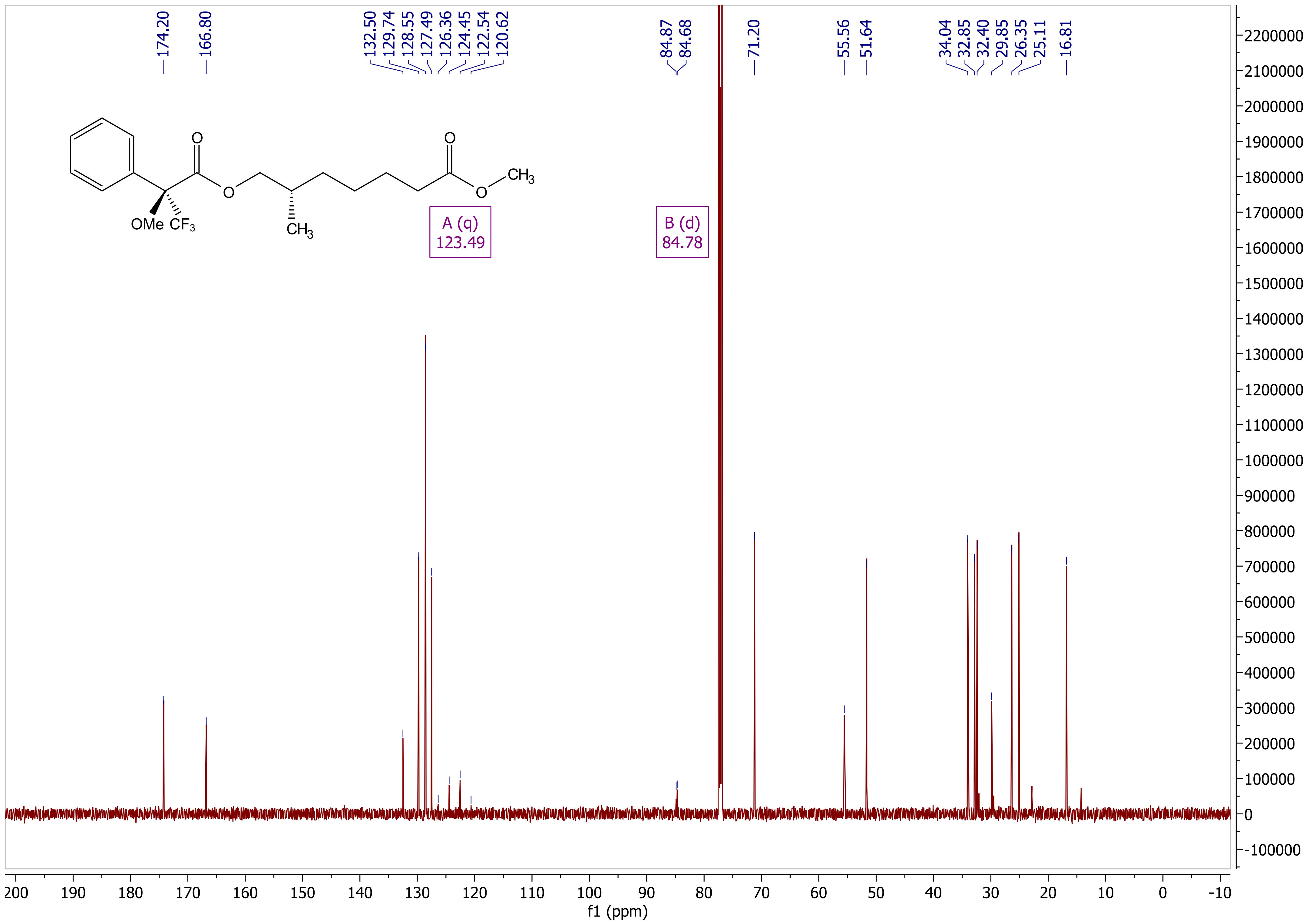

174.20
166.80
132.50
129.74
128.55
127.49
126.36
124.45
122.54
120.62
84.87
84.68
71.20
55.56
51.64
34.04
32.85
32.40
29.85
26.35
25.11
16.81
O
O
O
O
OMe CF3
CH3
CH3
A (q)
123.49
B (d)
84.78
f1 (ppm)

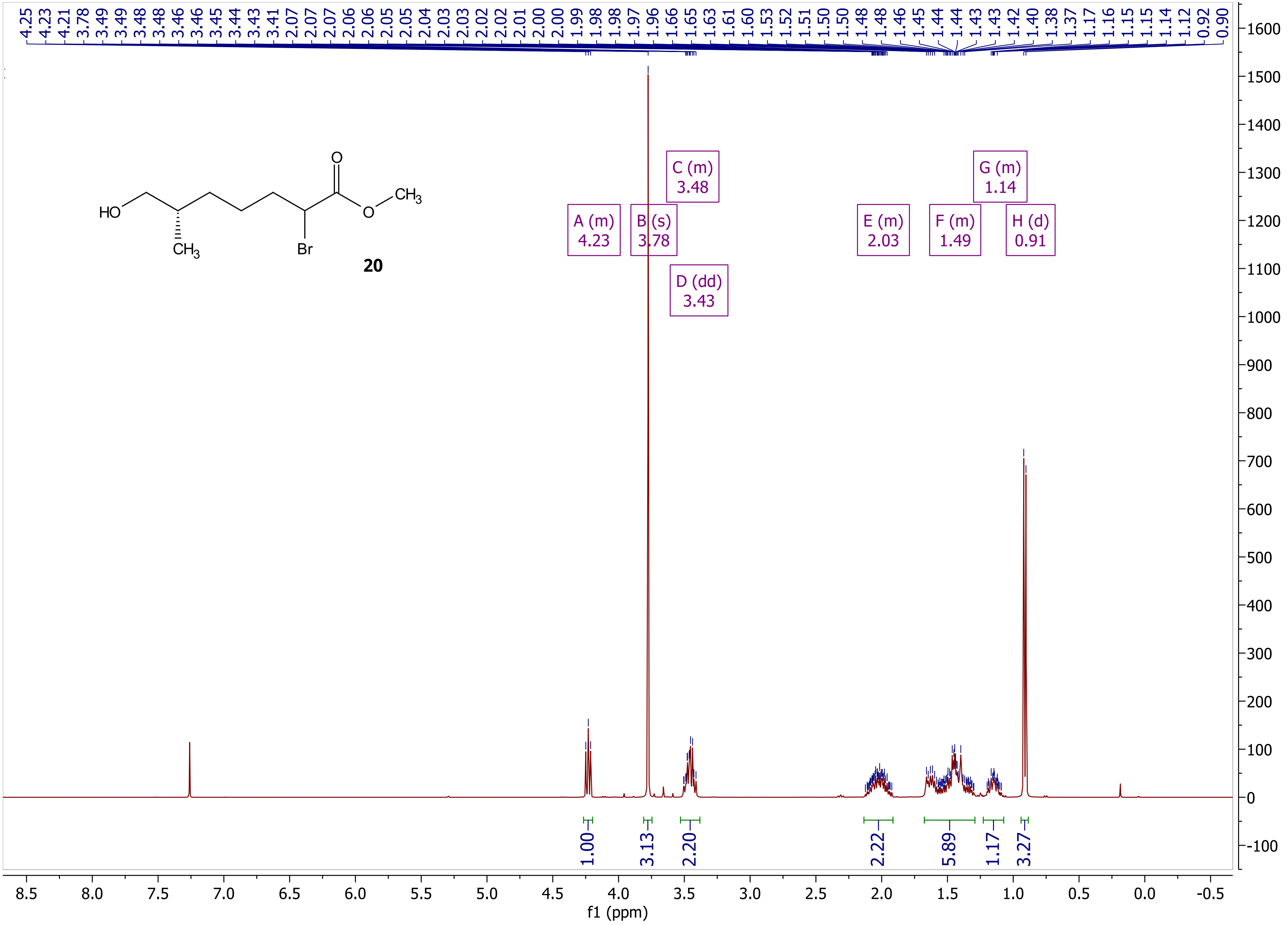
4.25
4.23
4.21
3.78
3.49
3.49
3.48
3.48
3.46
3.46
3.45
3.44
3.43
3.41
2.07
2.07
2.07
2.06
2.06
2.05
2.05
2.04
2.03
2.03
2.02
2.02
2.01
2.00
2.00
1.99
1.98
1.98
1.97
1.96
1.66
1.65
1.63
1.61
1.60
1.53
1.52
1.51
1.50
1.50
1.48
1.48
1.46
1.45
1.44
1.44
1.43
1.43
1.42
1.40
1.38
1.37
1.17
1.16
1.15
1.15
1.14
1.12
0.92
0.90
HO
CH3
Br
O
O
CH3
20
A (m) 4.23
B (s) 3.78
C (m) 3.48
D (dd) 3.43
E (m) 2.03
F (m) 1.49
G (m) 1.14
H (d) 0.91
1.00
3.13
2.20
2.22
5.89
1.17
3.27
f1 (ppm)

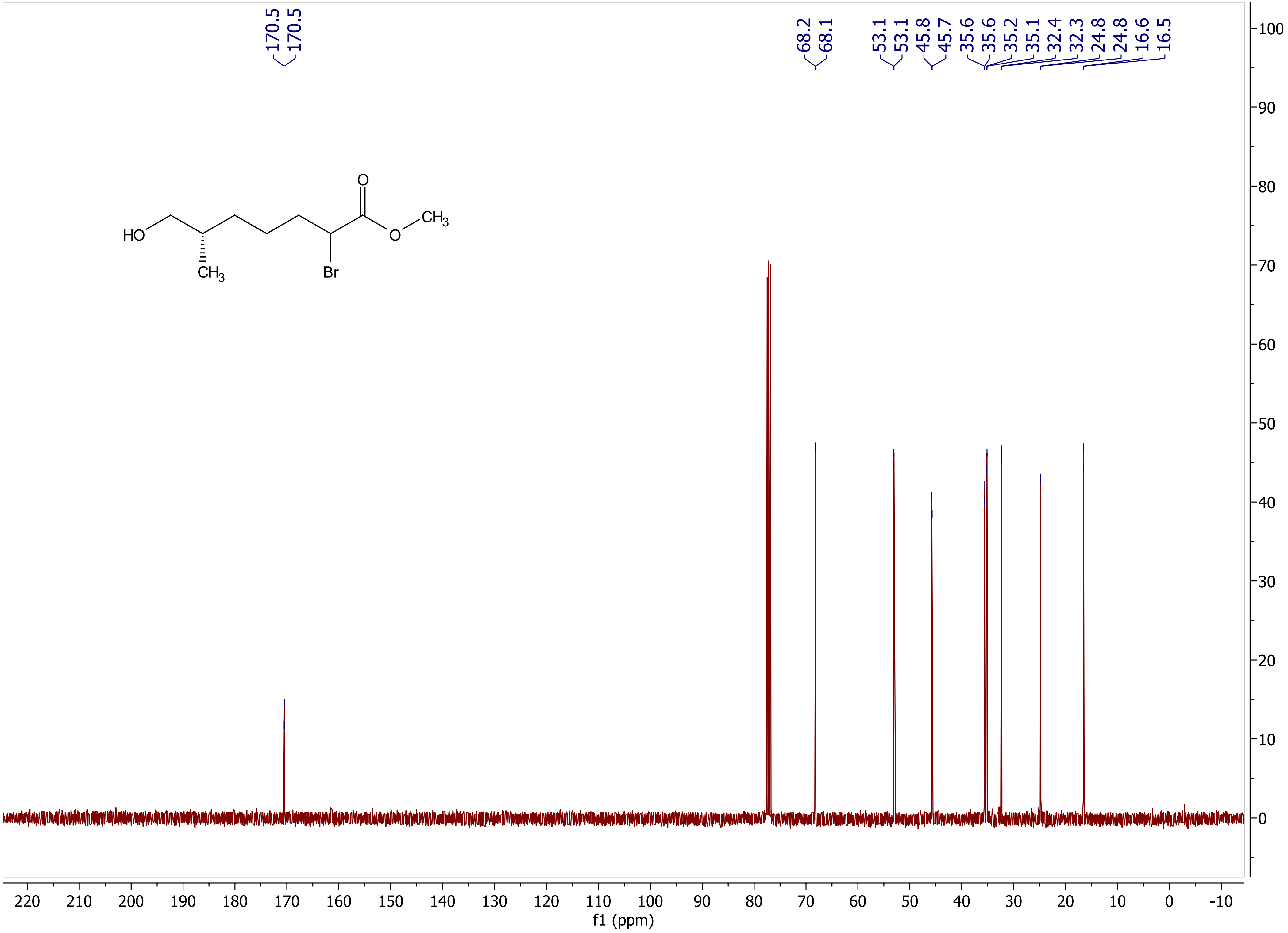

170.5
170.5
68.2
68.1
53.1
53.1
45.8
45.7
35.6
35.6
35.2
35.1
32.4
32.3
24.8
24.8
16.6
16.5
HO
CH_3
Br
O
O
CH_3
f1 (ppm)

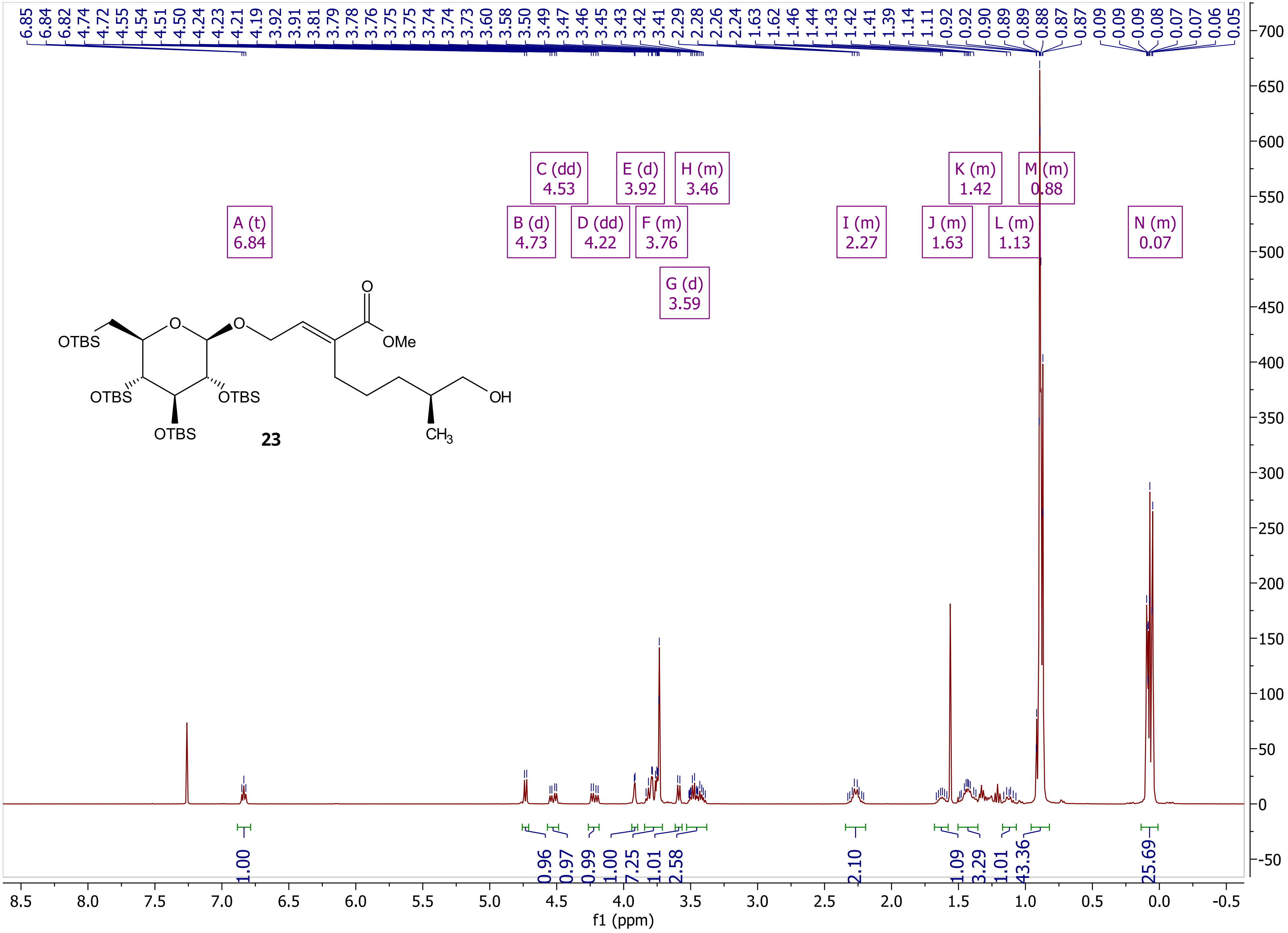

6.85 6.84 6.82 4.74 4.72 4.55 4.54 4.51 4.50 4.24 4.23 4.21 4.19 3.92 3.91 3.81 3.79 3.78 3.76 3.75 3.75 3.74 3.74 3.73 3.60 3.58 3.50 3.49 3.47 3.46 3.45 3.43 3.42 3.41 2.29 2.28 2.26 2.24 1.63 1.62 1.46 1.44 1.43 1.42 1.41 1.39 1.14 1.11 0.92 0.92 0.90 0.89 0.89 0.88 0.87 0.87 0.09 0.09 0.09 0.08 0.07 0.07 0.06 0.05
A (t) 6.84
B (d) 4.73
C (dd) 4.53
D (dd) 4.22
E (d) 3.92
F (m) 3.76
G (d) 3.59
H (m) 3.46
I (m) 2.27
J (m) 1.63
K (m) 1.42
L (m) 1.13
M (m) 0.88
N (m) 0.07
OTBS
OTBS
OTBS
OTBS
O
O
O
OMe
OH
CH3
23
1.00 0.96 0.97 0.99 1.00 7.25 1.01 2.58 2.10 1.09 3.29 1.01 43.36 25.69
f1 (ppm)

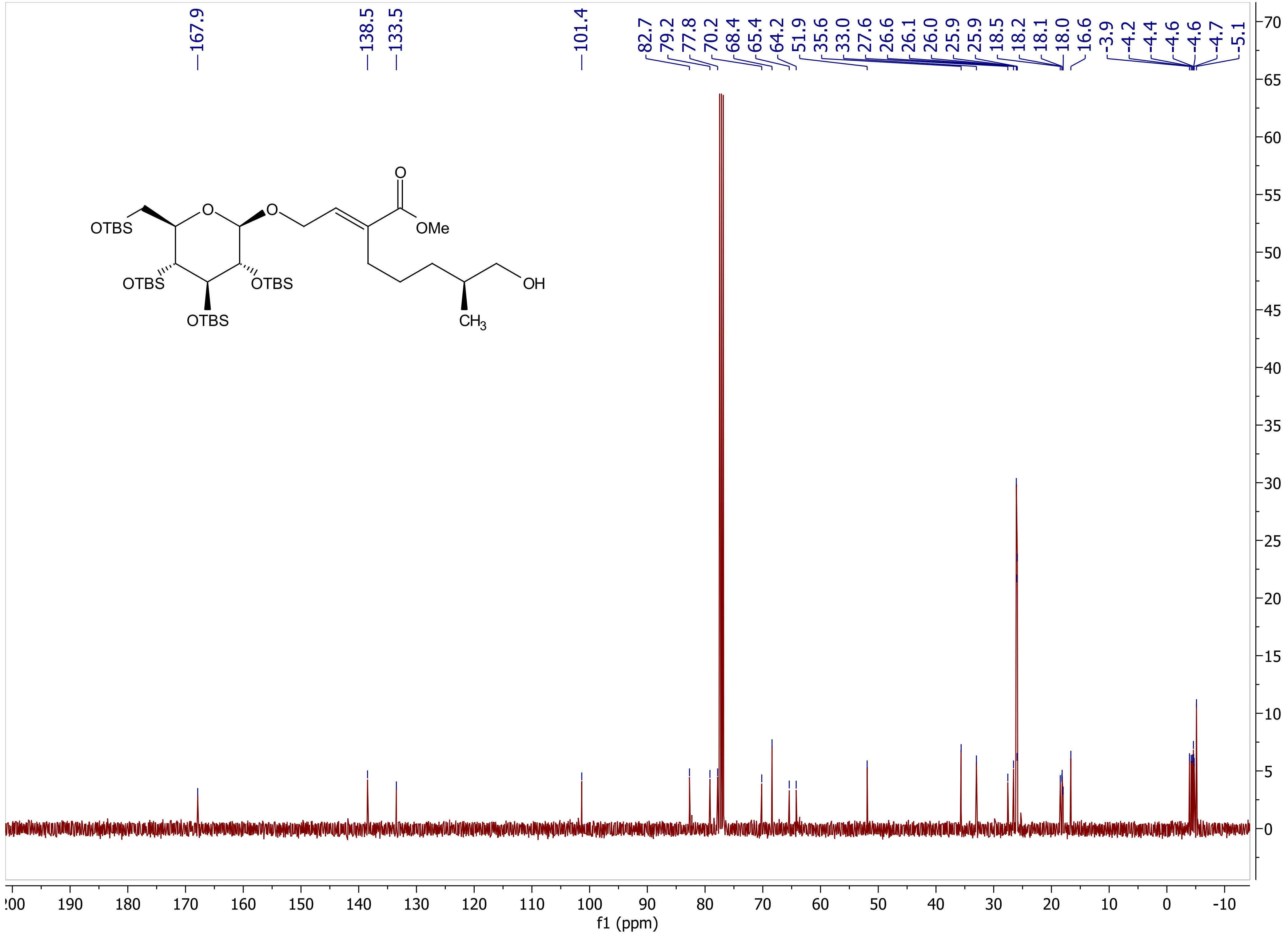
167.9
138.5
133.5
101.4
82.7
79.2
77.8
70.2
68.4
65.4
64.2
51.9
35.6
33.0
27.6
26.6
26.1
26.0
25.9
25.9
18.5
18.2
18.1
18.0
16.6
-3.9
-4.2
-4.4
-4.6
-4.6
-4.7
-5.1
O
OTBS
O
O
OMe
OTBS
OTBS
OTBS
OH
CH3
f1 (ppm)

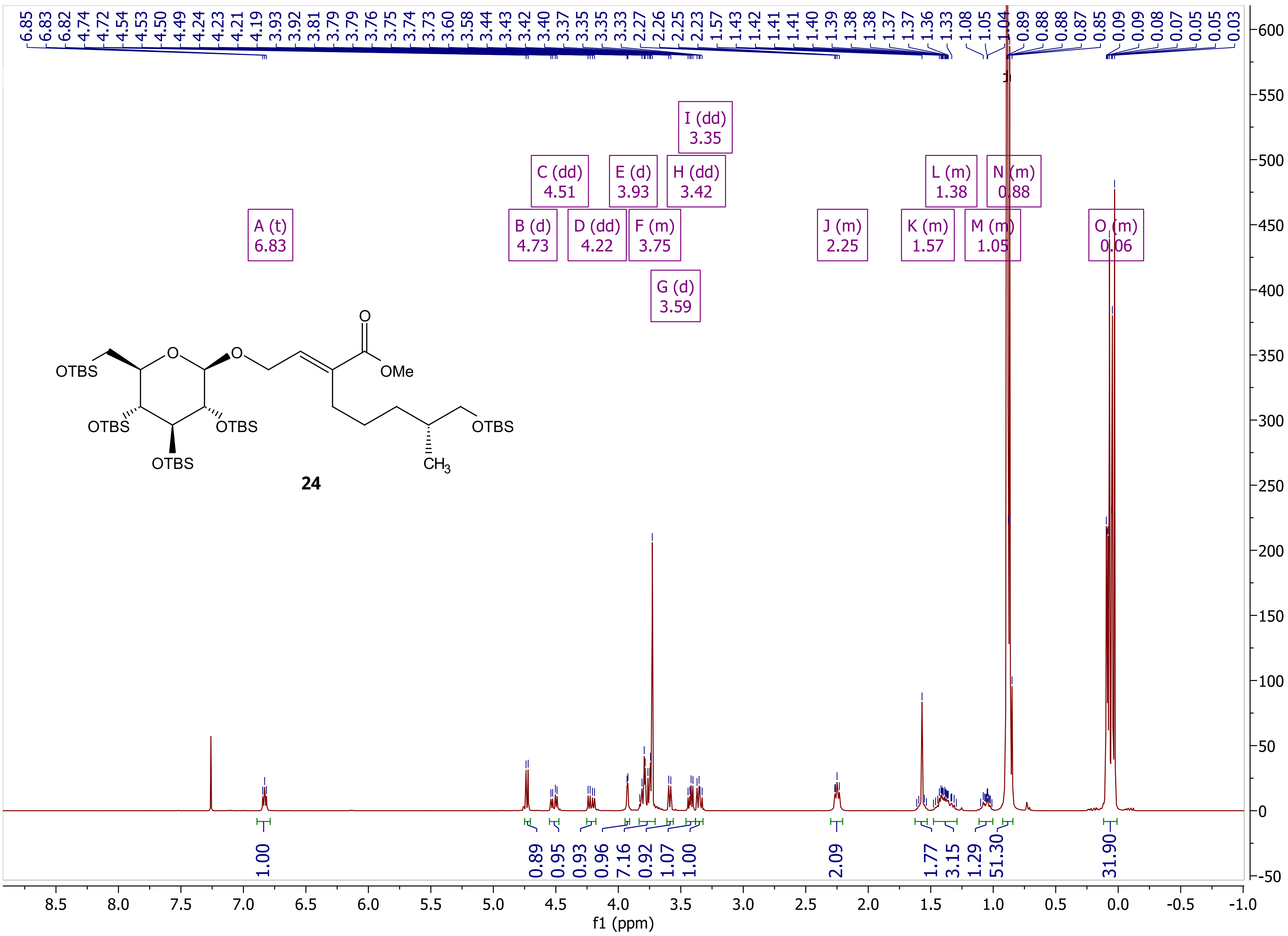

A (t) 6.83
B (d) 4.73
C (dd) 4.51
D (dd) 4.22
E (d) 3.93
F (m) 3.75
G (d) 3.59
H (dd) 3.42
I (dd) 3.35
J (m) 2.25
K (m) 1.57
L (m) 1.38
M (m) 1.05
N (m) 0.88
O (m) 0.06
OTBS
OTBS
OTBS
OTBS
OTBS
O
OMe
CH3
24
1.00
0.89
0.95
0.93
0.96
7.16
0.92
1.07
1.00
2.09
1.77
3.15
1.29
51.30
31.90
f1 (ppm)

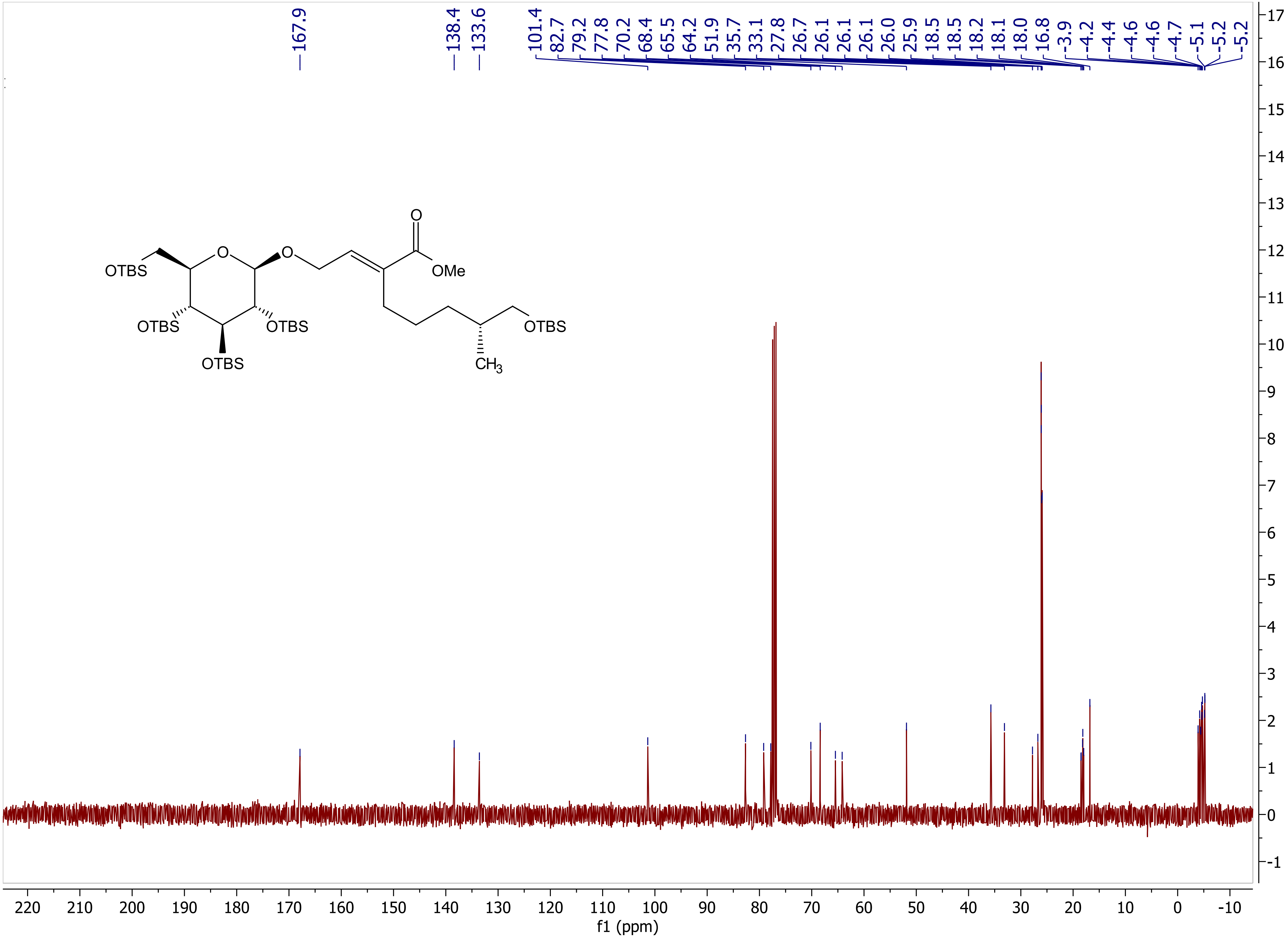
167.9
138.4
133.6
101.4
82.7
79.2
77.8
70.2
68.4
65.5
64.2
51.9
35.7
33.1
27.8
26.7
26.1
26.1
26.1
26.0
25.9
18.5
18.5
18.2
18.1
18.0
16.8
-3.9
-4.2
-4.4
-4.6
-4.6
-4.7
-5.1
-5.2
-5.2
OTBS
O
O
O
OMe
OTBS
OTBS
OTBS
OTBS
CH3
f1 (ppm)

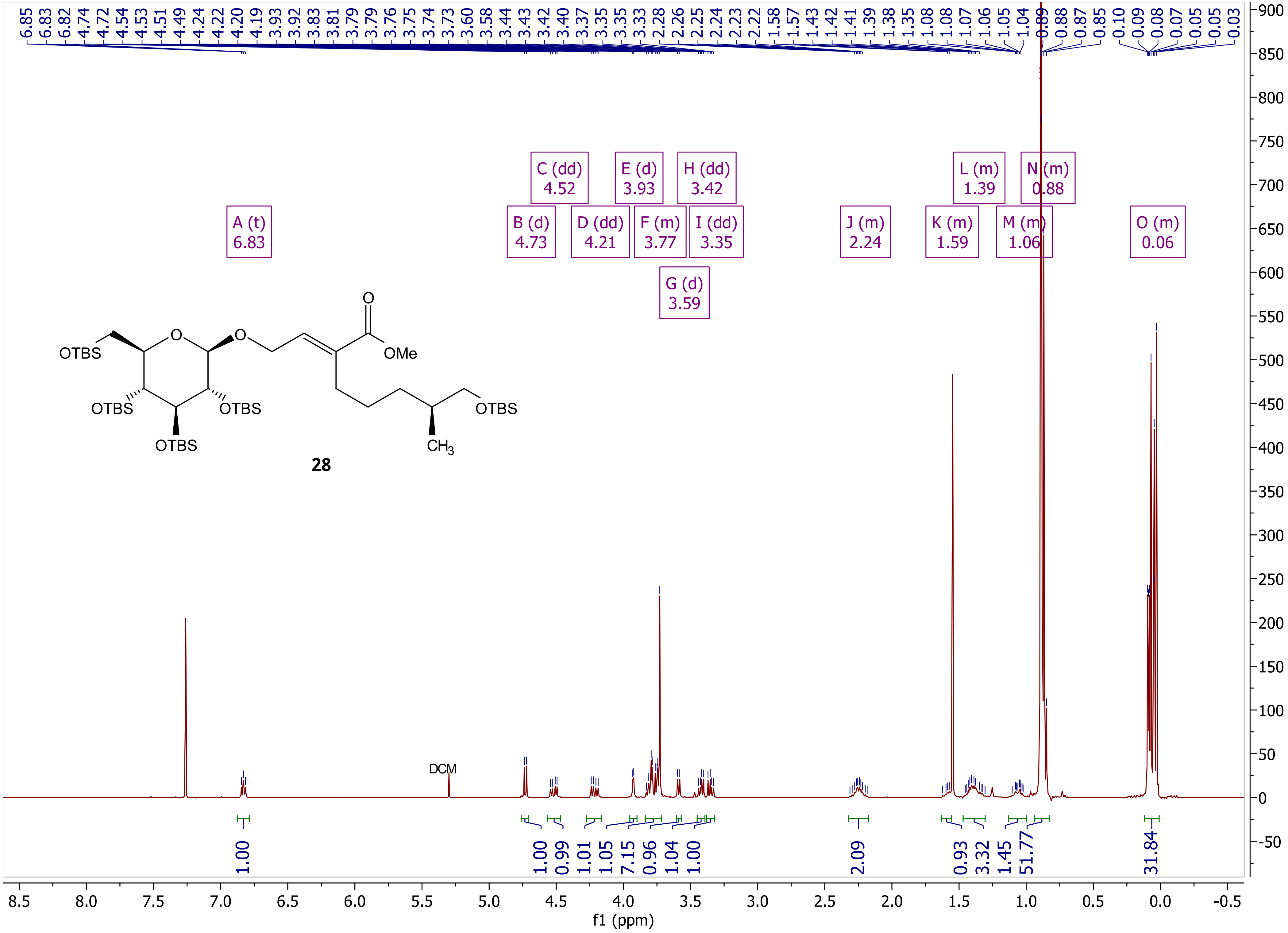
6.85 6.83 6.82 4.74 4.72 4.54 4.53 4.51 4.49 4.24 4.22 4.20 4.19 3.93 3.92 3.83 3.81 3.79 3.79 3.76 3.75 3.74 3.73 3.60 3.58 3.44 3.43 3.42 3.40 3.37 3.35 3.35 3.33 2.28 2.26 2.25 2.24 2.23 2.22 1.58 1.57 1.43 1.42 1.41 1.39 1.38 1.35 1.08 1.08 1.07 1.06 1.05 1.04 0.89 0.88 0.87 0.85 0.10 0.09 0.08 0.07 0.05 0.05 0.03
A (t) 6.83
B (d) 4.73
C (dd) 4.52
D (dd) 4.21
E (d) 3.93
F (m) 3.77
G (d) 3.59
H (dd) 3.42
I (dd) 3.35
J (m) 2.24
K (m) 1.59
L (m) 1.39
M (m) 1.06
N (m) 0.88
O (m) 0.06
OTBS
O
O
O
OMe
OTBS
OTBS
OTBS
OTBS
CH3
DCM
1.00 1.00 0.99 1.01 1.05 7.15 0.96 1.04 1.00 2.09 0.93 3.32 1.45 51.77 31.84
f1 (ppm)

28

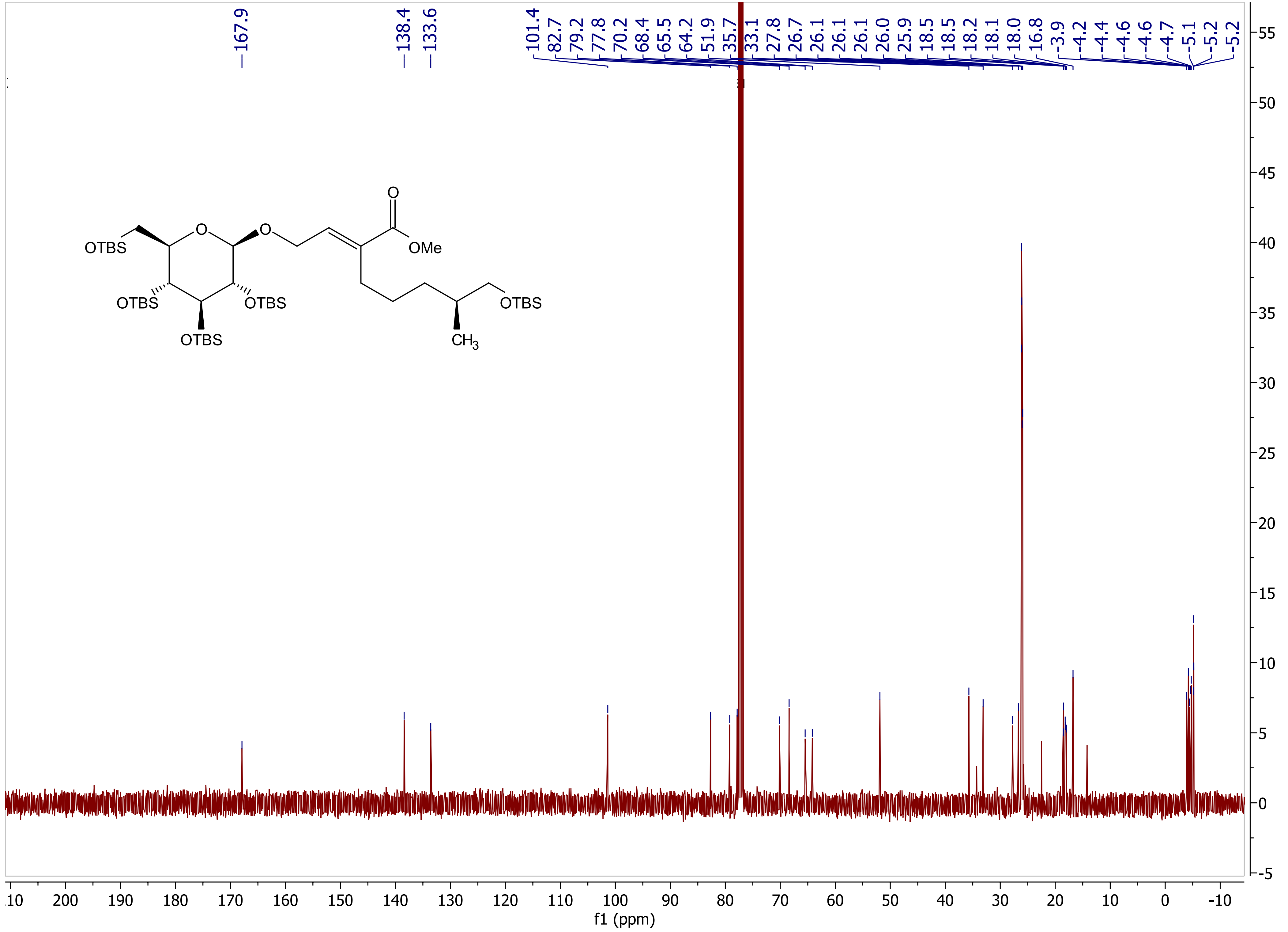

167.9
138.4
133.6
101.4
82.7
79.2
77.8
70.2
68.4
65.5
64.2
51.9
35.7
33.1
27.8
26.7
26.1
26.1
26.1
26.0
25.9
18.5
18.5
18.2
18.1
18.0
16.8
-3.9
-4.2
-4.4
-4.6
-4.6
-4.7
-5.1
-5.2
-5.2
OTBS
OTBS
OTBS
OTBS
OMe
OTBS
CH3
f1 (ppm)

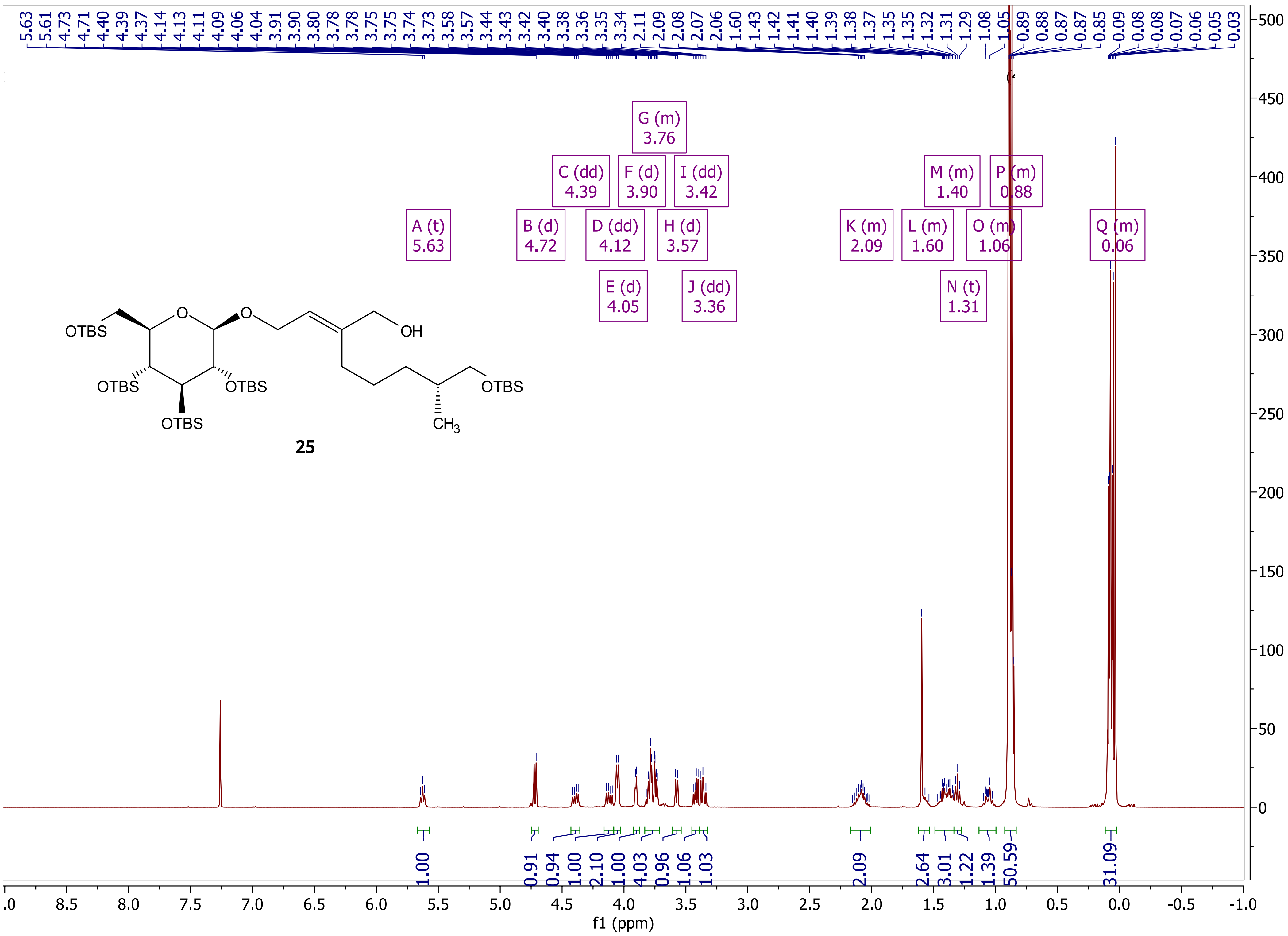
A (t) 5.63
B (d) 4.72
C (dd) 4.39
D (dd) 4.12
E (d) 4.05
F (d) 3.90
G (m) 3.76
H (d) 3.57
I (dd) 3.42
J (dd) 3.36
K (m) 2.09
L (m) 1.60
M (m) 1.40
N (t) 1.31
O (m) 1.06
P (m) 0.88
Q (m) 0.06
OTBS
OTBS
OTBS
OTBS
OTBS
OH
CH3
25
1.00
0.91
0.94
1.00
2.10
1.00
4.03
0.96
1.06
1.03
2.09
2.64
3.01
1.22
1.39
50.59
31.09
f1 (ppm)

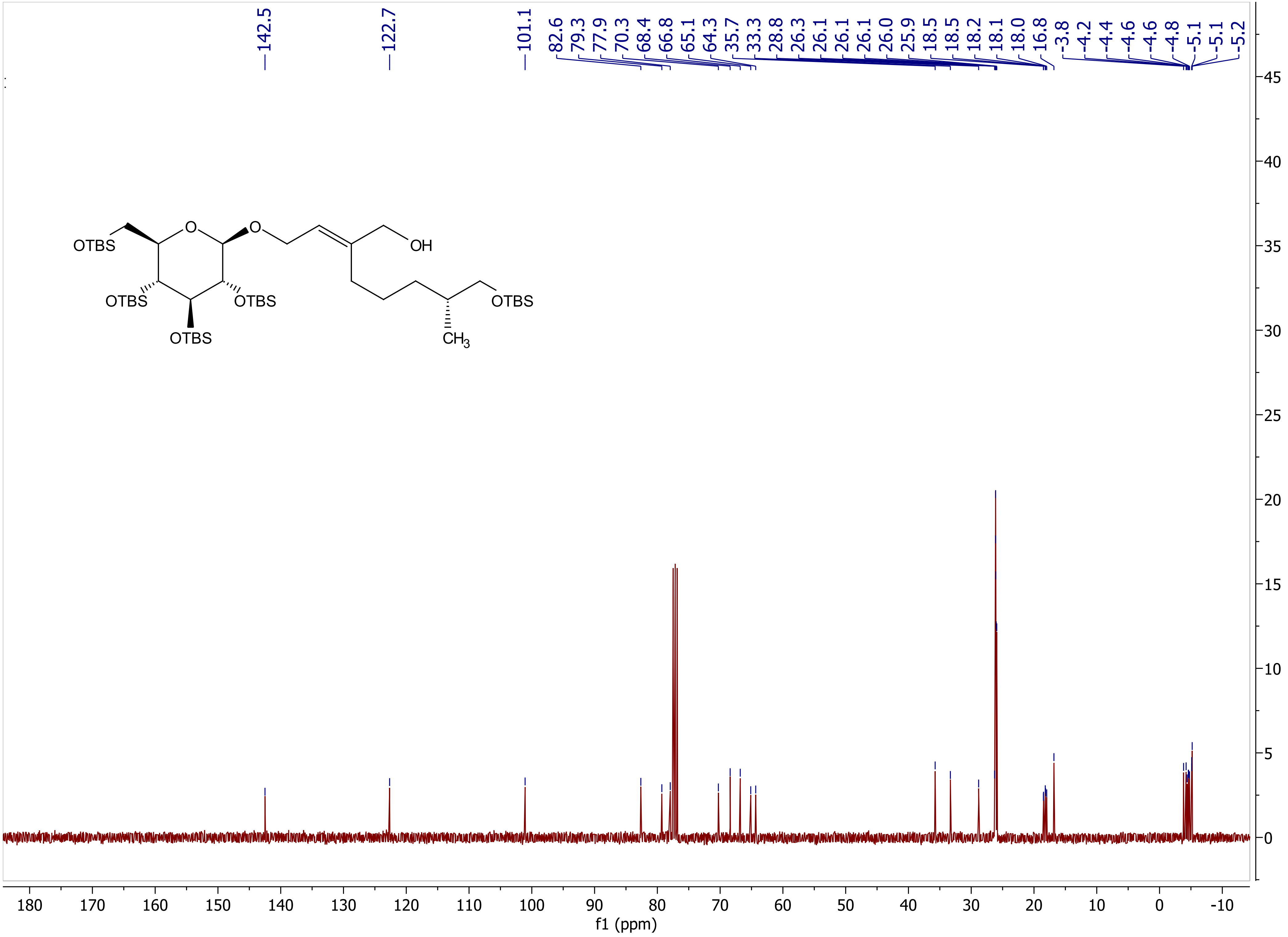

142.5
122.7
101.1
82.6
79.3
77.9
70.3
68.4
66.8
65.1
64.3
35.7
33.3
28.8
26.3
26.1
26.1
26.1
26.0
25.9
18.5
18.5
18.2
18.1
18.0
16.8
-3.8
-4.2
-4.4
-4.6
-4.6
-4.8
-5.1
-5.1
-5.2
OTBS
O
O
OH
OTBS
OTBS
OTBS
OTBS
CH3
f1 (ppm)

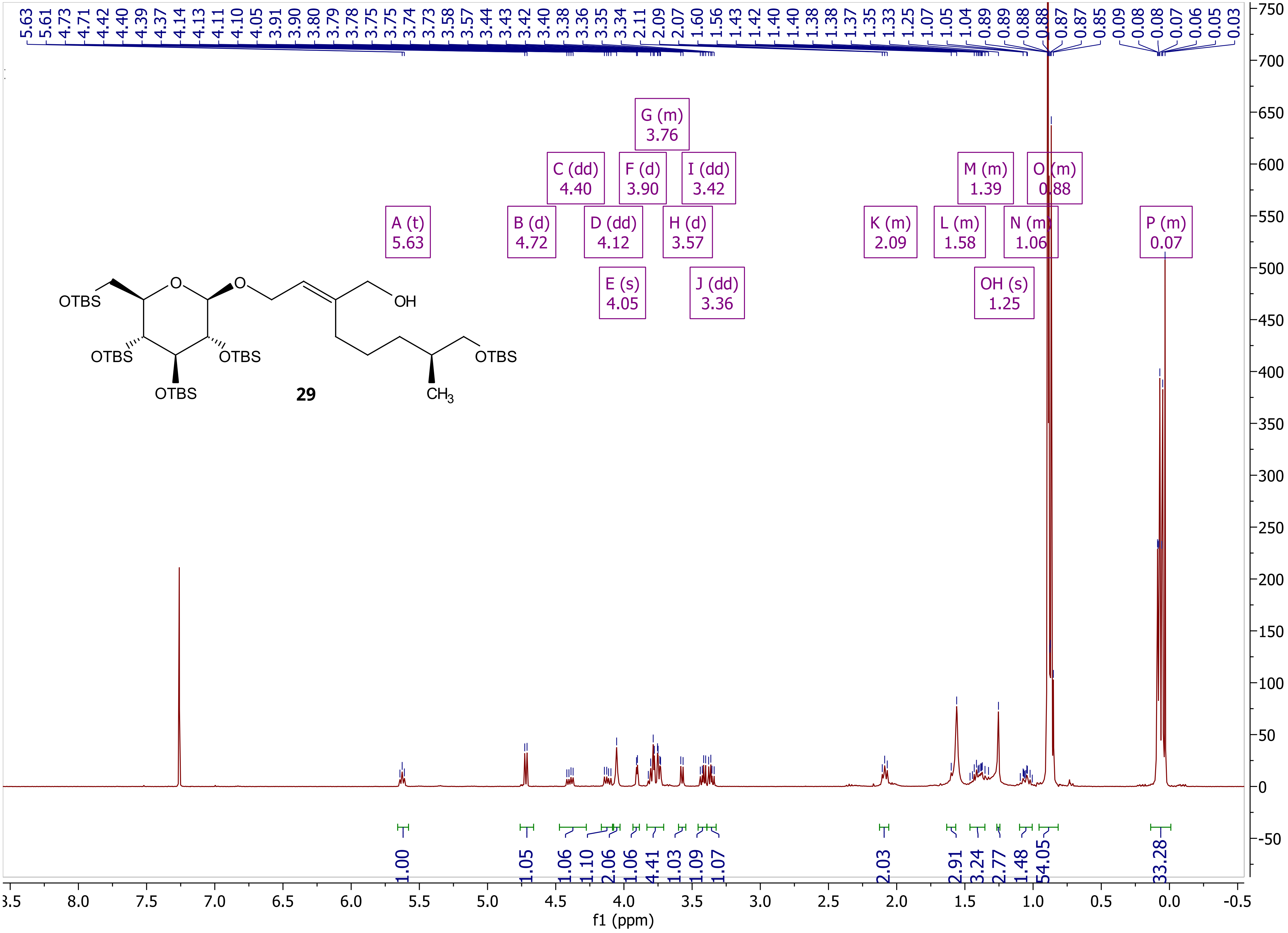
5.63 5.61 4.73 4.71 4.42 4.40 4.39 4.37 4.14 4.13 4.11 4.10 4.05 3.91 3.90 3.80 3.79 3.78 3.75 3.75 3.74 3.73 3.58 3.57 3.44 3.43 3.42 3.40 3.38 3.36 3.35 3.34 2.11 2.09 2.07 1.60 1.56 1.43 1.42 1.40 1.40 1.38 1.38 1.37 1.35 1.33 1.25 1.07 1.05 1.04 0.89 0.89 0.88 0.88 0.87 0.87 0.85 0.09 0.08 0.08 0.07 0.06 0.05 0.03
A (t) 5.63
B (d) 4.72
C (dd) 4.40
D (dd) 4.12
E (s) 4.05
F (d) 3.90
G (m) 3.76
H (d) 3.57
I (dd) 3.42
J (dd) 3.36
K (m) 2.09
L (m) 1.58
M (m) 1.39
OH (s) 1.25
N (m) 1.06
O (m) 0.88
P (m) 0.07
OTBS
O
O
OH
OTBS
OTBS
OTBS
OTBS
29
CH3
1.00 1.05 1.06 1.10 2.06 1.06 4.41 1.03 1.09 1.07 2.03 2.91 3.24 2.77 1.48 54.05 33.28
8.5 8.0 7.5 7.0 6.5 6.0 5.5 5.0 4.5 4.0 3.5 3.0 2.5 2.0 1.5 1.0 0.5 0.0 -0.5
f1 (ppm)
750 700 650 600 550 500 450 400 350 300 250 200 150 100 50 0 -50

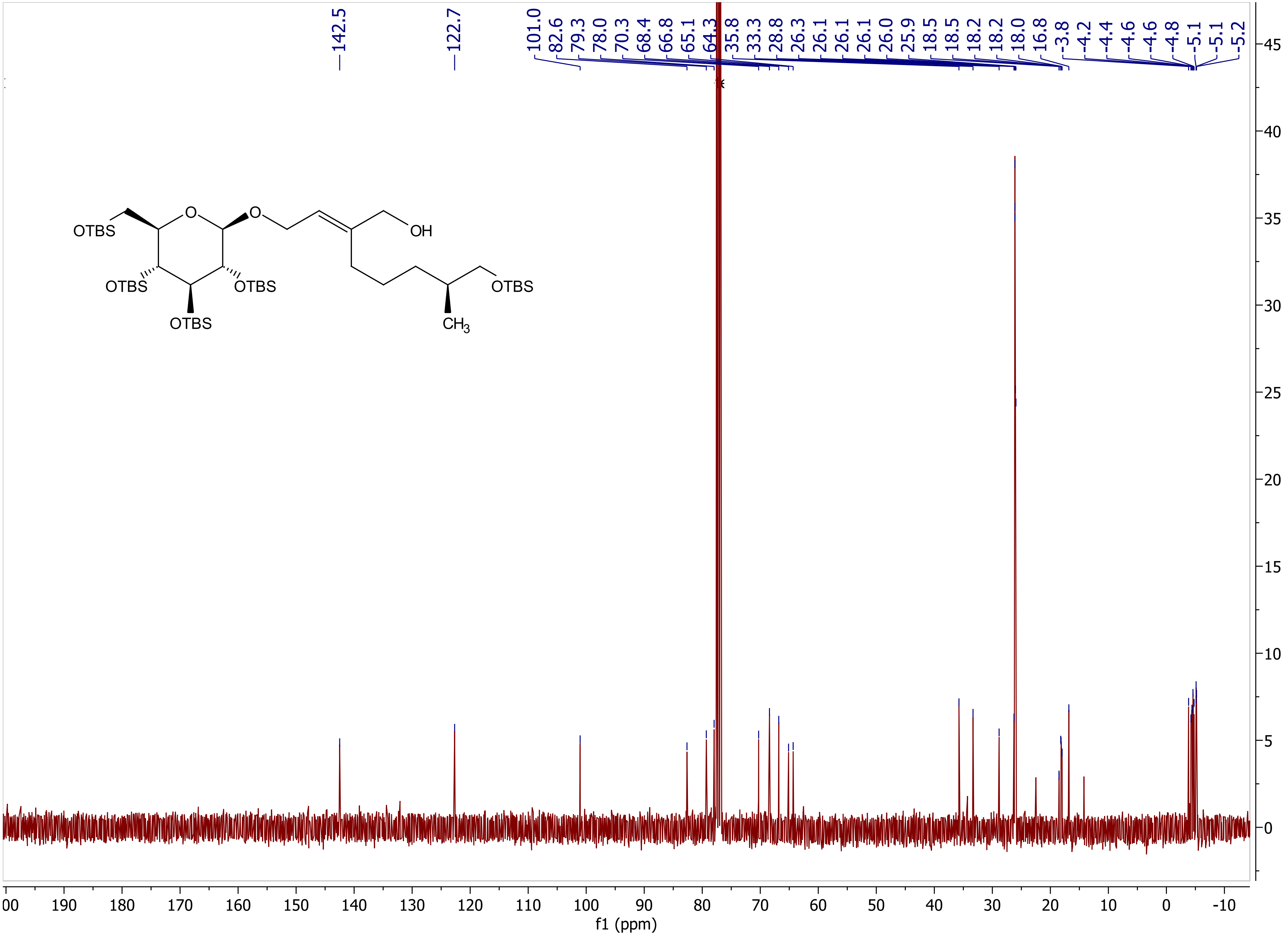
142.5
122.7
101.0
82.6
79.3
78.0
70.3
68.4
66.8
65.1
64.3
35.8
33.3
28.8
26.3
26.1
26.1
26.1
26.0
25.9
18.5
18.5
18.2
18.2
18.0
16.8
-3.8
-4.2
-4.4
-4.6
-4.6
-4.8
-5.1
-5.1
-5.2
OTBS
O
O
OH
OTBS
OTBS
OTBS
OTBS
CH_3
f1 (ppm)

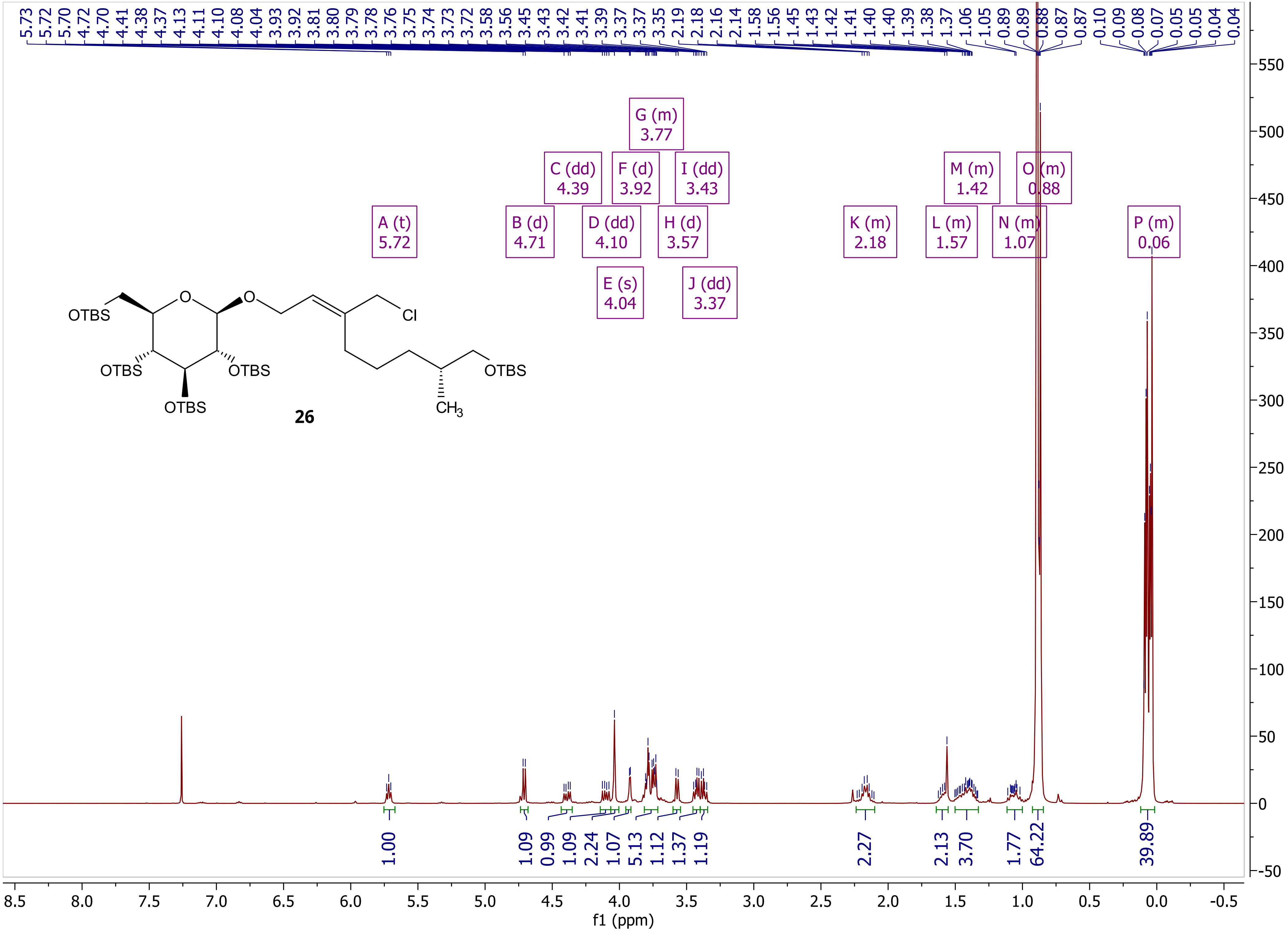

A (t) 5.72
B (d) 4.71
C (dd) 4.39
D (dd) 4.10
E (s) 4.04
F (d) 3.92
G (m) 3.77
H (d) 3.57
I (dd) 3.43
J (dd) 3.37
K (m) 2.18
L (m) 1.57
M (m) 1.42
N (m) 1.07
O (m) 0.88
P (m) 0.06
OTBS
Cl
CH3
26
f1 (ppm)

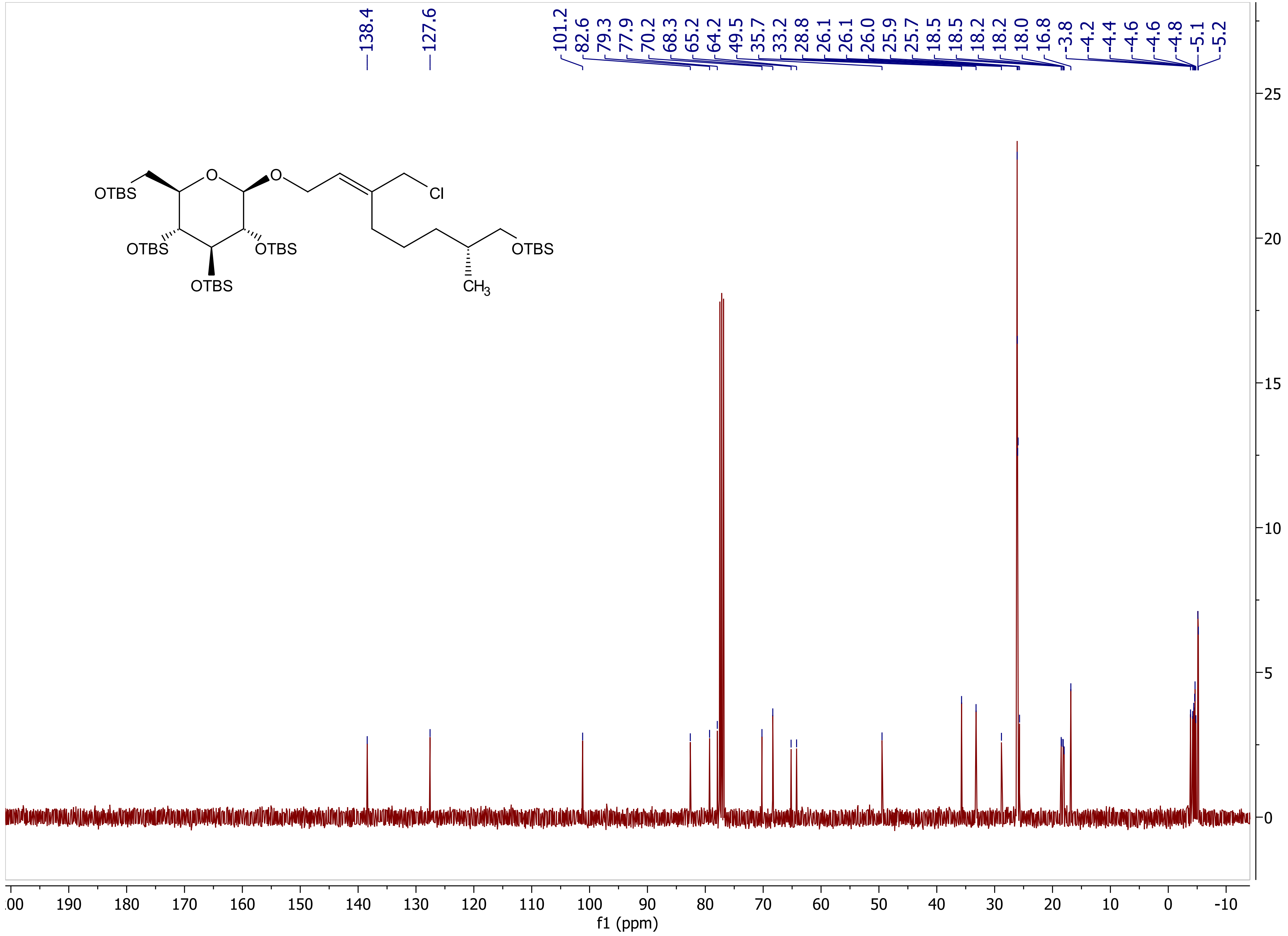

138.4
127.6
101.2
82.6
79.3
77.9
70.2
68.3
65.2
64.2
49.5
35.7
33.2
28.8
26.1
26.1
26.0
25.9
25.7
18.5
18.5
18.2
18.2
18.0
16.8
-3.8
-4.2
-4.4
-4.6
-4.6
-4.8
-5.1
-5.2
OTBS
O
O
Cl
OTBS
OTBS
OTBS
OTBS
CH3
f1 (ppm)

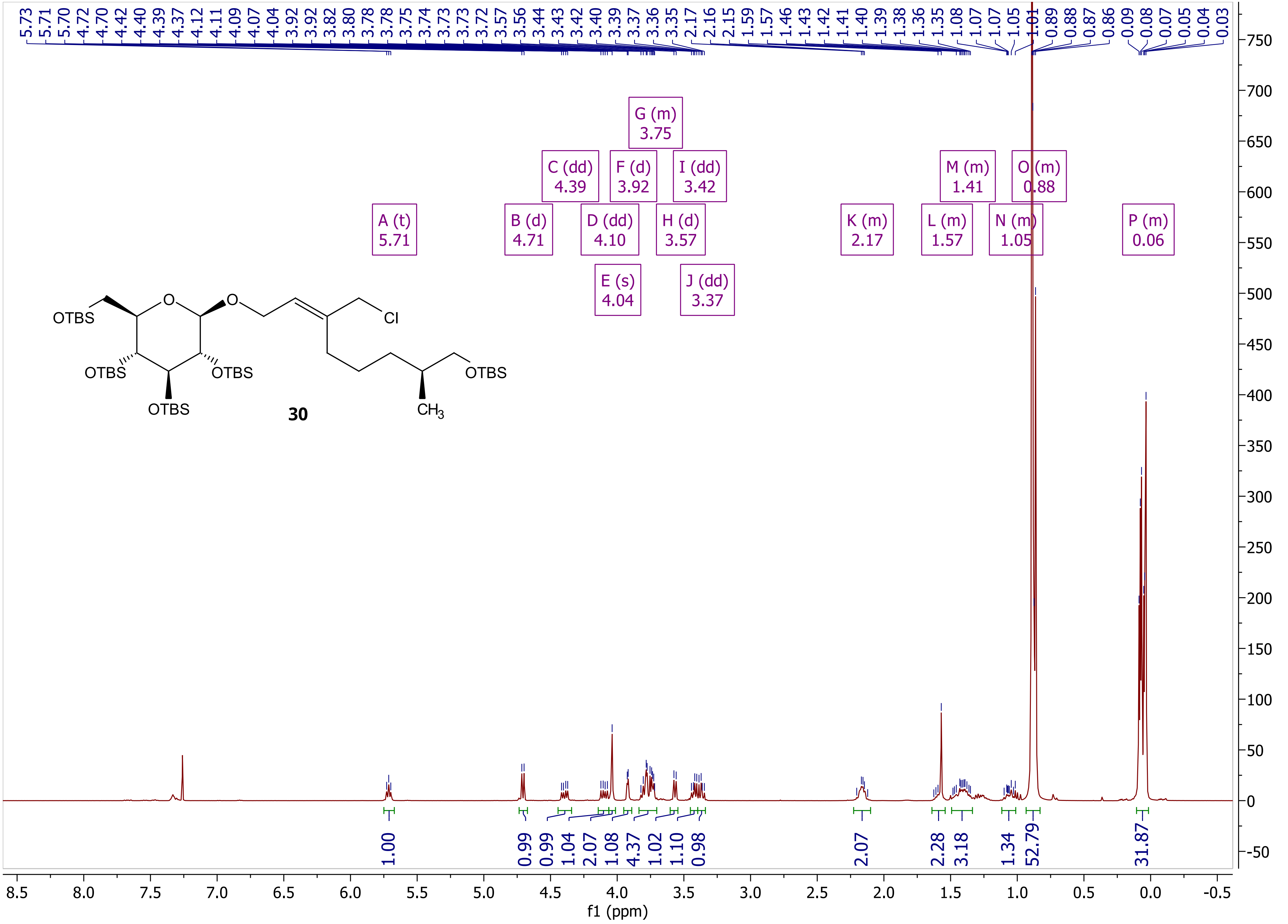

5.73 5.71 5.70 4.72 4.70 4.42 4.40 4.39 4.37 4.12 4.11 4.09 4.07 4.04 3.92 3.92 3.82 3.80 3.78 3.78 3.75 3.74 3.73 3.73 3.73 3.72 3.57 3.56 3.44 3.43 3.42 3.40 3.39 3.37 3.36 3.35 2.17 2.16 2.15 1.59 1.57 1.46 1.43 1.42 1.41 1.40 1.39 1.38 1.36 1.35 1.08 1.07 1.07 1.05 1.01 0.89 0.88 0.87 0.86 0.09 0.08 0.07 0.05 0.04 0.03
G (m) 3.75
C (dd) 4.39
F (d) 3.92
I (dd) 3.42
M (m) 1.41
O (m) 0.88
A (t) 5.71
B (d) 4.71
D (dd) 4.10
H (d) 3.57
K (m) 2.17
L (m) 1.57
N (m) 1.05
P (m) 0.06
E (s) 4.04
J (dd) 3.37
OTBS
O
O
Cl
OTBS
OTBS
OTBS
OTBS
CH3
30
1.00 0.99 0.99 1.04 2.07 1.08 4.37 1.02 1.10 0.98 2.07 2.28 3.18 1.34 52.79 31.87
f1 (ppm)

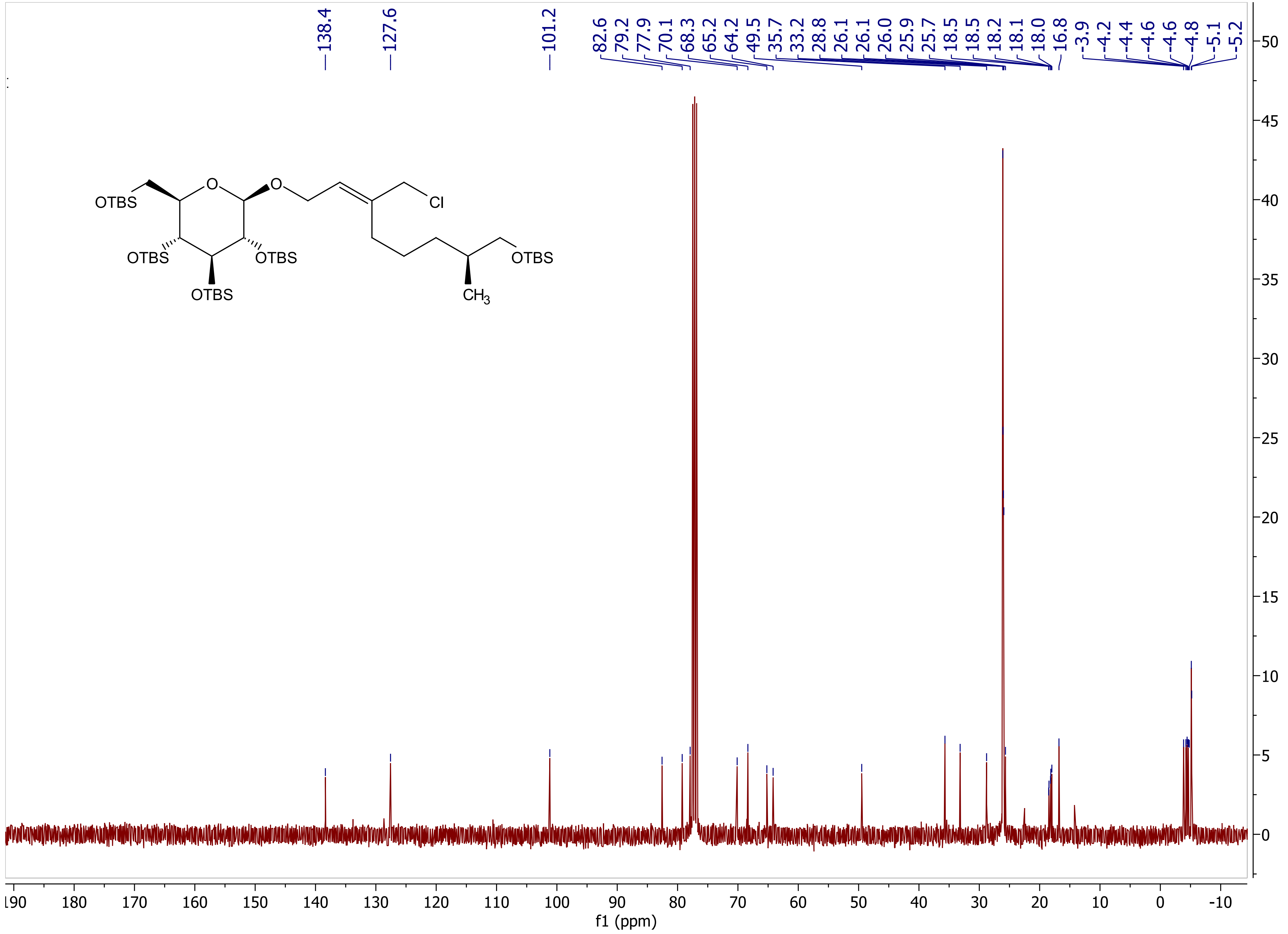
138.4
127.6
101.2
82.6
79.2
77.9
70.1
68.3
65.2
64.2
49.5
35.7
33.2
28.8
26.1
26.1
26.0
25.9
25.7
18.5
18.5
18.2
18.1
18.0
16.8
-3.9
-4.2
-4.4
-4.6
-4.6
-4.8
-5.1
-5.2
OTBS
O
O
Cl
OTBS
OTBS
OTBS
OTBS
CH3
f1 (ppm)

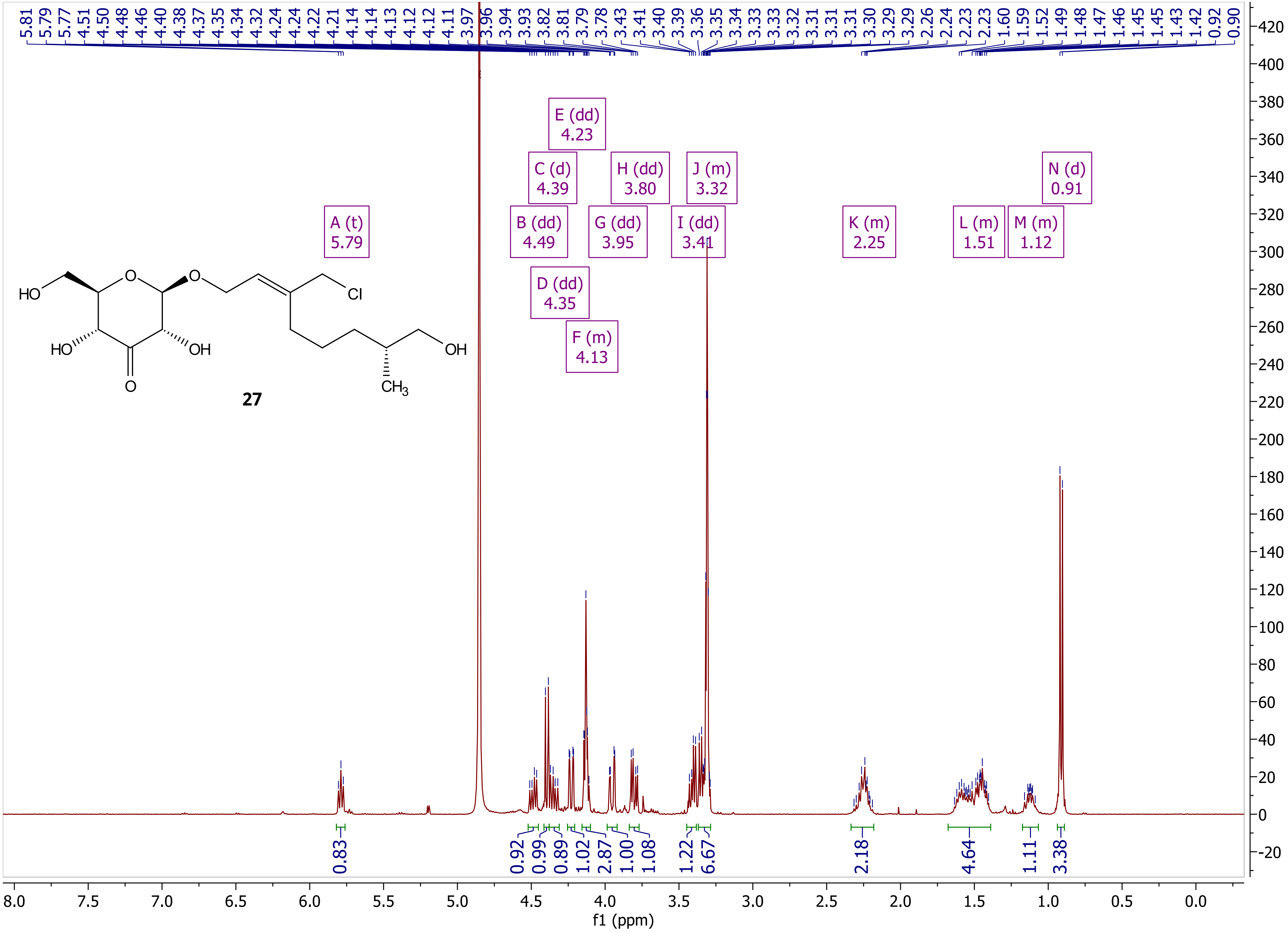

5.81 5.79 5.77 4.51 4.50 4.48 4.46 4.40 4.38 4.37 4.35 4.34 4.32 4.24 4.24 4.22 4.21 4.14 4.14 4.13 4.12 4.12 4.11 3.97 3.96 3.94 3.93 3.82 3.81 3.79 3.78 3.43 3.41 3.40 3.39 3.36 3.35 3.34 3.33 3.33 3.32 3.31 3.31 3.31 3.30 3.29 3.29 2.26 2.24 2.23 2.23 1.60 1.59 1.52 1.49 1.48 1.47 1.46 1.45 1.45 1.43 1.42 0.92 0.90
A (t) 5.79
B (dd) 4.49
C (d) 4.39
D (dd) 4.35
E (dd) 4.23
F (m) 4.13
G (dd) 3.95
H (dd) 3.80
I (dd) 3.41
J (m) 3.32
K (m) 2.25
L (m) 1.51
M (m) 1.12
N (d) 0.91
HO
O
O
Cl
HO
OH
OH
O
CH3
27
0.83 0.92 0.99 0.89 1.02 2.87 1.00 1.08 1.22 6.67 2.18 4.64 1.11 3.38
8.0 7.5 7.0 6.5 6.0 5.5 5.0 4.5 4.0 3.5 3.0 2.5 2.0 1.5 1.0 0.5 0.0
f1 (ppm)
420 400 380 360 340 320 300 280 260 240 220 200 180 160 140 120 100 80 60 40 20 0 -20

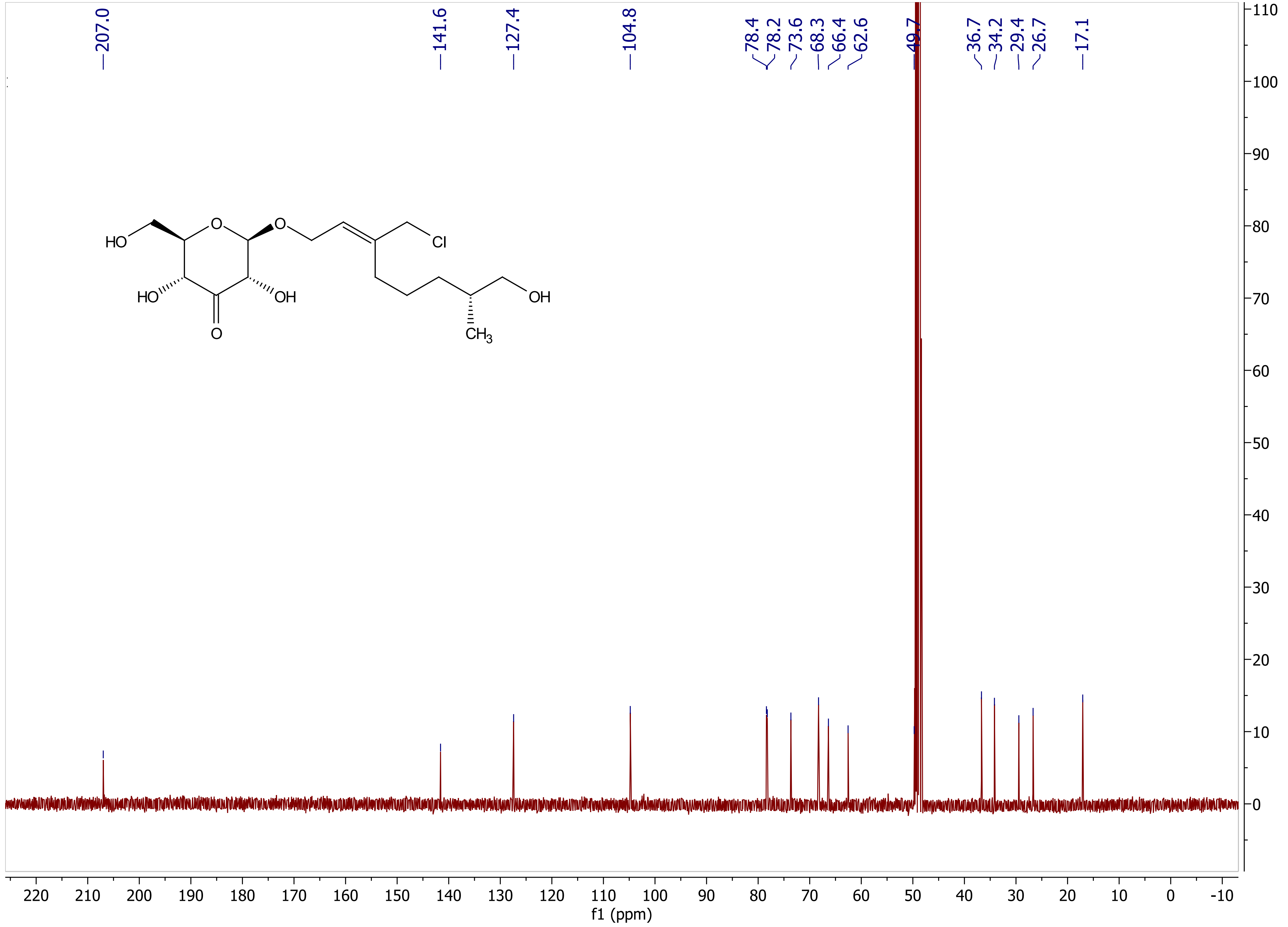
207.0
141.6
127.4
104.8
78.4
78.2
73.6
68.3
66.4
62.6
49.7
36.7
34.2
29.4
26.7
17.1
HO
O
O
Cl
HO
OH
OH
O
CH3
f1 (ppm)

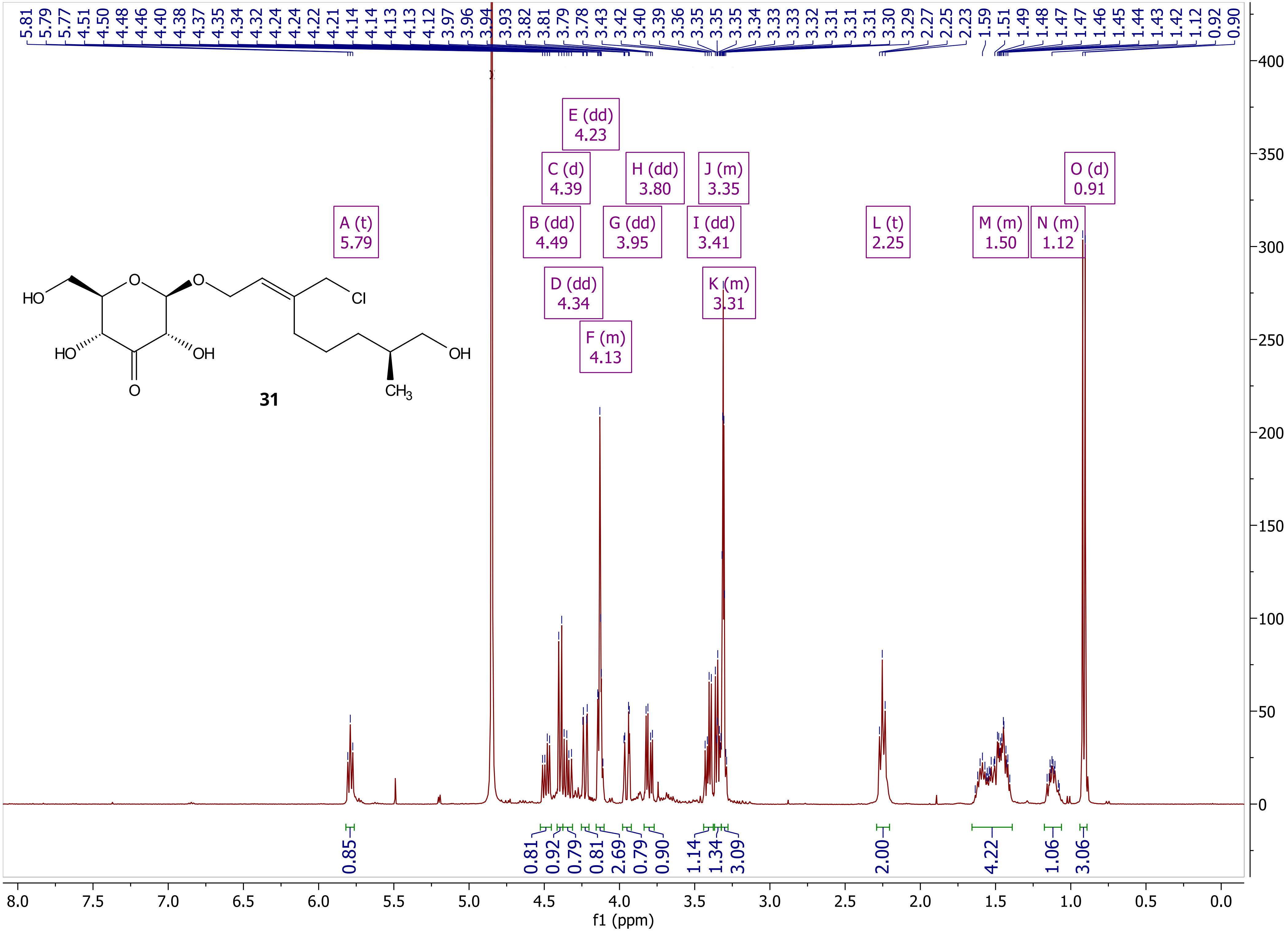

5.81 5.79 5.77 4.51 4.50 4.48 4.46 4.40 4.38 4.37 4.35 4.34 4.32 4.24 4.24 4.22 4.21 4.14 4.14 4.13 4.13 4.12 3.97 3.96 3.94 3.93 3.82 3.81 3.79 3.78 3.43 3.42 3.40 3.39 3.36 3.35 3.35 3.35 3.34 3.33 3.33 3.32 3.31 3.31 3.31 3.30 3.29 2.27 2.25 2.23 1.59 1.51 1.49 1.48 1.47 1.47 1.46 1.45 1.44 1.43 1.42 1.12 0.92 0.90
A (t) 5.79
B (dd) 4.49
C (d) 4.39
D (dd) 4.34
E (dd) 4.23
F (m) 4.13
G (dd) 3.95
H (dd) 3.80
I (dd) 3.41
J (m) 3.35
K (m) 3.31
L (t) 2.25
M (m) 1.50
N (m) 1.12
O (d) 0.91
HO
O
O
Cl
HO
OH
OH
O
CH3
31
0.85
0.81
0.92
0.79
0.81
2.69
0.79
0.90
1.14
1.34
3.09
2.00
4.22
1.06
3.06
f1 (ppm)

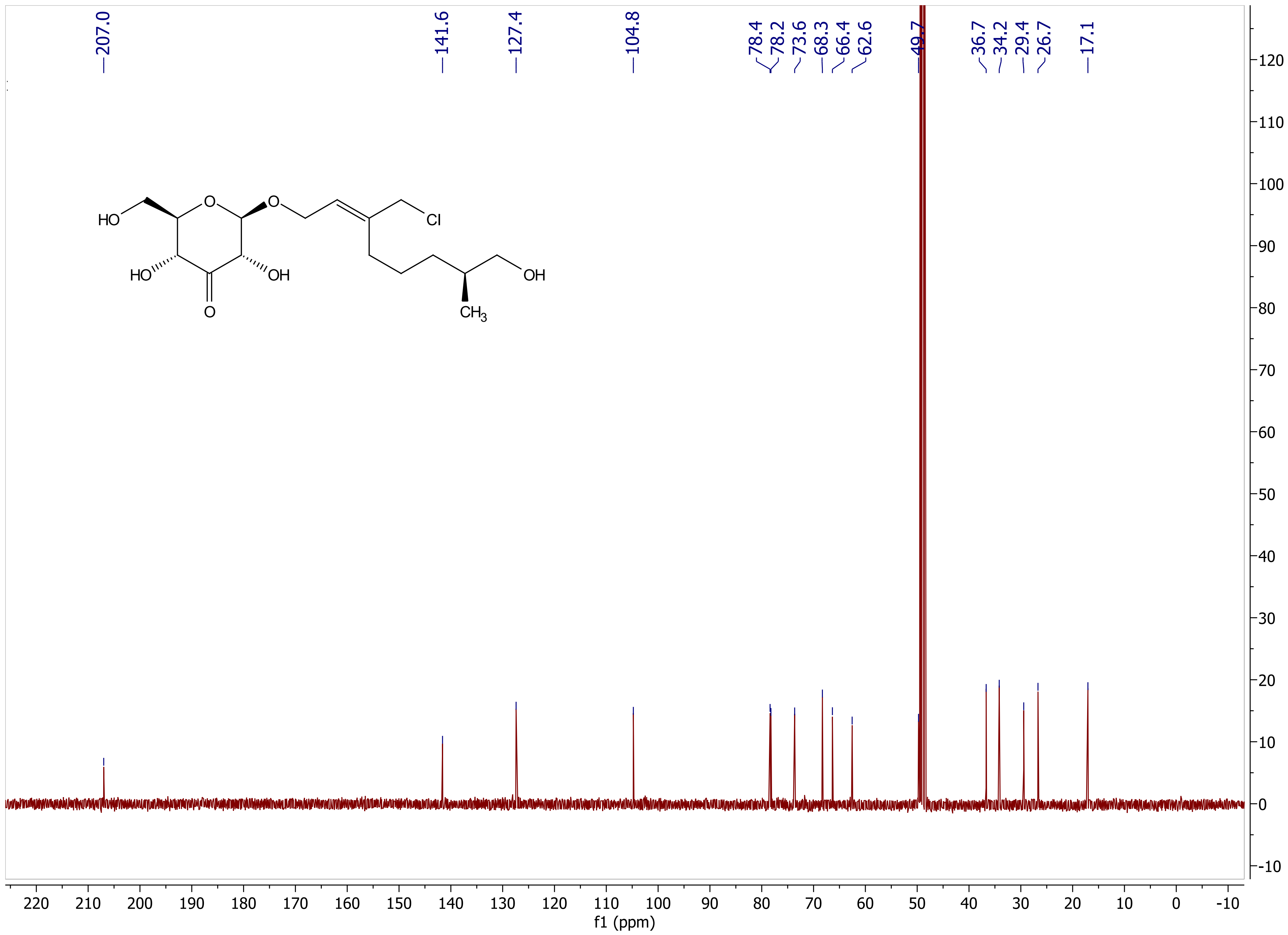

207.0
141.6
127.4
104.8
78.4
78.2
73.6
68.3
66.4
62.6
49.7
36.7
34.2
29.4
26.7
17.1
HO
O
O
Cl
HO
OH
OH
O
CH3
f1 (ppm)

1H NMR (600 MHz, CD3OD) of 7-epi-dissectol A

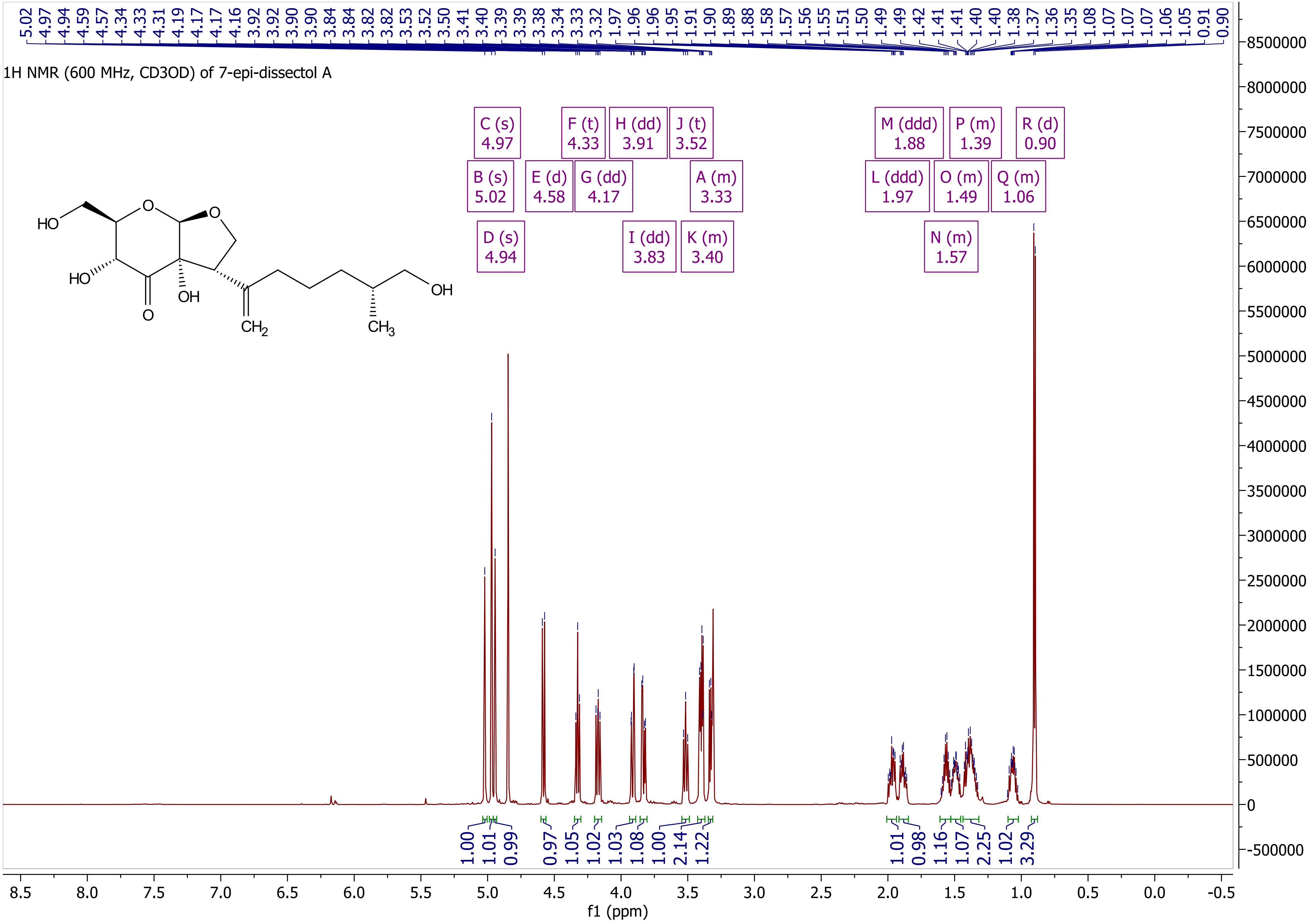

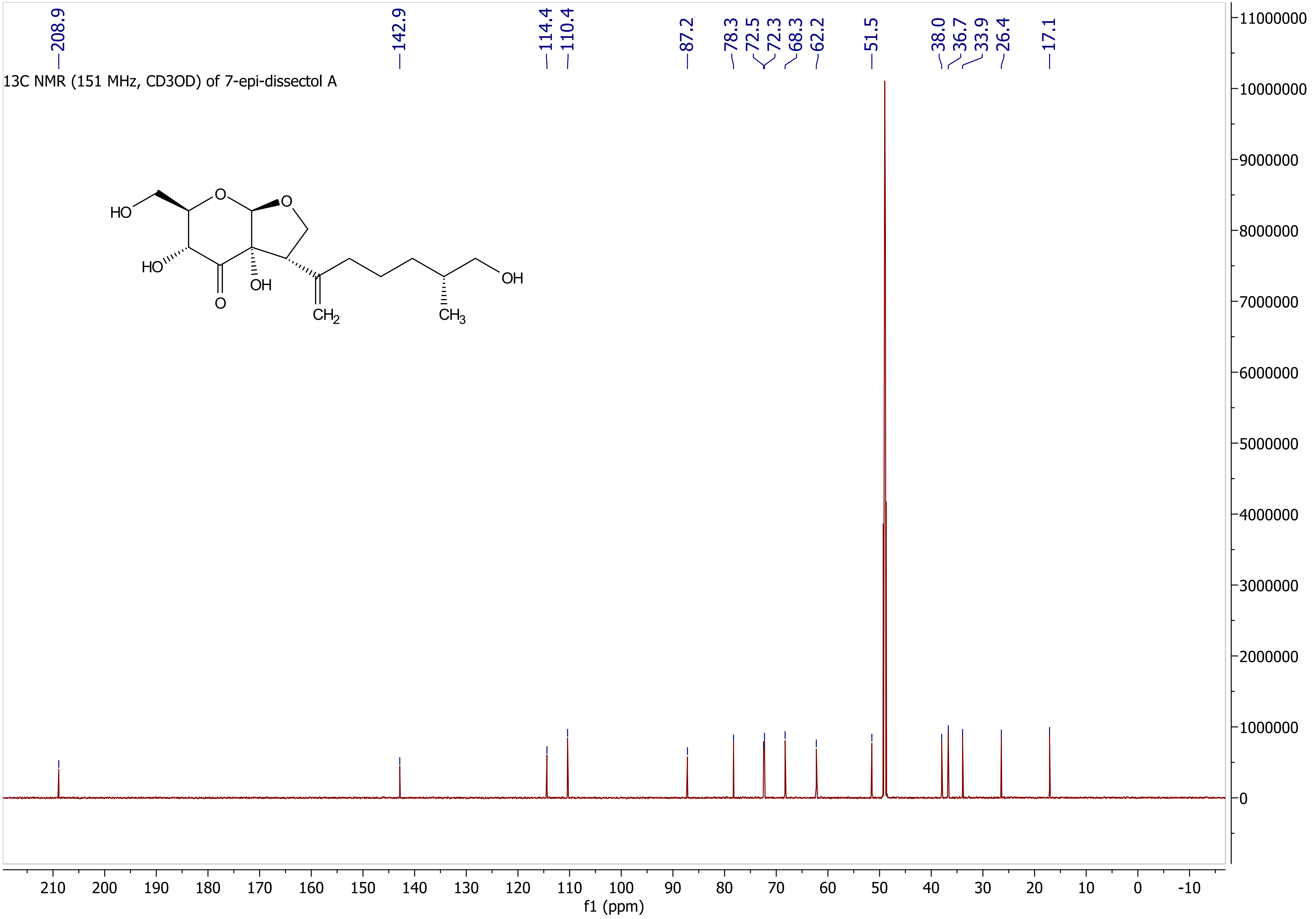
208.9
142.9
114.4
110.4
87.2
78.3
72.5
72.3
68.3
62.2
51.5
38.0
36.7
33.9
26.4
17.1
13C NMR (151 MHz, CD3OD) of 7-epi-dissectol A
HO
O
O
HO
OH
O
OH
CH2
CH3
11000000
10000000
9000000
8000000
7000000
6000000
5000000
4000000
3000000
2000000
1000000
0
210
200
190
180
170
160
150
140
130
120
110
100
90
80
70
60
50
40
30
20
10
0
-10
f1 (ppm)

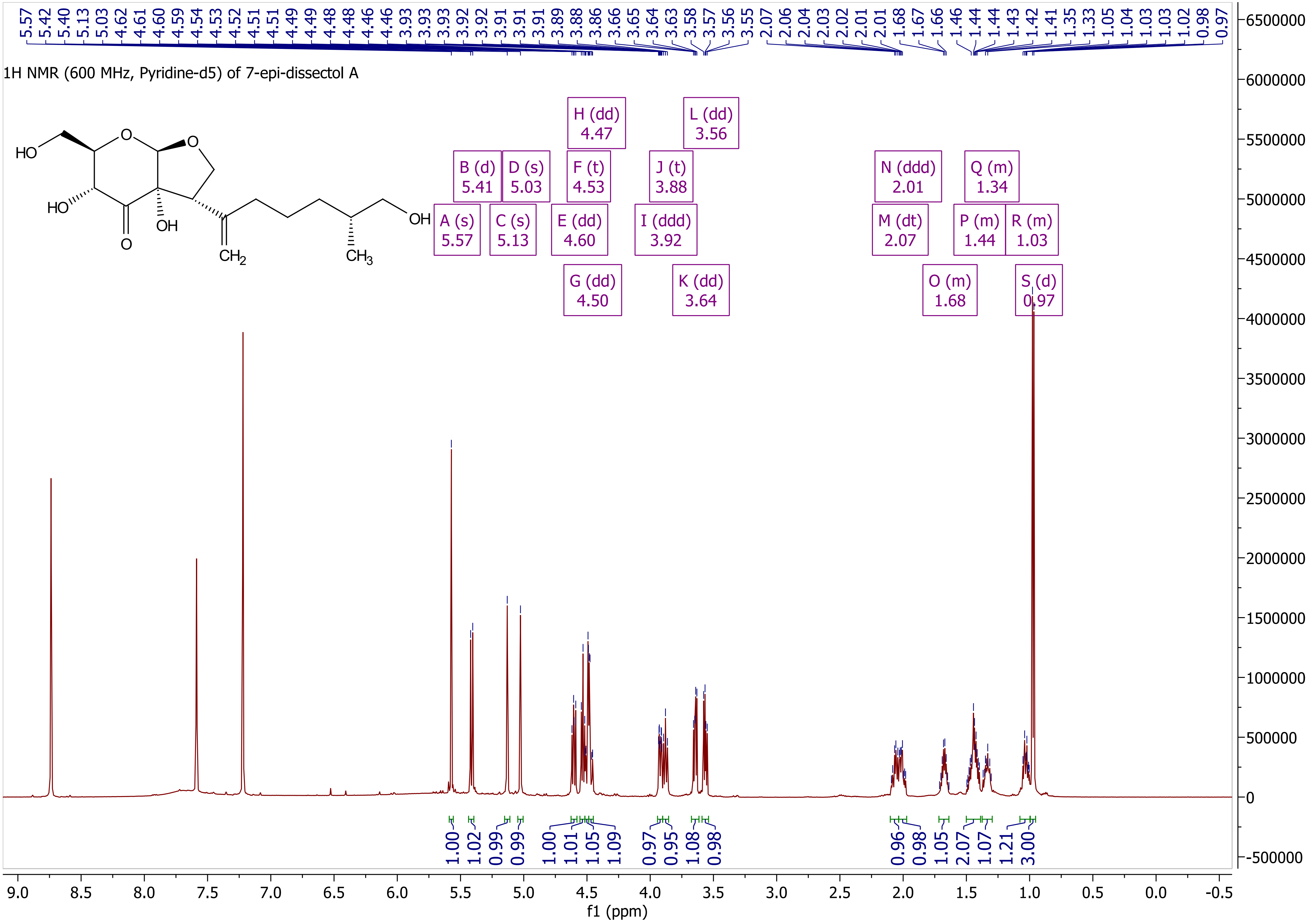
1H NMR (600 MHz, Pyridine-d5) of 7-epi-dissectol A
A (s) 5.57
B (d) 5.41
C (s) 5.13
D (s) 5.03
E (dd) 4.60
F (t) 4.53
G (dd) 4.50
H (dd) 4.47
I (ddd) 3.92
J (t) 3.88
K (dd) 3.64
L (dd) 3.56
M (dt) 2.07
N (ddd) 2.01
O (m) 1.68
P (m) 1.44
Q (m) 1.34
R (m) 1.03
S (d) 0.97
f1 (ppm)

13C NMR (151 MHz, Pyridine-d5) of 7-epi-dissectol A

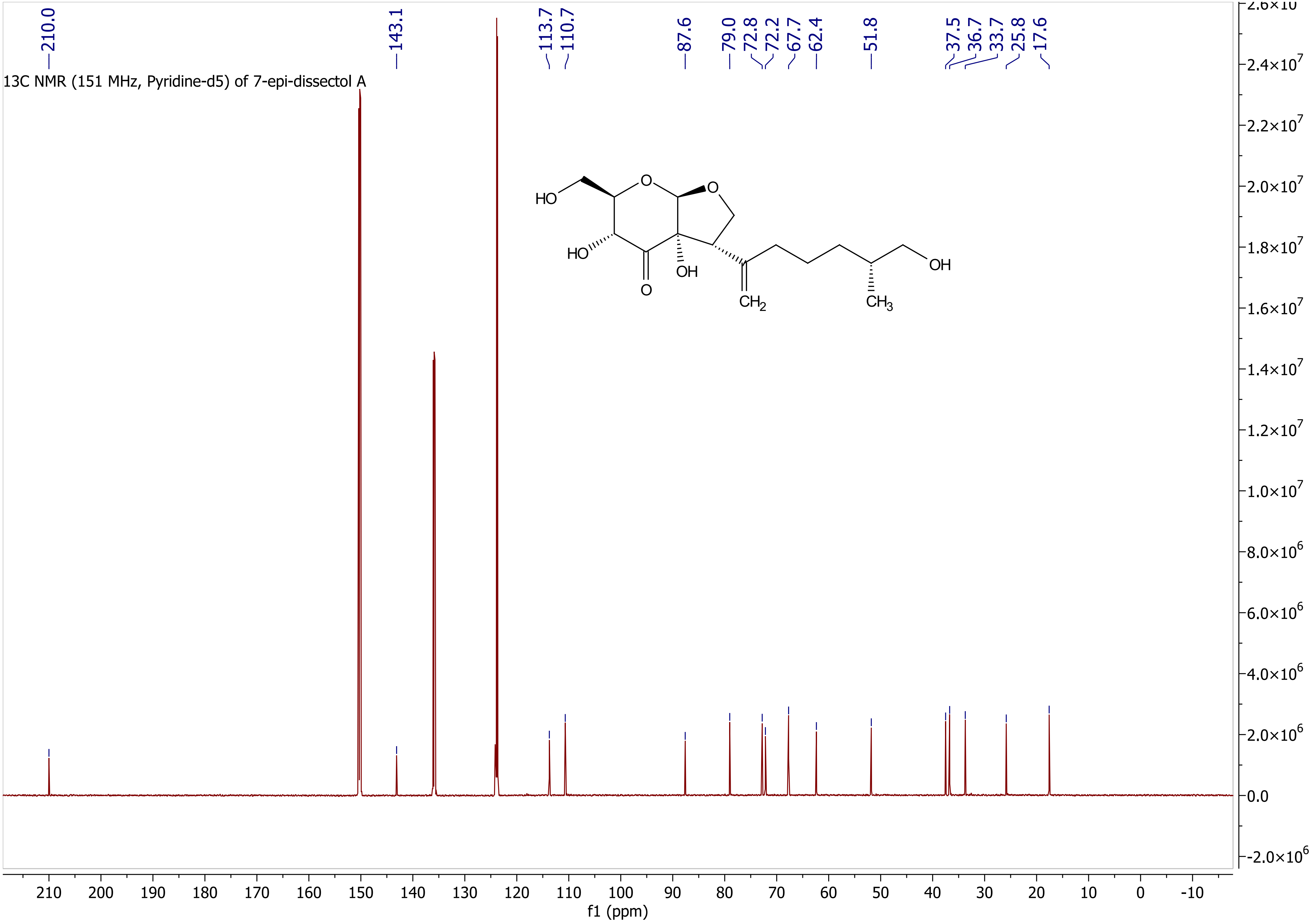

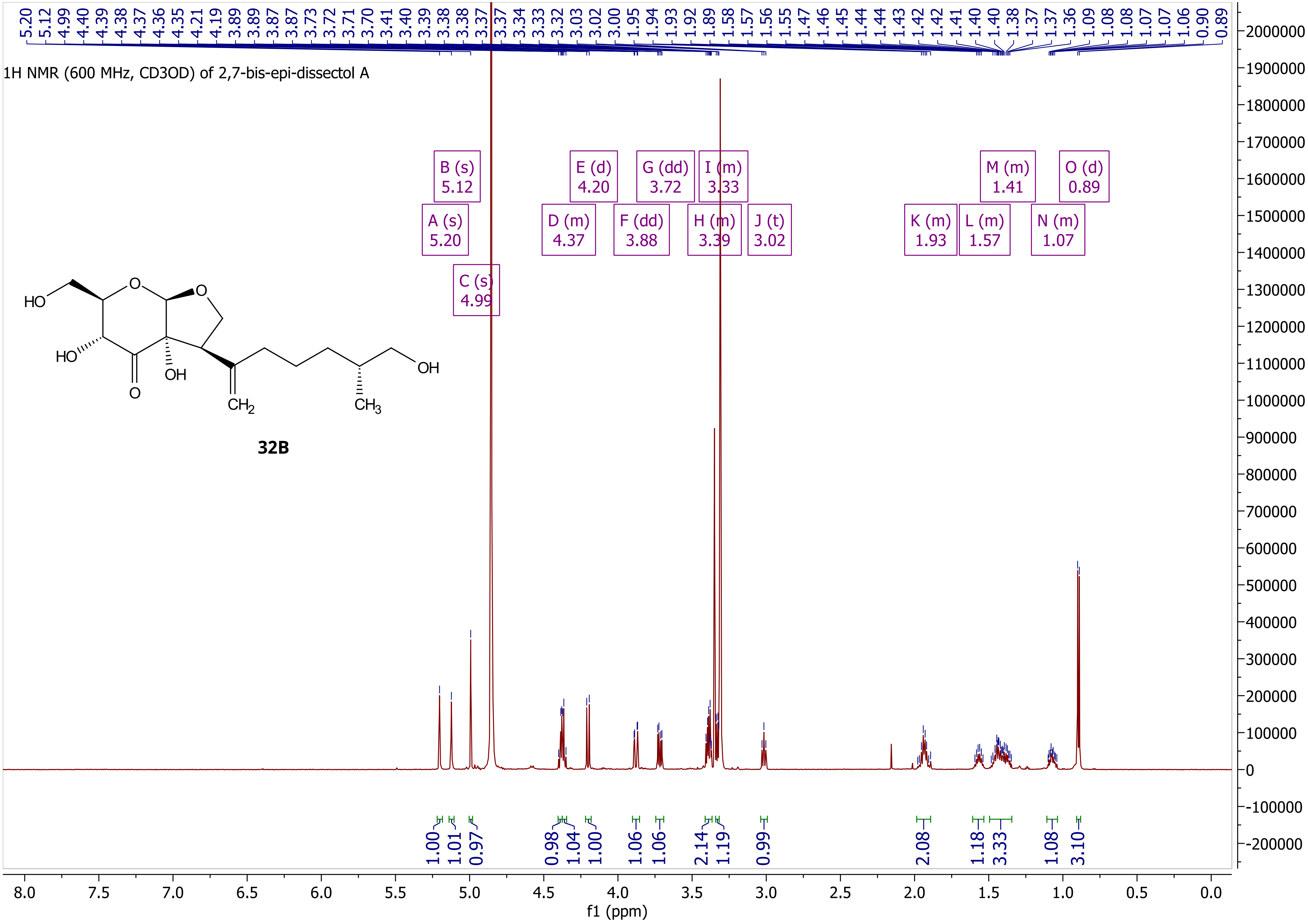
1H NMR (600 MHz, CD3OD) of 2,7-bis-epi-dissectol A
A (s) 5.20
B (s) 5.12
C (s) 4.99
D (m) 4.37
E (d) 4.20
F (dd) 3.88
G (dd) 3.72
H (m) 3.39
I (m) 3.33
J (t) 3.02
K (m) 1.93
L (m) 1.57
M (m) 1.41
N (m) 1.07
O (d) 0.89
HO
O
O
HO
OH
O
CH2
CH3
OH
32B
f1 (ppm)

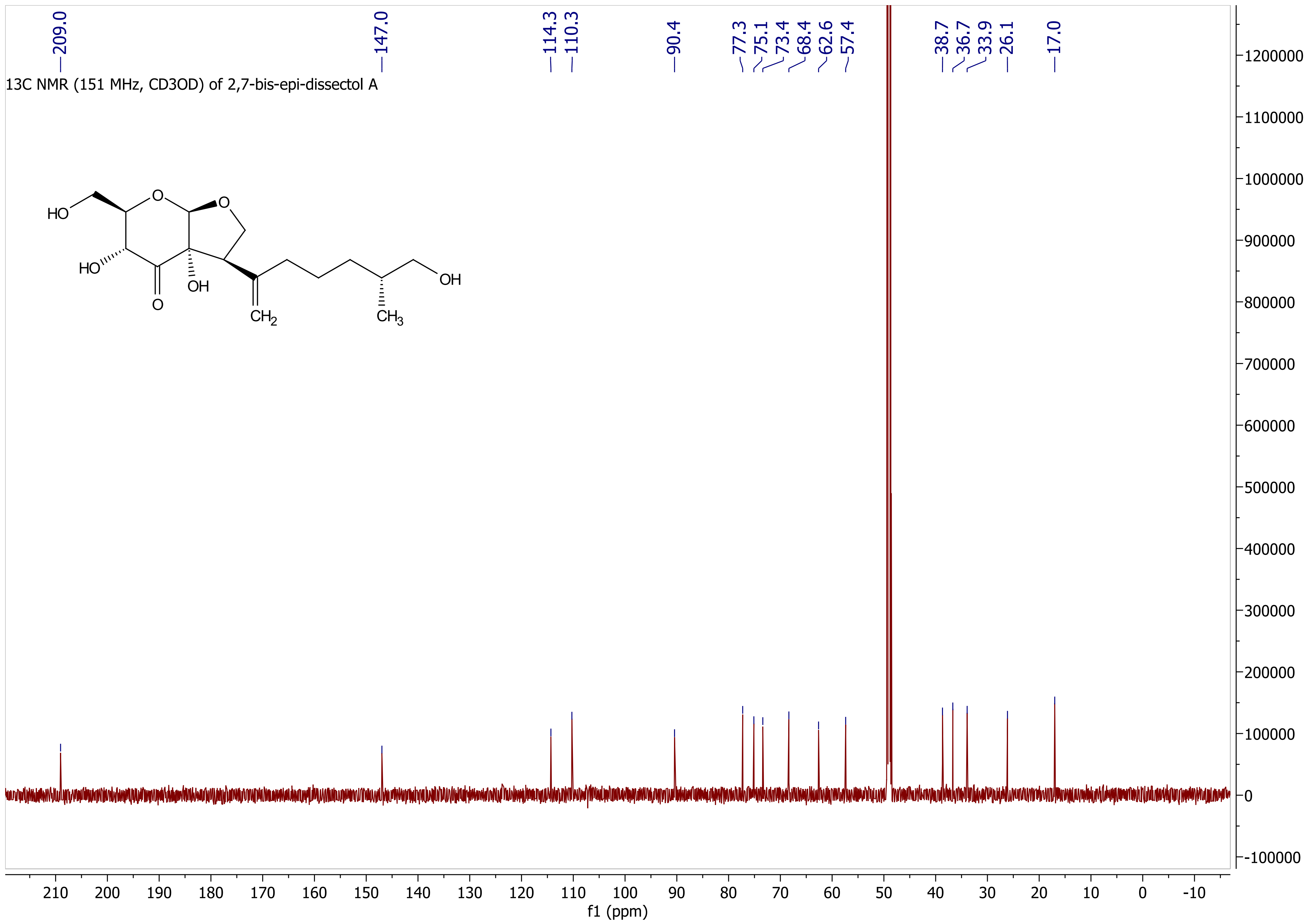

209.0
147.0
114.3
110.3
90.4
77.3
75.1
73.4
68.4
62.6
57.4
38.7
36.7
33.9
26.1
17.0
13C NMR (151 MHz, CD3OD) of 2,7-bis-epi-dissectol A
HO
O
O
HO
OH
O
CH2
CH3
OH
f1 (ppm)

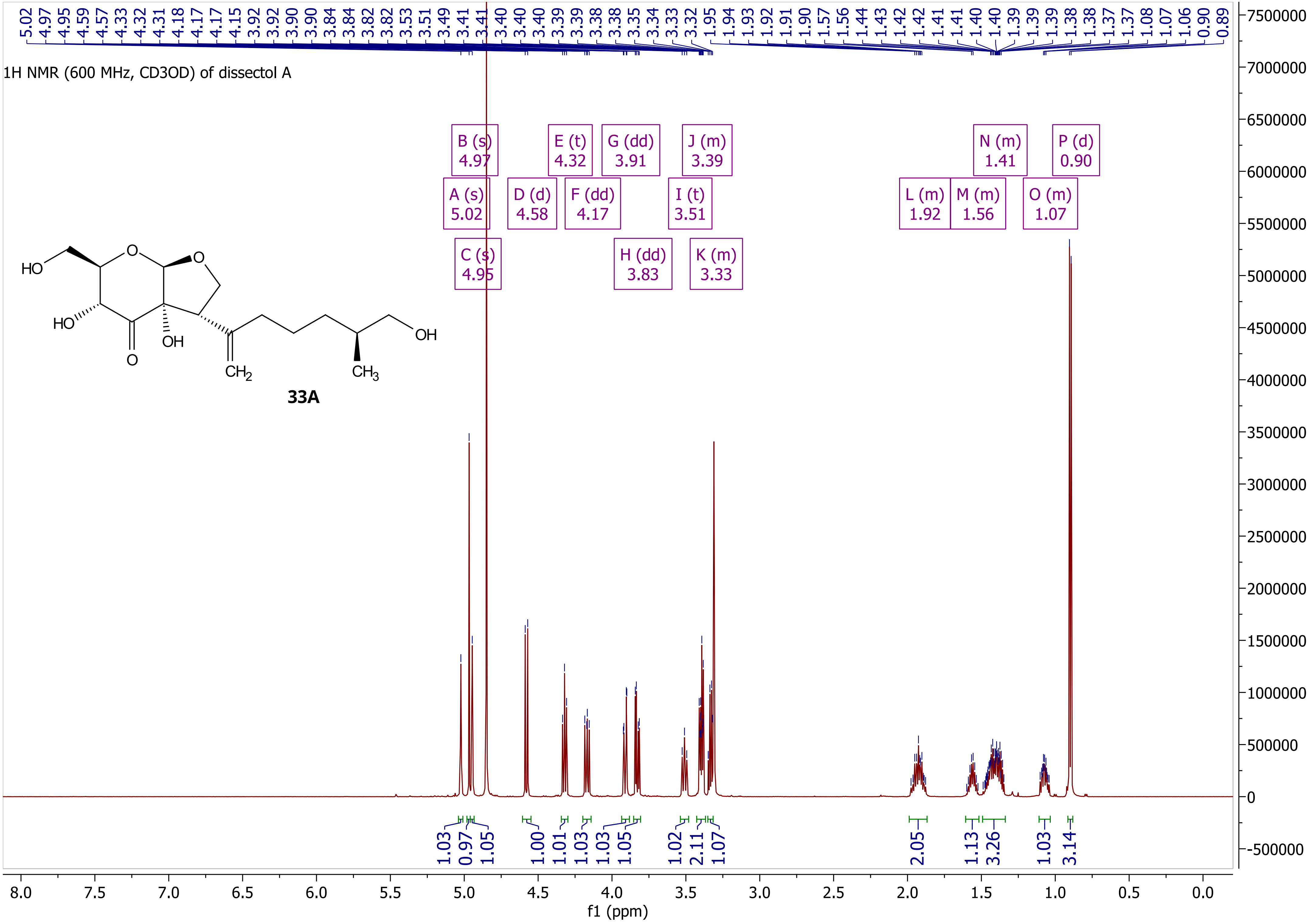

5.02 4.97 4.95 4.59 4.57 4.33 4.32 4.31 4.18 4.17 4.17 4.15 3.92 3.92 3.90 3.90 3.84 3.84 3.82 3.82 3.53 3.51 3.49 3.41 3.41 3.40 3.40 3.40 3.39 3.39 3.38 3.38 3.35 3.34 3.33 3.32 1.95 1.94 1.93 1.92 1.91 1.90 1.57 1.56 1.44 1.43 1.42 1.42 1.41 1.41 1.40 1.40 1.39 1.39 1.39 1.38 1.38 1.37 1.37 1.08 1.07 1.06 0.90 0.89
1H NMR (600 MHz, CD3OD) of dissectol A
B (s) 4.97
E (t) 4.32
G (dd) 3.91
J (m) 3.39
N (m) 1.41
P (d) 0.90
A (s) 5.02
D (d) 4.58
F (dd) 4.17
I (t) 3.51
L (m) 1.92
M (m) 1.56
O (m) 1.07
C (s) 4.95
H (dd) 3.83
K (m) 3.33
HO
O
O
HO
OH
O
OH
CH2
CH3
33A
1.03 0.97 1.05 1.00 1.01 1.03 1.03 1.05 1.02 2.11 1.07 2.05 1.13 3.26 1.03 3.14
8.0 7.5 7.0 6.5 6.0 5.5 5.0 4.5 4.0 3.5 3.0 2.5 2.0 1.5 1.0 0.5 0.0
f1 (ppm)
7500000 7000000 6500000 6000000 5500000 5000000 4500000 4000000 3500000 3000000 2500000 2000000 1500000 1000000 500000 0 -500000

13C NMR (151 MHz, CD3OD) of dissectol A

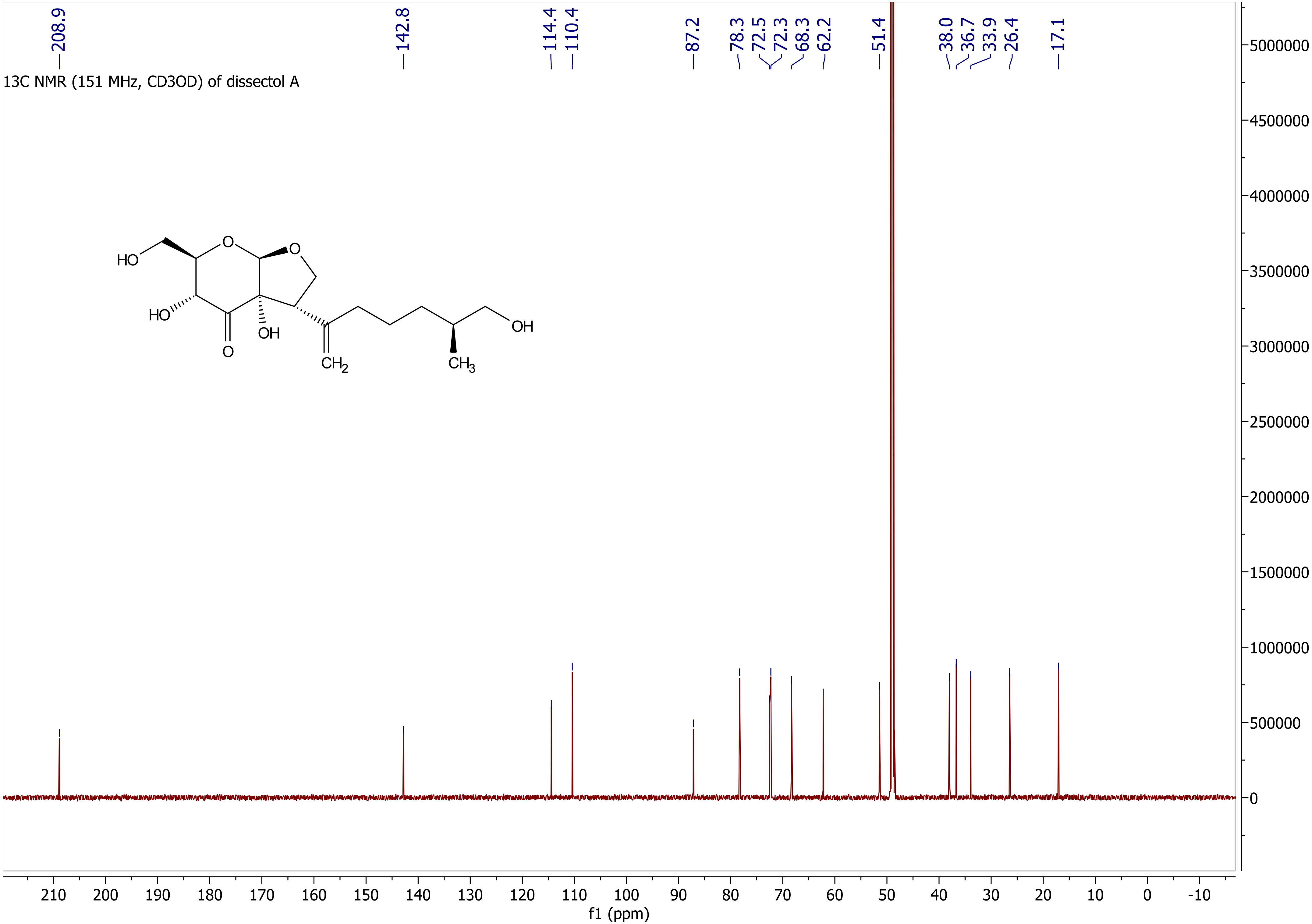

1H NMR (600 MHz, Pyridine-d5) of cissectol A (5)

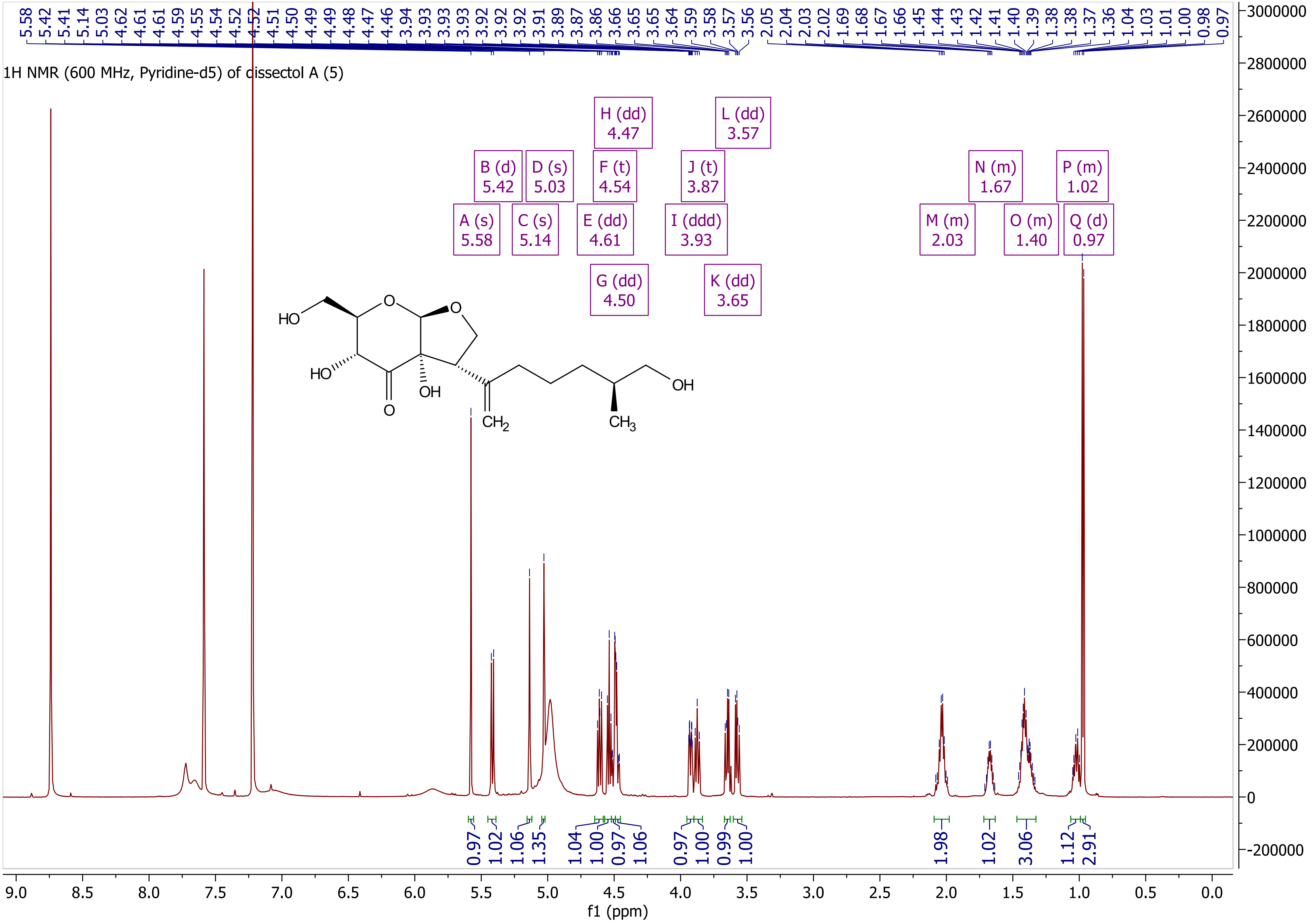

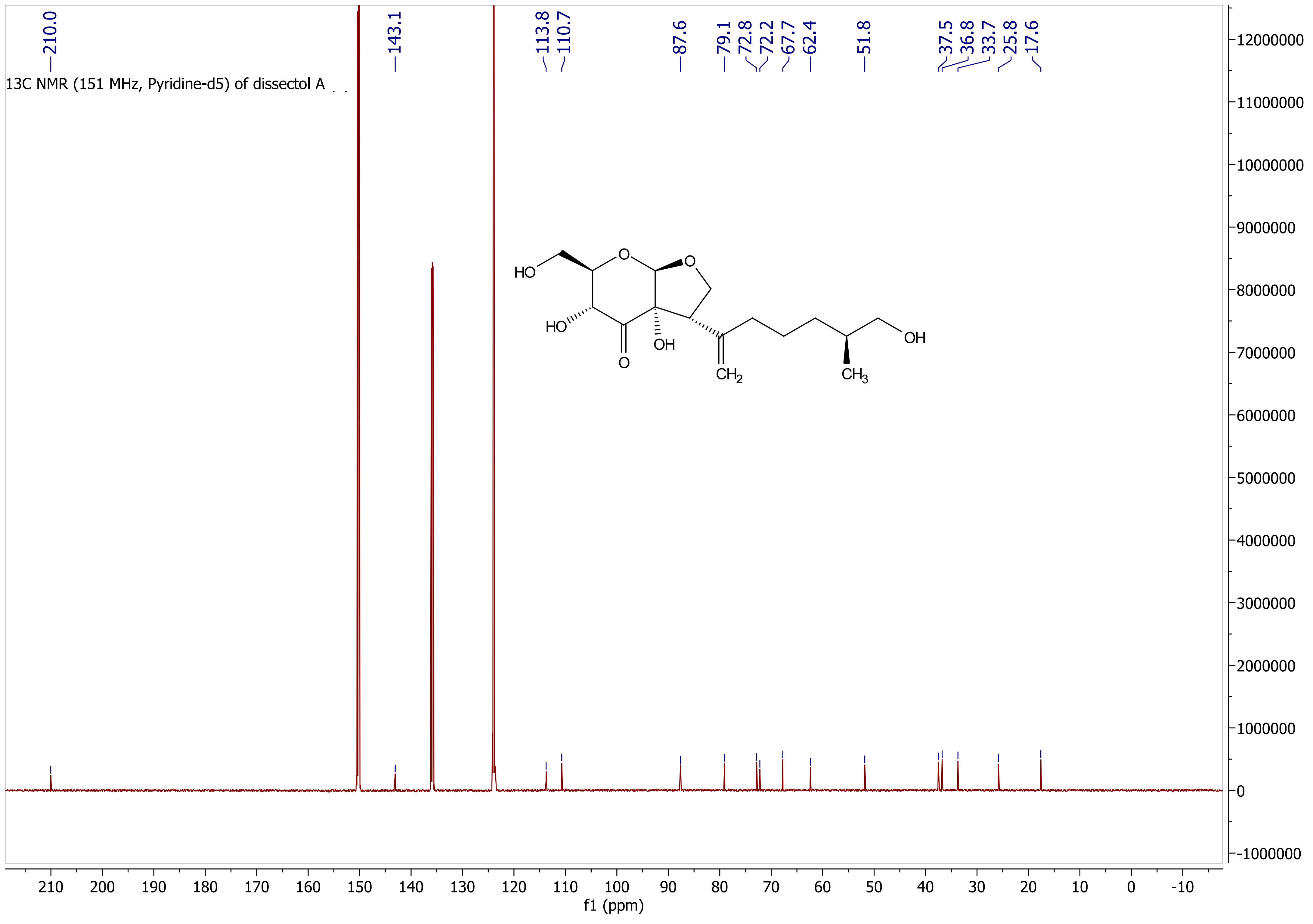

13C NMR (151 MHz, Pyridine-d5) of dissectol A
210.0
143.1
113.8
110.7
87.6
79.1
72.8
72.2
67.7
62.4
51.8
37.5
36.8
33.7
25.8
17.6
HO
O
O
HO
OH
O
OH
CH2
CH3
f1 (ppm)
210 200 190 180 170 160 150 140 130 120 110 100 90 80 70 60 50 40 30 20 10 0 -10
12000000
11000000
10000000
9000000
8000000
7000000
6000000
5000000
4000000
3000000
2000000
1000000
0
-1000000

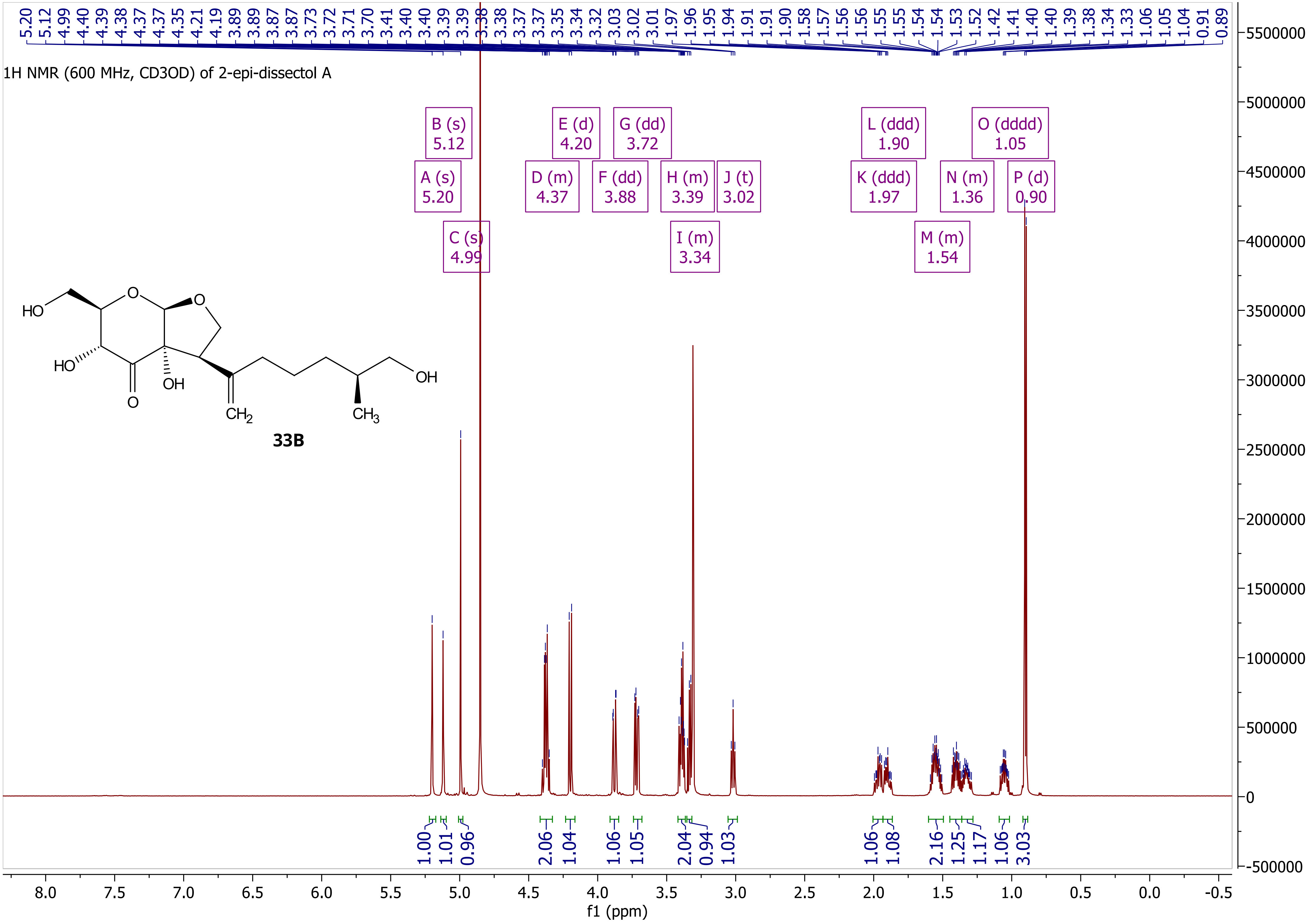
1H NMR (600 MHz, CD3OD) of 2-epi-dissectol A
5.20 5.12 4.99 4.40 4.39 4.38 4.37 4.37 4.35 4.21 4.19 3.89 3.89 3.87 3.87 3.73 3.72 3.71 3.70 3.41 3.40 3.40 3.39 3.39 3.38 3.38 3.37 3.37 3.35 3.34 3.32 3.03 3.02 3.01 1.97 1.96 1.95 1.94 1.91 1.91 1.90 1.58 1.57 1.56 1.56 1.55 1.55 1.54 1.54 1.53 1.52 1.42 1.41 1.40 1.40 1.39 1.38 1.34 1.33 1.06 1.05 1.04 0.91 0.89
A (s) 5.20
B (s) 5.12
C (s) 4.99
D (m) 4.37
E (d) 4.20
F (dd) 3.88
G (dd) 3.72
H (m) 3.39
I (m) 3.34
J (t) 3.02
K (ddd) 1.97
L (ddd) 1.90
M (m) 1.54
N (m) 1.36
O (dddd) 1.05
P (d) 0.90
HO
O
O
HO
OH
O
CH2
CH3
OH
33B
1.00 1.01 0.96 2.06 1.04 1.06 1.05 2.04 0.94 1.03 1.06 1.08 2.16 1.25 1.17 1.06 3.03
f1 (ppm)

13C NMR (151 MHz, CD3OD) of 2-epi-dissectol A

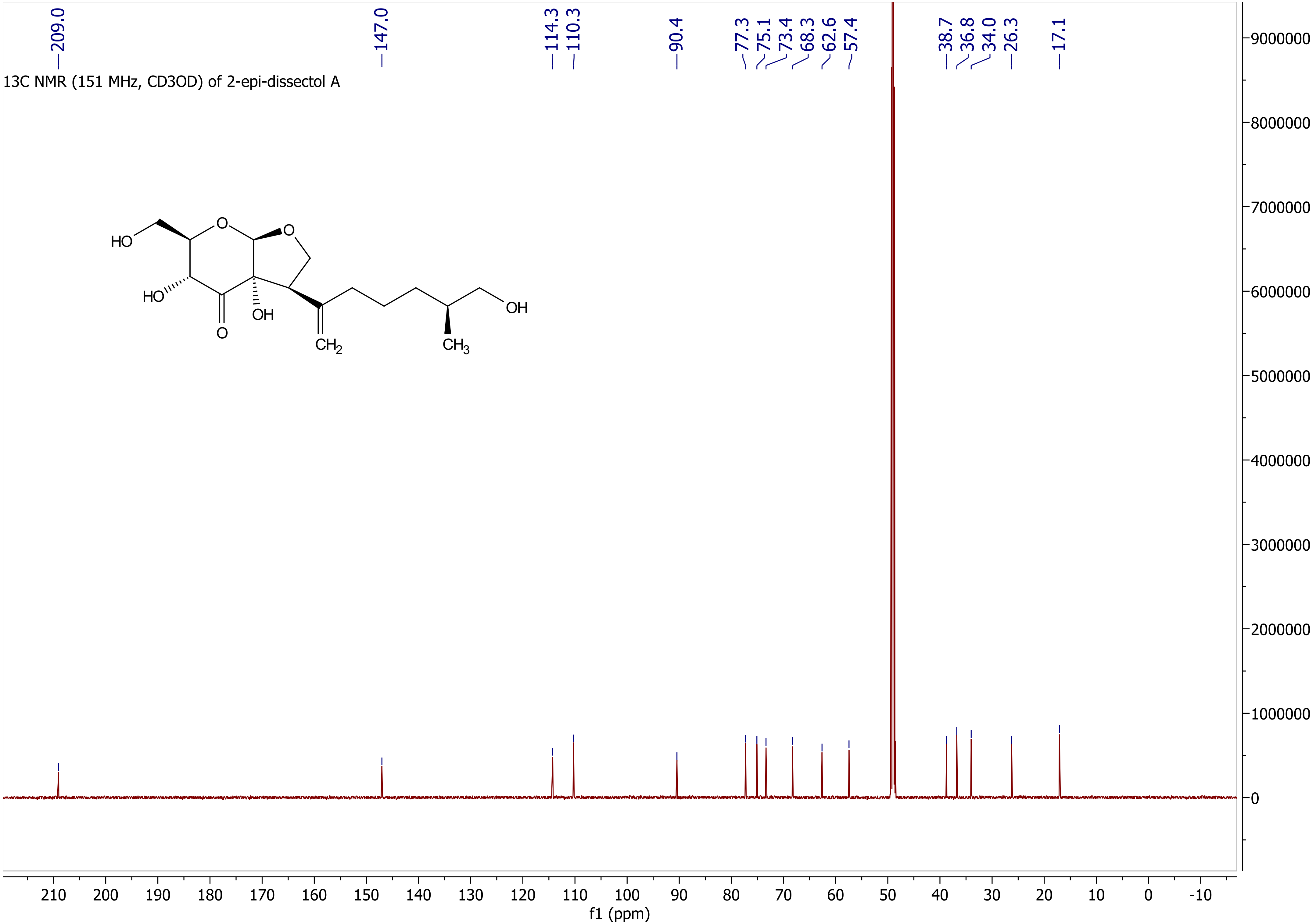

1H NMR comparison of natural isolate vs. synthetic dissectol A

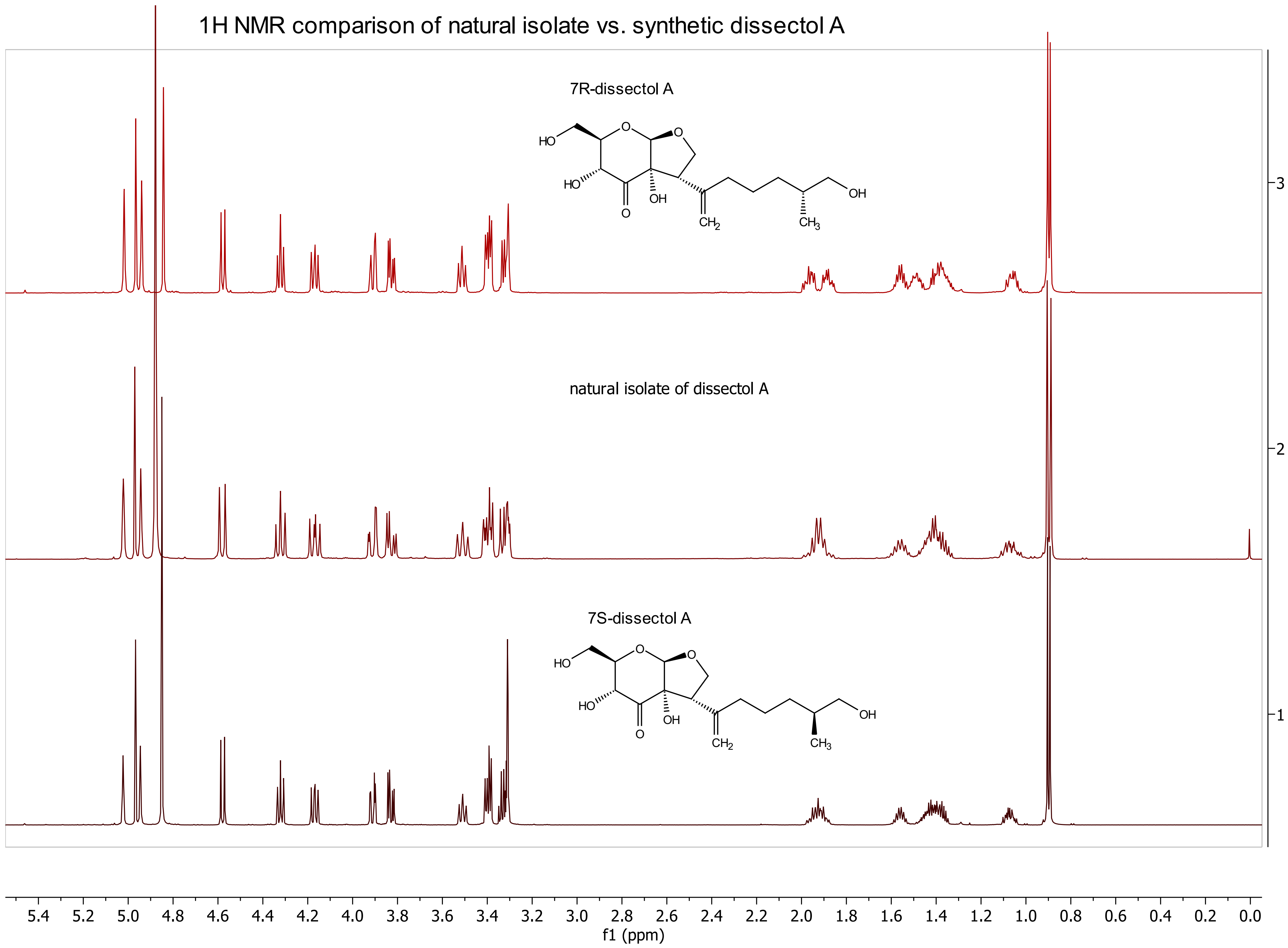

1H NMR comparison of natural isolate vs. synthetic dissectol A (zoom-in)

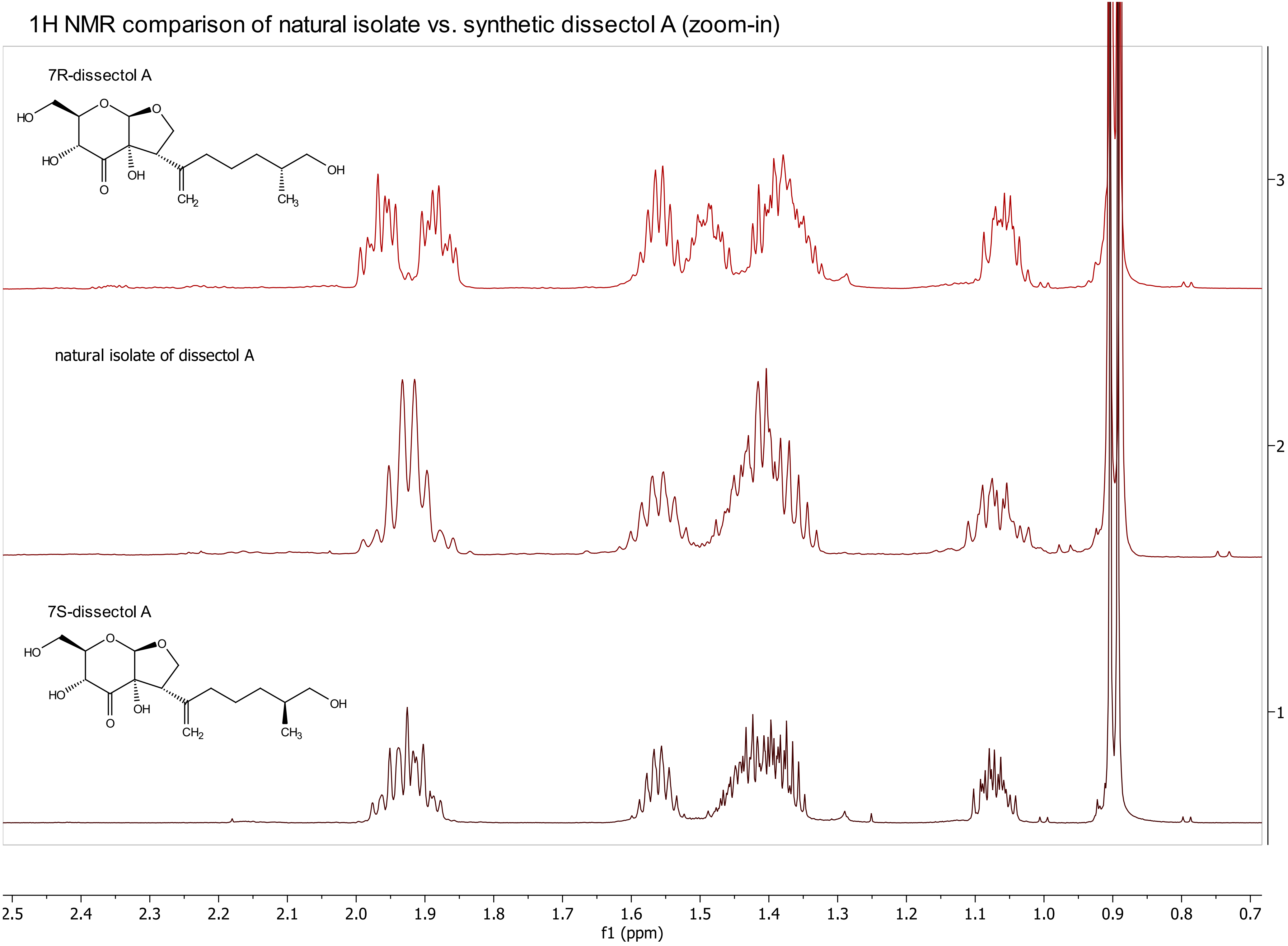

13C NMR comparison of natural isolate vs. synthetic dissectol A

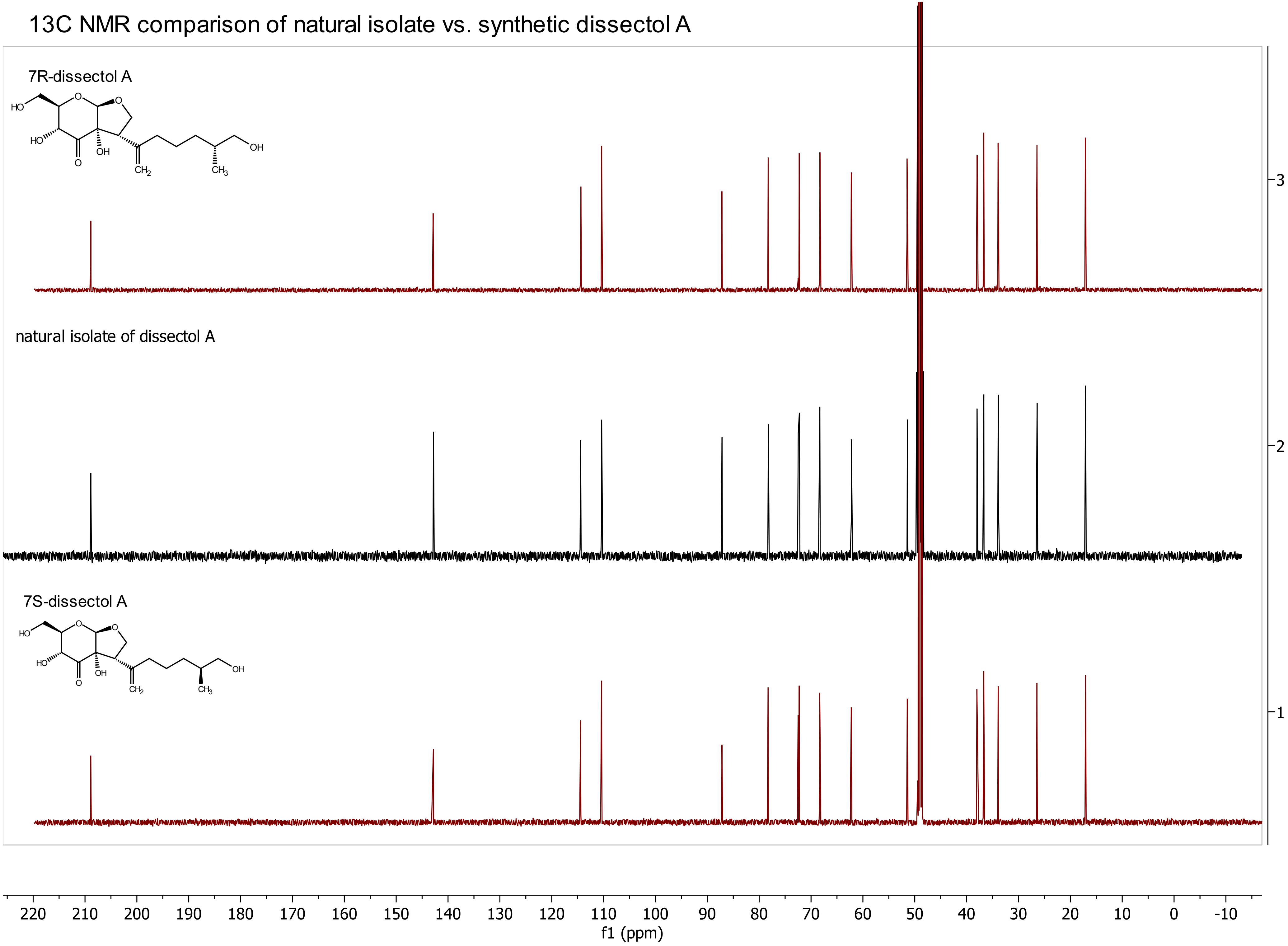

Supplement: SC-015-D4SC01745E-s001 [file SC-015-D4SC01745E-s001.pdf]
